# Mild and Chemoselective Carboxylic Acid Reduction Promoted by Borane Catalysis

**DOI:** 10.1002/anie.202207647

**Published:** 2022-09-21

**Authors:** Danijela Lunic, Nil Sanosa, Ignacio Funes‐Ardoiz, Christopher J. Teskey

**Affiliations:** ^1^ Institute of Organic Chemistry RWTH Aachen University Landoltweg 1 52074 Aachen Germany; ^2^ Department of Chemistry, Centro de Investigación en Síntesis Química (CISQ) Universidad de La Rioja Madre de Dios 53 26006 Logroño Spain

**Keywords:** Boron, Density-Functional Calculations, Hydroboration, Reduction, Selectivity

## Abstract

Although considerable advances have been made in developing chemoselective transformations of ubiquitous carboxylic acid groups, many challenges still exist. For instance, their selective reduction is problematic if both more nucleophilic and more electrophilic groups are present in the starting material. Here, we address this problem with a simple and mild protocol using bench‐stable reagents at ambient temperatures. This platform is able to tolerate a diverse range of functionality, leaving ketones, esters, nitro‐groups, olefins, nitriles and amides untouched. A combination of experimental and computational mechanistic experiments demonstrate that this reaction proceeds via hidden borane catalysis with small quantities of in situ generated BH_3_ playing a key role in the exquisite selectivity that is observed.

## Graphical abstract

A mild, chemoselective method for the reduction of carboxylic acids, in the presence of both more nucleophilic and more electrophilic functional groups is reported. Experiments and DFT calculations point to a mechanism driven by hidden borane catalysis.

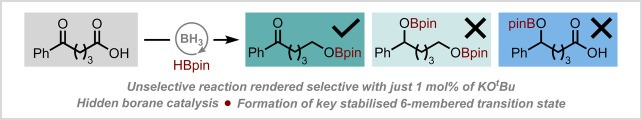

Chemoselective transformations allow the diversification of complex molecules without complex protecting group strategies.^[1]^ Carboxylic acids are prevalent in natural and unnatural molecules (Scheme 1a) and can be reduced to alcohols, another versatile functional group. However, as a result of being neither the most electrophilic, nor the most nucleophilic carbonyl group (Scheme 1b), methods that are entirely selective for carboxylic acid reduction in the presence of other functionalities are rare.[^2^, ^3^, ^4^]

**Scheme 1:**
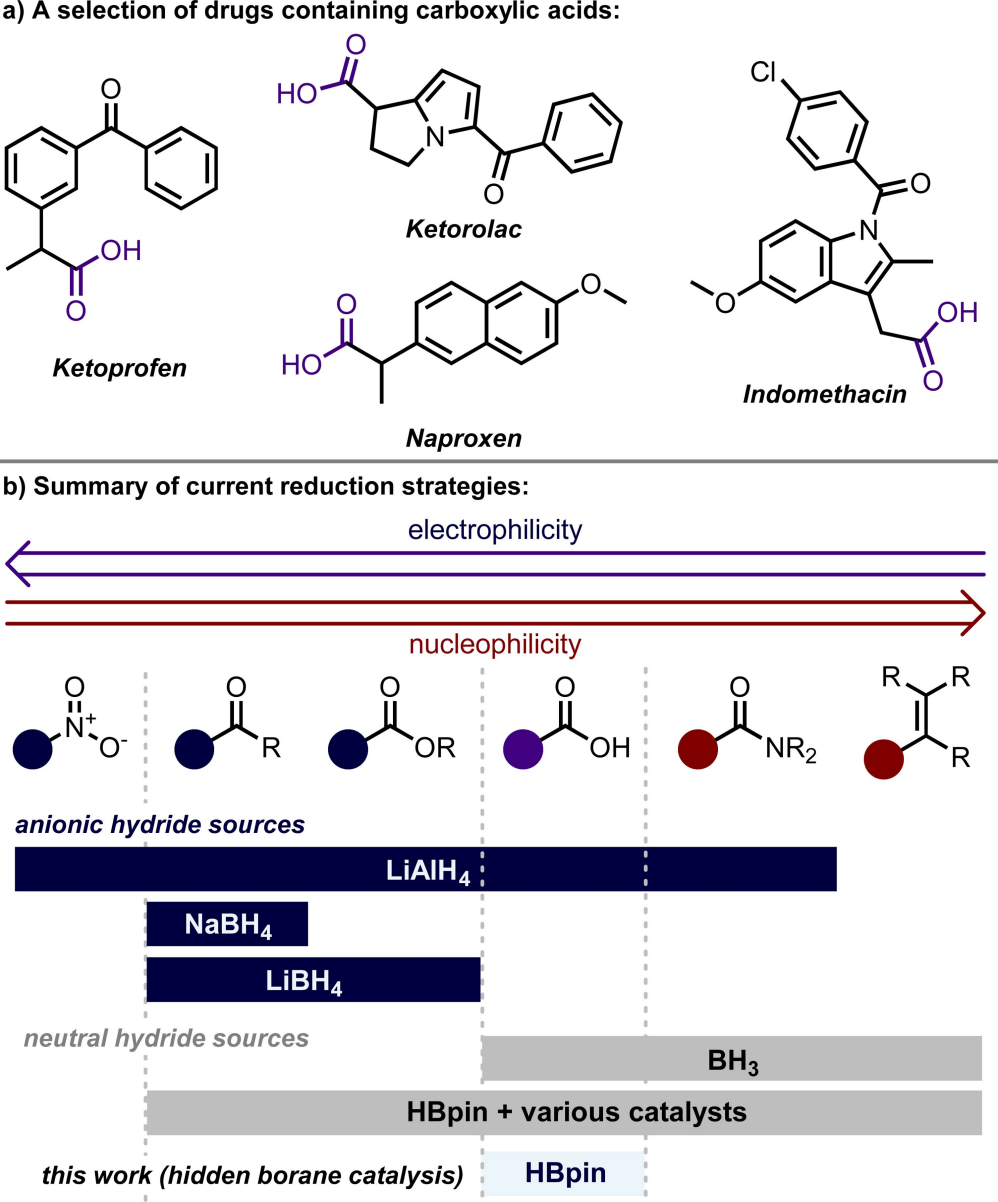
Overview of complex carboxylic acid containing molecules and methods for their reduction.

Strong anionic reducing agents, such as lithium aluminium hydride, can reduce carboxylic acids.^[5]^ However, these reactions are not selective when other functional groups such as ketones, esters, amides or nitro groups are present in the same molecule. Less nucleophilic anionic reducing agents, such as sodium or lithium borohydride, are no longer reactive towards carboxylic acids at ambient conditions but remain reactive towards more electrophilic functionality. The use of more forcing conditions, such as combining NaBH_4_ with various additives allows carboxylic acid reduction.[^6^, ^7^, ^8^, ^9^] However, these methods require the use of expensive and toxic reagents, long reaction times, high temperatures and most still remain unselective.

Some of these approaches may generate BH_3_ in situ, a neutral reducing agent which can select for carboxylic acid reduction in the presence of the more electrophilic carbonyl groups. However, borane also commonly reacts with more nucleophilic functionalities such as amides and alkenes. Furthermore, borane is unstable towards moisture and, as such, remains a non‐ideal reagent that does not display the level of selectivity required to selectively reduce carboxylic acids where more nucleophilic functionalities are also present in complex molecules.^[10]^

Pinacolborane, first reported in the early 1990s,^[11]^ is widely used for hydroboration reactions and is attractive due to its stability. In combination both with metal catalysts[^12^, ^13^] and without,[^14^, ^15^, ^16^] it is able to reduce a range of carbonyl groups from aldehydes through to amides. However, it is notable that most approaches to increase reactivity, for instance with amides, do not do so selectively making them unsuitable for complex substrates.[^17^, ^18^, ^19^] In this context, we recently reported a unique cobalt catalysed platform whereby the chemoselectivity of ketoacid hydroboration could be controlled by visible light.[^20^, ^21^] In line with our previous work,^[22]^ we suggested that the reactivity observed with carboxylic acid hydroboration was consistent with a Co^0^/Co^I^ catalytic cycle.^[23]^

Upon further mechanistic probes of this carboxylic acid selective hydroboration with in situ NMR (see Supporting Information for more details), we noted key features in the ^11^B NMR. Unlike with our previous report,^[23]^ we noted small peaks corresponding to BH_3_ (*δ*=−12 ppm) present throughout the reaction and BH_4_^−^ (*δ*=−42 ppm) during the induction phase. However, throughout the reaction, pinacolborane (28 ppm) remains the major boron species that is present, which rules out direct BH_3_ reduction of the carboxylic acid.

Pioneering recent work from Stephen Thomas's group on “hidden borane catalysis”,[^24^, ^25^, ^26^] has convincingly demonstrated that a number of reported catalytic systems for hydroboration—including purported catalysts based on metal complexes, hydride sources and bases—in fact, operate via promoted decomposition of pinacolborane into BH_3_ and BH_4_^−^ which are the active catalysts for hydroboration reactions.[^27^, ^28^] In addition, Jones and co‐workers have also demonstrated that carboxylic acids can react with HBpin to generate BH_3_.^[29]^ Cognizant of these facts, we decided to see if we could reproduce the selectivities of our cobalt catalysed reaction with these borane species.

Replacing HCo[PPh(OEt)_2_]_4_ (5 mol%) (Table 1, entry 1) with NaBH_4_ (5 mol%) resulted in slightly more over‐reduction (and unreacted starting material), though notably no ketone reduction product **3 a** (Table 1, entry 2), with ratios approaching the cobalt conditions.^[30]^ Next, upon switching to BH_3_⋅DMS (5 mol%), we observed exclusively product **2 a**, though with some slight mass loss during the reaction (Table 1, entry 3). Interestingly, use of a different hydride source, BNAH (1‐benzyl‐1,4‐dihydronicotinamide)—a simplified mimic of NADH—which is also unable to directly interact with the substrate, led to very similar ratios of products (Table 1, entry 4) to the cobalt and NaBH_4_ examples. Notably, as an organic hydride source, this may serve as a more soluble and functional group tolerant initiator.

**Table 1:** Exploration of promoters for acid selective hydroboration of **1 a**.

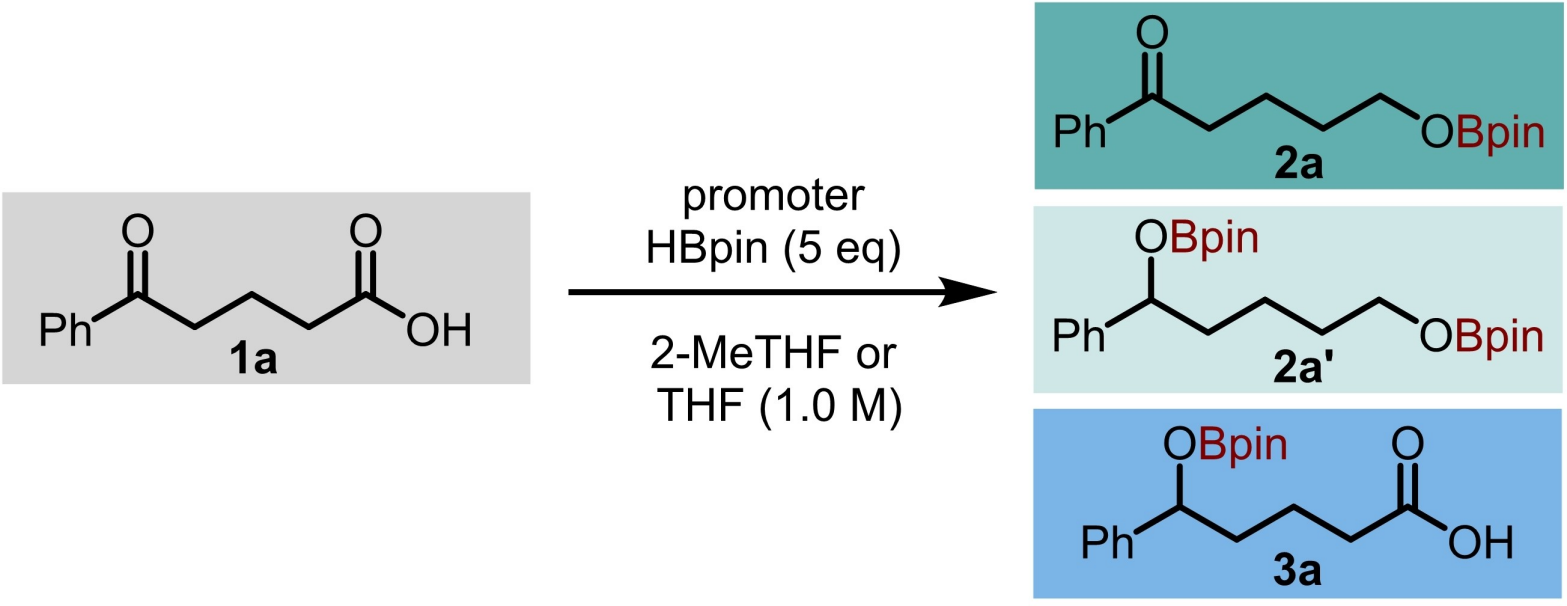			
Entry	Conditions	Yield [%]	Selectivity (**2 a:2 a′:3 a**)
1	HCo[PPh(OEt)_2_]_4_(5 mol%)	94	72 : 17 : 5
2	NaBH_4_ (5 mol%)	84	63 : 21:0
3	BH_3_⋅DMS (5 mol%)	75	75 : 0 : 0
4	BNAH (5 mol%)	82	62 : 20:0
5^[a]^	KO^*t*^Bu (1 mol%)	92	80 : 12:0
6^[a]^	KO^*t*^Bu (1 mol%), 0.1 M	0^[b]^	0 : 0 : 0
7	Without promoter	80	17 : 30 : 33

[a] Reaction carried out with 6 eq. of HBpin. [b] 85 % of starting material remaining.

Finally, we elected to see if one of the species previously reported to promote decomposition of pinacolborane would be more effective due to a slow release of catalytic species. With 1 mol% of KO^*t*^Bu in THF‐d_8_, we observed formation of carboxylic acid reduction product **2 a** in high yield (Table 1, entry 5). When more diluted conditions were applied (Table 1, entry 6), only starting material was recovered, demonstrating the importance of highly concentrated solution for the reaction to occur. Finally, in the absence of the catalyst and any promoter (Table 1, entry 7),^[20]^ poor selectivity is observed, with all products obtained in similar proportions. The stability of all reagents, ease of reaction set‐up and exquisite selectivity encouraged us to investigate further the reaction scope of this hydroboration protocol (Scheme 2).

**Scheme 2:**
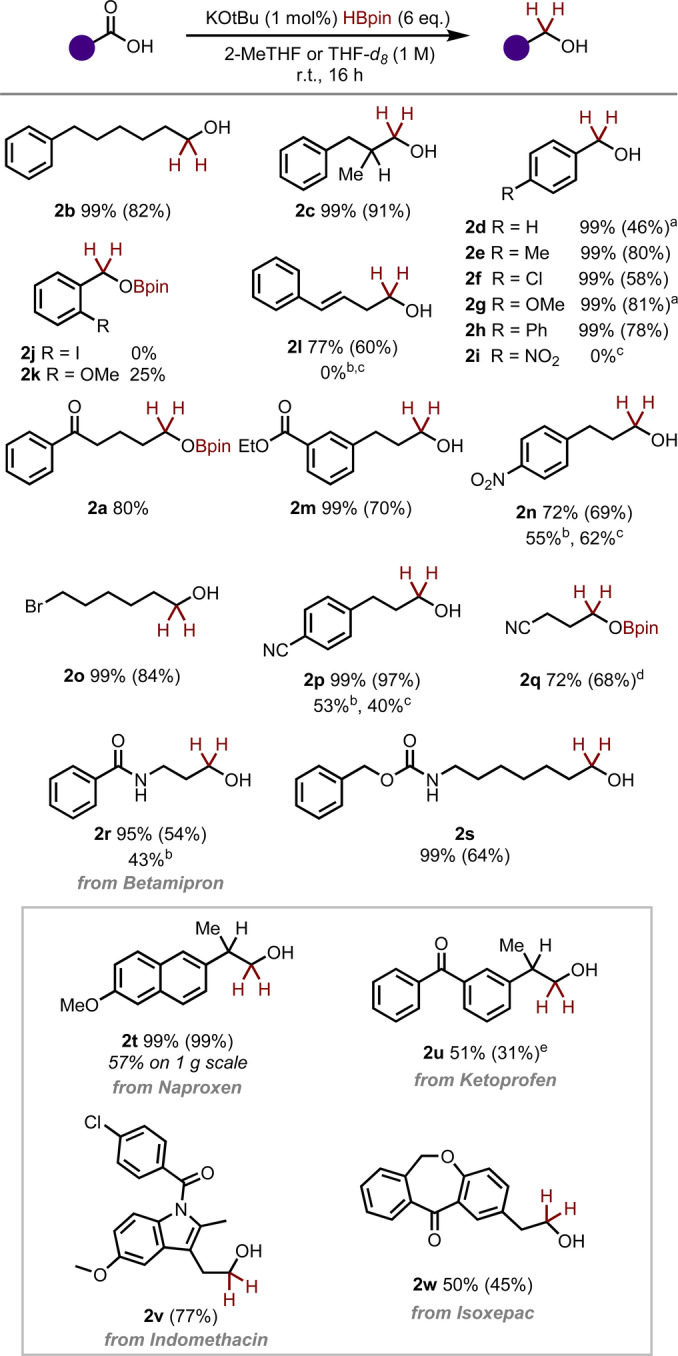
Substrate scope of carboxylic acid selective hydroboration. Yields determined by ^1^H NMR are given compared to CHBr_3_ or CH_2_Br_2_ as the internal standard. Isolated yields are given in parenthesis. [a] Reaction carried out with 7 eq. of HBpin. [b] The following reaction conditions were used: BH_3_DMS (1.3 eq), THF (0.5 M), 6 h, 0 °C‐r.t. [c] The following reaction conditions were used: BH_3_DMS (2.5 eq), THF‐d_8_ (0.5 M), 6 h, 0 °C‐r.t. [d] Isolated as a mixture with pinacol. [e] Reaction carried out with 5 mol% of KOtBu.

Based on the most simple conditions we had developed with potassium *tert*‐butoxide as the reaction promoter and 2‐MeTHF or THF as solvent, simple aliphatic carboxylic acids were hydroborated in excellent yield (**2 b**), including with α‐substitution (**2 c**). Aromatic examples were similarly reactive with substitution at the *para*‐position resulting in complete conversion to corresponding alcohols in most cases (**2 d**–**2 h**). Notably, however, the *para*‐nitro substituted example **2 i** returned only starting material. Similarly, o*rtho*‐iodinated benzoic acid, **2 j** did not give the desired product, however, 25 % of *ortho*‐methoxy substituted product **2 k** was observed after 14 hours, with the remainder of the mass‐balance as starting material. Styryl acetic acid underwent smooth hydroboration under the reaction conditions, with the double bond remaining untouched, to yield product **2 l**.

Next, we turned to substrates with other functional groups which may potential be susceptible to reductive conditions. As previously discussed, ketoacid substrate **1 a** was chemoselectively reduced to yield product **2 a**. A range of other electrophilic functionalities including esters (**2 m**), nitro groups (**2 n**) simple alkyl bromides (**2 o**) and aromatic and aliphatic nitriles (**2 p** and **2 q**) also remain unreacted.

Amide containing substrate **1 r** (biologically active drug Betamipron) underwent hydroboration exclusively at the carboxylic acid carbonyl, with the amide remaining untouched despite reports of more electrophilic boranes reducing such functionalities at ambient temperatures and, similarly, carbamates were fully tolerated under these conditions (**2 s**).

Finally, we sought to demonstrate that our method is applicable to more complex settings. Four drugs, Naproxen, Ketoprofen, Indomethacin and Isoxepac were selectively reduced to give products **2 t**–**2 w**, demonstrating tolerance of ketone, amide and aryl halide functionality. Enantiopure Naproxen gave the reduced product **2 t** in 99 % e.e. revealing that no erosion of enantiopurity at the chiral centre occurs. In the cases of Ketoprofen and Isoxepac, the lower yield was only as a result of remaining starting material rather than poor selectivity. Upon scaling up the reaction protocol, we obtained product **2 t** in 57 % yield with one gram of Naproxen starting material.

To show the advancement of this method compared with the classical method for acid reduction, several substrates were subjected to reduction conditions with stoichiometric BH_3_ ⋅ DMS. The yields obtained from starting materials **1 n**, **1 p** and **1 r** were significantly lower than with our method, regardless of the number of equivalents of borane used. Interestingly, alkene containing substrate **1 l** yielded only a complex mixture of products with none of the desired reduction product **2 l** as we had obtained in good yield with our newly developed conditions.

The primary limitation with this method is the requirement for a relatively high concentration. As such, starting materials that were poorly soluble displayed limited reactivity and dilution significantly decreased the reaction rate, as shown in Table 1 (entry 6). However, we demonstrated that different promoters could be used in combination with different solvents which should broaden the utility of this reaction (Table 1, entry 4 and more details in the Supporting Information).

It has been previously demonstrated by Thomas et al. that, whilst TMEDA (tetramethylethylenediamine) does not interact with HBPin, it forms an adduct with BH_3_.^[25]^ As a result, if the reaction is inhibited upon addition of TMEDA, this is potentially indicative of BH_3_ being the catalytic species. In our case, only starting material **1 a** was recovered (60 %) upon addition of one equivalent of TMEDA (Scheme 3), suggesting that the active catalytic species could be BH_3_. Notably, TMEDA inhibits the reaction with other promoters as well with no product formed.

**Scheme 3:**
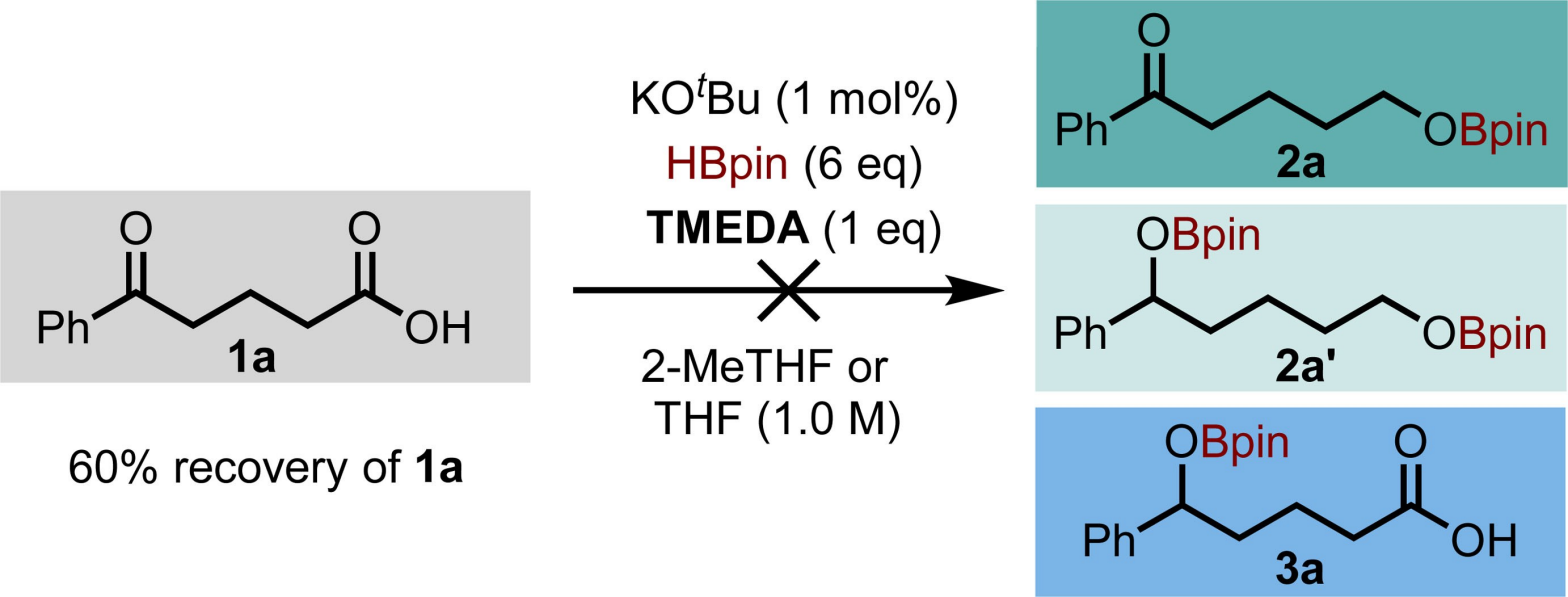
Reaction inhibition upon addition of TMEDA.

In order to shed further light into the origin of the enhanced selectivity when HBPin is activated, we carried out a DFT computational study at the CPCM(THF) M06‐2X/6‐311++G(3d,2p)//B3LYP/6‐31+G(d) level of theory (see computational details in the Supporting Information for further details) on the initial hydroboration step. We first analysed the direct attack of HBPin to the substrate **1 a**. Interestingly, the direct ketone hydroboration is favoured by 3.9 kcal mol^−1^ in substrate **1 a** and 2.3 kcal mol^−1^ in the boronic ester after initial OH activation by HBPin (see Figure S9). Also, hydroboration mediated by nucleophilic attack of BH_4_^−^ was found to favour ketone reduction (Figure S10). H(O^t^Bu)BPin^−^ mediated reduction was also calculated but, as for BH_4_^−^, the direct H^−^ transfer is favoured on the ketone site. However, these pathways could indicate a possible source of the minor amount of ketone overreduction which is observed. In general, the predicted selectivity appears consistent with the non‐promoted reaction (Table 1, entry 7) however it remained unclear how the promoters improve this selectivity. We therefore considered that in situ formation of BH_3_ may be key for the reaction selectivity, as BH_3_ ⋅ DMS catalysed selectively the reduction of the acid functional group (see Table 1, entry 3).

Activation of the OH group from the acid is both kinetically and thermodynamically accessible when BH_3_ is generated in the reaction media (Figure 1). The free energy barrier is only 10.6 kcal mol^−1^, 11.1 kcal mol^−1^ lower than HBPin mediated activation. We then explored the sequential activation of the remaining B−H bonds, to finally form intermediate **IV** through small free energy barriers in a very exergonic process (‐51.9 kcal mol^−1^). From intermediate **IV**, direct insertion of highly concentrated HBPin was explored.

**Figure 1:**
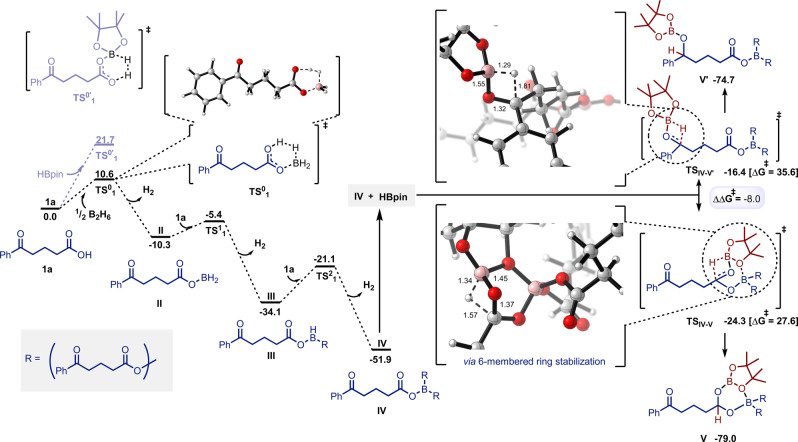
Free energy profile of BH_3_ mediated activation of substrate **1 a** and selective hydroboration with HBpin. Free energies in kcal mol^−1^ and bond lengths in Å.

Direct insertion of the H‐Bpin bond into the C=O bond of the ketone is highly strained due to the 4‐member ring transition state (**TS_IV‐V′_**). Thus, the free energy barrier is very high (35.6 kcal mol^−1^). In contrast, C=O hydroboration on the acid site is 8.0 kcal mol^−1^ lower than the ketone site due to the additional stabilisation of the incoming HBPin by the adjacent boron ester species (**TS_IV‐V_**). The free energy barrier is 27.6 kcal mol^−1^, which explains the observed reaction selectivity under BH_3_ catalytic activation. Finally, the resulting intermediates (**V** and **V′**) are very stable, so the back‐reaction is prevented and the selectivity is already determined at this step.

Taken together with our experimental observations and previous literature reports, this demonstrates that very low concentrations of BH_3_ are able to promote carboxylic acid reduction with pinacolborane, even in the presence of ketones. This is in stark contrast to the reactions without BH_3_ or a suitable promoter which are highly unselective.

In conclusion, we have shown through mechanistic investigations that carboxylic acid selective hydroboration, promoted by a number of different species, is a uniquely mild method for the chemoselective reduction of carboxylic acids. This significantly expands the scope of ‘hidden borane’ catalysed reactions, demonstrating the ubiquity of this concept and rendering transition metal catalysed processes obsolete for this transformation. Our method can be applied to complex drug molecules and tolerates a wide variety of other functional groups.

## Conflict of interest

The authors declare no conflict of interest.

## Supporting information

As a service to our authors and readers, this journal provides supporting information supplied by the authors. Such materials are peer reviewed and may be re‐organized for online delivery, but are not copy‐edited or typeset. Technical support issues arising from supporting information (other than missing files) should be addressed to the authors.

Supporting Information

## Data Availability

The data that support the findings of this study are available in the supplementary material of this article.
