# Supplementary material for: Mild and Chemoselective Carboxylic Acid Reduction Promoted by Borane Catalysis
Source: Angew Chem Int Ed Engl. 2022 Sep 21;61(43):e202207647. doi: 10.1002/anie.202207647 (PMC9825922; doi:10.1002/anie.202207647)
Supplement: Supplementary file 1 — Supporting Information [file ANIE-61-0-s001.pdf]

Angewandte Chemie
Eine Zeitschrift der Gesellschaft Deutscher Chemiker

Supporting Information

Mild and Chemoselective Carboxylic Acid Reduction Promoted by Borane Catalysis

D. Lunic, N. Sanosa, I. Funes-Ardoiz, C. J. Teskey**

Mild and Chemoselective Carboxylic Acid Reduction Promoted by Borane Catalysis

Danijela Lunic,[a] Nil Sanosa,[b] Ignacio Funes-Ardoiz*,[b] and Christopher J. Teskey*,[a]

[a]Institute of Organic Chemistry, RWTH Aachen University, Landoltweg 1, 52074 Aachen, Germany

[b]Department of Chemistry, Centro de Investigación en Síntesis Química (CISQ), Universidad de La Rioja, Madre de Dios 53, 26006 Logroño, Spain.

Table of Contents

1. Materials and Methods 1

2. Selected optimizations of reaction conditions 2

2.1 Effect of EDG and EWG on the reaction outcome 4

3. Synthesis of starting materials 5

Ethyl 3-(3-oxopropyl) benzoate (1m') 5

3-(3-(ethoxycarbonyl)phenyl) propanoic acid (1m) 5

3-(4-nitrophenyl) propanal (1n') 6

3-(4-nitrophenyl) propionic acid (1n) 6

3-(p-cyanophenyl) propionaldehyde (1p') 6

3-(4-cyanophenyl)propionic acid (1p) 6

Synthesis of 3-cyanopropanoic acid (1q) 7

4. Reduction reactions products 8

4.1. General procedure 1 8

1-phenyl-5-((4,4,5,5-tetramethyl-1,3,2-dioxaborolan-2-yl)oxy)pentan-1-one (2a) and 9

2,2'-((1-phenylpentane-1,5diyl)bis(oxy))bis(4,4,5,5-tetramethyl-1,3,2-dioxaborolane) (2a').. 9

6-phenyl-1-hexanol (2b) 9

2-methyl-3-phenylpropanol (2c) 9

Benzyl alcohol (2d) 9

4-methylbenzyl alcohol (2e) 10

4-chlorobenzyl alcohol (2f) 10

4-methoxybenzyl alcohol (2g) 10

Biphenyl-4-yl-methanol (2h) 10

2-((2-methoxybenzyl)oxy)-4,4,5,5-tetramethyl-1,3,2-dioxaborolane (2k) 10

(3E)-4-phenyl-3-buten-1-ol (2l) 12

Ethyl 3-(3-hydroxypropyl) benzoate (2m) 12

3-(4-nitrophenyl)-1-propanol (2n) 12

1-bromo-6-hexanol (2o) 12

4-(3-Hydroxypropyl) benzonitrile (2p) 13

4-((4,4,5,5-tetramethyl-1,3,2-dioxaborolan-2-yl)oxy)butanenitrile (2q) 13

N-(3-hydroxypropyl) benzamide (2r) 13

Benzyl(7-hydroxyheptyl)carbamate (2s) and 14

benzyl(7-((4,4,5,5(--tetramethyl-1,2,3-dioxaborolan-2-yl)oxy)heptyl) carbamate (2s') 14

2-(6-methoxy-2-naphthyl)-propyl alcohol (2t) 14

2-(3-benzoylphenyl)-propanol (2u) 14

(4-chlorophenyl)(3-(2-hydroxyethyl)-5-methoxy-2-methyl-1H-indol-1-yl)methanone (2v)... 15

2-(6,11-dihydro-11-oxodibanzoxepin-2-yl) ethanol (2w) 15
4.2. Reaction scale-up 16
4.3. Enantioselectivity of the reaction 16
4.4. General procedure 2 – Reduction of selected carboxylic acids with stoichiometric BH_3·DMS 18
3-(4-nitrophenyl)-1-propanol (2n-a and 2n-b) 19
4-(3-Hydroxypropyl) benzonitrile (2p-a and 2p-b) 20
N-(3-hydroxypropyl)benzamide (2r-a) 22
5. Mechanistic studies 23
5.1 Reaction with TMEDA 23
5.2. NMR monitoring of the reaction 23
5.3 NMR monitoring of reduction reactions promoted by borane catalysis 29
6. Computational details 35
Energy Profiles 35
XYZ Coordinated and Energies of the Calculated Species 39
7. Copies of NMR spectra 62
1-phenyl-5-((4,4,5,5-tetramethyl-1,3,2-dioxaborolan-2-yl)oxy)pentan-1-one (2a) and 62
2,2'-((1-phenylpentane-1,5diyl)bis(oxy))bis(4,4,5,5-tetramethyl-1,3,2-dioxaborolane) (2a') 62
6-phenyl-1-hexanol (2b) 63
2-methyl-3-phenylpropanol (2c) 64
Benzyl alcohol (2d) 65
4-methyl benzyl alcohol (2e) 66
4-chlorobenzyl alcohol (2f) 67
4-methoxybenzyl alcohol (2g) 68
Biphenyl-4-yl-methanol (2h) 69
2-((2-methoxybenzyl)oxy)-4,4,5,5-tetramethyl-1,3,2-dioxaborolane (2k) 70
(3E)-4-phenyl-3-buten-1-ol (2l) 71
Ethyl 3-(3-hydroxypropyl) benzoate (2m) 72
3-(4-nitrophenyl)-1-propanol (2n) 73
1-bromo-6-hexanol (2o) 74
4-(3-Hydroxypropyl) benzonitrile (2p) 75
4-((4,4,5,5-tetramethyl-1,3,2-dioxaborolan-2-yl)oxy)butanenitrile (2q) 76
N-(3-hydroxypropyl) benzamide (2r) 77
Benzyl(7-hydroxyheptyl)carbamate (2s) and benzyl(7-((4,4,5,5(--tetramethyl-1,2,3-dioxaborolan-2-yl)oxy)heptyl) carbamate (2s') 78
2-(6-methoxy-2-naphthyl)-propyl alcohol (2t) 79

2-(3-benzoylphenyl)-propanol (2u) 80
(4-chlorophenyl)(3-(2-hydroxyethyl)-5-methoxy-2-methyl-1H-indol-1-yl)methanone (2v)... 81
2-(6,11-dihydro-11-oxodibanzoxepin-2-yl) ethanol (2w)........ 82
8. References........ 83

1. Materials and Methods

Unless otherwise stated, all reactions were performed with standard Schlenk techniques. All reagents and starting materials were purchased at reagent grade and used as received. Anhydrous solvents were dried using an Innovative Technology PS-MD-5 solvent purification system. Thin layer chromatography (TLC) was performed on Merck Kieselgel 60 F254 aluminum plates with unmodified silica and visualized either under UV light or stained with potassium permanganate, vanillin, or cerium ammonium molybdate (Hanessian's stain). Column chromatography was performed with Merck silica gel 60 (35 – 70 mesh). Preparative TLC was performed using pre-coated TLC plates SIL G-50 UV250 (Layer: 0.50 mm silica gel 60 with fluorescent indicator UV_{250}); detection under UV light.

All ^{1}H, ^{13}C NMR and ^{11}B NMR spectra were recorded at ambient temperature on either Varian V-NMRS 600, Varian V-NMRS 400, Bruker AV-600 or Bruker AV-400 spectrometer. Chemical shifts (δ/ppm) were referenced to the residual solvent peak in ^{1}H (7.26 ppm for $CDCl_3$) (or 3.58 and 1.73 ppm for THF d_8) and ^{13}C spectra (77.16 ppm for $CDCl_3$). Coupling constants (J) are given in Hz. Signals are described as br = broad, s = singlet, d = doublet, dd = doublet of doublets, t = triplet, q = quartet, p = quintet, h = sextet and m = multiplet.

High-resolution mass spectrometry (HRMS) was performed using a Thermo Scientific LTQ Orbitrap XL spectrometer. Infrared (IR) spectra were recorded on a Perkin Elmer Spektrum 100 FT-IR spectrometer Spectrum 100 spectrometer with an UATR Diamond/KRS-5 crystal with attenuated total reflectance (ATR) and signals reported as wavenumbers in reciprocal centimeters. HPLC was performed using a Chiralpak IG (150 x 4,6) mm, 5µm, immobilized with (3-chloro-5-methylphenyl)carbamate.

2. Selected optimizations of reaction conditions

Optimization of the reaction condition was carried out with 4-benzoyl butyric acid **1a** on 0.1 mmol scale (Scheme 1).

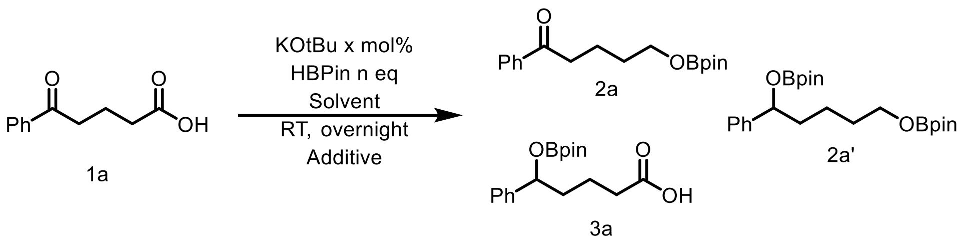

Scheme 1. 4-benzoyl butyric acid used for optimization of reaction conditions

Table 1. Optimization of reaction conditions

KOtBu (x mol%)	HBpin (n eq)	Solvent	2a [%]	2a' [%]	3a [%]	1a [%]	Additive
1 mol%	5	2-MeTHF 1M	60	9	-	4	-
3 mol%	5	2-MeTHF 1M	60	9	-	10	-
5 mol%	5	2-MeTHF 1M	56	9	-	15	-
1 mol%	2	2-MeTHF 1M	-	-	-	88	-
1 mol%	4	2-MeTHF 1M	44	-	-	28	-
**1 mol%**	**6**	**THF-d_8 1M**	**80**	**12**	**-**	**8**	**-**
1mol%	6	2-MeTHF 1M	-	-	-	60	TMEDA 1 eq
1 mol%	8	2-MeTHF 1M	85	12	-	-	-
1 mol%	10	2-MeTHF 1M	75	24	-	-	-
1 mol%	6	2-MeTHF 0.1M	-	-	-	85	-

Table 2. Optimization of reaction conditions- reaction time screening

KOtBu (x mol%)	HBpin (n eq)	Solvent	Reaction time	2a [%]	2a' [%]	3a [%]	1a [%]
1 mol%	4	2-MeTHF 1M	64 h	44	9	-	28
1 mol%	5	2-MeTHF 1M	64 h	67	5	-	6
1 mol%	6	2-MeTHF 1M	64 h	66	32	-	-

Table 3. Optimization of reaction conditions- solvents screening*

Solvent [1M]	2a [%]	2a' [%]	3a [%]	1a [%]
PhH	48	20	-	15
EtOAc	62	15	-	14
MeCN	56	11	-	15
Heptane	48	10	-	20

*All the reaction are carried out using 1 mol% of KOtBu and 6 eq of HBpin

Table 4. Replacement of KOtBu with different promoters

Promoter x mol%	HBpin eq	Solvent 1M	2a [%]	2a' [%]	3a [%]	1a [%]
$HCo[PPh(OEt)_2]_4$ 5 mol%	5	2-MeTHF	72	17	5	-
$NaBH_4$ 5 mol%	5	2-MeTHF	63	21	-	16
BNAH[1] 5 mol%	5	2-MeTHF	62	20	-	16
BH_3·DMS 5 mol%	5	2-MeTHF	75	-	-	-
BH_3·DMS 10 mol%	6	2-MeTHF	50	26	-	-
BH_3·DMS 20 mol%	6	2-MeTHF	45	18	-	-
K_2CO_3 6.4 mol%	6	THF-d_8	52	28	-	-
Cs_2CO_3 5.5 mol%	6	THF-d_8	64	19	-	-
NaHMDS 5 mol%	6	2-MeTHF	25	19	-	21
Ti(O*i*Pr)$_4$ 5 mol%	6	2-MeTHF	33	16	-	10
NaOTf 5 mol%	6	2-MeTHF	24	26	-	7
Fe(acac)$_3$ 5 mol%	6	2-MeTHF	21	28	-	7
Co(acac)$_2$ 5 mol%	6	2-MeTHF	33	22	-	10
No promoter	5	2-MeTHF	17	30	33	10

[1]BNAH- 1-Benzyl-1,4-dihydronicotinamide

The optimal reaction conditions that were chosen as follows: 6 equivalents of HBpin, 1 mol% KO^{t}Bu, 1 M solvent (2-MeTHF or THF) at room temperature, overnight (17 h).

2.1 Effect of EDG and EWG on the reaction outcome

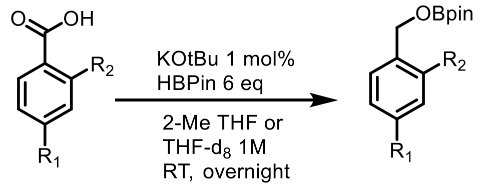

Scheme 2. Reduction of benzoic acid and its derivatives

Table 5. Effect of EDG and EWG in *p*- or *o*- position on the reaction outcome

Compound	R_1	R_2	Product [%][2]	RSM [%]
2d[1]	H	H	99	-
2e	Me	H	99	-
2f	Cl	H	99	-
2g[1]	OMe	H	99	-
2h	Ph	H	99	-
2i[1]	NO_2	H	-	100
2j	H	I	-	100
2k	H	OMe	25	44

[1] 7 eq of HBpin used

[2]NMR yield given. The yield was determined by using 1 eq of CH_2Br_2 as the internal standard

3. Synthesis of starting materials

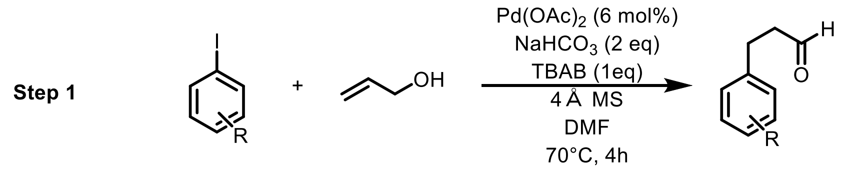

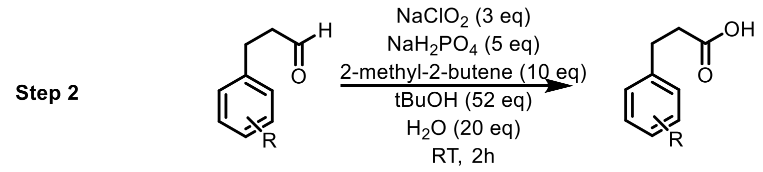

Compounds **1m**, **1n** and **1p** are synthesized following this protocol.

Step 1: Palladium (II) acetate (80.8 mg, 6 mol%, 0.36 mmol) was added to a suspension of the aryl-iodide substrate (1 eq, 6 mmol), allyl alcohol (0.60 ml, 1.5 eq, 9 mmol), $NaHCO_3$ (1.0 g, 2 eq, 12 mmol), tetrabutylammonium bromide (1.93 g, 1 eq, 6 mmol) and 4 Å molecular sieves (1.2 g) in 15 mL of dry DMF. The mixture was heated to 70 °C and allowed to stir for 4 h. The black suspension was then cooled to room temperature and filtered through a plug of celite with EtOAc (60 mL). The brown slurry was then poured over H_2O (80 mL) and extracted with EtOAc (3 × 50 mL). The combined organic layers were dried ($MgSO_4$) and concentrated under reduced pressure. The crude aldehyde was used in the next step without further purification.

Step 2: The crude aldehyde obtained in step 1 was dissolved in water (22 mL, 20 eq, 120 mmol) and tert-butyl alcohol (30 mL, 52 eq, 312 mmol). To this was added 2-methyl-2-butene (6.4 mL, 10 eq, 60 mmol), $NaClO_2$ (1.6 g, 3.0 eq, 18 mmol) and NaH_2PO_4 (3.6 g, 5.0 eq, 30 mmol). The mixture was left to stir for 2 hours at room temperature. The mixture was then diluted with EtOAc, and it was washed three times with water. The combined organic layers were washed with brine, dried ($MgSO_4$), filtered and concentrated under vacuum. The crude product was purified by column chromatography, as specified for each product.

Ethyl 3-(3-oxopropyl) benzoate (1m')

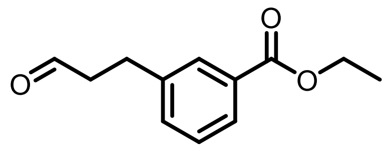

Prepared following step 1, **1m'** was obtained and the crude was used directly in the next step.

^{1}H NMR (400 MHz, Chloroform-*d*) δ 9.83 (s, 1H), 7.92 – 7.85 (m, 2H), 7.41 – 7.31 (m, 2H), 4.38 (q, *J* = 7.2 Hz, 2H), 3.02 (t, *J* = 7.5 Hz, 2H), 2.81 (t, *J* = 7.3 Hz, 2H), 1.39 (t, *J* = 7.2 Hz, 3H).

These data are in agreement with those reported previously in the literature.[1]

3-(3-(ethoxycarbonyl)phenyl) propanoic acid (1m)

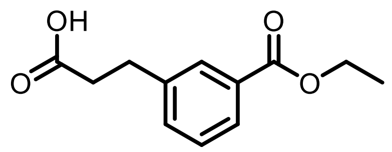

Prepared following step 2, **1m** was obtained after purification by column chromatography (DCM: AcOH 99:1) as bright yellow solid (403 mg, 1.82 mmol, 30 %).

¹H NMR (600 MHz, Chloroform-*d*) δ 7.92 – 7.87 (m, 2H), 7.42 – 7.40 (m, 2H), 7.37 (d, *J* = 7.8 Hz, 1H), 4.38 (d, *J* = 7.1 Hz, 2H), 3.02 (d, *J* = 7.8 Hz, 2H), 2.72 (d, *J* = 7.3 Hz, 2H), 1.40 (t, *J* = 7.1 Hz, 3H). **¹³C NMR** (151 MHz, Chloroform-*d*) δ 177.4, 166.7, 140.6, 133.0, 131.0, 129.5, 128.7, 127.9, 61.2, 35.3, 30.5, 14.5.

These data are in agreement with those reported previously in the literature.[2]

3-(4-nitrophenyl) propanal (1n')

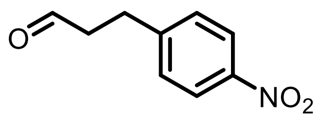

Prepared following step 1, **1n'** was obtained and the crude was used directly in the next step.

¹H NMR (600 MHz, Chloroform-*d*) δ 9.81 (t, *J* = 1.0 Hz, 1H), 8.17 – 8.08 (m, 2H), 7.38 – 7.30 (m, 2H), 3.07 – 3.00 (m, 2H), 2.84 – 2.81 (m, 2H).

These data are in agreement with those reported previously in the literature.[3]

3-(4-nitrophenyl) propionic acid (1n)

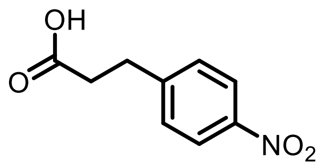

Prepared following step 2, **1n** was obtained after purification by column chromatography (DCM: AcOH= 99:1) as a brown solid (164 mg, 0.84 mmol, 14%).

¹H NMR (600 MHz, Chloroform-*d*) δ 8.19 – 8.14 (m, 2H), 7.41 – 7.36 (m, 2H), 3.07 (t, *J* = 7.5 Hz, 2H), 2.74 (t, *J* = 7.5 Hz, 2H). **¹³C NMR** (151 MHz, Chloroform-*d*) δ 176.4, 147.9, 147.0, 129.4, 124.0, 34.6, 30.4.

These data are in agreement with those reported previously in the literature.[2]

3-(p-cyanophenyl) propionaldehyde (1p')

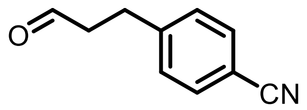

Prepared following step 1, **1p'** was obtained and the crude was used directly in the next step.

¹H NMR (600 MHz, Chloroform-*d*) δ 9.81 (t, *J* = 1.0 Hz, 1H), 7.59 – 7.54 (m, 2H), 7.33 – 7.28 (m, 2H), 3.00 (d, *J* = 7.4 Hz, 2H), 2.82 (d, *J* = 7.4 Hz, 2H).

These data are in agreement with those reported previously in the literature.[4]

3-(4-cyanophenyl)propionic acid (1p)

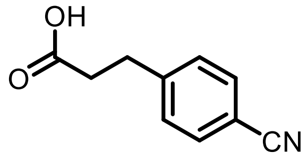

Prepared following step 2, **1p** was obtained after purification by column chromatography (DCM: AcOH= 99:1) as a yellow solid (127 mg, 0.72 mmol, 12%).

¹H NMR (600 MHz, Chloroform-*d*) 7.59 (d, *J* = 8.3 Hz, 2H), 7.33 (d, *J* = 8.3 Hz, 2H), 3.02 (t, *J* = 7.6 Hz, 2H), 2.71 (t, *J* = 7.6 Hz, 2H).
¹³C NMR (151 MHz, Chloroform-*d*) δ 176.3, 145.9, 132.5, 129.3, 119.0, 110.6, 34.6, 30.7. **IR (neat):** ν 2922, 2631, 2225, 1699, 1605, 1434, 1200, 933, 833 cm^{-1}.

These data are in agreement with those reported previously in the literature.[5]

Synthesis of 3-cyanopropanoic acid (1q)

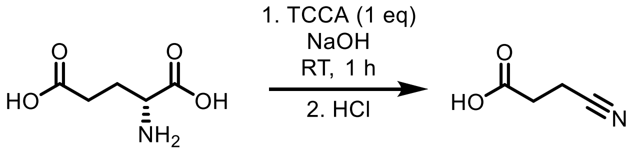

D- Glutamic acid (441.4 mg, 1 eq, 3 mmol) was dissolved in 1 M aqueous solution of NaOH (1.5 ml). It was treated with trichloroisocyanuric acid (464.8 mg, 0.67 eq, 2 mmol) at room temperature. It was left to stir for 1 h, and the reaction mixture was then treated with 1 M HCl (6 ml), followed by an aqueous solution of 3M HCl (0.1 ml). The crude reaction mixture was extracted with Et_2O (2x15 ml), organic layer was washed with water, dried ($MgSO_4$), and the solvent was removed under reduced pressure. Compound **1q** was obtained pure in 33% yield (99.9 mg, 1 mmol).

^{1}H NMR (600 MHz, $CDCl_3$) δ 9.82 (s, 1H), 2.78 (t, *J* = 7.2 Hz, 2H), 2.66 (t, *J* = 7.2 Hz, 2H).
^{13}C NMR (151 MHz, $CDCl_3$) δ 174.8, 118.2, 29.7, 12.9.
These data are in agreement with those reported previously in the literature.[6]

4. Reduction reactions products

4.1. General procedure 1

i. *For reactions carried out in THF-d_8*

In an oven-dried 4 ml vial were introduced starting material (0.1 mmol, 1 eq) and a stirring bar. A cap with rubber septum was used to close the vial and the system was then purged with argon. THF-d_8 (c = 1.0 M, 0.1 mL), KO^{t}Bu solution in THF-d_8 (c = 0.1 M, 1 mol%, 10 µL) and HBpin (6 eq, 0.6 mmol, 87 µL) were then added successively and the reaction was left to stir at room temperature overnight. After this time, an internal standard (1 eq, 0.1 mmol) and THF-d_8 (0.4 ml) were added to the crude reaction mixture to determine the yield by ^{1}H NMR. The resulting crude was then purified by column chromatography as detailed for the single compounds.

ii. *For reactions carried out in 2-MeTHF*

In an oven-dried 4 ml vial were introduced starting material (0.1 mmol, 1 eq) and a stirring bar. A cap with rubber septum was used to close the vial and the system was then purged with argon. 2-MeTHF (c = 1.0 M, 0.1 mL), KO^{t}Bu solution in 2-MeTHF (c = 0.1 M, 1 mol%, 10 µL) and HBpin (6 eq, 0.6 mmol, 87 µL) were then added successively and the reaction was left to stir at room temperature overnight. After this time, the solution was diluted with Et_2O and washed two times with distilled water. The combined aqueous layers were extracted two times with Et_2O and the combined organic layers were washed with brine, dried ($MgSO_4$), filtered and concentrated under vacuum. An internal standard (1 eq, 0.1 mmol) was added to the crude reaction mixture to determine the yield by ^{1}H NMR. The resulting crude was then purified by column chromatography as detailed for the single compounds.

Isolations: In some cases (noted below), separation of the products is difficult due to co-elution of both alcohol and boronic ester with pinacol which is large excess. In these cases, careful selection of eluent is required.

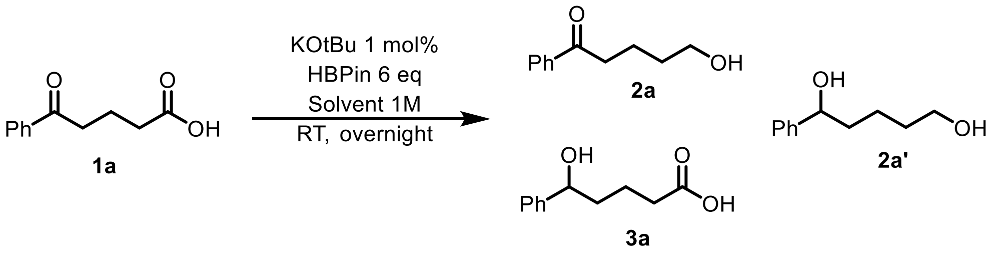

1-phenyl-5-((4,4,5,5-tetramethyl-1,3,2-dioxaborolan-2-yl)oxy)pentan-1-one (2a) and 2,2'-((1-phenylpentane-1,5diyl)bis(oxy))bis(4,4,5,5-tetramethyl-1,3,2-dioxaborolane) (2a')

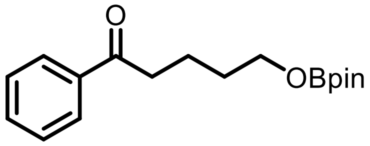

2a

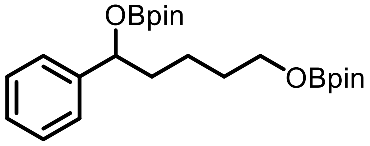

2a'

Prepared following general procedure 1, **2a** and **2a'** are obtained as a mixture. NMR yield was determined using $CHBr_3$ as the internal standard: **2a** was generated in 80% yield, **2a'** in 12% yield, and the starting material **1a** was recovered (8%).
Selected peaks for **1a**[7]: **^{1}H NMR** (600 MHz, THF-d_8) δ 3.05 (d, J = 7.2 Hz, 2H), 2.34 (d, J = 7.2 Hz, 2H).
Selected peaks for **2a**: **^{1}H NMR** (600 MHz, THF-d_8) δ 3.83 (d, J = 6.4 Hz, 2H), 3.00 (d, J = 7.3 Hz, 2H).
Selected peaks for **2a'**: **^{1}H NMR** (600 MHz, THF-d_8) δ 7.31 – 7.23 (m, 4H), 7.20 – 7.16 (m, 1H), 5.07 – 5.00 (m, 1H).

6-phenyl-1-hexanol (2b)

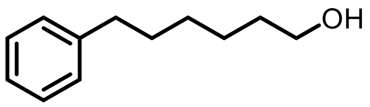

Prepared following general procedure 1, **2b** was obtained after purification by column chromatography (DCM) as a transparent oil (14.6 mg, 82 µmol, 82%).

^{1}H NMR (600 MHz, Chloroform-d) δ 7.31 – 7.25 (m, 2H), 7.20 – 7.15 (m, 3H), 3.64 (d, J = 6.6 Hz, 2H), 2.62 (d, J = 7.3 Hz, 2H), 1.67 – 1.61 (m, 2H), 1.60 – 1.55 (m, 2H), 1.44 (s, 1H), 1.41 – 1.36 (m, 4H). **^{13}C NMR** (151 MHz, Chloroform-d) δ 142.9, 128.5, 128.4, 125.8, 63.1, 36.0, 32.8, 31.6, 29.2, 25.7.

IR (neat): ν 3338, 2928, 2856, 1454, 1053, 743, 697 cm^{-1}. **HRMS (ESI):** m/z $[M+Na]^+$calculated for $[C_{12}H_{18}ONa]^+$: 201.1250, found: 201.1243.

2-methyl-3-phenylpropanol (2c)

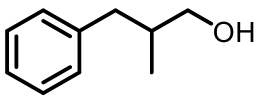

Prepared following general procedure 1, **2c** was obtained after purification by column chromatography (DCM) as a white solid (13.7 mg, 91 µmol, 91%).

^{1}H NMR (600 MHz, Chloroform-d) δ 7.30 – 7.24 (m, 2H), 7.21 – 7.14 (m, 3H), 3.53 (dd, J = 10.6, 5.8 Hz, 1H), 3.47 (dd, J = 10.6, 6.0 Hz, 1H), 2.75 (dd, J = 13.5, 6.3 Hz, 1H), 2.42 (dd, J = 13.5, 8.0 Hz, 1H), 1.99 – 1.89 (m, 1H), 0.91 (d, J = 6.7 Hz, 3H). **^{13}C NMR** (151 MHz, Chloroform-d) δ 140.8, 129.3, 128.4, 126.0, 67.8, 39.9, 37.9, 16.6.

IR (neat): ν 3398, 2922, 2856, 1725, 1454, 1130, 1032, 739, 698 cm^{-1}. **HRMS (ESI):** m/z $[M+Na]^+$calculated for $[C_{10}H_{14}ONa]^+$: 173.0937, found: 173.0938.

Benzyl alcohol (2d)

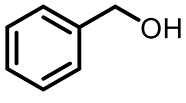

Prepared following general procedure 1 (on 0.2 mmol scale, with 7 eq of HBpin, after doing the work-up with 1M aqueous solution of NaOH), **2d** was obtained as a colorless oil after purification by column chromatography in 46% yield (10 mg, 92.5 µmol). The isolated yield is significantly lower as a result of difficulties in separation from pinacol.

^{1}**H NMR** (600 MHz, $CDCl_3$) δ 7.39 – 7.35 (m, 4H), 7.32 – 7.29 (m, 1H), 4.70 (s, 2H). ^{13}**C NMR** (151 MHz, $CDCl_3$) δ 141.0, 128.7, 127.8, 127.1, 65.6.
These data are in agreement with those reported previously in the literature. [8]

4-methylbenzyl alcohol (2e)

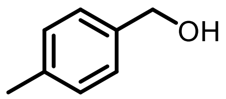

Prepared following general procedure 1, **2e** was obtained as a white solid after purification by column chromatography (DCM) in 80% yield (9.8 mg, 80.2 µmol, 80%).

^{1}**H NMR** (600 MHz, $CDCl_3$) δ 7.27 – 7.25 (m, 2H), 7.17 (d, *J* = 7.8 Hz, 2H), 4.65 (s, 2H), 2.35 (s, 3H). ^{13}**C NMR** (151 MHz, $CDCl_3$) δ 138.1, 137.6, 129.4, 127.3, 65.5, 21.3.
These data are in accordance with those previously reported. [8]

4-chlorobenzyl alcohol (2f)

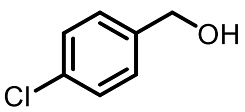

Prepared following general procedure 1, **2f** was obtained after purification by column chromatography in 58% yield (8.3 mg, 58 µmol).

^{1}**H NMR** (600 MHz, $CDCl_3$) δ 7.37 – 7.28 (m, 4H), 4.67 (s, 2H). ^{13}**C NMR** (151 MHz, $CDCl_3$) δ 139.4, 133.5, 128.8, 128.4, 64.7.
These data are in agreement with those reported previously in the literature.[9]

4-methoxybenzyl alcohol (2g)

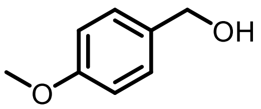

Prepared following general procedure 1 (with 7 eq of HBpin), **2g** was obtained as a transparent oil after purification by column chromatography (DCM) in 81% yield (11.2 mg, 81 µmol).

^{1}**H NMR** (600 MHz, $CDCl_3$) δ 7.29 (d, *J* = 8.2 Hz, 2H), 6.90 (d, *J* = 8.1 Hz, 2H), 4.62 (s, 2H), 3.81 (s, 3H). ^{13}**C NMR** (151 MHz, $CDCl_3$) δ 159.4, 133.3, 128.8, 114.1, 65.2, 55.4.
These data are in agreement with those previously reported in the literature. [8]

Biphenyl-4-yl-methanol (2h)

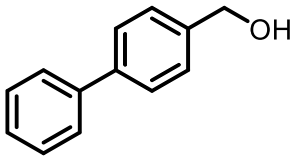

Prepared following general procedure 1, **2h** was obtained as a white solid after purification by column chromatography (DCM) in 78% yield (14.3 mg, 78 µmol).

^{1}**H NMR** (600 MHz, $CDCl_3$) δ 7.63 – 7.56 (m, 4H), 7.48 – 7.41 (m, 4H), 7.37 – 7.34 (m, 1H), 4.75 (s, 2H). ^{13}**C NMR** (151 MHz, $CDCl_3$) δ 141.0, 140.8, 140.0, 128.9, 127.6, 127.5, 127.5, 127.2, 65.3.
These data are in agreement with those reported previously in the literature.[10]

2-((2-methoxybenzyl)oxy)-4,4,5,5-tetramethyl-1,3,2-dioxaborolane (2k)

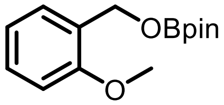

Prepared following general procedure 1, **2k** was obtained crude in 25% yield, with 44% of the starting material recovered. The NMR yield was determined by using CH_2Br_2 as the internal standard.

Characterization data for the remaining of the starting material **1k**: **^{1}H NMR** (600 MHz, THF-d_8) δ 10.88 (s, 1H), 7.80 (dd, *J* = 7.8, 1.8 Hz, 1H), 7.53 – 7.41 (m, 1H), 7.06 (d, *J* = 8.4 Hz, 1H), 6.98 – 6.93 (m, 1H), 3.87 (s, 3H).

Characterization data for the product **2k** (selected peaks): **^{1}H NMR** (600 MHz, THF-d_8) δ 7.34 – 7.29 (m, 1H), 7.20 – 7.14 (m, 1H), 6.90 – 6.83 (m, 2H), 3.79 (s, 3H).

These data are in close accordance with those previously reported in the literature.[11,12]

(3E)-4-phenyl-3-buten-1-ol (2l)

Prepared following general procedure 1, **2l** was obtained after purification by column chromatography (DCM) as a green oil (8.8 mg, 59.3 µmol, 60%).

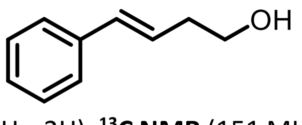

^{1}H NMR (600 MHz, Chloroform-*d*) δ 7.36 (d, *J* = 7.2 Hz, 2H), 7.30 (*app* t, *J* = 7.7 Hz, 2H), 7.22 (t, *J* = 7.4 Hz, 1H), 6.51 (d, *J* = 15.9 Hz, 1H), 6.21 (dd, *J* = 15.9, 7.2 Hz, 1H), 3.77 (t, *J* = 6.3 Hz, 2H), 2.50 (td, *J* = 6.3, 15.9 Hz, 2H). **^{13}C NMR** (151 MHz, Chloroform-*d*) δ 137.4, 133.0, 128.7, 127.4, 126.5, 126.2, 62.2, 36.6.

IR (neat): ν 3352, 2960, 1258, 1015, 793 cm^{-1}. **HRMS (ESI):** m/z [M+Na]$^+$ calculated for [$C_{10}H_{12}ONa$]$^+$: 171.0780, found: 171.0783.

Ethyl 3-(3-hydroxypropyl) benzoate (2m)

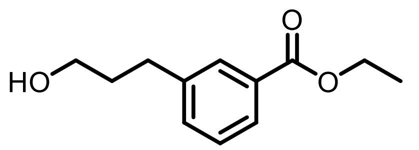

Prepared following general procedure 1, **2m** was obtained after purification by column chromatography (DCM: acetone 98:2), as a bright yellow solid (14.6 mg, 70 µmol, 70%).

^{1}H NMR (600 MHz, Chloroform-*d*) δ 7.92 – 7.84 (m, 2H), 7.41 – 7.38 (m, 1H), 7.37 – 7.33 (m, 1H), 4.37 (q, *J* = 7.1 Hz, 2H), 3.68 (d, *J* = 6.4 Hz, 2H), 2.77 (t, *J* = 7.9 Hz, 2H), 1.96 – 1.88 (m, 2H), 1.40 (t, *J* = 7.1 Hz, 3H).**^{13}C NMR** (151 MHz, Chloroform-*d*) δ 166.9, 142.2, 133.1, 130.8, 129.6, 128.5, 127.3, 62.2, 61.1, 34.2, 32.0, 14.5.

IR (neat): ν 3422, 2933, 1715, 1277, 1192, 1050, 749 cm^{-1}. **HRMS (ESI):** m/z [M+Na]$^+$calculated for [$C_{12}H_{16}O_3Na$]$^+$: 231.0992, found: 231.0989.

3-(4-nitrophenyl)-1-propanol (2n)

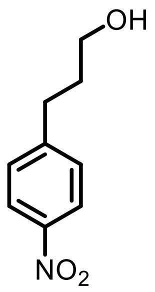

Prepared following general procedure 1, **2n** was obtained after purification by column chromatography (DCM: acetone 99:1) as a yellow oil (12.6 mg, 69 µmol, 69%).

^{1}H NMR (600 MHz, Chloroform-*d*) δ 8.14 (d, *J* =8.5 Hz, 2H), 7.36 (d, *J* = 8.5 Hz, 2H), 3.69 (d, *J* = 6.3 Hz, 2H), 2.84 (d, *J* = 7.9 Hz, 2H), 1.97 – 1.86 (m, 2H). **^{13}C NMR** (151 MHz, Chloroform-*d*) δ 150.0, 146.6, 129.4, 123.8, 61.9, 33.7, 32.1.

IR (neat): ν 3359, 2937, 1599, 1514, 1341, 1041, 795 cm^{-1}. **HRMS (ESI):** m/z [M+Na]$^+$calculated for [$C_9H_{11}O_3NNa$]$^+$: 204.0631, found: 204.0629.

1-bromo-6-hexanol (2o)

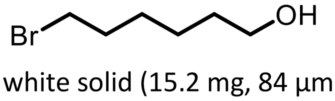

Prepared following general procedure 1, **2o** was obtained after purification by column chromatography (DCM: acetone 97:3) as a white solid (15.2 mg, 84 µmol, 84%).

^{1}H NMR (600 MHz, Chloroform-*d*) δ 3.65 (d, *J* = 6.6 Hz, 2H), 3.41 (d, J = 6.8 Hz, 2H), 1.92 – 1.85 (m, 2H), 1.62 – 1.56 (m, 2H), 1.50 – 1.45 (m, 2H), 1.43 – 1.38 (m, 2H).**^{13}C NMR** (151 MHz, Chloroform-d) δ 62.9, 33.9, 32.9, 32.7, 28.1, 25.1.

These data are in agreement with those reported previously in the literature.[13]

4-(3-Hydroxypropyl) benzonitrile (2p)

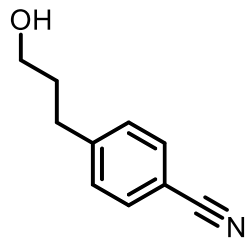

Prepared following general procedure 1, **2p** was obtained after purification by column chromatography (DCM: acetone 99:1) as a brown oil (15.6 mg, 97 µmol, 97%).

^{1}H NMR (600 MHz, Chloroform-*d*) δ 7.60 – 7.54 (m, 2H), 7.32 – 7.29 (m, 2H), 3.67 (d, *J* = 6.3 Hz, 2H), 2.82 – 2.73 (m, 2H), 1.93 – 1.85 (m, 2H). **^{13}C NMR** (151 MHz, Chloroform-*d*) δ 147.7, 132.4, 129.4, 119.2, 109.9, 61.9, 33.7, 32.4.

These data are in agreement with those reported previously in the literature.[14]

4-((4,4,5,5-tetramethyl-1,3,2-dioxaborolan-2-yl)oxy)butanenitrile (2q)

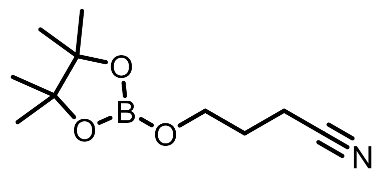

Prepared following general procedure 1 (on 0.186 mmol scale), **2q** was isolated as mixture with pinacol. The mixture is a yellow oil (147.9 mg, of which **2q** comprises 26.6 mg, 126 µmol, 68%, while pinacol comprises 121.3 mg, 1.03 mmol)

^{1}H NMR (600 MHz, $CDCl_3$) (selected peaks): δ 3.91 (t, *J* = 5.8 Hz, 2H), 2.43 (d, *J* = 7.2 Hz, 2H), 1.91 – 1.82 (m, 2H). **^{13}C NMR** (151 MHz, $CDCl_3$) δ 119.3, 83.2, 83.1, 62.6, 27.4, 24.1, 13.8.

IR (neat): ν 3405, 2980, 1478, 1415, 1316, 1271, 1136, 1023, 956, 671 cm^{-1}. **HRMS (EI):** m/z [M-Me]$^+$ calculated for [$C_9H_{15}O_3NB$]: 196.1140, found: 196.1142.

N-(3-hydroxypropyl) benzamide (2r)

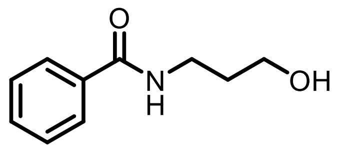

Prepared following general procedure 1, **2r** was obtained after purification by column chromatography (DCM: acetone 95:5 and preparative TLC DCM: acetone 9:1) as a white solid (9.7 mg, 54 µmol, 54%).

^{1}H NMR (600 MHz, Chloroform-*d*) δ 7.80 – 7.74 (m, 2H), 7.53 – 7.48 (m, 1H), 7.43 (d, *J* = 7.6 Hz, 2H), 6.76 (s, 1H), 3.73 (d, *J* = 5.5 Hz, 2H), 3.64 (d, *J* = 6.1 Hz, 2H), 2.94 (s, 1H), 1.80 (d, *J* = 5.7 Hz, 2H). **^{13}C NMR** (151 MHz, Chloroform-*d*) δ 168.7, 134.2, 131.8, 128.8, 127.1, 60.0, 37.4, 32.3.

IR (neat): ν 3306, 2935, 1634, 1540, 1305, 1064, 699 cm^{-1}. **HRMS (ESI):** m/z [M+Na]$^+$ calculated for [$C_{10}H_{13}O_2NNa$]$^+$: 202.0838, found: 202.0838.

Benzyl(7-hydroxyheptyl)carbamate (2s) and benzyl(7-((4,4,5,5(--tetramethyl-1,2,3-dioxaborolan-2-yl)oxy)heptyl) carbamate (2s')

2s

2s'

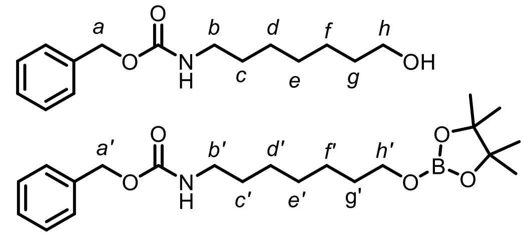

Prepared following general procedure 1 (on 0.2 mmol scale), **2s** and **2s'** are obtained as a mixture in total yield 64% (38.5 mg, out of which **2s** comprises 25.8 mg, 97 µmol, 48%, and **2s'** is 12.7 mg, 32 µmol, 16%). The products are obtained as a yellow oil after purification by preparative TLC (DCM: acetone=96:4).

^{1}H NMR (600 MHz, $CDCl_3$) δ 7.37 – 7.33 (m, 4H), 7.32 – 7.29 (m, 1H), 5.11 (s, 2H, a'), 5.09 (s, 2H, *a*), 4.76 (s, 1H, N**H**), 3.82 (d, *J* = 6.5 Hz, 2H, *h'*), 3.62 (t, *J* = 6.6 Hz, 2H, *h*), 3.18 (t, *J* = 7.1 Hz, 3H, *b* + *b'*), 1.58 – 1.45 (m, 6H, *c, c' + g, g'*), 1.36 – 1.28 (m, 8H, *d, d', e, e', f, f'),* 1.24 (s, 12H, $CH_2OB(OC(C\mathbf{H_3})_2)_2$).

^{13}C NMR (151 MHz, $CDCl_3$) δ 156.6, 136.8, 128.6, 128.5, 128.2 (2C), 128.0 127.9, 82.8 ($CH_2OB(O\mathbf{C}(CH_3)_2)_2$ 2n'), 66.9 (*a'*) 66.7 (*a*), 65.0 (*h'*), 63.0 (*h*), 41.2 (*b*, *b'*), 32.7 (*g*), 31.4 (*g'*), 30.0 (*c*), 29.8 (*c'*), 29.1 (*e*), 29.0 (*e'*), 26.8 (*f*), 26.7 (*f'*), 25.7 (*d*), 25.6 (*d'*), 24.7 ($CH_2OB(OC(\mathbf{C}H_3)_2)_2$, 2n'). **IR (neat):** ν 3338, 2932, 2858, 1695, 1527, 1451, 1372, 1327, 1248, 1114, 1042, 981 cm^{-1}.

HRMS (ESI): m/z $[M+Na]^+$calculated for $[C_{15}H_{23}O_3NNa]^+$: 288.1570, found: 288.1563.

2-(6-methoxy-2-naphthyl)-propyl alcohol (2t)

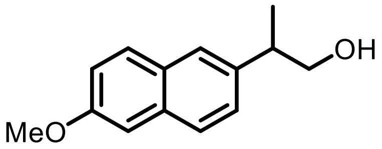

Prepared following general procedure 1, **2t** was obtained after purification by column chromatography (DCM) as a white solid (21.5 mg, 99 µmol, 99%).

^{1}H NMR (600 MHz, Chloroform-*d*) δ 7.71 (m, 2H), 7.61 (d, *J* = 1.8 Hz, 1H), 7.35 (dd, *J* = 8.4, 1.8 Hz, 1H), 7.17 – 7.11 (m, 2H), 3.92 (s, 3H), 3.78 (d, *J* = 6.8 Hz, 2H), 3.13 – 3.04 (m, 1H), 1.36 (d, *J* = 7.0 Hz, 3H). **^{13}C NMR** (151 MHz, Chloroform-*d*) δ 157.6, 138.8, 133.7, 129.2, 129.2, 127.3, 126.4, 126.0, 119.0, 105.8, 68.8, 55.4, 42.5, 17.8.

IR (neat): ν 3299, 2960, 2928, 2877, 1602, 1457, 1389, 1211, 1026, 851 cm^{-1}. **HRMS (ESI):** m/z $[M+Na]^+$ calculated for $[C_{14}H_{17}O_2]$: 217.1223, found: 217.1219.

2-(3-benzoylphenyl)-propanol (2u)

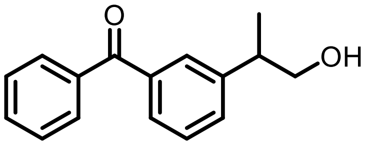

Prepared following general procedure 1 using 5 mol% of KO^{t}Bu (50 µl of a 0.1 M solution, 5 µmol), **2u** was obtained after purification by column chromatography (DCM: acetone 98:2, then preparative TLC DCM: acetone 98:2) as a white solid (7.5 mg, 31 µmol, 31%).

^{1}H NMR (600 MHz, Chloroform-*d*) δ 7.82 – 7.79 (m, 2H), 7.73 – 7.70 (m, 1H), 7.64 (dd, *J* = 7.5, 1.5 Hz, 1H), 7.61 – 7.57 (m, 1H), 7.51 – 7.46 (m, 3H), 7.43 (d, *J* = 7.6 Hz, 1H), 3.75 (dd, *J* = 6.8, 1.6 Hz, 2H), 3.09 – 3.00 (m, 1H), 1.32 (d, *J* = 7.0 Hz, 3H). **^{13}C NMR** (151 MHz, Chloroform-*d*) δ 196.9, 144.4, 138.0, 137.8, 132.6, 131.8, 130.2, 129.1, 128.8, 128.6, 128.4, 68.6, 42.5, 17.7.

IR (neat): ν 3429, 2961, 2927, 2874, 1653, 1595, 1446, 1281, 1029, 701 cm^{-1}. **HRMS (ESI):** m/z [M+Na]$^+$calculated for [$C_{16}H_{16}O_2Na$]$^+$: 263.1042, found: 263.1042.

(4-chlorophenyl)(3-(2-hydroxyethyl)-5-methoxy-2-methyl-1H-indol-1-yl)methanone (2v)

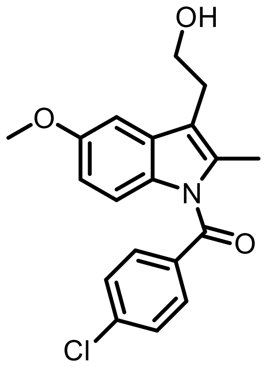

Prepared following general procedure 1, **2v** was obtained after purification by column chromatography (DCM: acetone 99:1) as a green solid (26.4 mg, 77 µmol, 77%).

^{1}H NMR (600 MHz, Chloroform-*d*) δ 7.67 – 7.62 (m, 2H), 7.49 – 7.45 (m, 2H), 6.95 (d, *J* = 2.5 Hz, 1H), 6.87 (d, *J* = 9.0 Hz, 1H), 6.67 (dd, *J* = 9.0, 2.5 Hz, 1H), 3.87 (d, *J* = 6.7 Hz, 2H), 3.84 (s, 3H), 2.95 (d, *J* = 6.7 Hz, 2H), 2.37 (s, 3H). **^{13}C NMR** (151 MHz, Chloroform-*d*) δ 168.4, 156.1, 139.3, 135.6, 134.2, 131.3, 131.2, 129.3, 116.0, 115.2, 111.5, 101.4, 62.3, 55.9, 27.8, 13.5.

IR (neat): ν 3438, 2932, 1675, 1595, 1473, 1360, 1318, 1221, 1041, 836, 731 cm^{-1}. **HRMS (ESI):** m/z [M+Na]$^+$calculated for [$C_{19}H_{18}O_3NClNa$]$^+$: 366.0867, found: 366.0859.

2-(6,11-dihydro-11-oxodibanzoxepin-2-yl) ethanol (2w)

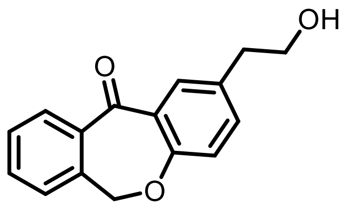

Prepared following general procedure 1, **2w** was obtained after purification by column chromatography (DCM: acetone 99:1, then preparative TLC DCM: Acetone 95:5) as a white solid (11.5 mg, 45.2 µmol, 45%).

^{1}H NMR (600 MHz, Chloroform-*d*) δ 8.09 (d, *J* = 2.3 Hz, 1H), 7.90 (dd, *J* = 7.7, 1.4 Hz, 1H), 7.56 (dd, *J* = 7.5, 1.4 Hz, 1H), 7.48 (dd, *J* = 7.5, 1.3 Hz, 1H), 7.41 – 7.35 (m, 2H), 7.02 (d, *J* = 8.3 Hz, 1H), 5.19 (s, 2H), 3.89 (d, *J* = 6.5 Hz, 2H), 2.90 (d, *J* = 6.5 Hz, 2H). **^{13}C NMR** (151 MHz, Chloroform-*d*) δ 191.2, 160.2, 140.7, 136.4, 135.8, 132.9, 132.4, 131.9, 129.6, 129.4, 127.9, 125.4, 121.1, 73.8, 63.6, 38.3.

IR (neat): ν 3433, 2925, 1716, 1644, 1604, 1487, 1298, 1017, 759 cm^{-1}. **HRMS (ESI):** m/z [M+Na]$^+$calculated for [$C_{16}H_{14}O_3Na$]$^+$: 277.0835, found: 277.0835.

4.2. Reaction scale-up

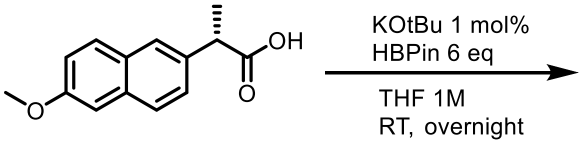

Naproxen (1.0 g, 1 eq, 4.35 mmol) was weighed into a round bottom flask and this was evacuated and back-filled with Argon. Dry THF (4.35 ml, 1 M) was added and then KO*t*Bu as a 1 M solution in THF (43.5 µl, 1 mol%, 43.5 µmol) and HBpin (3.78 ml, 6 eq, 26.1 mmol). The reaction mixture was left to stir overnight at room temperature. The reaction was quenched with water and extracted with EtOAc (3 x 20 ml). The combined organic phases were dried over $MgSO_4$, and the solvent was removed *in vacuo*. The crude product was purified by column chromatography (DCM) to obtain a while solid in 57% yield (535.4 mg, 2.47 mmol). The NMR data are in accordance with those reported for compound **2t**, and they match those reported previously in the literature.[15]

4.3. Enantioselectivity of the reaction

Both racemic and (*S*)-Naproxen (**1t**) were reduced to the corresponding alcohols (**2t-racemic** and **2t-(*S*)**, respectively) following general procedure 1, and *ee* was calculated to be 99%.

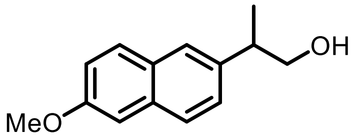

2t-racemic

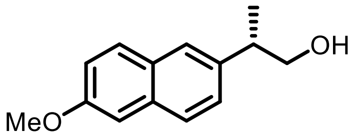

2t-(*S*)

Picture 1 shows HPLC spectrum of the product obtained by reducing racemic naproxen (**2t-racemic**), while **Picture 2** shows HPLC spectrum of the product obtained by reducing chiral naproxen (**2t-(*S*)**). **Picture 3** shows the overlap of the two spectra.

Column: Chiralpak IG, (150 x 4,6) mm, 5µ, SN: IG00CD-UE005

Pressure at start: 18 bar **Start flow:** 0.700 ml/min **Column oven:** 29.99 °C

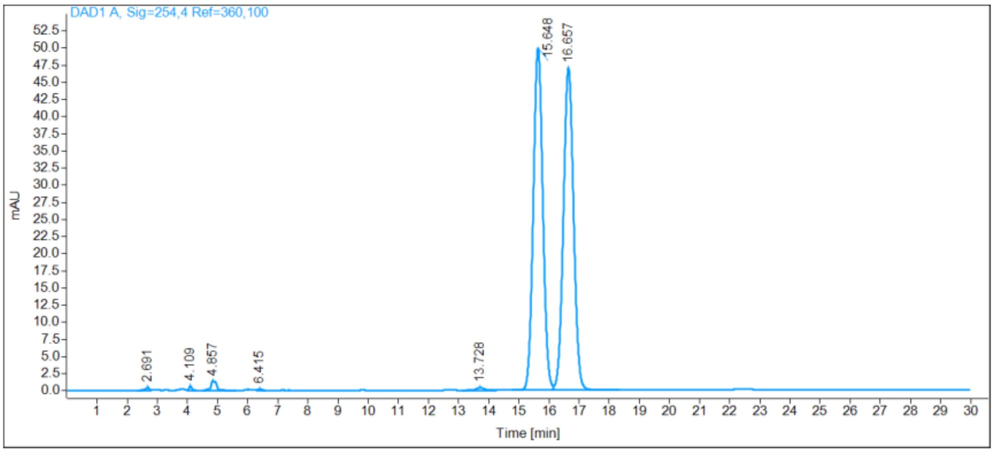

Name DL 944 rac

RT [min]	Type	Area%	Area	Height	Width [min]
2.69	BV	0.17	3.73	0.46	0.12
4.11	VB	0.17	3.71	0.62	0.09
4.86	BB	0.76	16.94	1.38	0.17
6.41	VB	0.08	1.82	0.20	0.14
13.73	BB	0.31	6.96	0.38	0.28
15.65	BV	49.07	1093.17	49.92	0.34
16.66	VB	49.44	1101.43	47.02	0.37
	Sum	100.00	2227.77		

Picture 1. HPLC spectrum of **2t-racemic**

Column: Chiralpak IG, (150 x 4,6) mm, 5µ, SN: IG00CD-UE005

Pressure at start: 18 bar **Start flow:** 0.700 ml/min **Column oven:** 30 °C

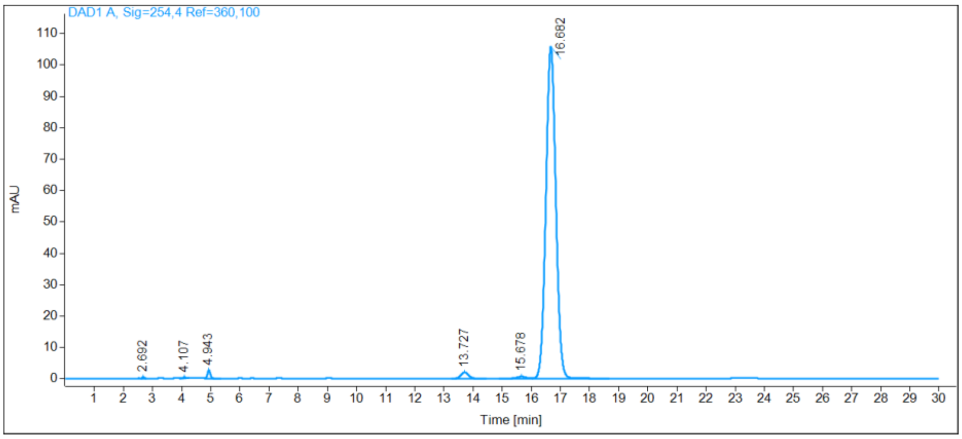

Name DL 936

RT [min]	Type	Area%	Area	Height	Width [min]
2.69	VB	0.08	2.14	0.42	0.08
4.11	VB	0.10	2.56	0.34	0.11
4.94	VB	0.68	17.42	2.62	0.10
13.73	BB	1.43	36.57	2.06	0.28
15.68	BV	0.54	13.71	0.59	0.35
16.68	VB	97.17	2485.34	105.62	0.37
	Sum	100.00	2557.74		

Picture 2. HPLC spectrum of **2t-(*S*)**

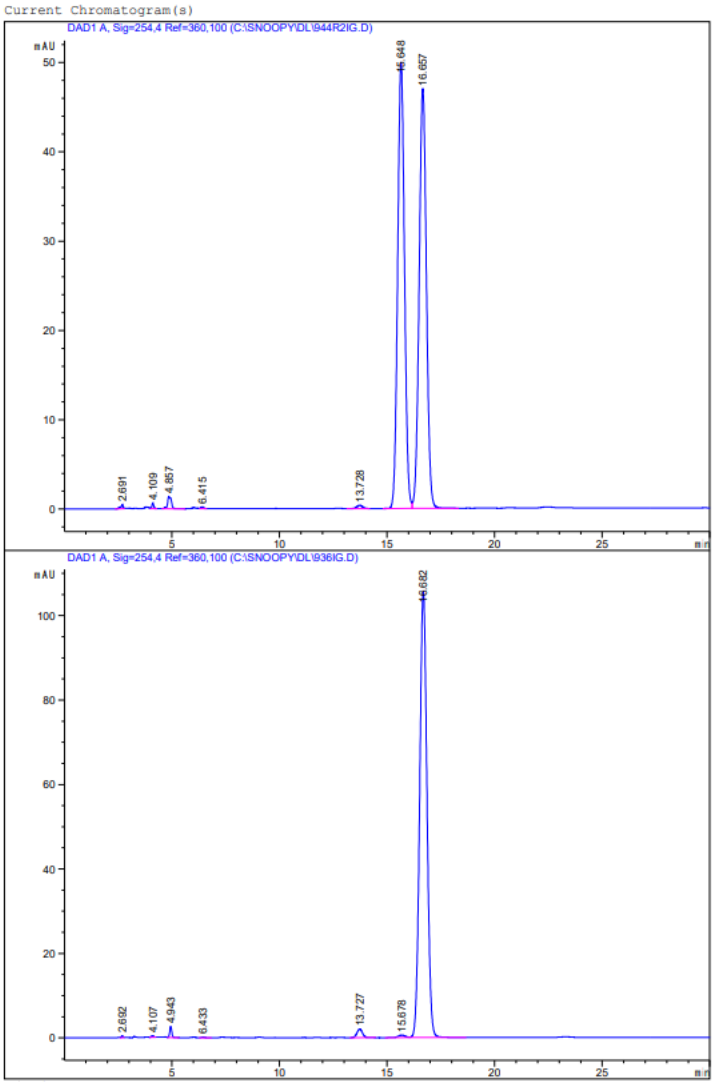

Picture 3. Overlap of HPLC spectra for **2t-racemic** (upper spectrum) and **2t-(*S*)** (bottom spectrum)

4.4. General procedure 2 – Reduction of selected carboxylic acids with stoichiometric BH_3·DMS

Conditions A: In an oven-dried 4 ml vial were introduced starting material (0.1 mmol, 1 eq) and a stirring bar. A cap with rubber septum was used to close the vial and the system was then purged with argon. Then, THF (c = 0.5 M, 0.2 mL) was added followed by the addition of BH_3·DMS (0.65 ml of prepared 0.2 M solution of BH_3·DMS in THF, 1.3 eq, 0.13 mmol) at 0°C. It was left to stir at room temperature during 6 h. The solution was diluted with Et_2O and washed two times with distilled water, the combined aqueous layers were extracted two times with Et_2O and the combined organic layers were washed with brine, dried ($MgSO_4$), filtered and concentrated under vacuum. Internal standard (1 eq, 0.1 mmol) was then added to the crude reaction mixture to determine ^1H NMR yield.

Conditions B: In an oven-dried 4 ml vial were introduced starting material (0.1 mmol, 1 eq) and a stirring bar. A cap with rubber septum was used to close the vial and the system was then purged with argon. Then, THF-d_8 (c = 0.5 M, 0.2 mL) was added followed by the addition of BH_3·DMS (1.25 ml of prepared 0.2 M solution of BH_3·DMS in THF, 2.5 eq, 0.25 mmol) at 0°C. It

was left to stir at room temperature during 6 h. After this time, internal standard $CHBr_3$ (1 eq, 0.1 mmol) was added to the crude reaction mixture to determine ^1H NMR yield.

3-(4-nitrophenyl)-1-propanol (2n-a and 2n-b)

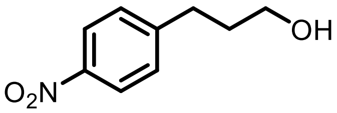

Prepared following general procedure 2 (conditions A for **2n-a**, conditions B for **2n-b**), product was obtained crude in 55% yield (**2n-a**) and 62% yield (**2n-b**). NMR yield was determined using $CHBr_3$ as the internal standard.

Characterization data for **2n-a:** ^1H NMR (600 MHz, Chloroform-*d*) δ 8.19 – 8.10 (m, 2H), 7.39 – 7.32 (m, 2H), 3.69 (d, *J* = 6.2 Hz, 2H), 2.83 (d, *J* = 7.8 Hz, 2H), 1.95 – 1.87 (m, 2H).

These data are in agreement with those previously.[17]

Spectrum of crude reaction mixture for 3-(4-nitrophenyl)-1-propanol (2n-a)

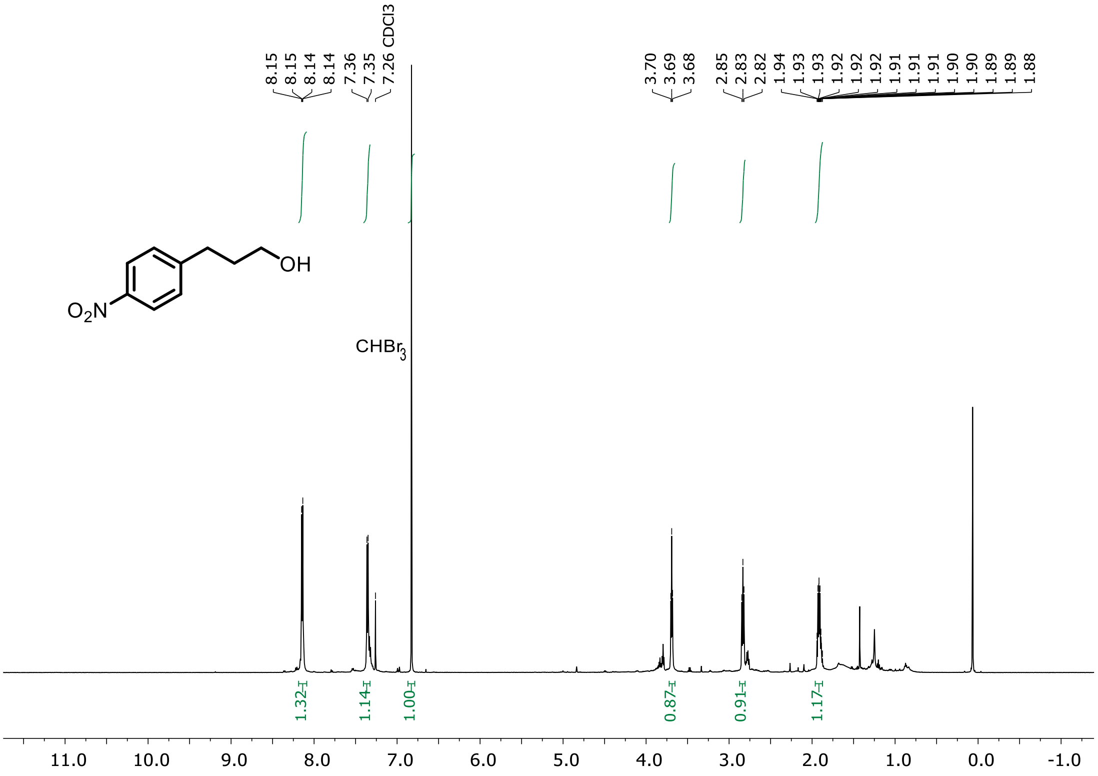

Spectrum of crude reaction for 3-(4-nitrophenyl)-1-propanol (2n-b)

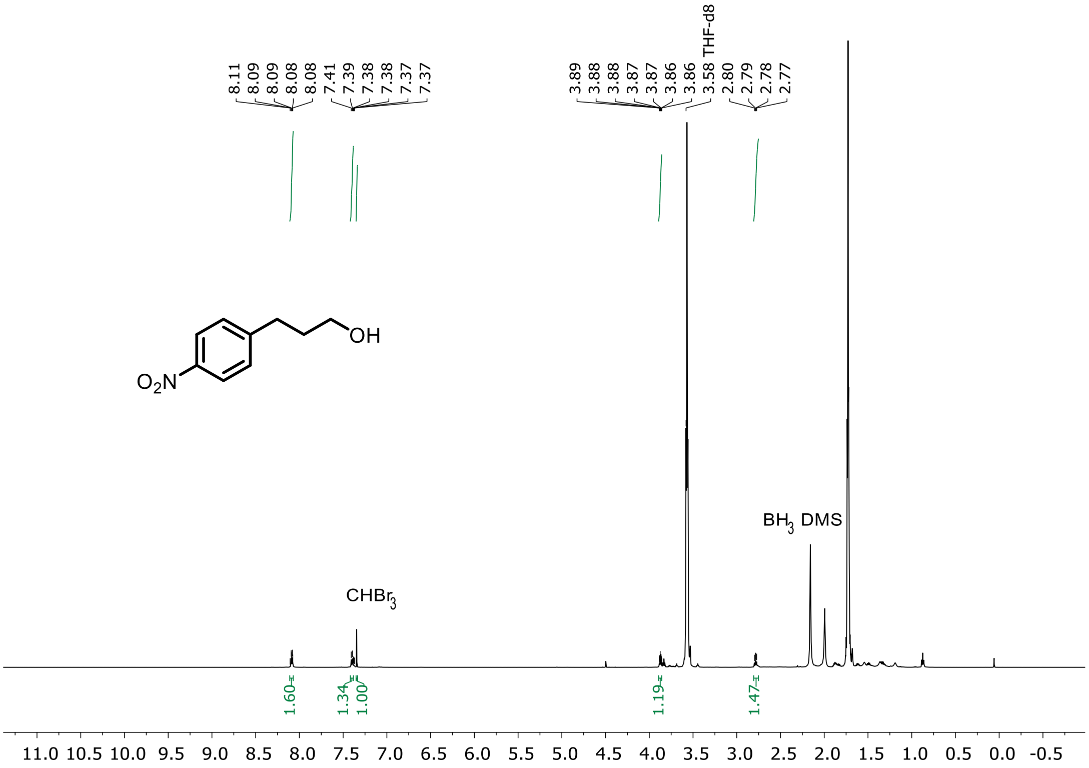

4-(3-Hydroxypropyl) benzonitrile (2p-a and 2p-b)

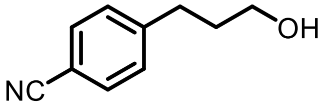

Prepared following general procedure 2 (conditions A for **2p-a,** conditions B for **2p-b**), product was obtained crude in 53% yield (**2p-a**) and 40% yield (**2p-b**). NMR yield was determined using $CHBr_3$ as the internal standard.

Characterization data for **2p-a:** ^{1}**H NMR** (600 MHz, Chloroform-*d*) δ 7.57 (d, *J* = 8.2 Hz, 2H), 7.30 (d, *J* = 8.1 Hz, 2H), 3.67 (d, *J* = 6.3 Hz, 2H), 2.80 – 2.75 (m, 2H), 1.94 – 1.86 (m, 2H).
Characterization data for **2p-b**: ^{1}**H NMR** (600 MHz, THF-d_8) δ 7.60 – 7.56 (m, 2H), 7.35 – 7.32 (m, 2H), 3.87 – 3.83 (m, 2H), 2.76 – 2.72 (m, 2H).

These data are in agreement with those reported previously in the literature.[14]

Spectrum of crude reaction for 4-(3-Hydroxypropyl) benzonitrile (2p-a)

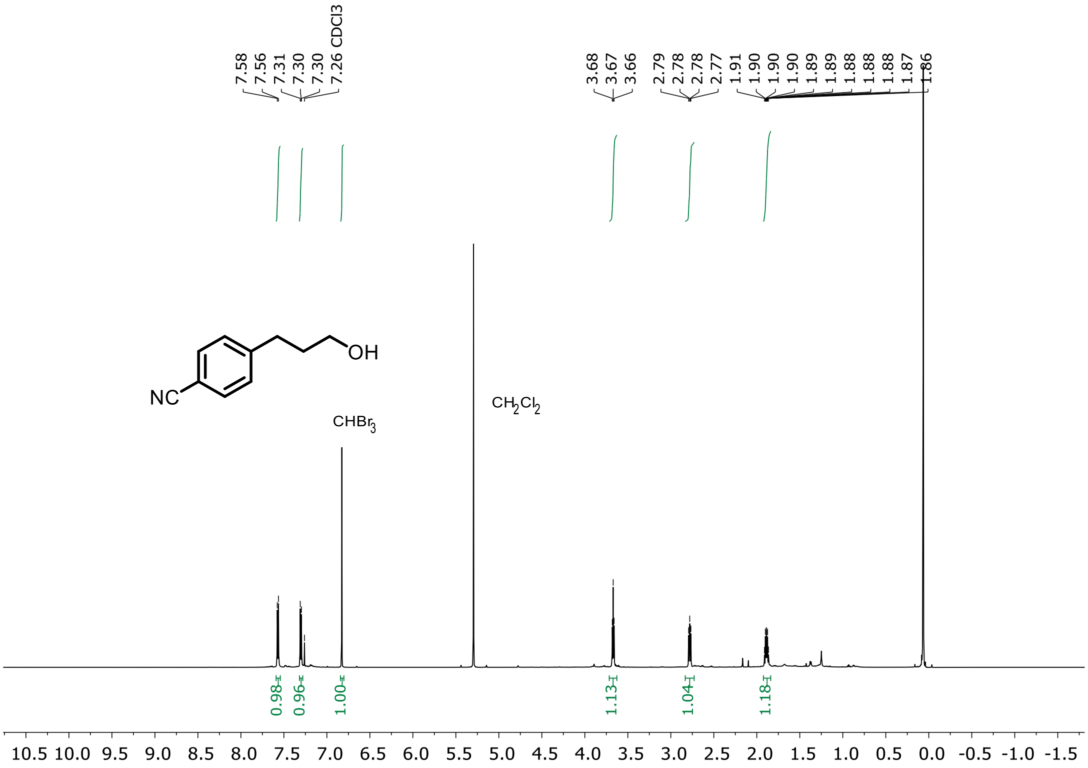

Spectrum of crude reaction for 4-(3-Hydroxypropyl) benzonitrile (2p-b)

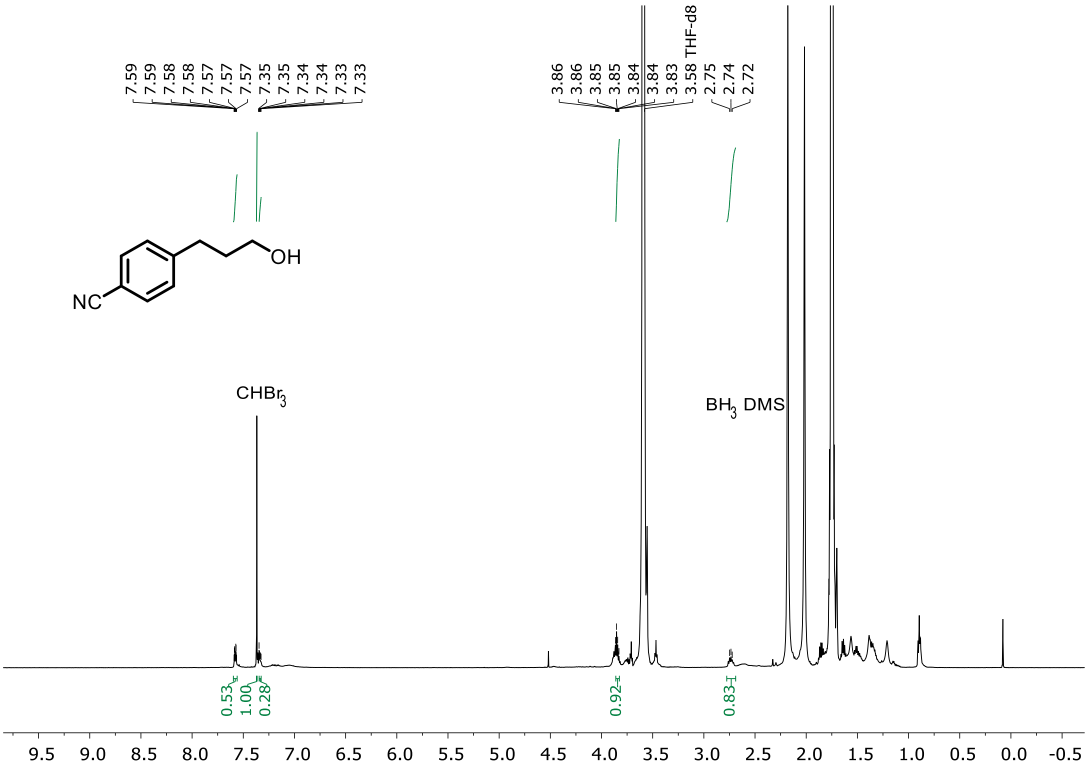

N-(3-hydroxypropyl)benzamide (2r-a)

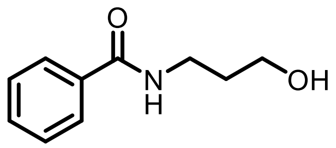

Prepared following general procedure 2 (conditions A), **2r-a** was obtained crude in 43% yield. NMR yield was determined using $CHBr_3$ as the internal standard.

^{1}H NMR (600 MHz, Chloroform-*d*) δ 7.77 (d, *J* = 7.6 Hz, 2H), 7.49 (d, *J* = 7.4 Hz, 1H), 7.42 (d, *J* = 7.6 Hz, 2H), 6.76 (s, 1H), 3.72 (d, *J* = 5.5 Hz, 2H), 3.63 (d, *J* = 6.2 Hz, 2H), 1.80 (d, *J* = 5.9 Hz, 2H).

These data are in close accordance with those previously reported.[16]

Spectrum of crude reaction mixture for *N*-(3-hydroxypropyl)benzamide (2r-a)

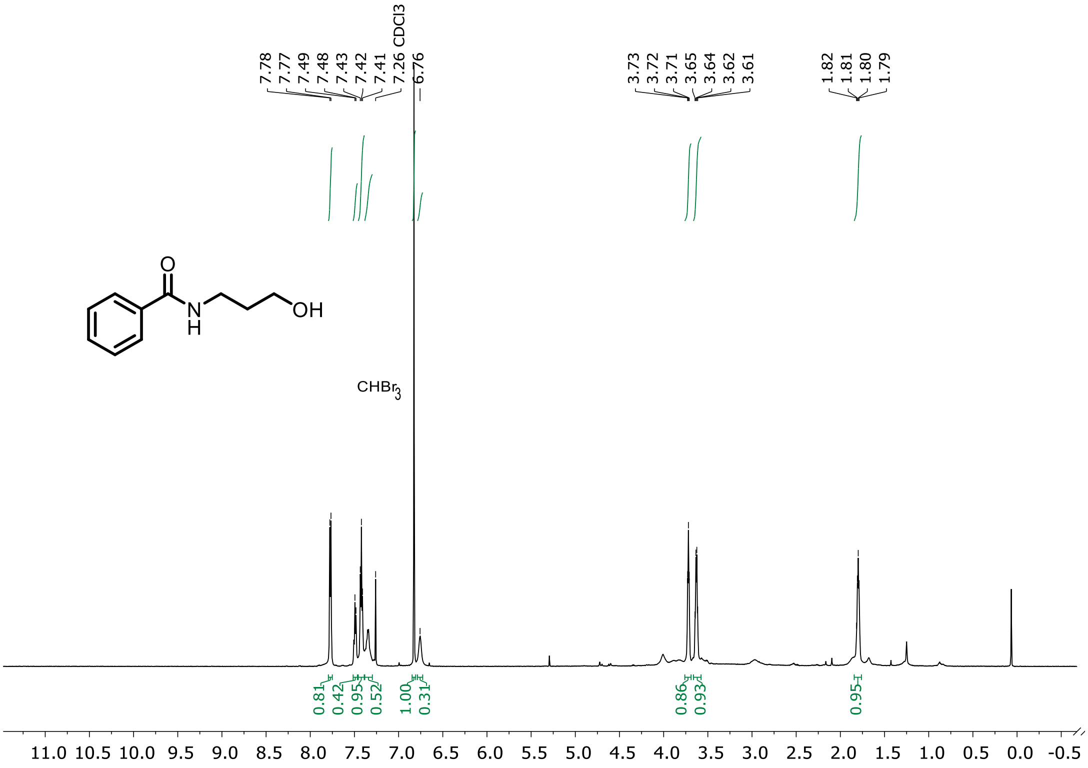

5. Mechanistic studies

5.1 Reaction with TMEDA

In an oven-dried 4 ml vial were introduced starting material **1a** (0.1 mmol, 1 eq, 19.22 mg) and a stirring bar. A cap with rubber septum was used to close the vial and the system was then purged with argon. 2-MeTHF or THF (c = 1.0 M, 0.1 mL), promoter, HBpin (5 eq, 0.5 mmol, 72.5 µL or 6 eq, 0.6 mmol, 87 µL) and TMEDA (1 eq, 0.1 mmol, 15.1 µL) were then added successively and it was left to stir at room temperature overnight. After this time, the solution was diluted with Et_2O and washed two times with distilled water, the combined aqueous layers were extracted two times with Et_2O and the combined organic layers were washed with brine, dried ($MgSO_4$), filtered and concentrated under vacuum.

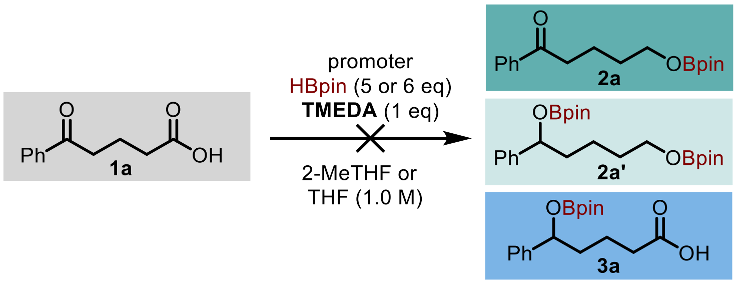

Table 6. Promoters used in the hydroboration reactions in the presence of TMEDA

Promoter x mol%	HBpin x eq	1a [%]
BNAH 5 mol%	5	15
BH_3·DMS 5mol%	5	25
KOtBu 1 mol%	6	60

5.2. NMR monitoring of the reaction

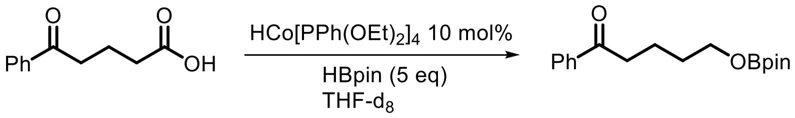

Hydroboration reaction with $HCo[PPh(OEt)_2]_4$ and HBpin was monitored *in situ* using ^1H NMR and ^{11}B NMR spectroscopy. In NMR tube were introduced, under inert conditions, $HCo[PPh(OEt)_2]_4$ (10 mol%, 30 µmol, 26 mg) starting material **1a** (57.7 mg, 1 eq, 0.3 mmol), THF-d_8 (c = 1.0 M, 0.3 mL), HBpin (5 eq, 1.5 mmol, 0.21 mL) and mesitylene as the internal standard (1 eq, 0.3 mmol, 41.55 µL). The reaction was monitored overnight, taking the spectra approximately every 6 minutes.

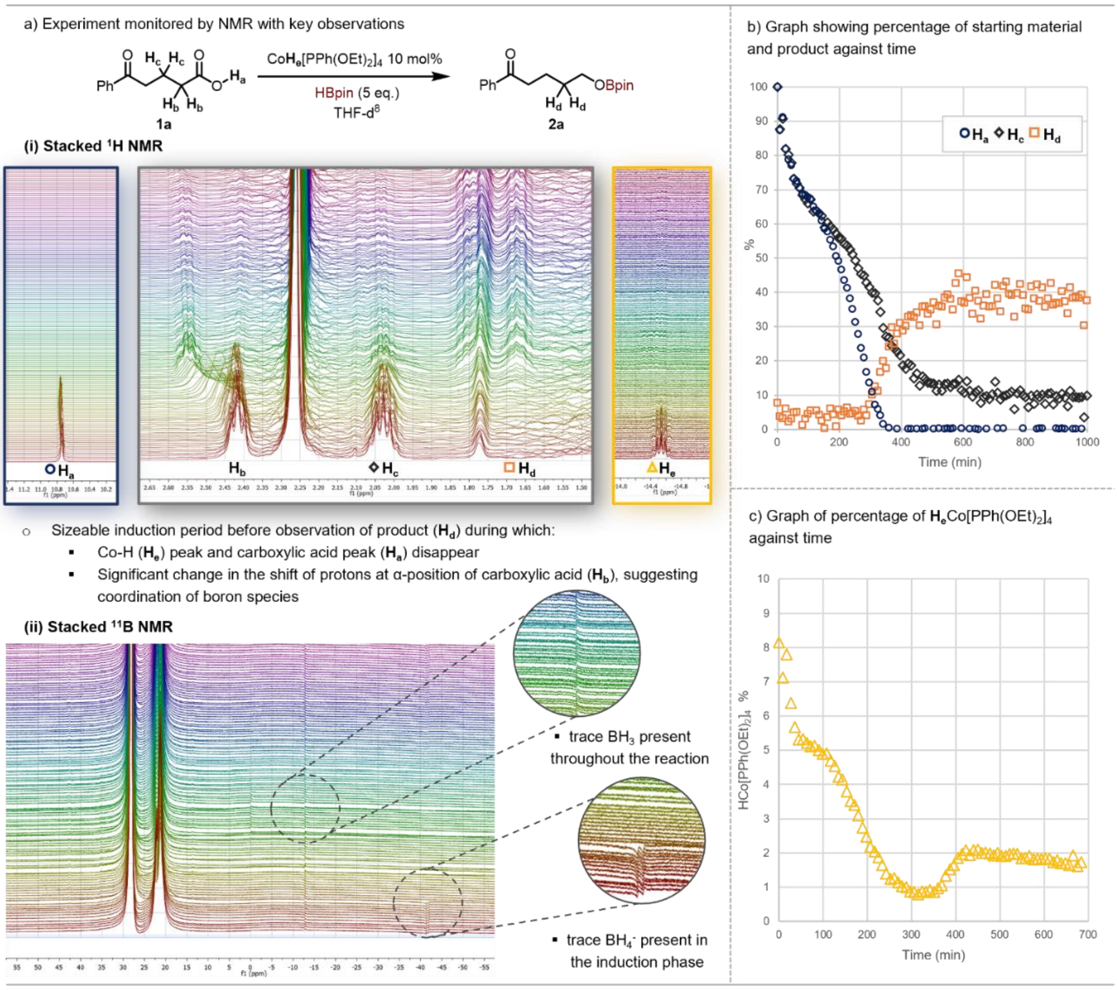

Figure S1: In situ NMR experiments with key features highlighted and graphs derived from integrals.

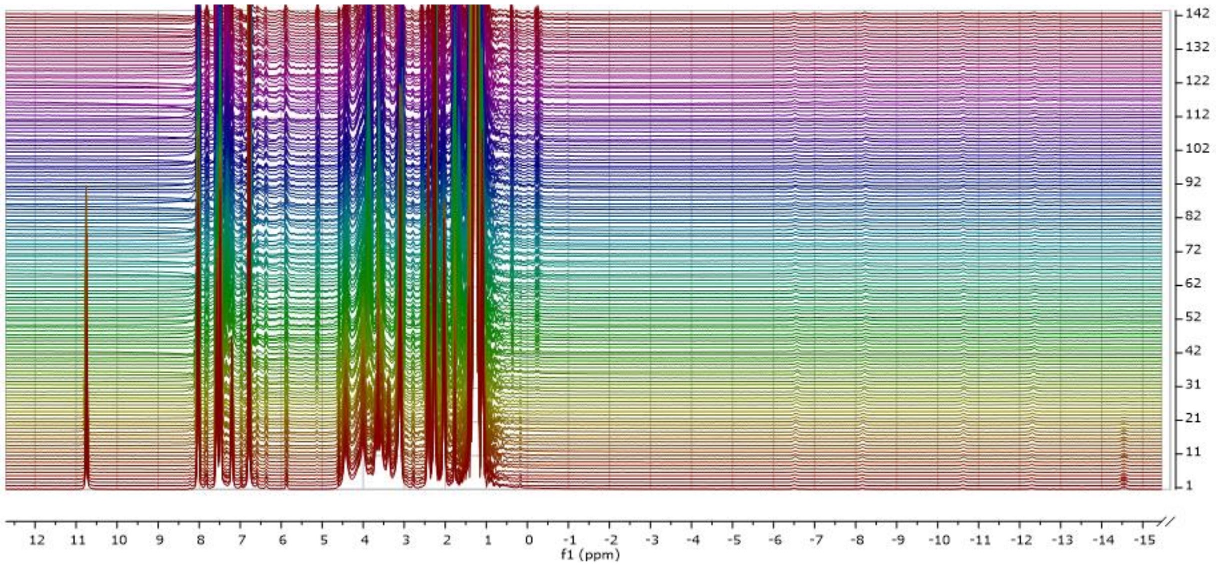

Figure S2a: Stacked ^{1}H NMR spectrum for hydroboration of 4-benzoyl-butyric acid with $HCo[PPh(OEt)_2]_4$ and HBpin

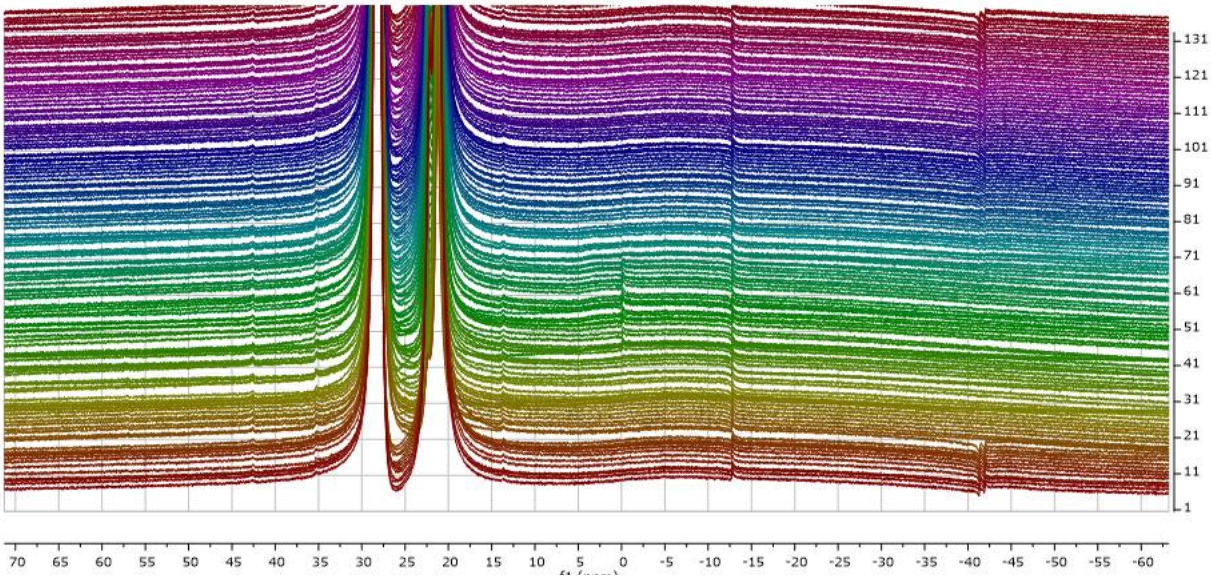

Figure S2b: Stacked ^{11}B NMR spectrum for hydroboration of 4-benzoyl-butyric acid with $HCo[PPh(OEt)_2]_4$ and HBpin

The following data was used to plot the graphs in Figure S1. These were obtained by extracting the integrals from Mestrenova and normalising against the internal standard (mesitylene).

Time (min)	H_a (%)	H_c (%)	H_d (%)	H_e (%)
0	85.9	98.0	8.3	8.1
9	80.5	91.1	3.3	7.1
18	83.2	93.8	3.9	7.8
27	76.2	86.3	5.9	6.4
36	72.2	82.8	2.1	5.7
45	70.0	80.3	3.4	5.3
54	69.6	79.8	6.5	5.3
63	69.1	78.0	5.4	5.2
72	69.0	78.6	1.3	5.1
81	68.7	77.8	2.3	5.1
90	68.6	76.5	4.0	5.0
99	68.2	76.4	6.3	4.9
108	67.7	76.9	4.7	4.9
117	66.7	75.6	1.9	4.7
126	65.5	74.7	5.3	4.5
135	64.4	73.8	4.2	4.2
144	62.9	74.1	3.3	4.1
153	61.4	72.6	3.4	3.8
162	59.7	72.6	3.6	3.5
171	57.9	72.5	7.2	3.4
180	56.0	72.2	5.9	3.1
189	53.9	71.2	2.4	2.7
198	51.7	69.5	6.9	2.5
207	49.2	69.4	4.5	2.2
216	46.3	68.8	6.4	2.0
225	43.4	67.8	6.5	1.9
234	40.4	66.6	4.7	1.6
243	37.1	65.8	6.2	1.4
252	33.7	64.6	6.8	1.2
261	30.2	62.3	7.0	1.3
270	26.5	61.7	5.8	1.1
279	22.9	60.6	7.6	1.0
288	19.2	59.8	7.7	1.0
297	15.8	57.6	10.7	0.9
306	12.5	55.4	12.5	0.9
315	9.7	55.0	12.1	0.8
324	7.0	52.5	12.5	0.9
333	5.1	48.7	18.2	0.9
342	3.4	44.9	17.1	0.9
351	2.4	43.1	20.2	0.9
360	1.6	41.4	21.5	1.0
369	1.2	39.4	23.8	1.1
378	0.9	36.0	29.4	1.3

387	0.8	35.7	25.9	1.5
396	0.8	34.3	28.2	1.6
405	0.8	32.7	32.2	1.9
414	0.8	30.9	32.6	2.0
423	0.8	29.8	34.5	2.1
432	0.9	29.7	32.6	1.9
441	0.9	28.5	33.7	2.1
450	0.8	25.4	36.3	2.1
459	0.9	24.9	34.7	2.0
468	0.9	24.3	37.0	2.0
477	1.0	23.8	36.5	2.0
486	1.0	22.5	37.0	1.9
495	0.9	22.6	37.2	2.0
504	1.0	22.1	38.6	1.9
513	0.9	20.4	37.6	1.9
522	1.0	20.2	36.2	2.0
531	0.9	19.9	37.4	2.0
540	1.0	20.1	41.4	2.0
549	1.1	19.9	40.1	1.8
558	1.0	19.6	38.7	1.9
567	0.9	18.4	40.5	1.9
576	1.1	19.3	35.6	1.8
585	1.0	18.1	41.5	1.8
594	1.0	18.4	42.2	1.8
603	1.0	16.9	39.0	1.8
612	1.0	17.5	40.1	1.8
621	1.0	17.5	41.4	1.7
630	1.0	16.5	40.1	1.8
639	1.1	18.0	39.5	1.7
648	1.0	16.4	43.5	1.7
657	1.1	16.7	38.9	1.6
666	0.9	14.6	37.3	1.9
675	1.1	16.8	40.8	1.6
684	0.9	14.0	42.2	1.7
693	0.9	16.2	41.2	1.8
702	0.8	14.3	37.4	1.9
711	1.0	16.6	38.9	1.7
720	1.0	14.6	38.3	1.6
729	1.0	16.0	40.4	1.6
738	0.9	13.4	42.0	1.7
747	0.9	13.9	44.3	1.6
756	0.9	14.4	40.6	1.6
765	0.8	13.5	37.1	1.8
774	0.8	14.0	38.9	1.7
783	1.0	14.2	39.7	1.4
792	0.9	14.0	41.3	1.6

801	0.9	13.6	38.7	1.6
810	0.9	13.5	37.2	1.5
819	0.9	13.6	40.5	1.5
828	0.9	13.4	42.7	1.5
837	0.9	12.1	41.4	1.5
846	0.9	13.7	43.3	1.6
855	0.8	12.6	37.6	1.5
864	0.9	13.7	42.0	1.5
873	0.9	13.7	40.5	1.4
882	0.8	13.2	37.7	1.5
891	0.9	12.1	40.1	1.5
900	0.8	13.4	41.3	1.5
909	0.8	13.2	38.8	1.5
918	0.8	12.2	38.5	1.4
927	0.8	13.6	41.1	1.5
936	0.8	12.5	38.6	1.4
945	0.9	12.9	39.0	1.3
954	0.9	12.5	39.6	1.4
963	0.8	12.2	38.9	1.5
972	0.8	12.6	41.5	1.5
981	0.7	12.4	41.2	1.4
990	0.7	12.3	40.5	1.5
999	0.8	11.8	40.0	1.4
1008	0.8	11.9	38.2	1.3
1017	0.8	12.3	39.8	1.4
1026	0.8	12.1	39.2	1.4
1035	0.8	13.0	39.9	1.4
1044	0.7	11.3	41.2	1.5

5.3 NMR monitoring of reduction reactions promoted by borane catalysis

In situ NMR monitoring of the hydroboration reactions were carried out for two substrates: **1b** and **1c.** These reactions were carried under the optimized conditions (KO^{t}Bu 1 mol%, HBpin 6 eq, THF-d_8 1M, under inert atmosphere, over 17 h) on 0.3 mmol scale, using mesitylene (41.6 µl, 1 eq, 0.3 mmol) as the internal standard.

Reaction schemes and stacked spectra are presented below.

Compound **1b**:

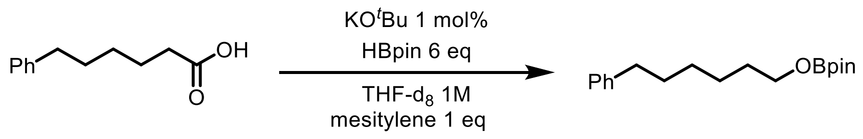

Stacked spectra for compound 1b:

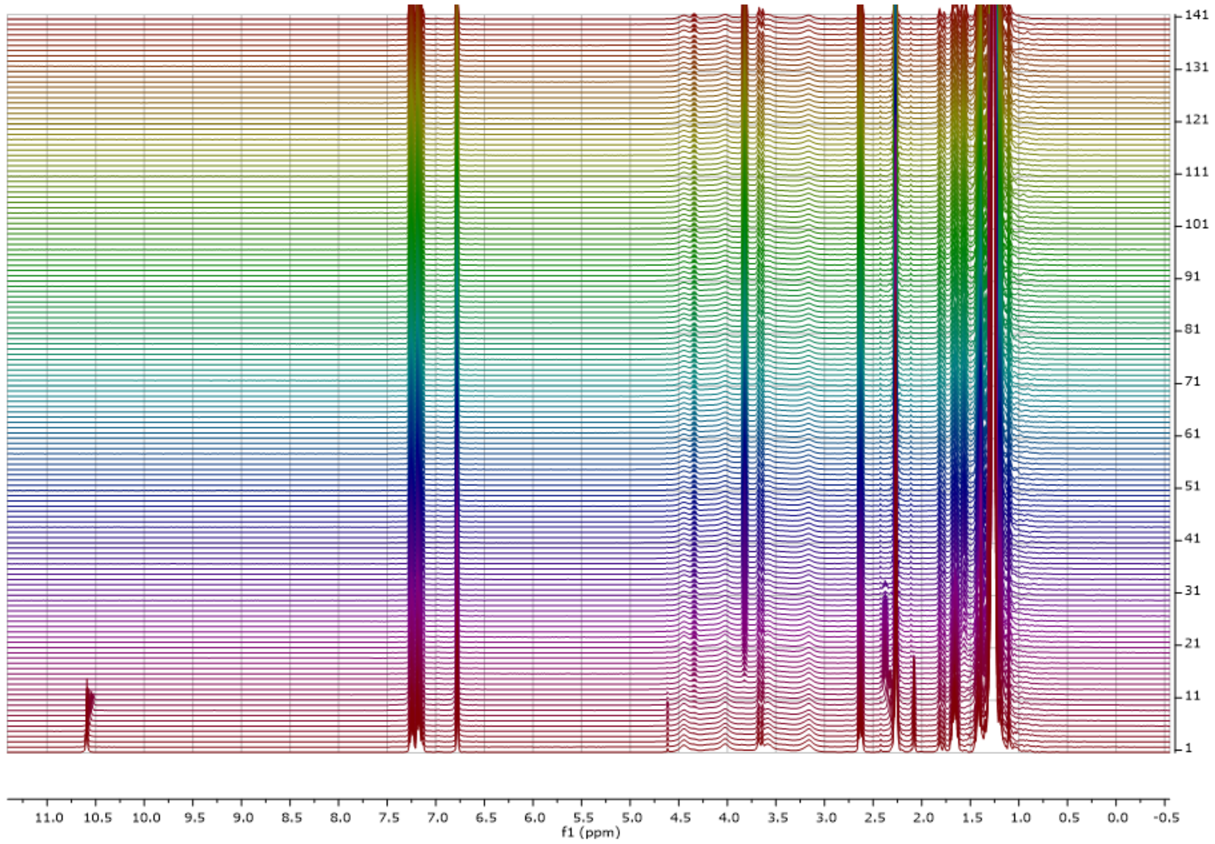

Figure S3: Stacked ^{1}H NMR spectrum for reduction of **1b**

Similar disappearance of the acidic proton (δ 10.6 ppm) and deshielding of the protons at the α-position to the carboxylic acid peak (δ 2.3 ppm) is observed before product formation.

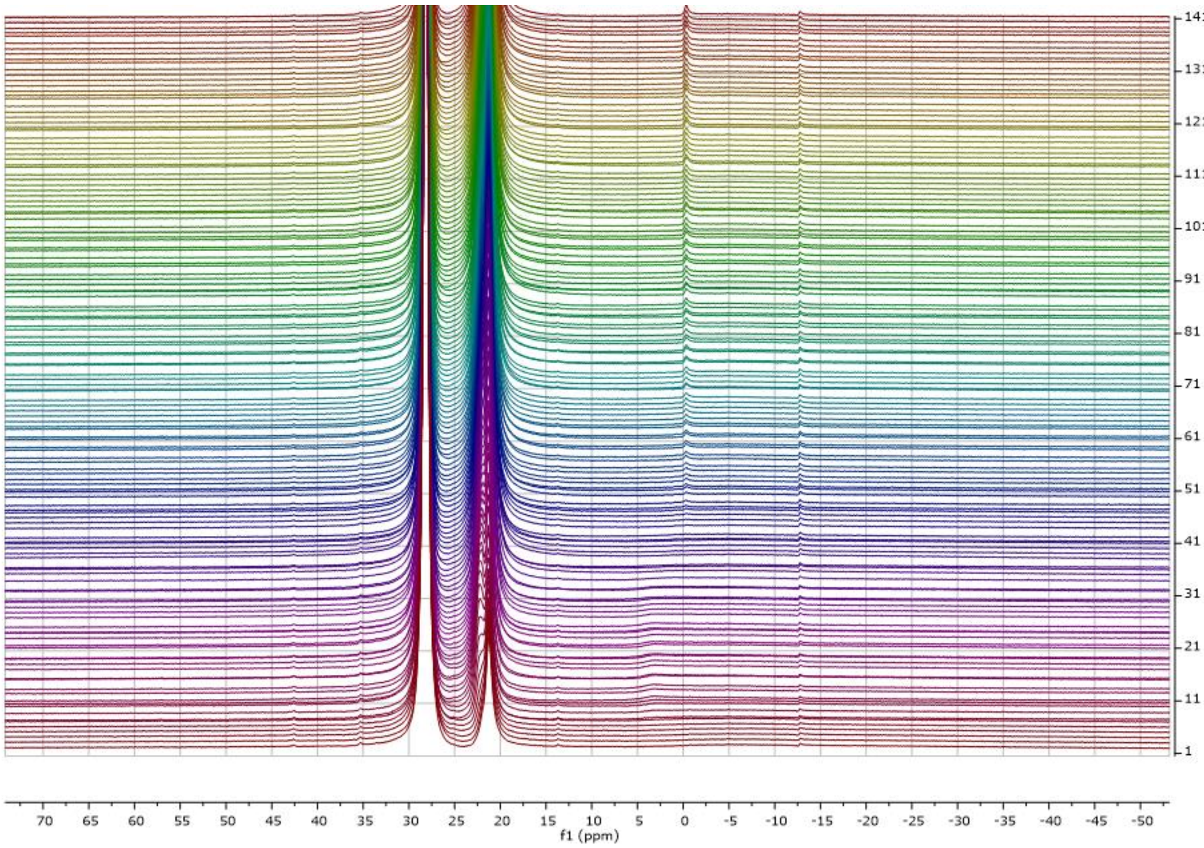

Figure S4: Stacked ^{11}B NMR spectrum for reduction of **1b**

A small peak corresponding to BH_3 (δ -12 ppm) is again present throughout the reaction.

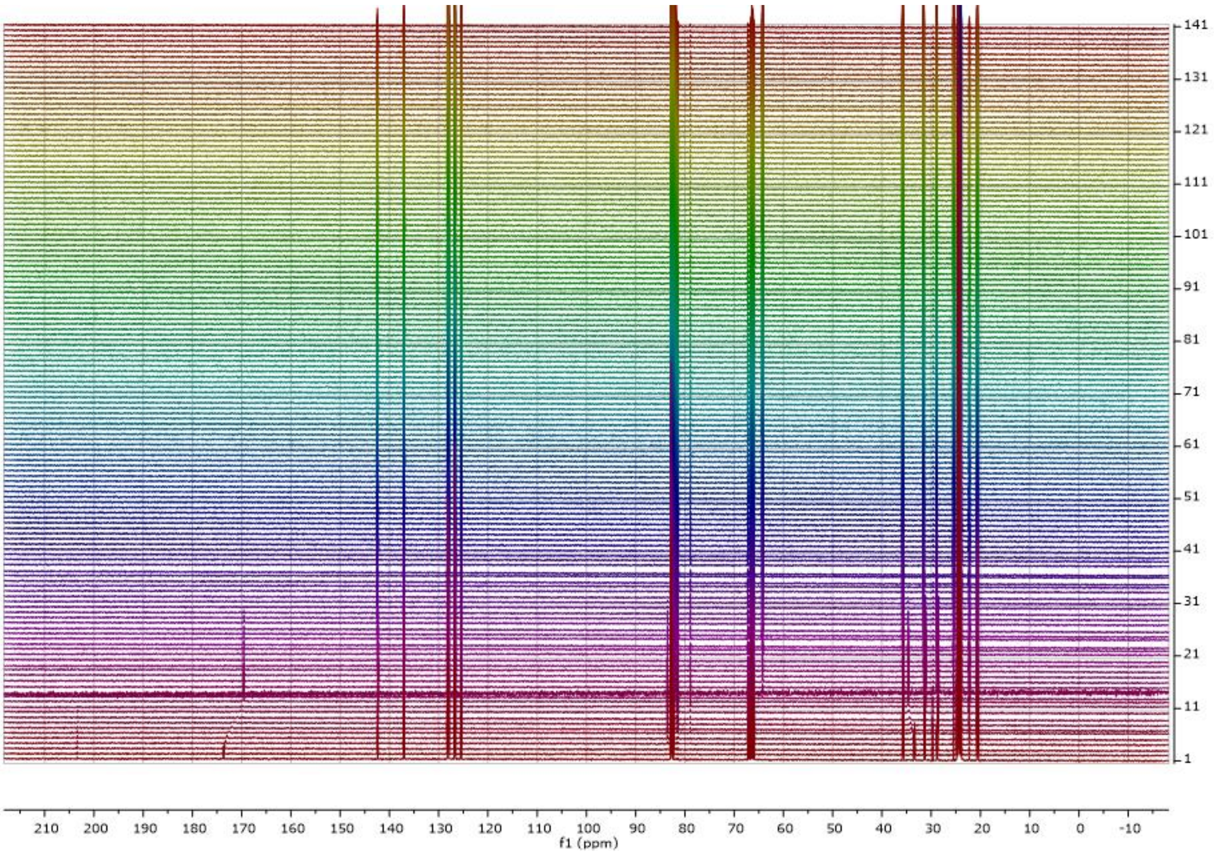

Figure S5: Stacked ^{13}C NMR spectrum for reduction of **1b**

Shielding of the carbonyl peak (δ 174 ppm) is observed in the induction phase of the reaction – consistent with our proposed mechanism.

Compound **1c**:

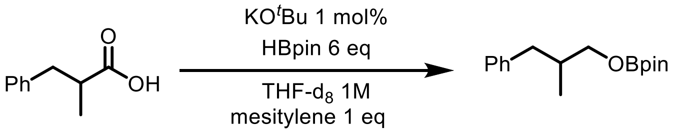

Stacked spectra for compound 1c:

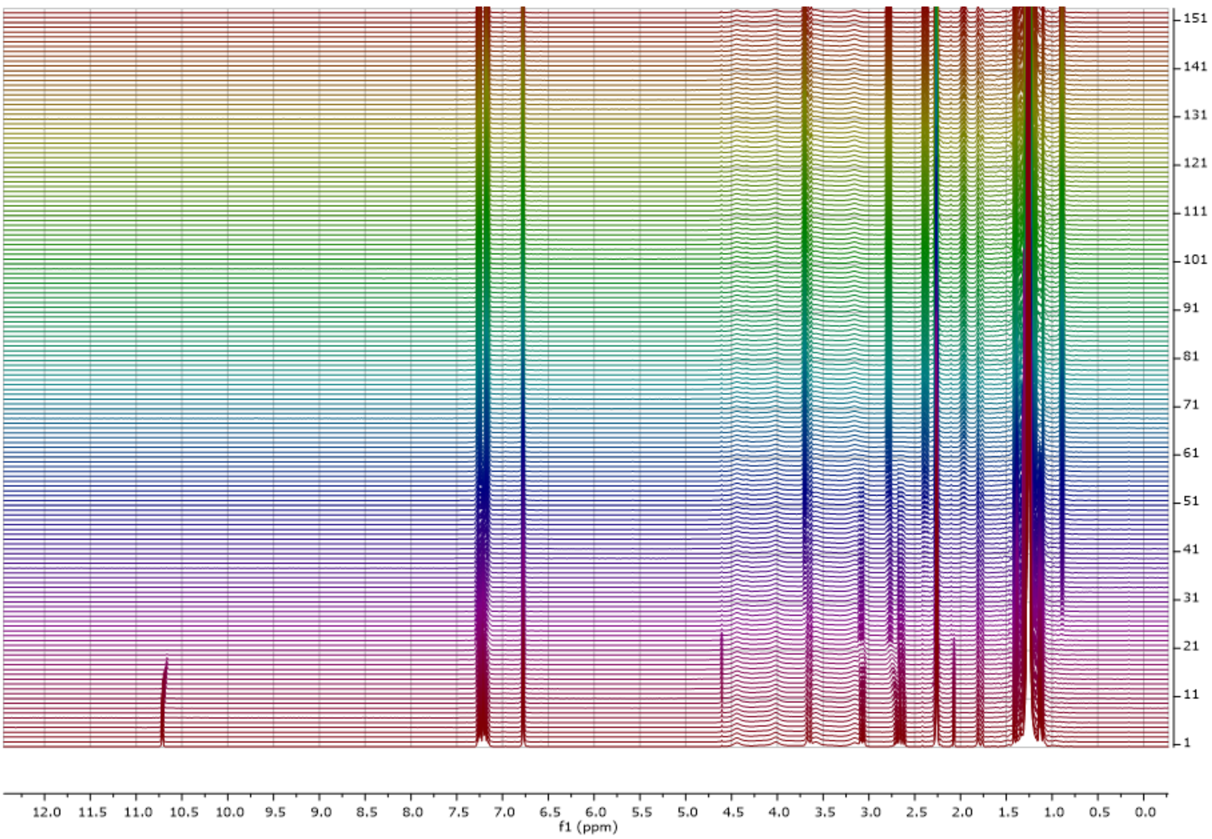

Figure S6: Stacked ^{1}H NMR spectrum for reduction of **1c**

Similar disappearance of the acidic proton (δ 10.7 ppm) and deshielding of the protons at the α-position to the carboxylic acid peak (δ 2.7 ppm) is observed before product formation.

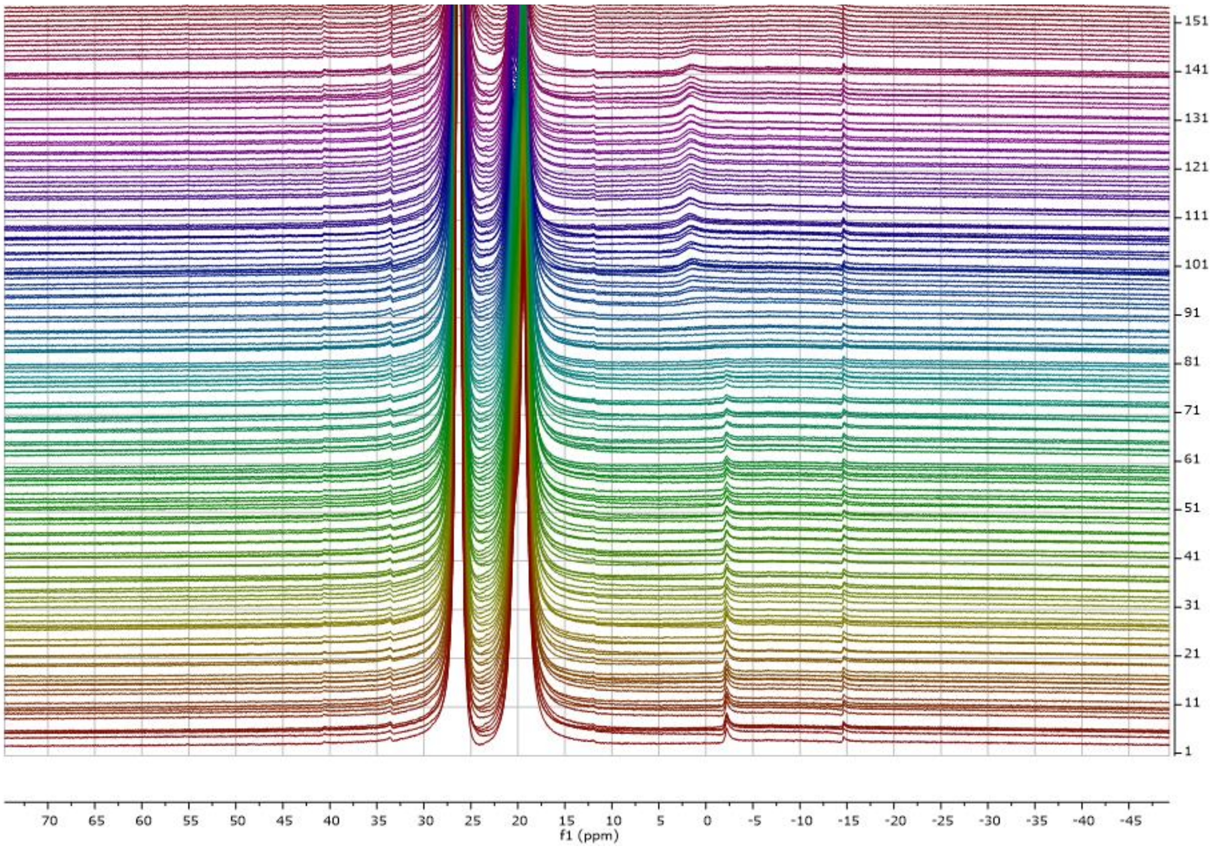

Figure S7: Stacked ^{11}B NMR spectrum for reduction of **1c**

A small peak corresponding to BH_3 (δ -12 ppm) is again present throughout the reaction.

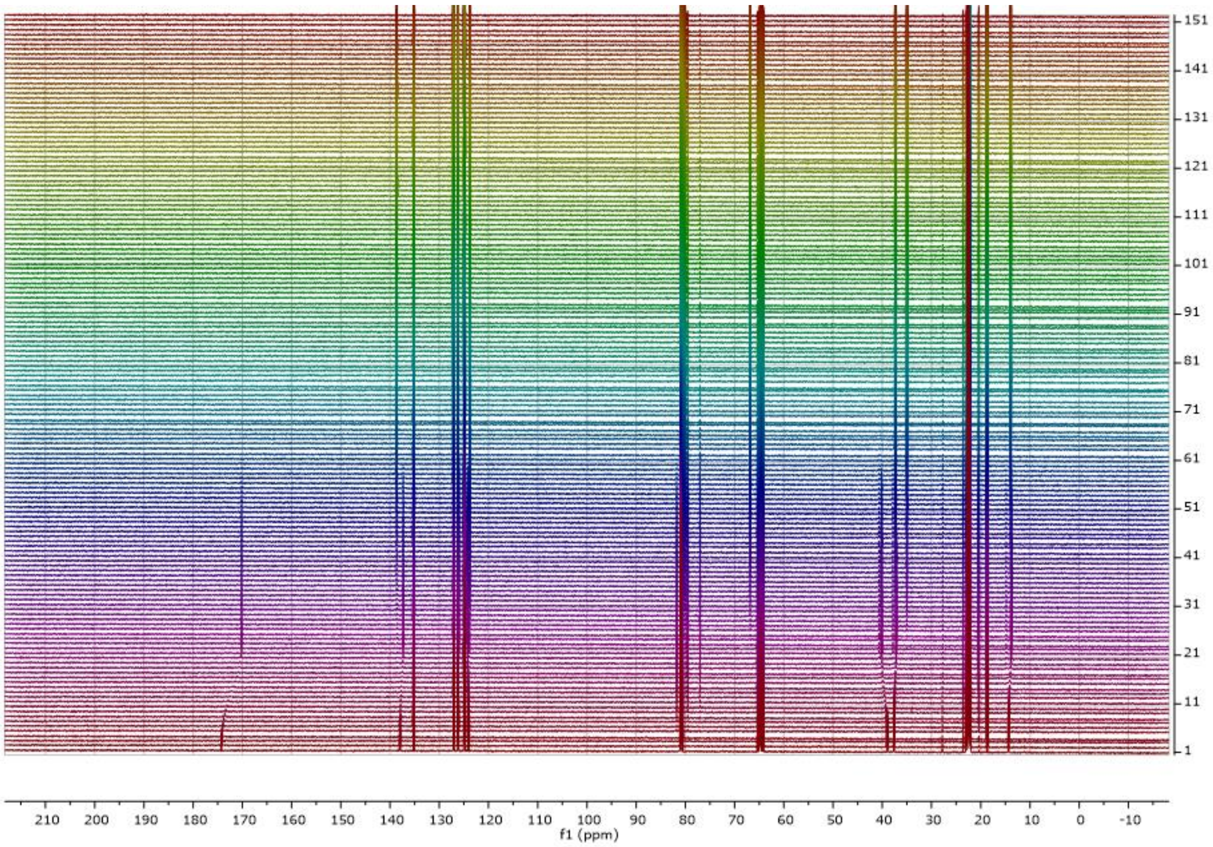

Figure S8: Stacked ^{13}C NMR spectrum for reduction of **1c**

Shielding of the carbonyl peak (δ 174 ppm) is observed in the induction phase of the reaction – consistent with our proposed mechanism. There appear to be diastereomeric intermediates which could correlate to proposed intermediate V.

6. Computational details

All the Density Functional Theory calculations were carried out using Gaussian16 program package.[18a] All the structures were optimized using the B3LYP functional including Grimme empirical dispersion correction with the standard 6-31+G(d) basis set.[18b] The nature of stationary points was confirmed by frequency calculations as minima (no imaginary frequencies) or transition states (one imaginary frequency). Transition states were further verified by relaxing the imaginary frequency towards the reactant and the product and doing IRC calculations when needed. In addition, single point calculations using the M062X 6-311++G(3d,2p). Solvation[19] was included in both optimizations and single point calculations using the CPCM implicit solvent model[20] and tetrahydrofuran as solvent. 1M standard state correction was considered by adding 1.89 kcal/mol to all the species except HBPin, where 6 M was used to reproduce the large number of equivalents used (2.96 kcal/mol). Finally, a comparison between B3LYP/6-311++G(3d,2p) and M062X/6-311++G(3d,2p) methods was performed to validate the results and all the methods yield very similar results. 3D structures were illustrated using the CYLview 1.0 program.[21]

Energy Profiles

Direct hydride insertion from HBpin

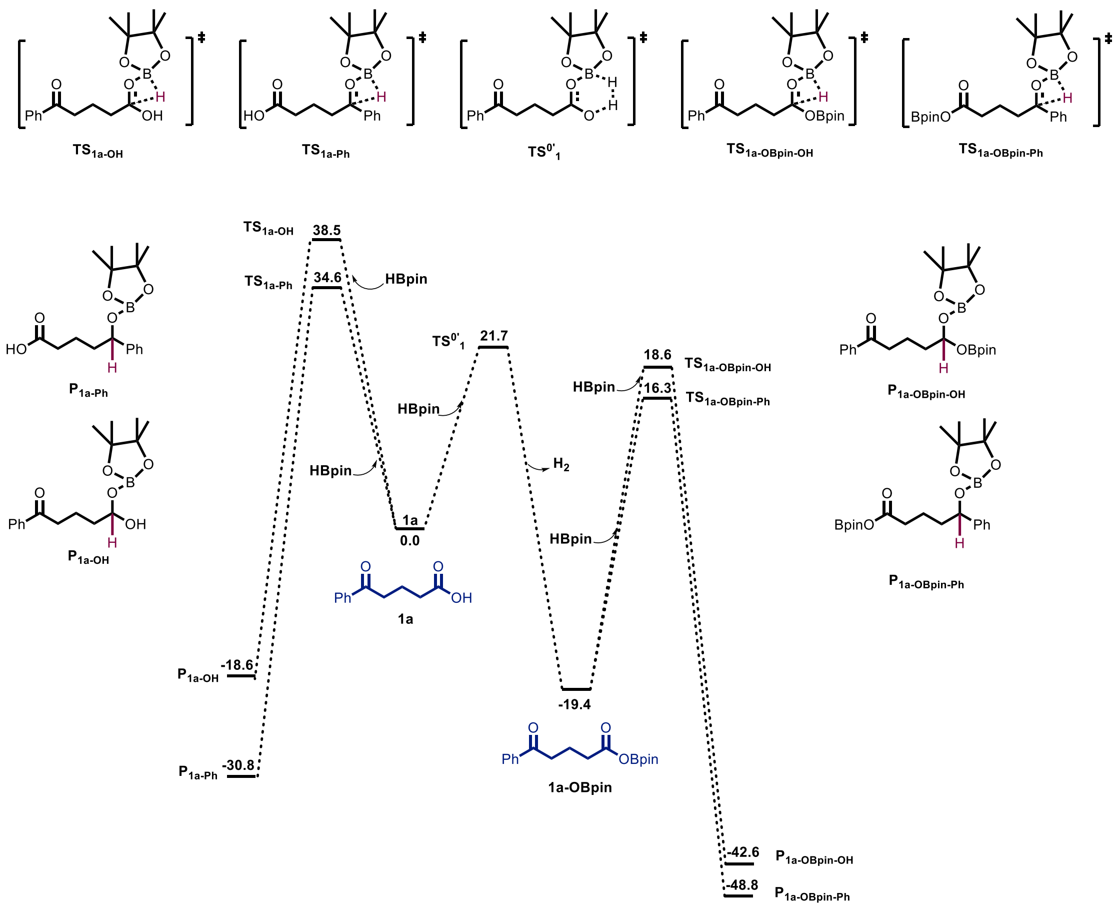

Figure S9: Free energy profile depicting the direct hydride insertion from HBpin⁻ to both carbonyl sites of **1a** and **1a**$_{\mathbf{OBpin}}$.

Borohydride attack with previous BH_3 coordination

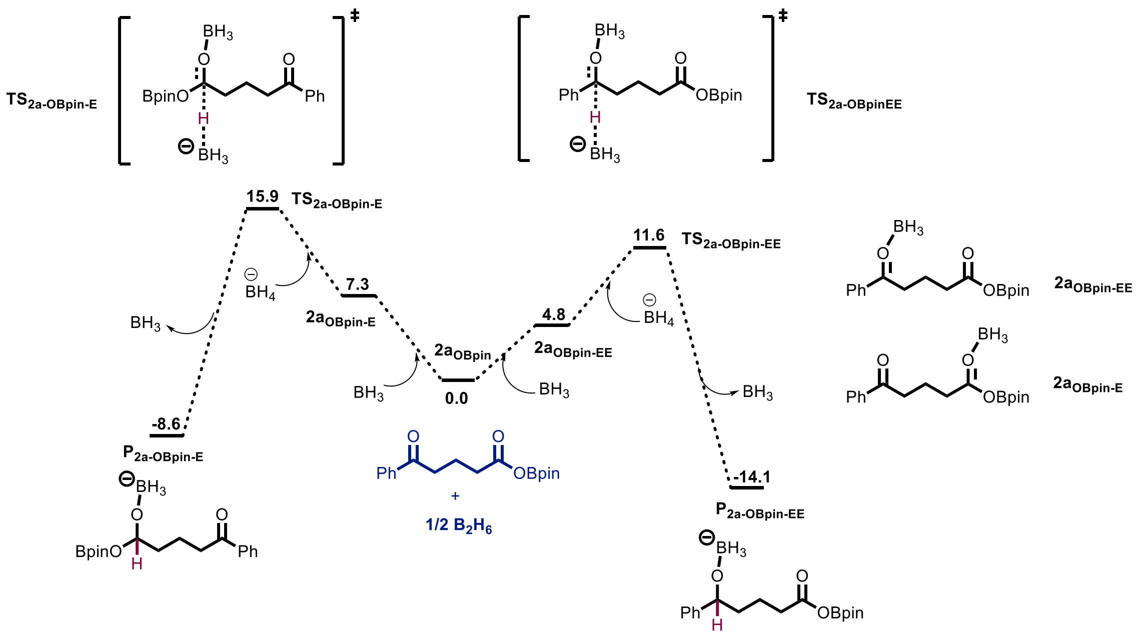

Figure S10: Free energy profile depicting BH_4^- attack to both carbonyl groups.

Benchmarking study

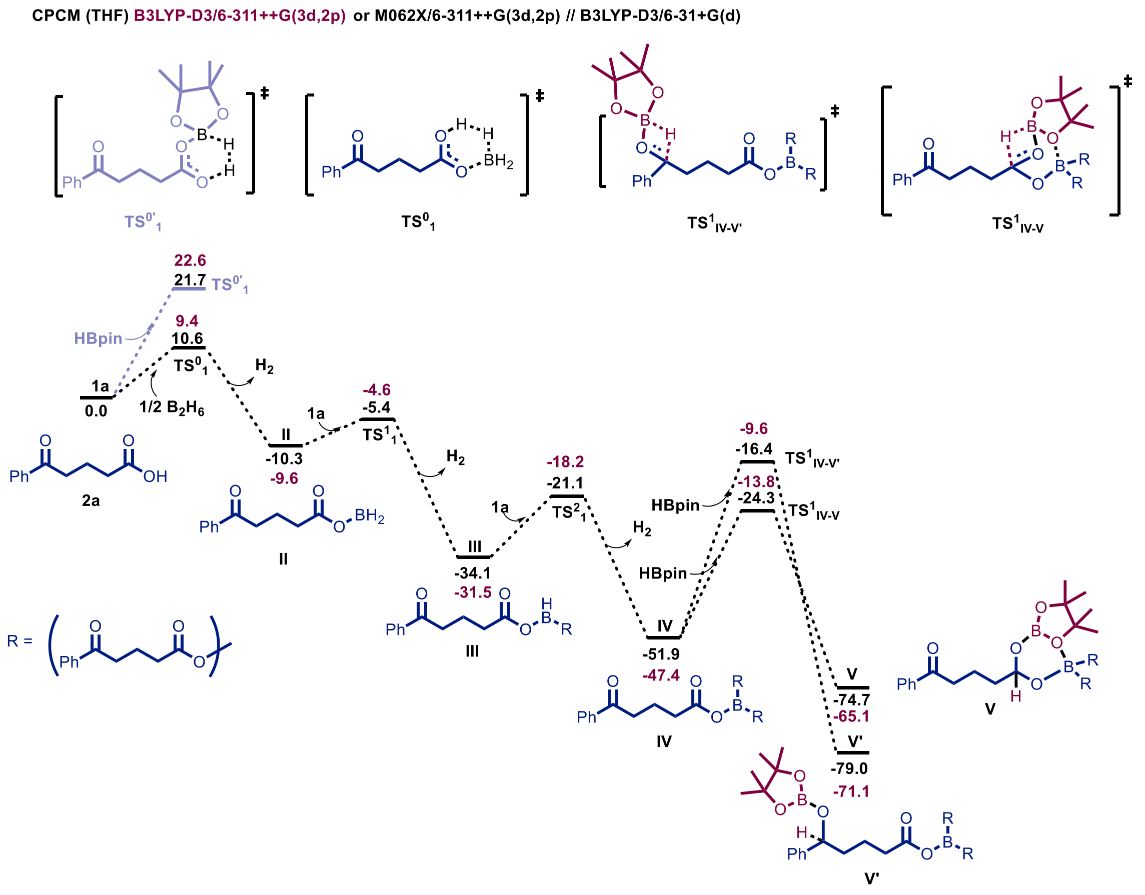

Figure S11. Benchmarking study of B3LYP (purple) and M06-2X (black) on the full free energy profile of the of hydroboration reduction mediated by BH_3.

HBpin-O^{t}Bu attack without previous coordination

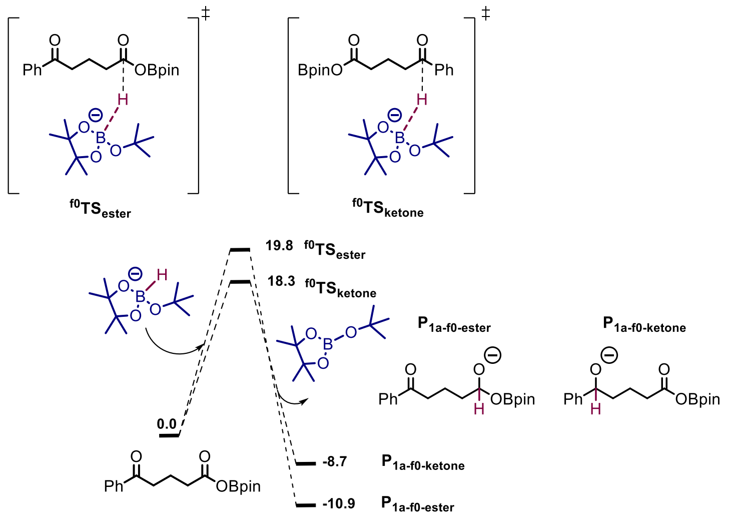

Figure S12: Free energy profile depicting HBpin-O^{t}Bu attack to uncoordinated substrate.

Comment: The energy profile shows the reverse trend to what is observed experimentally.

Direct HBpin-O^{t}Bu attack with BH_3 coordination

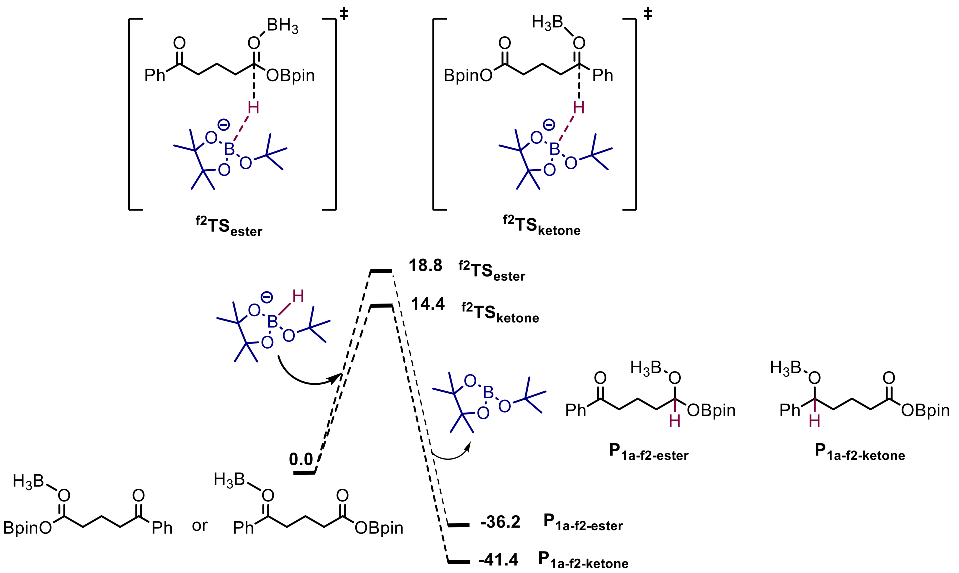

Figure S13: Free energy profile depicting HBpin-O^{t}Bu attack to coordinated substrate (BH_3)

Comment: The energy profile shows the reverse trend to what is observed experimentally.

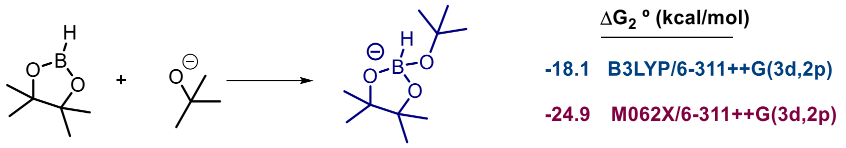

Figure S14: Formation of HBpin-O^{t}Bu from reaction between O^{t}Bu and HBpin.

Comment: Although HBpin-O^{t}Bu formation is exergonic (**Figure S14**), the selectivity (**Figures S12** & **S13**) suggest that direct reduction with this species is not a relevant pathway, when comparing to the experimentally observed results. Additionally, these pathways are unlikely, because the low amount of base (1 mol%) means the borohydride species would be formed in only very small quantities and subsequent decomposition to BH_3 has been detected experimentally.

XYZ Coordinated and Energies of the Calculated Species

Final free energies are calculated as the sum of E (large basis set: M02-6X) + $G_{Corr.}$.

Boron species

BH_4^-

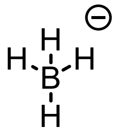

E= -27.326960075

G_{Corr}= 0.01389

-1 1

B	-0.00019600	0.00016900	0.00004400
H	0.74172200	0.43465700	-0.89090900
H	0.68655000	-0.37536400	0.95936400
H	-0.77180500	0.89503700	0.36847400
H	-0.65548900	-0.95517400	-0.43714800

B_2H_6

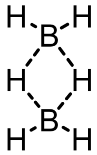

E= -53.2580896095

G_{Corr}= 0.040112

0 1

B	-0.88036000	0.00006600	0.00003300
B	0.88038100	0.00008300	-0.00004200
H	-0.00005500	0.00025600	-0.97794700
H	-0.00012500	0.00041800	0.97795400
H	1.46108700	1.04060400	0.00003800
H	1.46001200	-1.04104600	0.00011900
H	-1.46146200	1.04033600	-0.00009400
H	-1.45956000	-1.04131400	-0.00002300

HBpin

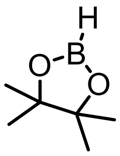

E(M026X) = -411.831630737

E(B3LYP) = -412.049153526

G_{Corr}= 0.15797

0 1

C	-0.78514900	-0.19413800	-0.04998900
C	0.78514500	-0.19414700	0.04998700
H	0.00002900	3.13883500	0.00002600
B	0.00001700	1.94901900	0.00002500
C	1.48203000	-0.46781800	-1.28508700
H	2.55260100	-0.26911000	-1.17417600
H	1.35585200	-1.51138100	-1.58996900
H	1.09519800	0.17719800	-2.08029800
C	1.35824200	-1.09088900	1.13979400
H	1.09484100	-2.13718300	0.94976200
H	2.45010700	-1.01130900	1.14374600
H	0.99046900	-0.81242500	2.12984400
C	-1.48204100	-0.46787900	1.28506600
H	-2.55261000	-0.26915400	1.17416200
H	-1.35587700	-1.51146000	1.58988900
H	-1.09520800	0.17708800	2.08031600
C	-1.35824300	-1.09082000	-1.13984500
H	-1.09486400	-2.13712700	-0.94986200
H	-2.45010600	-1.01121600	-1.14381100
H	-0.99044700	-0.81231100	-2.12987600
O	1.07751400	1.20489800	0.39240200
O	-1.07751100	1.20492800	-0.39233100

Substrates

1a

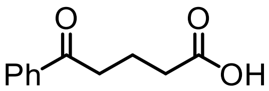

E(M026X)= -652.044764252

E(B3LYP)= -652.365810218

G_{Corr}= 0.168254

0 1

C	-0.70938100	0.69932500	-0.00013700
C	0.43386900	-0.30237800	-0.00016100
C	1.81573000	0.34807600	-0.00009400
H	0.31716100	-0.95731900	0.87470500
H	0.31719200	-0.95715500	-0.87516300
C	2.92112700	-0.70676800	0.00009300
H	1.92085700	0.99519100	-0.87663100
H	1.92070500	0.99533300	0.87635500
H	2.84230900	-1.36573100	0.87499600
H	2.84238700	-1.36599600	-0.87461600
C	-2.11444700	0.18430500	-0.00005200
C	-2.40990300	-1.18924100	-0.00010000
C	-3.17146600	1.11055200	0.00010300
C	-3.73592700	-1.62571200	-0.00001700
H	-1.61323300	-1.92521700	-0.00021100
C	-4.49449400	0.67486700	0.00019300
H	-2.93712400	2.16989900	0.00014800
C	-4.77961500	-0.69586300	0.00012800
H	-3.95334400	-2.68989300	-0.00006400
H	-5.30377100	1.39951600	0.00030600
H	-5.81115400	-1.03693900	0.00020300
O	-0.48779000	1.90784600	-0.00022500
C	4.31113800	-0.11896000	0.00010100
O	4.59097900	1.06553500	0.00018700
O	5.26205200	-1.08375400	-0.00000400
H	6.13629800	-0.64791500	-0.00002800

1a$_{OBpin}$

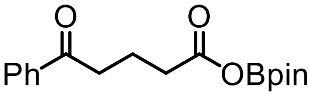

E= -1062.73214962

G_{Corr}= 0.326257

0 1

C	4.20366700	-0.72701200	-0.09667600
C	2.99132400	0.18951200	-0.09539700
C	1.66294900	-0.55055200	-0.23829900
H	3.00573500	0.77680700	0.83354400
H	3.11277300	0.92069500	-0.90690800
C	0.48806500	0.42558300	-0.23813400
H	1.66047400	-1.13341800	-1.16499500
H	1.54843500	-1.26955100	0.57914900
H	0.46184800	1.01920400	0.68599200
H	0.57445100	1.15667900	-1.05310000
C	5.56364100	-0.11656100	0.03630600
C	5.75436100	1.27002200	0.15851200
C	6.68530800	-0.96333300	0.03843100
C	7.04119900	1.79731900	0.28008400
H	4.90607300	1.94578900	0.15973300
C	7.96930800	-0.43703700	0.15978100
H	6.53166600	-2.03320800	-0.05612300
C	8.14988900	0.94603200	0.28093000
H	7.17737000	2.87081600	0.37387200
H	8.82942000	-1.10052700	0.16025700
H	9.15082400	1.35780300	0.37554300
O	4.07365800	-1.94402300	-0.20400200
C	-0.85512000	-0.24484800	-0.37637600
O	-1.05021400	-1.43766000	-0.46717700
O	-1.86284500	0.67287500	-0.42727300
C	-5.43821900	0.77239800	-0.19958800
C	-5.11766300	-0.59189300	0.52102200
B	-3.20299100	0.38263300	-0.20254900
C	-5.35368100	-1.81691500	-0.36461600
H	-4.93356400	-2.69851300	0.12901300
H	-6.42293700	-1.98727300	-0.52363700
H	-4.86907700	-1.70821100	-1.34001500
C	-5.78867400	-0.77416500	1.87517800
H	-6.87832500	-0.76992600	1.76352000
H	-5.49501900	-1.73787600	2.30326000
H	-5.50597600	0.01382300	2.57666600
C	-5.69482500	1.92617500	0.77185100
H	-5.72224800	2.86490300	0.20993400
H	-6.65408900	1.80342900	1.28401300
H	-4.90493600	2.00095100	1.52612100
C	-6.54149200	0.69930900	-1.24593900
H	-7.48743400	0.40109100	-0.78120400
H	-6.68252500	1.68515500	-1.70041600
H	-6.30120900	-0.01303900	-2.03819600
O	-3.66642900	-0.49871800	0.72893700
O	-4.17004500	1.06965400	-0.87869700

1a$_{OBpin-E}$

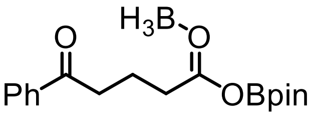

E= -1089.35815036

G_{Corr}= 0.35646

0 1

C	-3.89165700	-0.43609500	-0.46549000
C	-2.91007100	0.42173500	0.31773700
C	-1.47113000	0.28456700	-0.17265100
H	-3.23509000	1.46880900	0.25543500
H	-2.98136100	0.14874800	1.37955000
C	-0.52583600	1.16259300	0.68191100
H	-1.15309700	-0.76079800	-0.11597600
H	-1.40589600	0.58789600	-1.22160400
H	-0.83349200	2.20916500	0.63494600
H	-0.54036500	0.82518600	1.72234100
C	-5.34003300	-0.37960400	-0.09948900
C	-5.81861600	0.43330800	0.94175400
C	-6.24946400	-1.16829000	-0.82495400
C	-7.17974600	0.45533600	1.25032100
H	-5.13857800	1.05212000	1.51699700
C	-7.60766800	-1.14550000	-0.51710300
H	-5.87393900	-1.79324200	-1.62836600
C	-8.07579900	-0.33282100	0.52244900
H	-7.53946800	1.08719000	2.05715200
H	-8.30241200	-1.75831600	-1.08432600
H	-9.13502100	-0.31422600	0.76337100
O	-3.50177000	-1.16011100	-1.37813900
C	0.87108300	1.05851800	0.17106100
O	1.39793600	1.82993600	-0.65265900
O	1.55401500	0.01486900	0.59753200
C	4.84562900	-1.32346000	0.00888100
C	5.14400000	0.21815700	0.15737100
B	2.93229000	-0.17685500	0.36074700
C	5.34085500	0.93376600	-1.17942400
H	5.35051400	2.01394100	-1.00614500
H	6.29222700	0.64934100	-1.63887700
H	4.53285600	0.70808900	-1.88215900
C	6.26772100	0.56068900	1.12373500
H	7.21035800	0.12424800	0.77672900
H	6.39297400	1.64689400	1.17081000
H	6.06221100	0.19339400	2.13144500
C	5.13695500	-2.12635700	1.27675700
H	4.73428600	-3.13686000	1.15852800
H	6.21422100	-2.20346000	1.45182800
H	4.67281100	-1.67252900	2.15814100
C	5.48285500	-1.98195200	-1.20545200
H	6.57419300	-1.91275100	-1.14290000
H	5.21033600	-3.04152300	-1.23430400
H	5.15567600	-1.51628600	-2.13744100
O	3.87662100	0.72698400	0.71672400
O	3.37945900	-1.34195500	-0.16610900
B	0.71122800	3.14219400	-1.29610700
H	1.53992900	3.52448900	-2.08982800
H	-0.32157500	2.77003800	-1.81146600
H	0.53556200	3.90842100	-0.37035100

1a$_{OBpin-EE}$

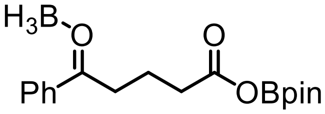

E= -1089.362614

G_{Corr}= 0.356841

0 1

C	-0.76537300	-0.59295300	0.07389800
C	0.43173400	-1.02391700	-0.73621600
C	1.75849300	-0.76822900	-0.02587400
H	0.30873000	-2.08730100	-0.97660100
H	0.38664800	-0.49294800	-1.69671000
C	2.94381500	-1.20682100	-0.91885700
H	1.84947400	0.29355100	0.22310400
H	1.78767400	-1.32382800	0.91613400
H	2.83746400	-2.26114200	-1.18302900
H	2.95015300	-0.62340900	-1.84325400
O	-0.72876200	-0.04756100	1.15589300
C	4.23902700	-1.03749500	-0.18494100
O	4.74134500	-1.99494900	0.45868300
B	4.18928500	-3.47635600	0.59221700
H	4.96386700	-4.00318700	1.36063500
H	3.05720600	-3.40233900	1.02665600
H	4.22518800	-3.96017000	-0.52439500
O	-1.92824900	-0.92484400	-0.54995500
C	-4.80351700	1.03100300	0.45238000
C	-5.43256100	-0.22027600	-0.27044300
B	-3.16978100	-0.37708000	-0.24426900
O	-4.29326000	-1.14582300	-0.33640000
O	-3.38830800	0.93134200	0.06890400
C	-6.56195400	-0.89853500	0.49238800
H	-7.39896100	-0.20545000	0.62939000
H	-6.92432700	-1.76014600	-0.07733300
H	-6.23407200	-1.24887300	1.47350500
C	-5.85156600	0.05994600	-1.71488000
H	-6.09930700	-0.88744200	-2.20344800
H	-6.73372500	0.70660200	-1.75040700
H	-5.04670800	0.53814000	-2.28229700
C	-5.33662800	2.37700300	-0.01798900
H	-6.41120400	2.45149500	0.18071900
H	-4.83382200	3.18175900	0.52771300
H	-5.16757100	2.52824900	-1.08639000
C	-4.84845300	0.93822500	1.97882700
H	-4.23198300	1.73748900	2.40136100
H	-5.87063600	1.05804400	2.35081100
H	-4.45754700	-0.01967100	2.33617300
C	4.94511500	0.24745300	-0.13931200
C	4.44982400	1.37996600	-0.81430800
C	6.13631300	0.35613200	0.60794800
C	5.13038800	2.59339400	-0.74069500
H	3.53710700	1.32369800	-1.39572800
C	6.81363000	1.56806400	0.67450900
H	6.51450500	-0.51583500	1.12929500
C	6.31155500	2.68916100	0.00096100
H	4.74068200	3.46192700	-1.26232100
H	7.73086200	1.64425200	1.25032000
H	6.84160500	3.63566000	0.05497900

II

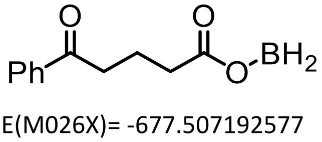

E(M026X)= -677.507192577

E(B3LYP)= -677.844440194

G_{Corr}= 0.177245

0 1

C	1.11035900	0.72830100	-0.00233800
C	-0.06426100	-0.23591400	-0.03753300
C	-1.42450800	0.45874100	-0.04965600
H	0.04588000	-0.88022900	-0.92099000
H	0.01618100	-0.90768100	0.82860800
C	-2.56118600	-0.56120000	-0.08672800
H	-1.52493500	1.09388700	0.83622200
H	-1.49458300	1.12332700	-0.91667500
H	-2.49239400	-1.20870400	-0.97149100
H	-2.52027400	-1.24143100	0.77519800
C	2.49765800	0.16783500	0.00991900
C	2.74815300	-1.21454700	-0.00791400
C	3.58418200	1.05878700	0.04079900
C	4.05903600	-1.69428400	0.00493600
H	1.92807400	-1.92392900	-0.03171600
C	4.89213200	0.57987100	0.05351300
H	3.38470700	2.12514500	0.05444200
C	5.13232900	-0.79930400	0.03567000
H	4.24161200	-2.76488600	-0.00894400
H	5.72455400	1.27737600	0.07734600
H	6.15202100	-1.17413500	0.04565800
O	0.92702500	1.94301500	0.01583100
C	-3.92993600	0.06208500	-0.09828100
O	-4.91994400	-0.90709800	-0.15781900
B	-6.17784800	-0.73161800	0.36070300
O	-4.18857800	1.24121100	-0.09507800
H	-6.43258600	0.23054000	1.01596400
H	-6.95079800	-1.61043900	0.13506700

III

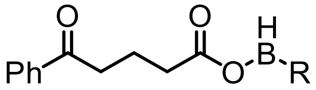

E(M062X)= -1328.41710391

E(B3LYP)= -1329.06102363

G_{Corr}= 0.346091

0 1

C	7.36729800	-0.28273500	0.47455300
C	5.98158100	0.17432000	0.04855500
C	4.86096100	-0.74773000	0.52481500
H	5.82582500	1.19485600	0.42563200
H	5.96882300	0.26043200	-1.04694300
C	3.49629100	-0.23271000	0.07114100
H	5.02300300	-1.75901400	0.13852300
H	4.88298500	-0.82633200	1.61649200
H	3.30095600	0.77910700	0.45105300
H	3.44197700	-0.14861100	-1.02320800
C	8.55054900	0.53246900	0.05712000
C	8.42111800	1.70867100	-0.70029500
C	9.83326800	0.10259800	0.43798300
C	9.55243100	2.43910700	-1.06816100
H	7.44306100	2.06276800	-1.00731100
C	10.96207200	0.83120300	0.07019800
H	9.92679800	-0.80670800	1.02244100
C	10.82358500	2.00241700	-0.68429200
H	9.44110000	3.34726700	-1.65347500
H	11.94893100	0.48964800	0.36933500
H	11.70325900	2.57163400	-0.97160400
O	7.51554900	-1.30017600	1.14704700
C	2.34899900	-1.10327900	0.50716300
O	1.14048300	-0.55863000	0.12697600
B	-0.00063900	-1.31080200	-0.03197400
O	2.42370700	-2.13447900	1.13328200
H	-0.00039900	-2.49415200	-0.04979500
O	-1.14206600	-0.55470700	-0.16812100
C	-2.35088800	-1.08863900	-0.56246200
C	-3.49800100	-0.23230400	-0.09868400

O	-2.42593200	-2.10075900	-1.21889200
C	-4.86329800	-0.73592900	-0.56310000
H	-3.30497300	0.79023300	-0.45001100
H	-3.44073000	-0.17950800	0.99747400
C	-5.98387300	0.16933600	-0.05555900
H	-5.02229900	-1.75863400	-0.20674200
H	-4.88882600	-0.78183200	-1.65655200
C	-7.37015700	-0.27660400	-0.49136100
H	-5.83096500	1.20121400	-0.40175400
H	-5.96785600	0.22186800	1.04202400
C	-8.55350500	0.52221900	-0.04366400
O	-7.51873800	-1.27189200	-1.19616200
C	-8.42366800	1.67330500	0.75133000
C	-9.83673400	0.10248700	-0.43400900
C	-9.55506200	2.38899000	1.14685400
H	-7.44520700	2.01919600	1.06628100
C	-10.96562000	0.81641300	-0.03874000
H	-9.93061500	-0.78739200	-1.04757900
C	-10.82672300	1.96250700	0.75331400
H	-9.44340000	3.27768900	1.76125400
H	-11.95287400	0.48287500	-0.34552300
H	-11.70647200	2.52017200	1.06224100

IV

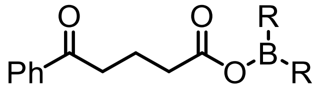

E(M026X)= -1979.31849839

E(B3LYP)= -1980.26918922

G_{Corr}= 0.515949

0 1

C	-2.93161800	5.21250700	0.51710900
C	-1.74962400	4.37989600	0.04850500
C	-1.73772300	2.96186600	0.61523600
H	-1.76116200	4.35411400	-1.05014400
H	-0.82615900	4.90966700	0.32104400
C	-0.52325900	2.18310100	0.11359500
H	-1.72857100	2.99891500	1.70926900
H	-2.65635600	2.43953000	0.32998300
H	-0.51236700	2.11831800	-0.98321500
H	0.41721700	2.67892900	0.38877300
C	-3.05295200	6.62186500	0.02924800
C	-2.10798200	7.19974000	-0.83505000
C	-4.15106600	7.38988400	0.45289900
C	-2.25944800	8.51890000	-1.26573900
H	-1.25107100	6.62949600	-1.17670500
C	-4.30228600	8.70608300	0.02267700
H	-4.87752500	6.93820300	1.12027100
C	-3.35552000	9.27381600	-0.83844300
H	-1.52275900	8.95594500	-1.93358800
H	-5.15499600	9.29082200	0.35585000
H	-3.47226800	10.30037200	-1.17451600
O	-3.77147900	4.74065900	1.27964900
C	-0.45844700	0.77557600	0.63295300
O	0.67067900	0.13196200	0.19150800
B	0.84702800	-1.23083800	0.13314300
O	-1.25397100	0.23550400	1.36782000
O	2.12011800	-1.75098000	0.16707800
C	3.20383600	-1.14716300	0.76748700
C	4.48685900	-1.52412600	0.07867600
O	3.08546300	-0.44485800	1.74320900
C	5.72986400	-0.92239600	0.73150600
H	4.39414000	-1.21300800	-0.97103900
H	4.53477300	-2.62119100	0.05468500
C	6.99629600	-1.34071000	-0.01272600
H	5.79075200	-1.24097600	1.77708300
H	5.65006500	0.16924500	0.74480500
C	8.27040100	-0.76481400	0.58345900
H	6.94171200	-1.03455500	-1.06691600
H	7.08784900	-2.43568600	-0.02885300
C	9.57897100	-1.10744900	-0.05606400
O	8.22917200	-0.03222200	1.56891700
C	9.66753100	-1.94816600	-1.17827500
C	10.75705400	-0.56790000	0.48822500
C	10.90968400	-2.24241400	-1.74318300
H	8.77414500	-2.37780500	-1.61818100
C	11.99641500	-0.86142600	-0.07577500
H	10.68239700	0.08078200	1.35468400
C	12.07518800	-1.70048600	-1.19378500
H	10.96691000	-2.89372100	-2.61050800
H	12.90075500	-0.43888100	0.35274600
H	13.04128600	-1.93032400	-1.63447500
O	-0.15605500	-2.15676300	-0.03131400
C	-1.37430000	-1.90381500	-0.61863900
C	-2.44887500	-2.79582800	-0.06788500
O	-1.49656900	-1.07173300	-1.48810100
C	-3.80091300	-2.61074900	-0.75392300
H	-2.51141600	-2.58296800	1.00826400
H	-2.09295400	-3.83170300	-0.14452500
C	-4.85916800	-3.52497900	-0.14055000
H	-3.70518800	-2.81963400	-1.82429400
H	-4.11803700	-1.56662900	-0.66893300
C	-6.22545900	-3.39378000	-0.79360300
H	-4.96855700	-3.31911200	0.93331800
H	-4.54586400	-4.57624100	-0.20868100
C	-7.35177000	-4.23048900	-0.27388200
O	-6.40397900	-2.61767000	-1.72923800
C	-7.18547900	-5.13605900	0.78733100
C	-8.61755700	-4.10241600	-0.87103000
C	-8.26412400	-5.89742500	1.24073400
H	-6.21934500	-5.25439900	1.26572400
C	-9.69384000	-4.86183800	-0.41813600
H	-8.73972200	-3.40116600	-1.68987100
C	-9.51891100	-5.76199900	0.63983800
H	-8.12471300	-6.59472800	2.06175000
H	-10.66813500	-4.75459400	-0.88639900
H	-10.35768200	-6.35484600	0.99393400

Products

$P_{1a\text{-}OBpin\text{-}E}$

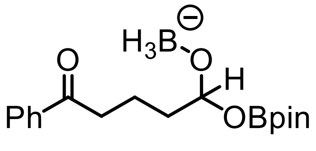

E= -1090.09600378

G_{Corr}= 0.36624

-1 1

C	3.85692500	-0.27020200	0.56309900
C	2.86870000	0.39365200	-0.37822000
C	1.43478800	0.45332400	0.14418100
H	3.23562200	1.40685200	-0.59771400
H	2.90524100	-0.14150700	-1.33869900
C	0.50225000	1.13409700	-0.85978500

H	1.07818700	-0.55976700	0.35884900
H	1.40960600	1.00209600	1.09098800
H	0.86728700	2.14579300	-1.07686500
H	0.49403000	0.58250600	-1.81054700
C	5.29551500	-0.35999800	0.14654500
C	5.75302400	0.14091100	-1.08348000
C	6.21427500	-0.96691200	1.01934300
C	7.10110400	0.03602200	-1.43187400
H	5.06490500	0.61396600	-1.77539000
C	7.55966300	-1.07112700	0.67256800
H	5.85522500	-1.35146400	1.96829200
C	8.00647300	-0.56930300	-0.55569000
H	7.44321600	0.42704000	-2.38587800
H	8.26083500	-1.54170100	1.35616000
H	9.05541500	-0.64974900	-0.82759000
O	3.49909400	-0.72987400	1.64516000
C	-0.94146500	1.27649300	-0.37098000
O	-1.06973800	1.92974700	0.83253100
O	-1.47042600	-0.06713400	-0.21650200
C	-4.77094200	-1.41907700	-0.05884200
C	-5.05745600	0.12811600	-0.08708700
B	-2.81001700	-0.25344300	-0.15906900
C	-5.34918300	0.71276700	1.29837400
H	-5.34104400	1.80506600	1.23023600
H	-6.33085700	0.39626800	1.66556300
H	-4.58990600	0.41217900	2.02733100
C	-6.12849200	0.56253300	-1.07988800
H	-7.09047600	0.09956300	-0.83274400
H	-6.25261500	1.64954100	-1.03438500
H	-5.86268500	0.29103900	-2.10422500
C	-4.96020300	-2.09044400	-1.42279500
H	-4.56407500	-3.10991500	-1.37436600
H	-6.01865500	-2.14502300	-1.69716200
H	-4.42383500	-1.55199600	-2.21091400
C	-5.52742700	-2.18785500	1.01759300
H	-6.60878300	-2.09696400	0.86526400
H	-5.26502700	-3.24986100	0.96571100
H	-5.28369800	-1.82354900	2.01825100
O	-3.77379100	0.67003900	-0.51403400
O	-3.34615000	-1.46339200	0.24559700
B	-1.03927200	3.43548400	0.79242100
H	-1.35017600	3.83805400	1.91570900
H	0.08935100	3.86843100	0.49896900
H	-1.84965600	3.85751800	-0.05322700
H	-1.53820200	1.78232000	-1.14876300

$P_{1a\text{-}OBpin\text{-}EE}$

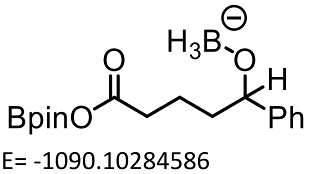

E= -1090.10284586

G_{Corr}= 0.364744

-1 1

C	-0.61988900	-0.72228000	0.07576100
C	0.55947700	-1.20999700	-0.72365700
C	1.91578400	-0.91140300	-0.08887000
H	0.42394200	-2.28811000	-0.88420600
H	0.47918300	-0.75560300	-1.72177400
C	3.06751900	-1.40692600	-0.96402100
H	2.00873400	0.16768600	0.08059100
H	1.97722400	-1.39045700	0.89391100
H	2.95216600	-2.48130500	-1.14837500
H	3.03634100	-0.90710500	-1.94267300

O	-0.57771800	-0.10322200	1.11730500
C	4.46713800	-1.18400400	-0.33506100
O	4.61975200	-1.82812600	0.89666100
B	4.65095100	-3.32517500	0.89021800
H	5.07626000	-3.69023800	1.99311700
H	3.52713900	-3.82941200	0.70688600
H	5.41085400	-3.73927800	-0.01012400
H	5.19774900	-1.59363100	-1.06007500
C	4.74957900	0.30555100	-0.19103300
C	5.01228700	1.08457000	-1.32767600
C	4.72176500	0.93705300	1.05810100
C	5.23405300	2.46010200	-1.22315000
H	5.04698700	0.60791900	-2.30560500
C	4.94243100	2.31416900	1.16947600
H	4.52772000	0.33380300	1.93839100
C	5.19796400	3.08279800	0.02988800
H	5.44014700	3.04449800	-2.11676700
H	4.91460400	2.78694300	2.14855800
H	5.37152900	4.15252000	0.11572200
O	-1.80370600	-1.09325200	-0.50232700
C	-4.60898300	1.02971200	0.33308700
C	-5.28276900	-0.27466700	-0.23917000
O	-3.20754100	0.85365700	-0.06409900
O	-4.16988200	-1.23093400	-0.22267800
B	-3.02258200	-0.48803000	-0.23591200
C	-5.72298100	-0.14025400	-1.69852800
H	-6.00274400	-1.12807500	-2.07765300
H	-6.58964500	0.52176700	-1.78987600
H	-4.91746900	0.25238600	-2.32732600
C	-6.41510000	-0.83417900	0.61099100
H	-7.22961800	-0.10562600	0.68707500
H	-6.81244400	-1.74089100	0.14356700
H	-6.07711700	-1.08708900	1.61832500
C	-4.62429600	1.10082600	1.86155900
H	-3.97971500	1.92387100	2.18490200
H	-5.63531700	1.28560500	2.23766900
H	-4.24823400	0.17639900	2.31101000
C	-5.12271500	2.33094100	-0.26750000
H	-6.19142300	2.45077000	-0.05876100
H	-4.59182900	3.17692100	0.18064100
H	-4.97168400	2.36470000	-1.34874500

V

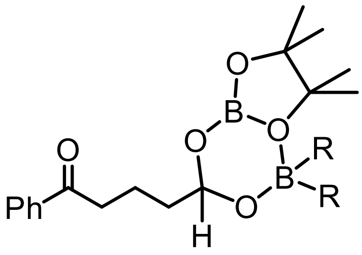

E(M062X)= -2391.21363851

E(B3LYP)= -2392.37377494

G_{Corr}= 0.70586

0 1

C	-0.99870500	-2.05596700	-0.71837000
C	-0.54042700	-3.48034300	-0.97388100
C	0.72840100	-3.86863500	-0.21006500
H	-1.36908000	-4.14893200	-0.70957200
H	-0.38160100	-3.57696800	-2.05549600
C	1.14680700	-5.30389300	-0.52084700
H	1.54104700	-3.18390300	-0.47147600
H	0.56056000	-3.76101000	0.86669200

H	0.33653900	-6.00353800	-0.27044500
H	1.31729400	-5.42755900	-1.59965800
O	-1.35368100	-1.93520700	0.69165100
C	2.40035300	-5.75048800	0.21206200
O	2.98359900	-4.99428700	0.98549900
H	-1.91471500	-1.82522800	-1.27160800
C	2.91536300	-7.13644700	-0.02508700
C	2.27745000	-8.03458700	-0.89698900
C	4.07686700	-7.54886000	0.65003400
C	2.79190900	-9.31817800	-1.08863500
H	1.37962100	-7.74192300	-1.43033800
C	4.59043100	-8.82951300	0.45858800
H	4.56508400	-6.85089800	1.32211200
C	3.94804500	-9.71784500	-0.41221500
H	2.29070700	-10.00455600	-1.76502300
H	5.48907900	-9.13753300	0.98551300
H	4.34752100	-10.71699700	-0.56234200
O	0.01394600	-1.15430300	-1.04521000
B	-0.23448200	0.23039100	-0.87289800
C	-0.96254700	0.77619300	3.01365400
C	0.00865500	1.22482600	1.86673200
B	-1.05541900	-0.81990500	1.37520600
O	-0.51912100	0.33924000	0.75616600
O	-1.21238900	-0.63833200	2.71058900
C	-2.31635500	1.48577000	2.96996200
H	-2.99587300	0.99095600	3.66969400
H	-2.22097100	2.53467500	3.26519900
H	-2.76347400	1.44388200	1.97263000
C	-0.11600900	2.67977900	1.46187000
H	0.13602800	3.30509700	2.32489100
H	0.57584300	2.90984600	0.65163600
H	-1.12397700	2.92883300	1.13045500
C	1.45818000	0.81061500	2.09058300
H	2.02100900	0.94033700	1.16574400
H	1.91042700	1.44078300	2.86164300
H	1.53740200	-0.23397500	2.40586900
C	-0.34538700	0.86366500	4.40210600
H	-0.05888900	1.89604400	4.62773300
H	-1.08130700	0.54422000	5.14596400
H	0.53619500	0.22587300	4.49268200
O	-1.49861300	0.62366600	-1.45254600
C	-1.94561700	1.86507100	-1.68563800
C	-3.42015900	1.86899700	-2.01594800
O	-1.25689500	2.86943300	-1.60995500
C	-4.25373000	1.53212000	-0.76538900
H	-3.61102500	1.12509000	-2.79857800
H	-3.68380600	2.85760300	-2.40181400
C	-5.74969300	1.53603800	-1.06761000
H	-4.03991200	2.26059700	0.02483300
H	-3.95756200	0.55102200	-0.38144300
C	-6.60911000	1.22378400	0.14684700
H	-5.98246400	0.80605000	-1.85527300
H	-6.05794100	2.51192800	-1.46766100
C	-8.09547200	1.18452800	-0.01642000
O	-6.09461700	1.00744400	1.24171900
C	-8.72081500	1.43960300	-1.24839100
C	-8.88904100	0.88247100	1.10353900
C	-10.11182000	1.39339400	-1.35627700
H	-8.13172900	1.67540300	-2.12800200
C	-10.27694800	0.83584400	0.99549800
H	-8.40024200	0.68645100	2.05214800
C	-10.89170500	1.09164800	-0.23612800
H	-10.58539900	1.59256600	-2.31323100
H	-10.88098000	0.60088500	1.86725700
H	-11.97418900	1.05574800	-0.32160300
O	0.92061700	1.05191400	-1.08012200

C	1.33620600	1.39617000	-2.30630100
C	2.42965700	2.44182200	-2.29688300
O	0.86389600	0.93197200	-3.33169400
C	3.32254800	2.45741100	-1.05309700
H	1.92050100	3.41114100	-2.40259600
H	3.01797100	2.30062600	-3.20949900
C	4.43990900	3.48948700	-1.18448800
H	3.75631300	1.46462900	-0.88925700
H	2.72366000	2.68427000	-0.16819200
C	5.32219400	3.58450300	0.04943900
H	4.01939000	4.48575300	-1.38268100
H	5.07639700	3.26118800	-2.05049800
C	6.46343800	4.55220200	0.04327100
O	5.10265400	2.88450000	1.03547400
C	6.74663800	5.36920100	-1.06403200
C	7.27593000	4.64470600	1.18629100
C	7.82094700	6.25985500	-1.02736100
H	6.13513400	5.31784000	-1.95823100
C	8.34800700	5.53331000	1.22268100
H	7.05219800	4.01145700	2.03840300
C	8.62289500	6.34356500	0.11446600
H	8.03117000	6.88696400	-1.88891500
H	8.96977000	5.59657900	2.11119500
H	9.45886600	7.03698100	0.14161600

V’

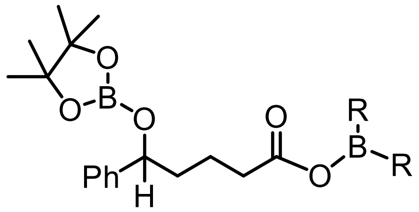

E(M062X)= -2391.219887

E(B3LYP)= -2392.38273916

G_{Corr}= 0.705258

0 1

C	2.73509300	-4.13135100	-0.80851700
C	1.66138600	-3.14862300	-1.32208000
C	0.92839000	-2.39378600	-0.21073600
H	2.14171600	-2.42507300	-1.98950500
H	0.95128300	-3.72367400	-1.92926700
C	-0.17261500	-1.49273200	-0.77387200
H	0.49461300	-3.09469400	0.51199700
H	1.64012500	-1.77571500	0.34368200
H	0.16901300	-0.98464200	-1.68896500
H	-1.06550500	-2.05762500	-1.06189300
C	2.11729000	-5.36901300	-0.18711600
C	1.64509600	-6.38837100	-1.02510500
C	1.95944600	-5.50204100	1.19717000
C	1.01840900	-7.51690500	-0.49230000
H	1.77020500	-6.29995400	-2.10232300
C	1.33378300	-6.63200100	1.73346600
H	2.33325600	-4.72238400	1.85209200
C	0.85929500	-7.64178800	0.89198800
H	0.66117700	-8.29991300	-1.15589200
H	1.21814800	-6.72161900	2.81045400
H	0.37460000	-8.52009100	1.30960600
O	3.58437200	-3.51435100	0.17131800
C	-0.57285900	-0.38196400	0.15460200
O	0.05874500	0.02938500	1.10275200
O	-1.77634400	0.17948800	-0.19375700
H	3.35236700	-4.43614900	-1.66248200
B	-2.11782800	1.48908800	0.05536200
C	5.45852700	-0.50468100	0.40410100
C	5.49214300	-0.75945000	-1.14730900
B	4.34398100	-2.42033700	-0.08620800
O	4.47133900	-1.79767200	-1.30762600
O	5.05684500	-1.81007200	0.92326700
C	4.37181800	0.48913300	0.82078000
H	4.27275100	0.47659300	1.91051600
H	4.64019000	1.50166000	0.51603000
H	3.39980900	0.23691100	0.38527100
C	5.10130300	0.44052100	-1.99929900
H	5.78334400	1.27787000	-1.81984800
H	5.15949400	0.17660900	-3.06021700
H	4.08184900	0.76945200	-1.78707600
C	6.81601000	-1.35342100	-1.63207600
H	6.69947000	-1.68467400	-2.66877000
H	7.61814500	-0.60950000	-1.59702500
H	7.11399600	-2.21561900	-1.02687500
C	6.79482000	-0.11141000	1.01777600
H	7.15002800	0.83074100	0.58794300
H	6.67526800	0.03475500	2.09612200
H	7.55444600	-0.87994200	0.85730300
O	-3.43647600	1.84516900	0.20533800
C	-4.43288400	1.01251000	0.67015100
C	-5.77682200	1.42580100	0.13597000
O	-4.20539100	0.10797800	1.43733400
C	-6.93025700	0.57376800	0.66230100
H	-5.71614200	1.38719100	-0.96044000
H	-5.91391100	2.48702600	0.38379300
C	-8.26394500	1.03864300	0.08119200
H	-6.96201300	0.62720100	1.75523800
H	-6.76079500	-0.47745800	0.40840700
C	-9.45437400	0.22774200	0.56662300
H	-8.24101400	0.99298800	-1.01657300
H	-8.44372600	2.09438800	0.32818600
C	-10.81727000	0.58403500	0.06226800
O	-9.30332700	-0.70094000	1.35667900
C	-11.02986000	1.63639100	-0.84402600
C	-11.91930200	-0.16257400	0.51319700
C	-12.31884800	1.93454400	-1.28932400
H	-10.19716800	2.22828700	-1.20815600
C	-13.20545200	0.13517100	0.06882200
H	-11.74890600	-0.97393800	1.21305400
C	-13.40787500	1.18558300	-0.83441700
H	-12.47201900	2.75010400	-1.99013900
H	-14.05026400	-0.44822500	0.42372500
H	-14.41057500	1.41867400	-1.18179500
O	-1.21971200	2.53398600	0.09622400
C	-0.00913500	2.52912600	-0.55422100
C	1.02893800	3.33814900	0.16948500
O	0.14589200	1.92690700	-1.59284200
C	2.33487300	3.45143800	-0.61420100
H	1.18447400	2.83733600	1.13569100
H	0.60159400	4.32016900	0.40728700
C	3.45976100	4.05633700	0.23116400
H	2.18121600	4.04734500	-1.51797300
H	2.63573000	2.45377300	-0.94872800
C	4.78315200	4.07556600	-0.52195700
H	3.55668900	3.50303000	1.16909700
H	3.22152100	5.09401200	0.50883800
C	6.06588400	3.89467700	0.22415800
O	4.79270800	4.21969000	-1.74262500
C	6.13668900	3.92250300	1.62682700
C	7.24189100	3.67573000	-0.51515500
C	7.35852000	3.73604600	2.27506000
H	5.24683400	4.09674800	2.22157600
C	8.45833100	3.47691500	0.13249000
H	7.18119600	3.65468500	-1.59814300
C	8.51896200	3.50713500	1.53089900
H	7.40235500	3.76332800	3.35985100
H	9.35823500	3.29556100	-0.44810900
H	9.46703500	3.35005100	2.03750000

H_2

H—H

E= -1.16902527348

G_{Corr}= -0.001364

0 1

H	0.00000000	0.00000000	0.37153500
H	0.00000000	0.00000000	-0.37153500

$P_{1a\text{-}OH}$

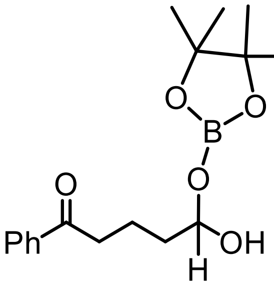

E= -1063.92693035

G_{Corr}=0.351806

0 1

C	0.99306000	-1.61348400	-0.03275800
C	-0.48964700	-1.71365100	-0.34584200
C	-1.33156300	-0.57737600	0.24303600
H	-0.59346000	-1.73653500	-1.43789800
H	-0.84078800	-2.68295000	0.02996800
C	-2.80263100	-0.72571800	-0.14000100
H	-1.23400100	-0.56813600	1.33303000
H	-0.95400900	0.38587200	-0.11477300
H	-2.91358200	-0.74213500	-1.23369300
H	-3.19391200	-1.69264700	0.20767300
O	1.50572400	-0.40525000	-0.62896200
C	-3.69808500	0.37156500	0.40948500
O	-3.23797000	1.27709300	1.10116600
C	4.81518700	0.96425600	-0.68627300
C	4.88983500	-0.04533900	0.52049600
B	2.80961000	-0.07778700	-0.42284100
O	3.68480100	-0.85525400	0.31274900
O	3.37328000	1.06879100	-0.92127900
C	5.43475800	0.41260600	-1.97277500
H	5.18067800	1.07918300	-2.80276900
H	6.52521900	0.35873900	-1.89630900
H	5.05257300	-0.58635700	-2.20652000
C	6.10264500	-0.96519800	0.51212700
H	7.02527300	-0.37827300	0.57773100
H	6.06383900	-1.63404900	1.37803700
H	6.14027500	-1.57595900	-0.39259500
C	4.73523400	0.62838700	1.88570300
H	4.60380100	-0.14355100	2.65057500
H	5.62346600	1.21499200	2.13963600
H	3.86279600	1.28900500	1.91187400
C	5.36040700	2.35398100	-0.38560100
H	6.42205400	2.29834700	-0.12130700
H	5.26429800	2.98655200	-1.27389200
H	4.81986400	2.83075700	0.43511900
C	-5.16121100	0.32994800	0.09184500
C	-5.99089500	1.34437200	0.59926800
C	-5.72910900	-0.68677700	-0.69398000
C	-7.35735500	1.34362700	0.32830000
H	-5.54677600	2.12720400	1.20518600
C	-7.09854800	-0.68772900	-0.96540300
H	-5.11138000	-1.48119100	-1.09824800
C	-7.91459800	0.32616700	-0.45553100
H	-7.98928900	2.13290100	0.72558800
H	-7.52705200	-1.47879700	-1.57403300
H	-8.98019100	0.32437400	-0.66758100
O	1.17772400	-1.61018000	1.36334700
H	2.13734600	-1.64514700	1.52965000
H	1.54153000	-2.45269400	-0.47898100

$P_{1a\text{-}Ph}$

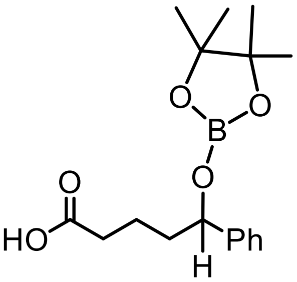

E= -1063.94450266

G_{Corr}= 0.350015

0 1

C	-0.61977700	0.98045600	0.63752500
C	-1.11215100	-0.36496500	1.20042600
C	-1.90064200	-1.20943400	0.19701500
H	-0.23547000	-0.92166200	1.55523100
H	-1.73036400	-0.15401300	2.08280200
C	-2.29726800	-2.55707000	0.79692200
H	-2.80052400	-0.67170900	-0.12055400
H	-1.29830500	-1.37245700	-0.70299500
H	-1.41251500	-3.12254200	1.11892200
H	-2.90801900	-2.42496600	1.70001600
C	-1.76075800	1.87735500	0.20168800
C	-2.50259000	2.55466500	1.17910800
C	-2.12651900	2.01258600	-1.14217000
C	-3.59534000	3.34783500	0.82245800
H	-2.22234100	2.46349900	2.22645700
C	-3.21838800	2.80874000	-1.50218100
H	-1.55275100	1.49543200	-1.90352000
C	-3.95795200	3.47688000	-0.52251800
H	-4.15826300	3.86909200	1.59219800
H	-3.49073600	2.90491000	-2.54998300
H	-4.80577400	4.09585100	-0.80325000
O	0.25215900	0.75312300	-0.48276000
C	-3.07406000	-3.43586200	-0.15200700
O	-3.37781700	-3.16493800	-1.29926000
H	-0.05180400	1.48864300	1.42527400
C	3.63432200	-0.34439500	-0.79590900
C	3.59983300	0.18140800	0.68766800
B	1.55651200	0.43159300	-0.28897000
O	2.16264900	0.29287400	0.94056700
O	2.40340800	0.21505300	-1.35256500
C	3.51530600	-1.86772400	-0.89711000
H	3.35381800	-2.14207500	-1.94430100
H	4.42769900	-2.36160400	-0.54838800
H	2.67057200	-2.24374900	-0.31071800
C	4.20345000	-0.76615100	1.71577200
H	5.26316200	-0.93978300	1.49872100
H	4.12944700	-0.32257800	2.71407800
H	3.68749600	-1.72886000	1.72907600
C	4.18817100	1.58586300	0.84454300
H	3.95755900	1.95784800	1.84780000
H	5.27587200	1.57559600	0.72316100
H	3.76349300	2.28254300	0.11458400
C	4.81463900	0.15208800	-1.62046800
H	5.75871400	-0.18119300	-1.17552000
H	4.75380700	-0.25741000	-2.63405500
H	4.82720100	1.24207600	-1.69134000
O	-3.41704000	-4.61408800	0.42296300
H	-3.91306900	-5.13869200	-0.23496900

$P_{1a\text{-}OBpin\text{-}OH}$

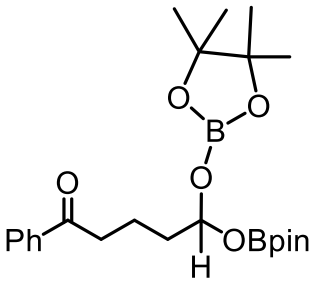

E= -1474.62363318

G_{Corr}= 0.511816

0 1

C	0.16011400	-0.17005200	0.91079800
C	-1.30578200	0.04786200	1.24931800
C	-2.27145100	-0.32126300	0.11983700
H	-1.52693000	-0.53990800	2.14967200
H	-1.42282100	1.10497400	1.52096800
C	-3.72143500	-0.07393800	0.53093600
H	-2.03730900	0.26577100	-0.77388600
H	-2.14042100	-1.37359600	-0.15174100
H	-3.97007400	-0.65677600	1.42925300
H	-3.86410000	0.97823500	0.81641700
O	0.37073000	-1.54758000	0.64033700
C	-4.73567600	-0.41198300	-0.54833700
O	-4.37620400	-0.83744300	-1.64364900
H	0.79981500	0.13231600	1.74493600
C	3.83055700	-2.08215500	-0.18084100
C	3.28363400	-3.53634100	0.06805900
B	1.62550100	-2.01057400	0.36939300
O	1.83504000	-3.32901800	0.04293500
O	2.77288100	-1.25332300	0.40210800
C	3.91100700	-1.71772100	-1.66544100
H	4.10656600	-0.64655500	-1.76166100
H	4.72076100	-2.26162500	-2.16177300
H	2.97303100	-1.94000600	-2.18411400
C	3.65120400	-4.54888800	-1.00838600
H	4.73946700	-4.65583900	-1.07590200
H	3.22890200	-5.52643200	-0.75400600
H	3.26747700	-4.25391500	-1.98758700
C	3.63138600	-4.08099100	1.45606100
H	3.06266000	-4.99986400	1.62972400
H	4.69747500	-4.31634300	1.53405000
H	3.37502400	-3.36509500	2.24375600
C	5.14334800	-1.76228200	0.52053600
H	5.93552100	-2.43300700	0.17026100
H	5.44426800	-0.73478600	0.29218100
H	5.05554300	-1.86049400	1.60491100
C	-6.19075000	-0.21723200	-0.25155800
C	-7.13101100	-0.53939500	-1.24505000
C	-6.64459300	0.27467000	0.98368800
C	-8.49420300	-0.37413500	-1.01047100
H	-6.77480000	-0.91911500	-2.19698100
C	-8.01067100	0.44068300	1.21872400
H	-5.94020300	0.53083800	1.76752400
C	-8.93727900	0.11687400	0.22351400
H	-9.21218100	-0.62678200	-1.78557500
H	-8.35044300	0.82195500	2.17739700
H	-10.00018400	0.24614100	0.40782700
O	0.52158200	0.57114200	-0.24820600
C	2.68607200	3.29629200	0.64537100
C	3.00245400	3.06793800	-0.87985400
B	1.44318500	1.57204800	-0.17065400
O	1.97108600	2.06957900	0.99868600
O	1.89677500	2.20749900	-1.30187800
C	3.91119400	3.42124200	1.54133400
H	4.51490800	4.28569700	1.24389400
H	3.59447900	3.56904800	2.57893600
H	4.53567400	2.52621100	1.49777100
C	4.29356600	2.27881300	-1.10986400
H	4.34816200	1.98399900	-2.16247500
H	5.17554100	2.88277700	-0.87490800
H	4.31849200	1.37314400	-0.49708600
C	2.99133000	4.33065100	-1.73091600
H	3.75622100	5.03307200	-1.38193500
H	3.21491100	4.07468700	-2.77172500
H	2.01962000	4.82886500	-1.70159000
C	1.72077500	4.45783000	0.89448600
H	1.39968000	4.43487900	1.94063500
H	2.20301600	5.42164700	0.70374100
H	0.83095400	4.38279600	0.26108600

$P_{1a\text{-}OBpin\text{-}Ph}$

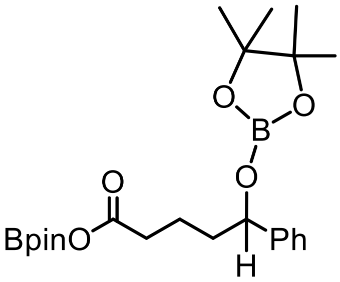

E= -1474.63190779

G_{Corr}= 0.510096

0 1

C	-2.93443700	1.36802700	-0.60088600
C	-1.62196700	0.98617800	-1.30879200
C	-0.38306600	1.07432100	-0.41499600
H	-1.73469900	-0.03379200	-1.69797200
H	-1.50523700	1.64479300	-2.17939100
C	0.87032700	0.61475500	-1.15613700
H	-0.24454800	2.10379100	-0.06710600
H	-0.52680600	0.45780000	0.47872800
H	0.76619800	-0.42069000	-1.50902200
H	1.04341800	1.21545700	-2.05912500
C	-2.92600700	2.79753200	-0.09824000
C	-3.11406500	3.84038300	-1.01554600
C	-2.68862500	3.11026400	1.24486200
C	-3.05897900	5.17298000	-0.60133800
H	-3.30761800	3.60886200	-2.06087400
C	-2.63609100	4.44375900	1.66273700
H	-2.54799000	2.30744000	1.96042600
C	-2.81818300	5.47945100	0.74222600
H	-3.21001400	5.96989800	-1.32466500
H	-2.45114300	4.67182000	2.70921100
H	-2.77772400	6.51530800	1.06798300
O	-3.16785200	0.48277800	0.50769500
C	2.12571900	0.67806100	-0.32451700
O	2.19672400	1.02433500	0.83498200
H	-3.75311900	1.25089200	-1.32023300
C	-4.30023900	-2.89141200	0.77612800
C	-4.84403200	-2.47858200	-0.64253600
B	-3.71667400	-0.74256500	0.30954300
O	-4.09304700	-1.25219600	-0.91402400
O	-3.94924300	-1.59877200	1.36230700
C	-3.01030500	-3.71399300	0.71011700
H	-2.59064800	-3.79669600	1.71758600
H	-3.20169000	-4.72347600	0.33287400
H	-2.26339000	-3.23798900	0.06642900
C	-4.55871100	-3.48083800	-1.75298400
H	-5.03823500	-4.44090500	-1.53261400

H	-4.96440200	-3.10799700	-2.69917800
H	-3.48672200	-3.64696900	-1.88121000
C	-6.32667900	-2.09803500	-0.63392900
H	-6.58193300	-1.64172100	-1.59555400
H	-6.96107300	-2.97856200	-0.49205800
H	-6.55098300	-1.37634200	0.15814300
C	-5.32105600	-3.57638300	1.67508100
H	-5.66752500	-4.50932500	1.21698600
H	-4.85888600	-3.82150800	2.63688200
H	-6.18641900	-2.93697600	1.86373800
O	3.22256300	0.32417100	-1.05569700
C	6.04033300	-0.83764100	0.89388400
C	6.65496300	-0.56120400	-0.53032100
B	4.44257000	-0.05679500	-0.51217500
O	5.60169600	0.23591100	-1.17258700
O	4.59756400	-0.79969100	0.62094800
C	6.82240300	-1.82620100	-1.37449300
H	7.07162300	-1.53884000	-2.40065500
H	7.63044400	-2.45438300	-0.98706000
H	5.90226700	-2.41880900	-1.39816200
C	7.94160000	0.25245500	-0.52489700
H	8.72857600	-0.28331600	0.01676800
H	8.28260800	0.40732900	-1.55354400
H	7.80007900	1.23021500	-0.05916900
C	6.32234300	0.27649800	1.90386900
H	5.71221200	0.10942100	2.79673400
H	7.37542100	0.28098600	2.20151400
H	6.07072300	1.26120600	1.49753100
C	6.38399200	-2.19671600	1.48713100
H	7.46617900	-2.28632900	1.63083900
H	5.90173300	-2.30310000	2.46405000
H	6.04717400	-3.01494700	0.84679500

Transition States

$TS_{1a\text{-}OH}$

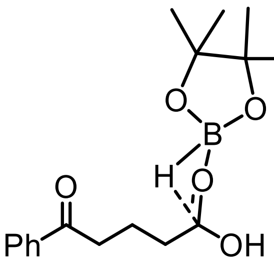

E= -1063.83062813

G_{Corr}= 0.346476

0 1

C	1.12122500	-1.55311500	-0.61290800
C	-0.09845800	-0.75740800	-0.99972500
C	-1.30052000	-1.19286000	-0.13358900
H	0.12739900	0.29788000	-0.82976600
H	-0.31564300	-0.89362600	-2.06497500
C	-2.52062700	-0.32883100	-0.44586200
H	-1.53831300	-2.24643900	-0.31173100
H	-1.04094700	-1.09865200	0.92535700
H	-2.29367200	0.72987500	-0.26303300
H	-2.77956500	-0.39943400	-1.51126500
O	1.77385400	-1.29536700	0.48940700
C	-3.74598800	-0.70901300	0.37158400
O	-3.70224000	-1.64264400	1.16871700
H	2.27520100	-0.61581600	-1.47013100
C	4.95626500	0.09317600	0.34882300
C	4.13826600	1.40572300	0.06812400
B	2.79961200	-0.42854700	-0.28361000
O	2.77256400	0.92964800	0.13672900
O	4.13413500	-0.92403600	-0.27690800
C	5.05535800	-0.23296800	1.84491200
H	5.44883300	-1.24850400	1.95951100
H	5.72967700	0.45674400	2.36428100
H	4.07336900	-0.18978400	2.32613200
C	4.32419100	2.50580200	1.10819300
H	5.37399400	2.81729500	1.15689000
H	3.72365300	3.38015200	0.83394800
H	4.01148400	2.17445700	2.10133400
C	4.38220800	1.96841600	-1.33841700
H	3.64217600	2.75038000	-1.53956000
H	5.38030600	2.41018200	-1.42810800
H	4.27679700	1.19154700	-2.10245100
C	6.34344800	0.06129400	-0.28447900
H	6.96276200	0.88038700	0.09944700
H	6.84122700	-0.88319600	-0.03857500
H	6.28907600	0.14494400	-1.37273300
C	-5.00353900	0.07660300	0.18255000
C	-6.13444500	-0.27060300	0.94152900
C	-5.09046700	1.14605800	-0.72454900
C	-7.32633500	0.43559400	0.79803800
H	-6.06075300	-1.09729500	1.64028200
C	-6.28529500	1.85325300	-0.86818700
H	-4.23302800	1.43481100	-1.32266200
C	-7.40399300	1.50014600	-0.10803400
H	-8.19442900	0.15975100	1.38984600
H	-6.34214900	2.67854800	-1.57194300
H	-8.33308600	2.05192800	-0.22042700
O	1.25052300	-2.79203800	-1.06549000
H	0.67912800	-2.95549600	-1.83928500

$TS_{1a\text{-}Ph}$

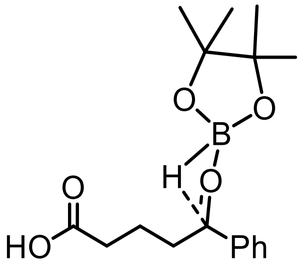

E= --1063.83604639

G_{Corr}= 0.346476

0 1

C	0.55538600	0.44644600	-0.08590500
C	1.47502600	-0.54381800	-0.74787400
C	2.73115600	-0.74522300	0.13021500
H	0.93531300	-1.48934100	-0.84120100
H	1.76142200	-0.21395600	-1.74988100
C	3.62762200	-1.82803900	-0.46820200
H	3.28894800	0.19242300	0.21509100
H	2.42867900	-1.03177000	1.14259200
H	3.09493800	-2.78427900	-0.54463700
H	3.93013800	-1.56979800	-1.49137200
C	0.82413600	1.88703500	-0.13110900
C	1.77326600	2.43782100	-1.01520000
C	0.11946500	2.73482900	0.74645100
C	2.01612300	3.80810000	-1.01277500
H	2.31954700	1.80433000	-1.70451000
C	0.36765800	4.10354800	0.74583300

H	-0.61642900	2.30167700	1.41325500
C	1.31501000	4.64148700	-0.13329000
H	2.74920500	4.22786100	-1.69455500
H	-0.17572200	4.75301900	1.42527500
H	1.50663200	5.71068100	-0.13464300
O	-0.25900900	-0.01427000	0.84465500
C	4.88414000	-2.06292200	0.33486600
O	5.20797500	-1.46966900	1.34672600
H	-0.83027400	0.22700100	-1.23259200
C	-3.66401200	-0.29904500	0.38720800
C	-3.24674000	-1.59143300	-0.40336100
B	-1.42062700	-0.20961900	-0.16750500
O	-1.80864800	-1.58267700	-0.25745800
O	-2.56670700	0.60227100	0.10842000
C	-3.71997300	-0.52808300	1.90398600
H	-3.81055600	0.44198700	2.40430000
H	-4.58175100	-1.14195200	2.18862500
H	-2.80919600	-1.01586600	2.26467000
C	-3.78845800	-2.89450900	0.17669800
H	-4.88469900	-2.89022500	0.17919300
H	-3.45440500	-3.73985800	-0.43514300
H	-3.43752400	-3.05345700	1.19928500
C	-3.58402300	-1.50543900	-1.89829800
H	-3.09104500	-2.33295300	-2.41972400
H	-4.66227600	-1.58340500	-2.07424700
H	-3.22838000	-0.56576900	-2.33281200
C	-4.96222600	0.34585100	-0.08869600
H	-5.80293100	-0.34957800	0.01914900
H	-5.17806500	1.23386500	0.51585300
H	-4.89585400	0.65393700	-1.13516000
O	5.64676300	-3.03911000	-0.20850600
H	6.44309100	-3.14847500	0.34674500

$TS_{1a\text{-}OBpin\text{-}OH}$

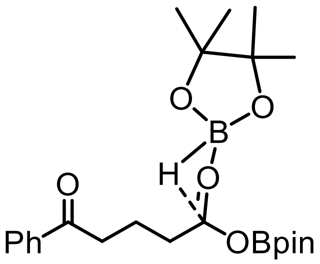

E= -1474.52041083

G_{Corr}= 0.505202

0 1

C	1.21425500	-0.10118800	-0.65471700
C	0.05341500	-0.70300600	0.09293900
C	-1.18841400	-0.77239600	-0.82126100
H	0.35637000	-1.70258100	0.41180400
H	-0.16590500	-0.10046500	0.97606000
C	-2.37430100	-1.35404700	-0.05566700
H	-1.45037400	0.22604600	-1.18551100
H	-0.97360300	-1.38217600	-1.70456100
H	-2.15436100	-2.37804400	0.27381200
H	-2.55305400	-0.77636300	0.86156200
O	2.03370200	-0.85034800	-1.33405400
C	-3.66017800	-1.36749800	-0.86743000
O	-3.68250200	-0.92523500	-2.01330200
H	2.34899500	-0.12035700	0.66548900
C	5.30531000	-1.28307300	-0.28978200
C	4.63756500	-2.29314200	0.71187700
B	3.04131300	-0.87367700	-0.13554600
O	3.23872100	-2.19571600	0.35150600
O	4.29297100	-0.25225100	-0.40879100
C	5.53105900	-1.89166700	-1.68021400
H	5.79974400	-1.09070000	-2.37701100
H	6.34425200	-2.62572400	-1.67168100
H	4.62463600	-2.37921100	-2.05211300
C	5.08449400	-3.74206400	0.54659600
H	6.16425100	-3.83392800	0.71162400
H	4.57428500	-4.37343000	1.28241500
H	4.84984200	-4.12186100	-0.45070400
C	4.78786300	-1.86250100	2.17696600
H	4.13568600	-2.48654200	2.79722200
H	5.81733500	-1.98582900	2.52979300
H	4.49599600	-0.81669800	2.31652700
C	6.59542700	-0.64546600	0.21569700
H	7.35469400	-1.41160200	0.41161000
H	6.99232700	0.03846200	-0.54262000
H	6.42780300	-0.07668100	1.13364900
C	-4.89646800	-1.92343000	-0.23775700
C	-6.08468300	-1.94241900	-0.98821900
C	-4.90851700	-2.42274300	1.07548100
C	-7.25996700	-2.44877000	-0.43843800
H	-6.06847900	-1.55589100	-2.00184800
C	-6.08678300	-2.92945300	1.62619100
H	-4.00529600	-2.42027600	1.67586900
C	-7.26323100	-2.94347000	0.87137200
H	-8.17296700	-2.45940700	-1.02686000
H	-6.08572800	-3.31280700	2.64238600
H	-8.17952100	-3.33849800	1.30115200
O	1.15443600	1.16784200	-1.04027200
C	-0.87323700	3.38963400	0.97158000
C	-0.40784500	4.29696200	-0.23032400
B	0.43419800	2.21066300	-0.44631900
O	0.03199300	2.22981000	0.85102800
O	0.11991500	3.30403700	-1.18453300
C	-0.68414900	4.00466100	2.34961400
H	-1.28244300	4.91745600	2.44063100
H	-1.02046100	3.29962200	3.11621100
H	0.36191500	4.25115400	2.54294500
C	0.75826700	5.22112000	0.12096800
H	1.14202600	5.67431100	-0.79794700
H	0.43409400	6.02299100	0.79110200
H	1.57608300	4.67488900	0.60155700
C	-1.52578000	5.07177300	-0.91186200
H	-1.99502800	5.76088100	-0.20157000
H	-1.11415100	5.66159100	-1.73665500
H	-2.29325100	4.40680600	-1.31359100
C	-2.29257800	2.84281100	0.81156300
H	-2.46552400	2.07435800	1.57095800
H	-3.03484200	3.63490000	0.94774300
H	-2.44594000	2.39180400	-0.17380400

$TS_{1a\text{-}OBpin\text{-}Ph}$

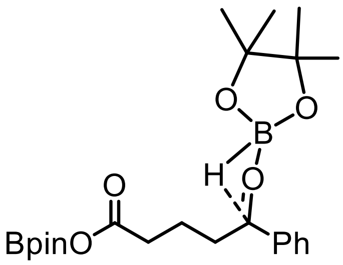

E= -1474.52340372

G_{Corr}= 0.50544

0 1

C	-2.38765000	0.85926300	-0.16180500
C	-1.20215600	0.39703200	-0.96500000
C	0.09654000	0.71364400	-0.18860900
H	-1.29420800	-0.68263000	-1.10606600
H	-1.17965700	0.86978800	-1.95029800
C	1.31062400	0.16109900	-0.93247400
H	0.20432200	1.79474200	-0.05678900
H	0.04275900	0.27224100	0.81178800
H	1.23814900	-0.92719300	-1.05725500
H	1.37826200	0.57363200	-1.94750000
C	-2.75790200	2.27634800	-0.09517100
C	-2.22903800	3.22449000	-0.99364700
C	-3.65594800	2.69530100	0.90650500
C	-2.58715600	4.56475500	-0.88441100
H	-1.54411000	2.92113500	-1.77697000
C	-4.00844200	4.03680800	1.01255500
H	-4.06455500	1.95605400	1.58503900
C	-3.47560800	4.97221600	0.11774900
H	-2.17652700	5.29180000	-1.57811600
H	-4.69842900	4.35608900	1.78760000
H	-3.75319600	6.01916500	0.19996700
O	-2.83972300	0.04464400	0.77270700
C	2.61507800	0.45356500	-0.23377300
O	2.73917900	1.05308900	0.81251000
H	-3.65325300	0.12792700	-1.23517400
C	-5.84747600	-1.62173900	0.51041200
C	-5.00650500	-2.57729100	-0.41130400
B	-3.90482500	-0.56973300	-0.17540500
O	-3.69470300	-1.97244600	-0.35580900
O	-5.25337100	-0.33027500	0.24119200
C	-5.66438600	-1.93030600	2.00294700
H	-6.10266300	-1.11489200	2.58827300
H	-6.16416000	-2.86294300	2.28748000
H	-4.60458200	-2.00817400	2.26421100
C	-4.90315600	-4.01495700	0.08903300
H	-5.89713000	-4.47191700	0.15978800
H	-4.30623100	-4.60945800	-0.61154100
H	-4.42587300	-4.06352000	1.07083200
C	-5.48626700	-2.56529400	-1.86928600
H	-4.74698100	-3.08375400	-2.48926800
H	-6.44769000	-3.07774500	-1.98215000
H	-5.59189700	-1.54242200	-2.24456700
C	-7.33385700	-1.55839200	0.17207100
H	-7.79674700	-2.54737900	0.27037800
H	-7.84231000	-0.87657500	0.86291900
H	-7.49694300	-1.19599500	-0.84596600
O	3.67404500	-0.01143700	-0.95094600
C	6.67258000	-0.61109600	0.98354500
C	7.18671600	-0.57509400	-0.50561700
B	4.94962600	-0.20805600	-0.43218800
O	6.04485000	0.02040900	-1.21350400
O	5.21724100	-0.70975600	0.80649500
C	7.39781100	-1.96485700	-1.10934200
H	7.56939100	-1.86269800	-2.18533700
H	8.26898100	-2.45789400	-0.66722000
H	6.52291600	-2.60594200	-0.96111600
C	8.40997300	0.30066800	-0.73787800
H	9.25961800	-0.07071400	-0.15478700
H	8.68646800	0.27272200	-1.79668000
H	8.22234000	1.33986200	-0.45902500
C	6.93132200	0.68901400	1.74732600
H	6.38577900	0.65854400	2.69538300
H	7.99659300	0.81145300	1.96583000
H	6.58829600	1.56235900	1.18368000
C	7.14311400	-1.81047300	1.79398700
H	8.23545400	-1.80912300	1.87499000
H	6.72735100	-1.75624500	2.80512300
H	6.82704200	-2.75338600	1.34245700

$TS_{1a\text{-}OBpin\text{-}E}$

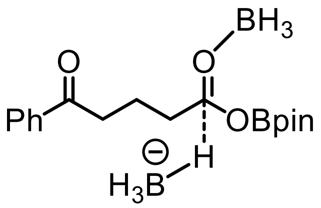

E= -1116.68775633

G_{Corr}= 0.389702

-1 1

C	-3.95856000	-0.53644300	-0.50502000
C	-2.96167200	0.39940900	0.15630300
C	-1.53340700	0.24312000	-0.35987700
H	-3.30713500	1.43210500	0.00907600
H	-2.99744700	0.22854500	1.24147900
C	-0.58699800	1.20548400	0.38359400
H	-1.19808600	-0.78963100	-0.21980800
H	-1.50454100	0.44950000	-1.43399300
H	-0.91865300	2.23886200	0.26628000
H	-0.55683500	0.97061500	1.44982500
C	-5.39387200	-0.46674300	-0.08288600
C	-5.84413800	0.43515600	0.89552600
C	-6.31797000	-1.33534500	-0.68812200
C	-7.19141800	0.46623200	1.26017800
H	-5.15189200	1.11644300	1.37785100
C	-7.66232900	-1.30423800	-0.32437400
H	-5.96419900	-2.02951000	-1.44320200
C	-8.10221600	-0.40220300	0.65188300
H	-7.52860700	1.16777300	2.01778100
H	-8.36814200	-1.98022900	-0.79880500
H	-9.15049500	-0.37689700	0.93653200
O	-3.60132300	-1.33625300	-1.36660000
C	0.80995900	1.09461800	-0.16448700
O	1.16973400	1.60237600	-1.26879300
O	1.44295100	-0.03256000	0.22730200
C	4.73781300	-1.35748600	0.57492900
C	5.00146600	-0.15576500	-0.40832300
B	2.80123000	-0.27181500	0.12100400
C	5.14787600	-0.58684900	-1.86934400
H	5.13417000	0.30403500	-2.50473700
H	6.09359400	-1.11306900	-2.03315900
H	4.32636300	-1.24044200	-2.17961300
C	6.14839600	0.76094200	-0.00484300
H	7.09163500	0.20400100	0.01738100
H	6.24658800	1.56885100	-0.73696000
H	5.98062500	1.20765900	0.97743900
C	5.08276300	-1.03375100	2.03065600
H	4.70381800	-1.83614700	2.67154800
H	6.16547300	-0.96126800	2.17350400
H	4.62249800	-0.09387900	2.35112600
C	5.36986500	-2.67756600	0.15548600
H	6.46047900	-2.58155700	0.11612200
H	5.12329800	-3.45252600	0.88852700
H	5.01204900	-3.00442600	-0.82346300
O	3.74986700	0.60105900	-0.31885500
O	3.27951100	-1.49444200	0.51145900
B	0.50468900	2.86298100	-1.92679100
H	1.18180400	3.08196400	-2.91407500
H	-0.64807100	2.58366300	-2.21257300
H	0.56120800	3.77017200	-1.11262700
B	1.92808000	2.56100900	2.07701400
H	2.82044400	3.36397200	1.83395200

H	2.34287500	1.53992900	2.61310900
H	0.98809900	3.06278200	2.68206800
H	1.46982500	2.19496700	0.94949600

$TS_{1a\text{-}OBpin\text{-}EE}$

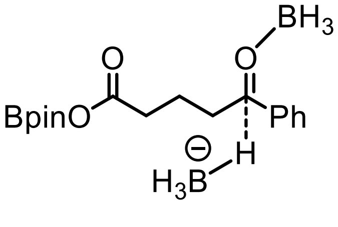

E= -1116.69285967

G_{Corr}= 0.387882

-1 1

C	0.96811300	-0.56107900	-0.26010600
C	-0.24501900	-1.13248700	0.42705100
C	-1.56682200	-0.69636500	-0.20007900
H	-0.14597100	-2.22568900	0.42126300
H	-0.19056600	-0.83723100	1.48397400
C	-2.75528000	-1.28244300	0.58680900
H	-1.62768800	0.39692200	-0.21261100
H	-1.60904800	-1.03358100	-1.24040200
H	-2.67181500	-2.36937400	0.64306000
H	-2.76240400	-0.90022000	1.60863700
O	0.96244500	0.20195800	-1.20182300
C	-4.05552900	-0.94769600	-0.10452300
O	-4.44556400	-1.64629800	-1.10165500
B	-3.98398000	-3.09056200	-1.45367500
H	-4.65288500	-3.39860300	-2.42443600
H	-2.78999000	-3.07428500	-1.71046200
H	-4.22050800	-3.80027100	-0.48717600
B	-5.58865300	-1.69089600	2.32644700
H	-6.72529700	-1.26484600	2.15176400
H	-4.93210500	-0.98240300	3.08159600
H	-5.56639800	-2.87414500	2.64762200
H	-5.01635900	-1.60509600	1.19881400
C	-4.60149200	0.43672500	-0.04133800
C	-4.19354200	1.34133000	0.95133900
C	-5.52952400	0.85676400	-1.00776400
C	-4.70047100	2.64035200	0.97385500
H	-3.49331800	1.03301100	1.71848800
C	-6.03769900	2.15476900	-0.98277300
H	-5.84315100	0.15821500	-1.77521300
C	-5.62410800	3.05160700	0.00794000
H	-4.37730800	3.32966500	1.74874100
H	-6.75468400	2.46772300	-1.73669000
H	-6.01926500	4.06358500	0.02765300
O	2.12113200	-1.04028300	0.29137600
C	5.02568500	1.05649500	-0.20941500
C	5.63023200	-0.31853400	0.26622900
O	3.60085300	0.89300200	0.10420300
O	4.49056400	-1.22977600	0.10517000
B	3.36869000	-0.45091800	0.13908200
C	5.99933500	-0.33812900	1.75106200
H	6.23027100	-1.36630900	2.04640900
H	6.87935200	0.28223200	1.94681900
H	5.17488500	0.02060400	2.37554100
C	6.78673700	-0.83412000	-0.57885700
H	7.62604700	-0.13105600	-0.54349100
H	7.13133600	-1.79503100	-0.18348900
H	6.49378600	-0.97683000	-1.62129900
C	5.11795300	1.26985200	-1.72177900
H	4.51444000	2.14082300	-1.99490100
H	6.15124600	1.45496500	-2.03122900
H	4.73808900	0.40468900	-2.27427400
C	5.54844700	2.27689600	0.53530900
H	6.62860800	2.37987000	0.38479800
H	5.06390100	3.17885400	0.14801300
H	5.34898300	2.21324300	1.60728600

$TS^0{}_1$

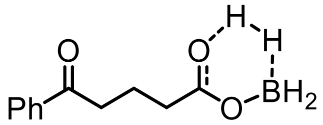

E(M026X)= -678.660170528

E(B3LYP)= -679.011570153

G_{Corr}= 0.193022

0 1

C	-1.20978100	0.70156500	0.01273100
C	-0.05880600	-0.29133600	-0.00835300
C	1.31677500	0.37233900	-0.00324600
H	-0.16589800	-0.96079000	0.85657300
H	-0.17267200	-0.93263600	-0.89346200
C	2.42892400	-0.67662100	-0.01866000
H	1.41386600	1.03118900	-0.87152700
H	1.41764500	1.00772900	0.88236000
H	2.35629000	-1.35428400	0.84140500
H	2.35619600	-1.31859400	-0.90755900
C	-2.61027100	0.17583300	0.00556900
C	-2.89478500	-1.19989100	-0.01701600
C	-3.67448500	1.09370400	0.02190700
C	-4.21727300	-1.64672000	-0.02282400
H	-2.09245600	-1.92959500	-0.02993400
C	-4.99397700	0.64756500	0.01590500
H	-3.44860700	2.15473800	0.03919900
C	-5.26820000	-0.72519900	-0.00643700
H	-4.42631700	-2.71241700	-0.04016000
H	-5.80897900	1.36562800	0.02855300
H	-6.29699600	-1.07439100	-0.01110800
O	-0.99599000	1.91124800	0.03523900
C	3.81574000	-0.10484100	-0.01546500
O	4.79495400	-0.96260800	0.10279900
B	6.20184700	-0.47764100	-0.00851300
O	4.05726000	1.12058600	-0.11298700
H	5.42979600	1.06089800	0.10259800
H	6.28273400	0.83893000	0.33625500
H	6.60376000	-0.49296900	-1.13975400
H	6.88944600	-0.92741100	0.86404400

$TS^{0'}{}_1$

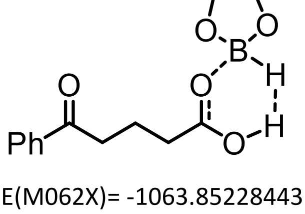

E(M062X)= -1063.85228443

E(B3LYP)= -1064.38934397

G_{Corr}= 0.341376

0 1

C	-4.29389900	-0.74097900	0.08436300
C	-3.02904000	0.09006500	-0.06001000
C	-1.74717600	-0.73554200	0.02856900
H	-3.07577000	0.62550800	-1.01841500
H	-3.03863600	0.86937600	0.71471200
C	-0.51498500	0.15609900	-0.12538600
H	-1.70992200	-1.25951400	0.98868900
H	-1.74890400	-1.50697100	-0.74766000
H	-0.51872300	0.68497700	-1.08819700
H	-0.48710100	0.94172200	0.64148300
C	-5.61766700	-0.04794100	0.01639900
C	-5.72858700	1.33827800	-0.18395000
C	-6.78746600	-0.81391900	0.15839700
C	-6.98465500	1.94458500	-0.24090700
H	-4.84200600	1.95270100	-0.29674200
C	-8.04069000	-0.20859000	0.10178700
H	-6.69541400	-1.88384400	0.31310900
C	-8.14175600	1.17343200	-0.09830100
H	-7.05916600	3.01695300	-0.39638700
H	-8.93856800	-0.80976000	0.21304000
H	-9.11877200	1.64653900	-0.14272600
O	-4.23145800	-1.95617200	0.25409200
C	0.79221100	-0.57123100	-0.04154000
O	1.86719100	0.14824100	-0.14205500
C	5.15354900	0.60488300	0.74218100
C	5.38644200	0.04312700	-0.70751500
B	3.25295700	-0.43999000	-0.02943500
C	6.10341600	-1.31259900	-0.71480800
H	6.05193600	-1.73478200	-1.72379100
H	7.15809200	-1.20787100	-0.44011900
H	5.63469000	-2.01963700	-0.02318400
C	6.08422200	1.00811200	-1.65938400
H	7.08010300	1.26704000	-1.28220400
H	6.20521100	0.53694400	-2.64076300
H	5.50974300	1.92821900	-1.79003400
C	4.85577100	2.10887700	0.75664000
H	4.50842400	2.39083400	1.75608900
H	5.75013800	2.69727000	0.52570100
H	4.07312400	2.36653300	0.03632200
C	6.26302400	0.28065800	1.73640700
H	7.21413500	0.71430000	1.40679200
H	6.01797600	0.70623700	2.71551700
H	6.39319800	-0.79762900	1.85556500
O	4.02586200	-0.16239000	-1.17302100
O	3.94240200	-0.08839600	1.15086100
O	0.85504700	-1.82251800	0.11732600
H	2.11409200	-1.97002400	0.10559800
H	3.06871400	-1.80918500	0.04951700

TS[1]$_1$

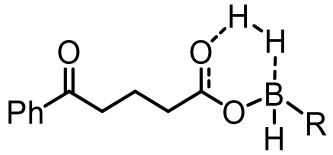

E(M062X)= --1329.55802796

E(B3LYP)= -1330.21606641

G_{Corr}= 0.362318

0 1

C	6.57868800	0.35682100	0.75003300
C	5.53990700	-0.06981800	-0.27489600
C	4.33773200	-0.78322900	0.34043700
H	5.21555000	0.82182600	-0.82905000
H	6.03190100	-0.71550700	-1.01561300
C	3.33211900	-1.18314500	-0.73932400
H	4.67283800	-1.67219600	0.88395700
H	3.85554800	-0.13162700	1.07558000
H	2.96260500	-0.30576000	-1.28804800
H	3.78798700	-1.83285400	-1.49774300
C	7.80342200	1.07104400	0.27371400
C	8.02165200	1.36170400	-1.08348500
C	8.76573500	1.46306800	1.22010700
C	9.17927200	2.03085700	-1.48434800
H	7.29541400	1.07054800	-1.83452600
C	9.92071700	2.13083200	0.81978800
H	8.59179800	1.23587900	2.26666200
C	10.12988700	2.41631800	-0.53480500
H	9.33820100	2.25060900	-2.53608800
H	10.65816600	2.42938700	1.55937000
H	11.03040300	2.93712000	-0.84815600
O	6.41657900	0.11989100	1.94457300
C	2.12505000	-1.90139900	-0.22271500
O	1.28358800	-2.34338300	-1.12684400
B	-0.00275300	-2.93824700	-0.70435200
O	1.90865400	-2.09554700	0.99554900
H	0.80143000	-2.85258100	0.88593400
H	0.09687100	-3.38603000	0.58254000
H	-0.24132100	-3.96251100	-1.27081300
O	-0.99515200	-1.89034300	-0.69648300
C	-2.24174900	-2.13022600	-0.25921700
C	-3.14658700	-0.93272200	-0.44343800
O	-2.57834300	-3.19415400	0.23558300
C	-4.57583700	-1.15982700	0.04548200
H	-3.12937600	-0.66871300	-1.50949000
H	-2.67986000	-0.08538100	0.07693600
C	-5.43464400	0.08529100	-0.16664400
H	-4.56703600	-1.42799700	1.10686000
H	-5.01839800	-2.01018100	-0.48326500
C	-6.87177200	-0.07505800	0.30034000
H	-5.44882600	0.36573600	-1.22938500
H	-4.99763000	0.94669400	0.35791000
C	-7.80869600	1.08240600	0.14687400
O	-7.26081700	-1.13066600	0.79428600
C	-7.40321600	2.30637000	-0.41116000
C	-9.13837200	0.93532000	0.57751700
C	-8.31002400	3.36048500	-0.53516100
H	-6.38308200	2.44646700	-0.75163500
C	-10.04314700	1.98722000	0.45382800
H	-9.44590900	-0.01206900	1.00765200
C	-9.63016900	3.20340500	-0.10338400
H	-7.98591900	4.30244200	-0.96813200
H	-11.06856300	1.86263800	0.79007500
H	-10.33489500	4.02460400	-0.20034600

TS[2]$_1$

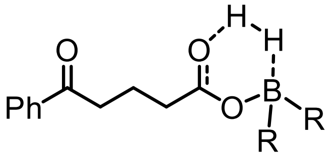

E(M026X)= -1980.45484906

E(B3LYP)= -1981.41950872

G_{Corr}= 0.531089

0 1

C	7.74537200	-2.05002500	-0.54244600
C	6.43359200	-1.75879900	0.16872300
C	5.24478900	-1.63013000	-0.78143800
H	6.25424000	-2.55672600	0.90249400
H	6.55712400	-0.83883200	0.75701000
C	3.95926600	-1.34270500	-0.00628800
H	5.43176400	-0.82934800	-1.50345100
H	5.13188200	-2.55199700	-1.36056600
H	3.74311200	-2.13043200	0.72741300
H	4.03872600	-0.41409300	0.57550100
C	8.98615800	-2.20349500	0.27827700
C	8.97700800	-2.09297100	1.67890400
C	10.20028200	-2.46718900	-0.37877200
C	10.15900000	-2.24357200	2.40605800
H	8.05399600	-1.89031000	2.21113300
C	11.37956800	-2.61719800	0.34705900
H	10.20106100	-2.55153500	-1.46041400
C	11.36109100	-2.50555900	1.74254600
H	10.14085000	-2.15646000	3.48853100
H	12.31258700	-2.82070800	-0.17065700
H	12.28029400	-2.62254500	2.30976200
O	7.78624500	-2.15652300	-1.76572700
C	2.74131000	-1.20458200	-0.86525900
O	1.60976600	-1.01657500	-0.21606900
B	0.36501800	-0.76114200	-0.94098800
O	2.75947100	-1.26515200	-2.11059900
H	1.38107400	-1.20256200	-2.33806200
H	0.48356000	-1.18267600	-2.27029500
O	-0.64772000	-1.61127800	-0.41763100
C	-1.84759800	-1.75652600	-1.02343900
C	-2.79995700	-2.57016700	-0.18210500
O	-2.09417700	-1.28581100	-2.11847800
C	-4.16894000	-2.77221800	-0.82844800
H	-2.88814500	-2.06520400	0.78935600
H	-2.31252700	-3.53132600	0.02984300
C	-5.08749800	-3.59447500	0.07294500
H	-4.05308500	-3.27250500	-1.79524900
H	-4.62755000	-1.80030400	-1.03715600
C	-6.46544100	-3.83744600	-0.52004900
H	-5.21395200	-3.09983800	1.04618200
H	-4.63352800	-4.57021200	0.29650300
C	-7.45843400	-4.62736800	0.27373200
O	-6.76164300	-3.39634900	-1.62793600
C	-7.15871500	-5.14897000	1.54338200
C	-8.73321000	-4.85217000	-0.27359100
C	-8.11532100	-5.88032700	2.24966300
H	-6.18281900	-4.99011600	1.98907300
C	-9.68766400	-5.58189500	0.43130300
H	-8.95893500	-4.44681600	-1.25438100
C	-9.38018300	-6.09799900	1.69592200
H	-7.87307100	-6.27902400	3.23053800
H	-10.66994500	-5.74954600	-0.00118400
H	-10.12383900	-6.66719800	2.24673800
O	0.09514800	0.62631100	-1.17275000
C	-0.25753300	1.45505800	-0.16553100
C	-0.53775300	2.85066300	-0.66916300
O	-0.33464100	1.09437500	0.99453600
C	-0.93611900	3.83113900	0.43202600
H	-1.32269300	2.76971500	-1.43316800
H	0.35828900	3.19385400	-1.20361000
C	-1.21435700	5.21891500	-0.14170400
H	-0.13999900	3.89201300	1.18109700
H	-1.82377800	3.46105900	0.95508400
C	-1.61392300	6.24416700	0.90635900
H	-2.01212700	5.16937000	-0.89614400
H	-0.33075000	5.59884300	-0.67373500
C	-1.91779800	7.64113500	0.46339000
O	-1.68688400	5.93660000	2.09374800
C	-1.84126000	8.03387500	-0.88336500
C	-2.29151600	8.58915100	1.43124100
C	-2.13226200	9.34824200	-1.25290000
H	-1.55551200	7.32230700	-1.65032000
C	-2.58210700	9.90059700	1.06238000
H	-2.34915100	8.27994700	2.46963700
C	-2.50282100	10.28315200	-0.28208000
H	-2.06978500	9.64117500	-2.29696300
H	-2.87003800	10.62533600	1.81862500
H	-2.72908900	11.30578100	-0.57097900

$TS^2_{IV\text{-}V'}$

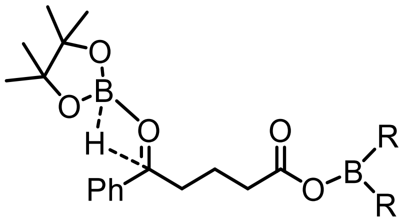

E(M026X)= -2391.10957773
E(B3LYP)= -2392.27417727
G_{Corr}= 0.694771

0 1

C	3.08848300	-3.25939300	0.82653700
C	1.71902800	-2.95547400	0.28099600
C	1.25264300	-1.58246200	0.81548900
H	1.79297800	-2.92057500	-0.80863700
H	0.99966100	-3.73285700	0.55052100
C	-0.09109500	-1.19994600	0.19854900
H	1.16433100	-1.61004800	1.90608000
H	2.00077200	-0.82137700	0.57199300
H	-0.02704700	-1.14789700	-0.89640300
H	-0.86364800	-1.94791200	0.41874700
C	3.27405600	-3.72862700	2.20324900
C	2.19610200	-4.20100800	2.97827300
C	4.56632900	-3.68868600	2.76333800
C	2.40873700	-4.61616800	4.28939100
H	1.19614400	-4.24902200	2.56287200
C	4.77263200	-4.10278900	4.07521100
H	5.38926500	-3.33400800	2.15441700
C	3.69527600	-4.56716700	4.83897900
H	1.57516000	-4.97821600	4.88308800
H	5.76944200	-4.06779200	4.50398200
H	3.85761400	-4.89243400	5.86257700
O	4.11972500	-2.73205500	0.19499900
C	-0.59469200	0.13329500	0.67381000
O	-0.03935900	0.88085900	1.44684700
O	-1.82219200	0.42337800	0.14015600
H	3.38330700	-4.73699700	-0.17867300
B	-2.35869100	1.68423600	0.00885600
C	6.32822100	-4.58777400	-1.68193400
C	5.17660300	-4.51380300	-2.74849400
B	4.33427900	-4.00776000	-0.66607400
O	4.17742500	-3.72873800	-2.05918000
O	5.59251000	-4.65021400	-0.43720800
C	7.19528900	-3.32123400	-1.66211200
H	7.83635700	-3.34599400	-0.77440700
H	7.83808800	-3.25771900	-2.54712400
H	6.57776600	-2.41911700	-1.61363300

C	5.55288500	-3.79771200	-4.04208900
H	6.38561200	-4.30924600	-4.53874700
H	4.69831500	-3.79704000	-4.72783400
H	5.84138900	-2.76048500	-3.85522200
C	4.57774700	-5.89029000	-3.06831700
H	3.66093100	-5.75025400	-3.65099200
H	5.26776900	-6.50381200	-3.65757200
H	4.32310400	-6.43514900	-2.15367500
C	7.21653000	-5.82341500	-1.78974700
H	7.71524700	-5.85838600	-2.76551700
H	7.99082200	-5.79186700	-1.01505800
H	6.64068100	-6.74296700	-1.65863900
O	-3.72466400	1.83916100	-0.03612100
C	-4.63303200	0.99938800	0.57137000
C	-5.95085200	1.00608400	-0.15379600
O	-4.36466200	0.38859200	1.57856900
C	-7.02127600	0.15198000	0.52292100
H	-5.75680300	0.66733500	-1.18098000
H	-6.26867900	2.05320200	-0.24671700
C	-8.33223000	0.19144900	-0.25956900
H	-7.18763600	0.50777800	1.54475500
H	-6.67115100	-0.88156900	0.60828600
C	-9.43771800	-0.64248200	0.36764600
H	-8.17644200	-0.16174800	-1.28859800
H	-8.69238900	1.22542900	-0.35434600
C	-10.77253900	-0.68724900	-0.30663100
O	-9.24108400	-1.26858500	1.40634900
C	-11.03889600	0.02191100	-1.48985200
C	-11.79152700	-1.46700400	0.26688400
C	-12.29919000	-0.04820200	-2.08602700
H	-10.27043700	0.63101500	-1.95317600
C	-13.04890000	-1.53716700	-0.32837600
H	-11.57973100	-2.01268300	1.18042000
C	-13.30547100	-0.82691700	-1.50732400
H	-12.49461900	0.50425500	-3.00055000
H	-13.82929500	-2.14320700	0.12296400
H	-14.28585300	-0.88084300	-1.97242500
O	-1.63431700	2.84014400	-0.15856900
C	-0.35315200	2.90588700	-0.65843200
C	0.39173100	4.08882600	-0.11136900
O	0.05592100	2.09663000	-1.45868200
C	1.79782800	4.23943900	-0.68875700
H	0.41567100	3.96825200	0.98047900
H	-0.22109800	4.98104100	-0.29663100
C	2.51287200	5.44767400	-0.08799400
H	1.74250700	4.34374700	-1.77709000
H	2.37638300	3.33094700	-0.49363800
C	3.91624600	5.65416700	-0.63432800
H	2.57881900	5.35095400	1.00468600
H	1.93563700	6.36547400	-0.26826600
C	4.72490100	6.80197400	-0.11739600
O	4.37903600	4.89591000	-1.48300100
C	4.22931900	7.69303500	0.84919300
C	6.02406500	6.99104900	-0.61901200
C	5.01823700	8.75130800	1.30356000
H	3.23024200	7.56980700	1.25288100
C	6.81119600	8.04687200	-0.16526000
H	6.40131400	6.29960200	-1.36515800
C	6.30912000	8.93015000	0.79803400
H	4.62543100	9.43454300	2.05097800
H	7.81413900	8.18364600	-0.55947600
H	6.92219900	9.75406300	1.15258600

$TS^2_{IV\text{-}V}$

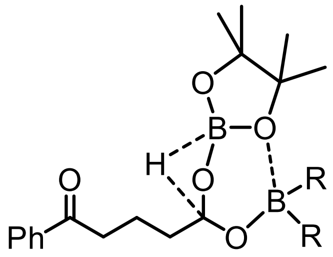

E(M026X)= -2391.12837292
E(B3LYP)= -2392.28704612
G_{Corr}=0.700858

0 1

C	-1.70459300	1.59819300	1.18746800
C	-2.81488200	2.59847500	1.24846300
C	-3.90735100	2.21684700	0.22810400
H	-3.22423300	2.60167800	2.26219000
H	-2.40754500	3.58913900	1.02723100
C	-5.05330800	3.22472900	0.28061300
H	-3.47907000	2.18112300	-0.77710300
H	-4.28248000	1.21265800	0.44756900
H	-5.47857900	3.26963600	1.29278200
H	-4.68295700	4.23697700	0.06820900
O	-1.91436300	0.34967000	1.71779900
C	-6.17671600	2.91100000	-0.69477300
O	-6.12097200	1.92278900	-1.42178300
H	-0.84486500	1.95705200	2.45534700
C	-7.35353800	3.83315600	-0.75008300
C	-7.46025200	4.96326300	0.07773400
C	-8.38501100	3.55448300	-1.66307700
C	-8.57691500	5.79664800	-0.00665600
H	-6.67876000	5.20084700	0.79125900
C	-9.49874400	4.38679000	-1.74768900
H	-8.29644600	2.68039900	-2.29973600
C	-9.59705500	5.51060000	-0.91846900
H	-8.64980800	6.66738500	0.63848200
H	-10.29018000	4.16280300	-2.45723700
H	-10.46547000	6.16021400	-0.98340200
O	-0.84623800	1.70271300	0.20863500
B	0.30366500	0.74367100	0.16187000
C	0.97297100	-0.12230600	3.86121300
C	1.44245200	-0.46994900	2.40133800
B	-0.72962000	0.62188300	2.50071900
O	0.50784600	0.42699400	1.64986800
O	-0.47035400	0.03210400	3.72709000
C	1.55836800	1.19869300	4.37069100
H	1.04499300	1.47447900	5.29665200
H	2.62646300	1.09987400	4.58596000
H	1.42489100	2.00924500	3.64788500
C	2.87740100	-0.08753300	2.08548500
H	3.54600500	-0.61733800	2.77216600
H	3.13612400	-0.38181700	1.06614900
H	3.04556200	0.98487900	2.18916000
C	1.14743200	-1.91119400	2.00073000
H	1.31683100	-2.03879100	0.93068200
H	1.81902900	-2.58635000	2.53874100
H	0.11527200	-2.18887600	2.22883100
C	1.23147600	-1.24206000	4.86076200
H	2.30016600	-1.47630800	4.90894400
H	0.90848300	-0.92057600	5.85563500
H	0.68249200	-2.14839100	4.59665200
O	1.50018300	1.43360100	-0.26602600

C	1.70574800	1.80394900	-1.53794600
C	3.05766600	2.45215800	-1.74540200
O	0.88133000	1.65652300	-2.42618600
C	4.21396600	1.72830400	-1.04336400
H	2.98402800	3.48209000	-1.36720300
H	3.22443600	2.51762500	-2.82471100
C	5.55535500	2.38500400	-1.35974600
H	4.23812700	0.67733000	-1.35387800
H	4.04823000	1.73052500	0.03611700
C	6.73216200	1.72404900	-0.66076100
H	5.53819300	3.44645500	-1.07412800
H	5.74424300	2.37435400	-2.44215800
C	8.10534300	2.27064200	-0.89416300
O	6.56214000	0.76112000	0.08387700
C	8.34139200	3.36794600	-1.73920100
C	9.19277400	1.66306000	-0.24331200
C	9.63904900	3.84636800	-1.92864300
H	7.52000500	3.85500000	-2.25337200
C	10.48756600	2.14054500	-0.43241100
H	9.00419400	0.81610400	0.40809300
C	10.71341500	3.23447700	-1.27652400
H	9.81051400	4.69514200	-2.58432100
H	11.32093400	1.66341200	0.07542000
H	11.72289400	3.60774500	-1.42492400
O	0.08693000	-0.48154200	-0.56050400
C	-0.95713200	-0.86846100	-1.31029600
C	-0.77801900	-2.28068000	-1.82232500
O	-1.95035300	-0.19655600	-1.52452100
C	-1.27751900	-3.29860100	-0.77920200
H	0.28014800	-2.45922800	-2.04064000
H	-1.35098200	-2.38184500	-2.74898200
C	-1.10518600	-4.73147800	-1.27676700
H	-2.33312400	-3.10766800	-0.55682300
H	-0.72918800	-3.16787100	0.15787800
C	-1.55041500	-5.78111300	-0.27202200
H	-0.05272400	-4.92267600	-1.52940000
H	-1.66527300	-4.88304000	-2.20985600
C	-1.43634900	-7.22565700	-0.64572800
O	-1.99205700	-5.45377000	0.82697600
C	-0.93388700	-7.64028300	-1.89046700
C	-1.84588400	-8.19684500	0.28388400
C	-0.84399500	-8.99902900	-2.19782300
H	-0.61076200	-6.91129400	-2.62561400
C	-1.75608600	-9.55261400	-0.02293300
H	-2.23300500	-7.87017000	1.24338100
C	-1.25433300	-9.95676400	-1.26598600
H	-0.45427800	-9.30883300	-3.16317400
H	-2.07570400	-10.29500000	0.70285600
H	-1.18400000	-11.01390700	-1.50665

HBpin-OtBu

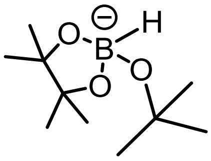

E= -645.007362207

G_{Corr}= 0.272926

-1 1

C	-1.84194600	-0.79402500	0.04927000
C	-1.83160800	0.76758800	-0.11690100
B	0.31723700	0.04974500	0.59368600
C	-2.41790600	1.48373900	1.11562500
H	-2.20526200	2.55627000	1.03343700
H	-3.50472800	1.35627100	1.19157900
H	-1.95743400	1.11355700	2.03676700
C	-2.54708400	1.27485800	-1.37155200
H	-3.60213000	0.97224400	-1.37612000
H	-2.51018900	2.37085200	-1.40270500
H	-2.07180800	0.89464700	-2.28000000
C	-1.68921800	-1.51843800	-1.30158400
H	-1.50086300	-2.58190600	-1.11220000
H	-2.59267900	-1.43725500	-1.91903300
H	-0.84027900	-1.11632300	-1.86144700
C	-3.07316800	-1.34646200	0.77197100
H	-3.99571600	-1.09060900	0.23518600
H	-3.01017000	-2.44010700	0.83115900
H	-3.14463400	-0.95680100	1.79133200
O	-0.44399300	1.05877200	-0.21282900
O	-0.68279700	-1.02942300	0.84314400
O	1.45606700	-0.48286300	-0.17701700
H	0.69708200	0.52543000	1.67349200
C	2.78839300	-0.00967200	-0.03283500
C	3.39112400	-0.46924900	1.30936700
H	3.34635200	-1.56278300	1.38481400
H	4.44111700	-0.16003100	1.39639200
H	2.83310100	-0.04439200	2.14878900
C	3.58920600	-0.63799500	-1.18367400
H	3.53094000	-1.73195900	-1.13117500
H	3.17787200	-0.31981300	-2.14925700
H	4.64566000	-0.34400600	-1.14149800
C	2.86474400	1.52478700	-0.14344500
H	2.43390700	1.85856700	-1.09460500
H	2.30704800	2.00050800	0.66868900
H	3.90719000	1.86623200	-0.09586400

Bpin-OtBu

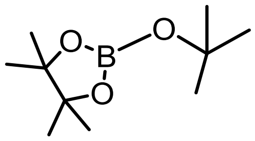

E= -644.34456909

G_{Corr}= 0.267306

-1 1

C	1.99078100	-0.71198200	-0.05927700
C	1.60812400	0.80840000	0.06941600
B	-0.27588200	-0.46177200	-0.15278100
C	1.76599100	1.58402400	-1.24146100
H	1.30146800	2.56880400	-1.12918200
H	2.82135300	1.72820000	-1.49358600
H	1.27694600	1.06770100	-2.07382700
C	2.29603300	1.54799300	1.20950200
H	3.38291400	1.53676800	1.07181700
H	1.96626300	2.59206100	1.22366400
H	2.06213800	1.10153700	2.17866600
C	2.30155300	-1.36983400	1.28856500
H	2.36829400	-2.45324900	1.14728100

H	3.25485200	-1.01625100	1.69399200
H	1.51512000	-1.16842000	2.02326200
C	3.09996600	-1.00732000	-1.06099600
H	4.02579200	-0.50013500	-0.76753700
H	3.29636300	-2.08423200	-1.08657900
H	2.82912200	-0.68466600	-2.06893700
O	0.17616800	0.74110100	0.35189600
O	0.74816400	-1.30232400	-0.54560800
O	-1.56580500	-0.85696800	-0.27972500
C	-2.73677600	-0.03604400	0.00495600
C	-2.72775900	1.20037000	-0.89928500
H	-2.67417800	0.90081400	-1.95199600
H	-3.64791400	1.77683100	-0.75273800
H	-1.87560900	1.84712500	-0.67335200
C	-3.92961500	-0.93481300	-0.32312500
H	-3.90296900	-1.23754600	-1.37559900
H	-3.91136700	-1.83672900	0.29839400
H	-4.86904600	-0.40326700	-0.13757900
C	-2.74356800	0.34992400	1.48722900
H	-2.70458400	-0.54824700	2.11374300
H	-1.88928700	0.98692500	1.73178100
H	-3.66285200	0.89605200	1.72656700

OtBu

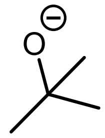

E= -233.119116796

G_{Corr}= 0.093258

-1 1

O	-0.00249100	-0.00119600	1.50967200
C	-0.00023900	-0.00004400	0.13238300
C	0.76447900	1.23149700	-0.43242300
H	1.80213000	1.22018500	-0.07233900
H	0.78312300	1.25954700	-1.53307700
H	0.29288700	2.15652000	-0.07412100
C	0.68524200	-1.27654900	-0.43411600
H	0.15560700	-2.17028800	-0.07736400
H	0.70315800	-1.30411900	-1.53479000
H	1.72113400	-1.33160300	-0.07278500
C	-1.44755900	0.04607900	-0.43604800
H	-1.47904500	0.04776100	-1.53675400
H	-2.01331000	-0.82477100	-0.07804100
H	-1.95730000	0.95043400	-0.07688200

^{f2}TS$_{ester}$

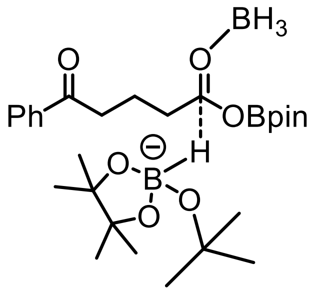

E= -1734.37113679

G_{Corr}= 0.65475

-1 1

C	-4.94268900	-1.55406900	-0.24382900
C	-4.94268900	-1.55406900	-0.24382900
C	-3.70092800	-0.68033100	-0.25728800
C	-2.43186000	-1.41461400	-0.68234400
H	-3.88468800	0.17265400	-0.92521900
H	-3.57245200	-0.24743100	0.74459300
C	-1.22953600	-0.44992600	-0.64000200
H	-2.25459500	-2.26743600	-0.01841400
H	-2.55452400	-1.81323400	-1.69428700
H	-1.39619700	0.39838300	-1.30349500
H	-1.06712100	-0.06759800	0.36866200
C	-6.24768500	-0.93469200	0.15078900
C	-6.35689900	0.42024900	0.50550000
C	-7.39913100	-1.74008600	0.16671700
C	-7.59343000	0.95720800	0.86831400
H	-5.48393600	1.06363800	0.50145400
C	-8.63297400	-1.20421400	0.52865400
H	-7.30807600	-2.78570600	-0.10827800
C	-8.73254400	0.14724500	0.88074100
H	-7.66637000	2.00615700	1.14066600
H	-9.51682800	-1.83580900	0.53739000
H	-9.69430000	0.56647000	1.16331800
O	-4.88374700	-2.74329600	-0.54730200
C	0.02221800	-1.14789300	-1.06408400
O	0.34919300	-1.41100100	-2.24418600
O	0.64474600	-1.80670800	-0.08448300
C	3.89954100	-3.00678100	0.79465700
C	4.14685200	-2.48507600	-0.67227800
B	1.98850300	-2.15502500	-0.07508100
C	4.17207500	-3.60125700	-1.71862100
H	4.15915600	-3.15228800	-2.71648400
H	5.07689100	-4.21006000	-1.62521300
H	3.29969900	-4.25633400	-1.62794800
C	5.35689800	-1.57449200	-0.83165200
H	6.27537500	-2.10965900	-0.56637400
H	5.43817900	-1.25366200	-1.87507200
H	5.27494600	-0.68350600	-0.20557900
C	4.38094100	-2.03099100	1.86876300
H	4.02028600	-2.36881300	2.84527700
H	5.47408000	-1.98774000	1.90109100
H	3.99364200	-1.02434800	1.69184000
C	4.42283700	-4.40950400	1.07105500
H	5.51022000	-4.44177000	0.94196500
H	4.19493800	-4.68916500	2.10475200
H	3.96989300	-5.14921700	0.40712200
O	2.93987600	-1.69645400	-0.93265600
O	2.43425600	-3.03158700	0.87396000
C	0.56180500	3.47084800	-1.00820600
C	2.13698100	3.55157000	-0.90539900
B	1.30632900	1.52406900	0.05610600
C	2.60898200	4.61468700	0.10221300
H	3.68619200	4.49369900	0.26559600
H	2.43225500	5.63370700	-0.26324300
H	2.10628600	4.50018500	1.06591000
C	2.83639400	3.81059500	-2.24481200
H	2.50972000	4.75997300	-2.68837200
H	3.92088100	3.86641000	-2.08985700
H	2.63792300	3.00651700	-2.95854300
C	0.08338300	2.93490700	-2.37078200
H	-0.99743200	2.75917900	-2.31771000
H	0.27234900	3.64837000	-3.18164400
H	0.56176300	1.98553600	-2.62100100
C	-0.15575400	4.79269600	-0.72370100
H	0.14876700	5.57029200	-1.43541800
H	-1.23887100	4.65099000	-0.82184600

H	0.04814100	5.14905400	0.28960900
O	2.50833200	2.26394100	-0.41792400
O	0.22586700	2.53287500	0.00693900
H	1.06973900	0.59531800	-0.75901100
B	-0.26592800	-0.88761100	-3.60117700
H	-1.29668700	-0.28625600	-3.38587700
H	0.59963400	-0.18347600	-4.08596100
H	-0.44523700	-1.90329400	-4.24929000
O	1.42836400	0.81356000	1.31979700
C	1.68373000	1.35725200	2.61299300
C	3.12582600	1.88557300	2.71449600
H	3.28609200	2.70886100	2.01685100
H	3.34066200	2.23394500	3.73354600
H	3.83762700	1.08985200	2.46693900
C	1.50257000	0.18534300	3.59107000
H	1.70209000	0.49715800	4.62401900
H	0.47608700	-0.19702200	3.53600600
H	2.18425200	-0.63262000	3.33679200
C	0.68944300	2.46996400	2.98900000
H	0.78907400	3.33231400	2.32806800
H	-0.33953100	2.10252800	2.90329300
H	0.85811300	2.79218300	4.02519000

$^{f2}TS_{ketone}$

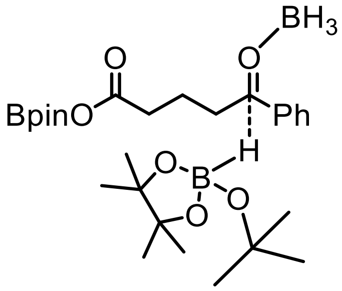

E= -1734.3773333

G_{Corr}= 0.653964

-1 1

C	-3.15475100	-0.57421500	0.03898200
C	-1.79843900	-0.01133100	0.37454600
C	-0.75728600	-1.07358700	0.71897100
H	-1.47352900	0.59868000	-0.47873400
H	-1.93171100	0.69429600	1.20565600
C	0.59492900	-0.40079300	1.02890000
H	-1.09298500	-1.65608700	1.58320700
H	-0.64966500	-1.77109700	-0.11805800
H	0.95146900	0.16769300	0.16751200
H	0.47428300	0.29474800	1.86043900
C	1.63064900	-1.41686600	1.41412200
O	-3.46004700	-1.74720800	0.03201400
O	1.79460600	-1.75955100	2.62863800
C	2.77368600	2.59500400	-0.60159100
C	3.38383600	2.94376500	0.81440700
B	3.76618900	0.66121200	0.39935300
C	2.32845000	2.94349600	1.93487900
H	2.84132000	3.03201600	2.89940800
H	1.62858700	3.78202500	1.84051200
H	1.76003700	2.01226400	1.94548900
C	4.14151000	4.27343800	0.86154900
H	3.48185900	5.11309600	0.60959600
H	4.52779000	4.43952500	1.87416200
H	4.98913300	4.27748500	0.17127700
C	3.58061200	3.20358800	-1.76134600
H	3.21691600	2.78139800	-2.70521600
H	3.46796200	4.29326300	-1.80927900
H	4.64424700	2.96923200	-1.67400500
C	1.30679200	3.00698500	-0.76920600
H	1.18667800	4.09296200	-0.66867400
H	0.95617000	2.71921100	-1.76774800
H	0.66412800	2.52001100	-0.03219200
O	4.30125400	1.87894800	1.03806100
O	2.87778600	1.17271900	-0.67401700
H	3.02838000	0.00818300	1.21561300
C	2.20295600	-2.32316100	0.39606400
C	2.09867000	-2.03554000	-0.97408100
C	2.82817600	-3.51510400	0.80053800
C	2.59705000	-2.92900500	-1.91968900
H	1.67021100	-1.09599800	-1.30008600
C	3.33436900	-4.40254900	-0.14658200
H	2.90756900	-3.73652700	1.85905300
C	3.21636600	-4.11375000	-1.51074100
H	2.51612500	-2.69297900	-2.97685100
H	3.81715900	-5.32053600	0.17683700
H	3.60955700	-4.80629400	-2.25013000
O	-4.02844700	0.41557200	-0.30804800
C	-7.52216500	-0.40999700	0.13383700
C	-7.54998300	0.88166900	-0.76757600
B	-5.40990100	0.28216200	-0.31138000
O	-6.15786500	0.96943500	-1.22525800
O	-6.13068500	-0.43081000	0.60142700
C	-8.44043100	-0.36242700	1.34695100
H	-9.48411800	-0.26290800	1.02932900
H	-8.34549300	-1.29242800	1.91646600
H	-8.19484600	0.47166600	2.00792900
C	-7.73768300	-1.70464500	-0.65271900
H	-7.51430500	-2.55613700	-0.00275300
H	-8.77460700	-1.79181000	-0.99167700
H	-7.07961000	-1.75961600	-1.52564700
C	-8.45703100	0.78990400	-1.98673200
H	-9.49674900	0.63984300	-1.67601900
H	-8.40376500	1.72340000	-2.55609000
H	-8.16696000	-0.03141500	-2.64566400
C	-7.83386600	2.16395200	0.01762300
H	-7.64963800	3.02593000	-0.63105400
H	-8.87578600	2.19999400	0.35042400
H	-7.18518500	2.25065600	0.89514000
B	1.20030400	-1.05294200	3.88724300
H	1.65077900	0.08030400	3.92266000
H	1.58482600	-1.72906000	4.82436500
H	-0.01688200	-1.04994400	3.79399400
O	4.76053700	-0.32956300	0.05308200
C	5.80128000	-0.24793900	-0.91773600
C	6.75401700	0.92612000	-0.63490600
H	7.58626600	0.92539900	-1.35120800
H	6.23136900	1.88243900	-0.70190500
H	7.16745000	0.84217900	0.37683300
C	5.23146700	-0.14176500	-2.34286200
H	6.04311300	-0.15985100	-3.08232200
H	4.56321900	-0.98642700	-2.53976100
H	4.65924300	0.77814400	-2.46827300
C	6.57564600	-1.56915800	-0.78875100
H	5.90610300	-2.41700700	-0.97353400
H	7.40443200	-1.61431900	-1.50646300
H	6.98643400	-1.67063600	0.22303100

$^{f0}TS_{ester}$

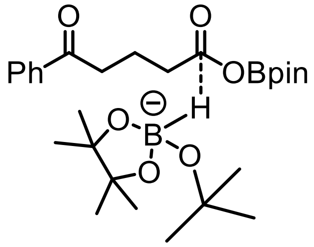

E= -1734.37113679

G$_{Corr}$= 0.65475

-1 1

C	3.80252600	-2.32513500	-0.27972200
C	2.39250500	-1.96354500	0.12546600
C	1.44287100	-3.15058300	0.26208900
H	2.43914700	-1.40499700	1.07000500
H	2.00534300	-1.22744800	-0.58806700
C	0.08166900	-2.75883900	0.84054600
H	1.30439100	-3.63466900	-0.71091500
H	1.89744000	-3.90008100	0.92139000
H	-0.47465900	-3.67153600	1.10279000
H	0.19686800	-2.17330500	1.75640400
C	4.81452300	-1.22106300	-0.36325100
C	4.44924200	0.12701400	-0.20760700
C	6.16063800	-1.54780300	-0.59959900
C	5.42220700	1.12624800	-0.28771700
H	3.41180900	0.40698600	-0.04995000
C	7.12878400	-0.54829500	-0.67406900
H	6.43199900	-2.59188400	-0.71982000
C	6.76023900	0.79406800	-0.51741100
H	5.13108900	2.16702400	-0.17196500
H	8.16799900	-0.81068900	-0.85328000
H	7.51382100	1.57514100	-0.57581800
O	4.13013000	-3.48619100	-0.52955200
C	-0.83533500	-1.99813000	-0.11669200
O	-0.90625500	-2.26130300	-1.32653300
O	-2.01294000	-1.67393500	0.62097100
C	-5.37612800	-0.54898900	0.47395600
C	-4.91132400	-0.22384000	-0.99372500
B	-3.18353200	-1.13439900	0.19273800
C	-4.60880200	1.26146900	-1.20923500
H	-4.17036600	1.39749200	-2.20183200
H	-5.52346300	1.86070100	-1.15637800
H	-3.89427900	1.63513200	-0.47049700
C	-5.84355500	-0.73247100	-2.08723500
H	-6.83036400	-0.26404500	-1.99852200
H	-5.43214600	-0.47489800	-3.06883900
H	-5.96593200	-1.81712500	-2.04235900
C	-6.17044300	-1.85548600	0.57971400
H	-6.29530600	-2.10978800	1.63726900
H	-7.16358800	-1.75743200	0.12936400
H	-5.64633400	-2.68249700	0.08959600
C	-6.12477500	0.57897900	1.17439000
H	-7.04622600	0.82409300	0.63440000
H	-6.39805100	0.26451800	2.18728000
H	-5.51256600	1.48065300	1.24965800
O	-3.64784700	-0.94843100	-1.09028600
O	-4.11069100	-0.76376000	1.15588700
C	1.07837700	2.00648200	-1.25594600
C	-0.49309300	1.96007400	-1.37156400
B	0.13732500	0.62547200	0.42730300
C	-0.96398100	0.93508100	-2.41403000
H	-2.04455400	0.80926900	-2.32576300
H	-0.73104800	1.26721100	-3.43282700
H	-0.51618100	-0.04678200	-2.24593000
C	-1.14472000	3.31197000	-1.66270500
H	-0.78471000	3.72271100	-2.61407400
H	-2.23060500	3.18890300	-1.73727800
H	-0.93810300	4.03703000	-0.87071600
C	1.60360100	3.34878700	-0.72256100
H	2.66938200	3.24604700	-0.48954700
H	1.49253400	4.14986400	-1.46272700
H	1.08452400	3.64751400	0.19138400
C	1.82045000	1.67286400	-2.55335500
H	1.55967800	2.37978600	-3.35069700
H	2.90156400	1.73423800	-2.38505200
H	1.58907300	0.66095200	-2.89468000
O	-0.89373900	1.52500200	-0.06566800
O	1.35934400	0.99423800	-0.28463300
O	0.21199600	0.40033100	1.83884900
H	-0.18419600	-0.60751900	0.01855300
C	0.68642900	1.30443800	2.83974700
C	2.18267200	1.60537000	2.65142600
H	2.36353600	2.13458000	1.71500100
H	2.55758100	2.21474700	3.48388300
H	2.75258900	0.66945600	2.62394700
C	-0.13010700	2.60633400	2.84542500
H	-0.01617500	3.14767800	1.90395600
H	-1.19481200	2.38312700	2.97777100
H	0.19372000	3.25903500	3.66628000
C	0.48133000	0.57110400	4.17279300
H	-0.57876900	0.33006800	4.31349600
H	1.05036200	-0.36594000	4.18084000
H	0.81425000	1.18708300	5.01701300

$^{f0}TS_{ketone}$

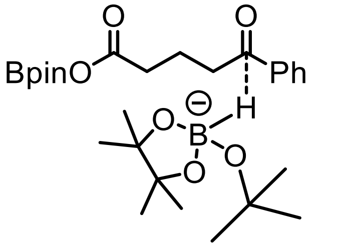

E= -1707.73018678

G$_{Corr}$= 0.622067

-1 1

C	2.35690000	-1.16499900	-1.41907000
C	1.05302800	-1.76875600	-0.86978400
C	-0.11011500	-0.77941200	-0.87500200
H	0.79934400	-2.63667600	-1.49986000
H	1.21584700	-2.15208600	0.14417400
C	-1.37886000	-1.40474200	-0.30079000
H	0.16708300	0.10400300	-0.29350000
H	-0.29188700	-0.43380100	-1.89689100
H	-1.68008700	-2.29695400	-0.86867900
H	-1.22146000	-1.75668800	0.72796100
C	3.61882000	-1.98385100	-1.16539700
C	3.57560200	-3.35270500	-0.87082200
C	4.87151500	-1.36662900	-1.30218900
C	4.75588900	-4.09023500	-0.71266600
H	2.62096100	-3.85797300	-0.76046700

C	6.04874800	-2.09435200	-1.13391800
H	4.90146900	-0.30148800	-1.50623100
C	5.99692600	-3.46388800	-0.84002700
H	4.70075400	-5.15260400	-0.48671000
H	7.01063700	-1.59496600	-1.22543800
H	6.91445800	-4.03241200	-0.71009800
O	2.30336000	-0.54034700	-2.52998900
C	-2.56410800	-0.47640700	-0.27670600
O	-2.60022600	0.65683500	-0.70496600
O	-3.64088500	-1.05541600	0.33984300
C	-7.16560800	-0.48914300	0.65308400
C	-6.84329100	0.27162000	-0.68862600
B	-4.95014100	-0.62896700	0.17729800
C	-6.73201000	1.78791100	-0.51506000
H	-6.32422300	2.22133600	-1.43335500
H	-7.71256100	2.23636300	-0.32717000
H	-6.06432700	2.05027600	0.31158400
C	-7.76334800	-0.06674300	-1.85351800
H	-8.79589900	0.21280100	-1.61761500
H	-7.45341600	0.49501300	-2.74049200
H	-7.73568000	-1.13167400	-2.09480300
C	-7.76405300	-1.88022700	0.43350600
H	-7.78316900	-2.41355500	1.38901800
H	-8.78898500	-1.81376100	0.05562500
H	-7.16959000	-2.46670100	-0.27433200
C	-8.00194800	0.30068500	1.65045200
H	-8.98101200	0.53984200	1.22122800
H	-8.16337100	-0.29965100	2.55158100
H	-7.51098100	1.23186600	1.94160000
O	-5.49541700	-0.21468200	-1.00370500
O	-5.82855900	-0.69274800	1.22272000
C	3.51535800	2.94642500	-0.49369800
C	1.95619900	2.85846800	-0.30563700
B	2.96071500	0.91028800	0.51217500
C	1.22521800	2.47900500	-1.60003600
H	0.17091000	2.29243700	-1.36934300
H	1.27253100	3.29164700	-2.33504100
H	1.64052700	1.56752400	-2.03883500
C	1.31933400	4.11653200	0.28614400
H	1.48474300	4.98188500	-0.36745500
H	0.23749400	3.96871600	0.38307000
H	1.72157400	4.34555300	1.27650200
C	4.19331000	3.80516100	0.58652300
H	5.27855400	3.67204600	0.51317500
H	3.96962300	4.87124600	0.46247300
H	3.87909000	3.49527200	1.58800900
C	3.96199900	3.41955200	-1.87589700
H	3.57895900	4.42503100	-2.08932100
H	5.05694700	3.45860500	-1.91781200
H	3.61452900	2.73957800	-2.65750400
O	1.81909300	1.78957600	0.64605100
O	3.93845200	1.58788200	-0.30537600
O	3.52761000	0.35526200	1.69677200
H	2.54975800	-0.18998200	-0.29960600
C	2.89191300	-0.41898800	2.71775600
C	3.30378000	-1.88749000	2.53501100
H	4.39629800	-1.97652700	2.54405700
H	2.89670500	-2.51282400	3.33953800
H	2.94561800	-2.27298800	1.57790400
C	3.44049900	0.11034400	4.05113900
H	4.53491900	0.04906700	4.05992000
H	3.15292400	1.15889300	4.19053000
H	3.05300000	-0.47236400	4.89588900
C	1.36133500	-0.29601700	2.71157900
H	1.05182500	0.74342500	2.84707100
H	0.93996600	-0.65057200	1.76874300
H	0.94111800	-0.90041300	3.52463100

$P_{1a\text{-}f2\text{-}ketone}$

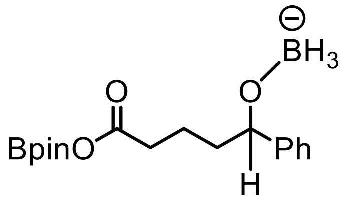

E= -1090.10284586

G_{Corr}= 0.364744-1 1

-1 1

C	-0.61988900	-0.72228000	0.07576100
C	0.55947700	-1.20999700	-0.72365700
C	1.91578400	-0.91140300	-0.08887000
H	0.42394200	-2.28811000	-0.88420600
H	0.47918300	-0.75560300	-1.72177400
C	3.06751900	-1.40692600	-0.96402100
H	2.00873400	0.16768600	0.08059100
H	1.97722400	-1.39045700	0.89391100
H	2.95216600	-2.48130500	-1.14837500
H	3.03634100	-0.90710500	-1.94267300
O	-0.57771800	-0.10322200	1.11730500
C	4.46713800	-1.18400400	-0.33506100
O	4.61975200	-1.82812600	0.89666100
B	4.65095100	-3.32517500	0.89021800
H	5.07626000	-3.69023800	1.99311700
H	3.52713900	-3.82941200	0.70688600
H	5.41085400	-3.73927800	-0.01012400
H	5.19774900	-1.59363100	-1.06007500
C	4.74957900	0.30555100	-0.19103300
C	5.01228700	1.08457000	-1.32767600
C	4.72176500	0.93705300	1.05810100
C	5.23405300	2.46010200	-1.22315000
H	5.04698700	0.60791900	-2.30560500
C	4.94243100	2.31416900	1.16947600
H	4.52772000	0.33380300	1.93839100
C	5.19796400	3.08279800	0.02988800
H	5.44014700	3.04449800	-2.11676700
H	4.91460400	2.78694300	2.14855800
H	5.37152900	4.15252000	0.11572200
O	-1.80370600	-1.09325200	-0.50232700
C	-4.60898300	1.02971200	0.33308700
C	-5.28276900	-0.27466700	-0.23917000
O	-3.20754100	0.85365700	-0.06409900
O	-4.16988200	-1.23093400	-0.22267800
B	-3.02258200	-0.48803000	-0.23591200
C	-5.72298100	-0.14025400	-1.69852800
H	-6.00274400	-1.12807500	-2.07765300
H	-6.58964500	0.52176700	-1.78987600
H	-4.91746900	0.25238600	-2.32732600
C	-6.41510000	-0.83417900	0.61099100
H	-7.22961800	-0.10562600	0.68707500
H	-6.81244400	-1.74089100	0.14356700
H	-6.07711700	-1.08708900	1.61832500
C	-4.62429600	1.10082600	1.86155900
H	-3.97971500	1.92387100	2.18490200
H	-5.63531700	1.28560500	2.23766900
H	-4.24823400	0.17639900	2.31101000
C	-5.12271500	2.33094100	-0.26750000
H	-6.19142300	2.45077000	-0.05876100
H	-4.59182900	3.17692100	0.18064100

H	-4.97168400	2.36470000	-1.34874500

P$_{1a\text{-}f2\text{-}ester}$

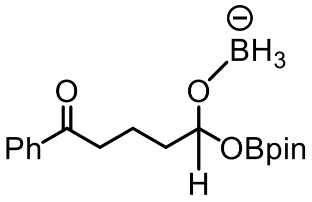

E= -1090.09600378

G$_{Corr}$= 0.36624

-1 1

C	3.85692500	-0.27020200	0.56309900
C	2.86870000	0.39365200	-0.37822000
C	1.43478800	0.45332400	0.14418100
H	3.23562200	1.40685200	-0.59771400
H	2.90524100	-0.14150700	-1.33869900
C	0.50225000	1.13409700	-0.85978500
H	1.07818700	-0.55976700	0.35884900
H	1.40960600	1.00209600	1.09098800
H	0.86728700	2.14579300	-1.07686500
H	0.49403000	0.58250600	-1.81054700
C	5.29551500	-0.35999800	0.14654500
C	5.75302400	0.14091100	-1.08348000
C	6.21427500	-0.96691200	1.01934300
C	7.10110400	0.03602200	-1.43187400
H	5.06490500	0.61396600	-1.77539000
C	7.55966300	-1.07112700	0.67256800
H	5.85522500	-1.35146400	1.96829200
C	8.00647300	-0.56930300	-0.55569000
H	7.44321600	0.42704000	-2.38587800
H	8.26083500	-1.54170100	1.35616000
H	9.05541500	-0.64974900	-0.82759000
O	3.49909400	-0.72987400	1.64516000
C	-0.94146500	1.27649300	-0.37098000
O	-1.06973800	1.92974700	0.83253100
O	-1.47042600	-0.06713400	-0.21650200
C	-4.77094200	-1.41907700	-0.05884200
C	-5.05745600	0.12811600	-0.08708700
B	-2.81001700	-0.25344300	-0.15906900
C	-5.34918300	0.71276700	1.29837400
H	-5.34104400	1.80506600	1.23023600
H	-6.33085700	0.39626800	1.66556300
H	-4.58990600	0.41217900	2.02733100
C	-6.12849200	0.56253300	-1.07988800
H	-7.09047600	0.09956300	-0.83274400
H	-6.25261500	1.64954100	-1.03438500
H	-5.86268500	0.29103900	-2.10422500
C	-4.96020300	-2.09044400	-1.42279500
H	-4.56407500	-3.10991500	-1.37436600
H	-6.01865500	-2.14502300	-1.69716200
H	-4.42383500	-1.55199600	-2.21091400
C	-5.52742700	-2.18785500	1.01759300
H	-6.60878300	-2.09696400	0.86526400
H	-5.26502700	-3.24986100	0.96571100
H	-5.28369800	-1.82354900	2.01825100
O	-3.77379100	0.67003900	-0.51403400
O	-3.34615000	-1.46339200	0.24559700
B	-1.03927200	3.43548400	0.79242100
H	-1.35017600	3.83805400	1.91570900
H	0.08935100	3.86843100	0.49896900
H	-1.84965600	3.85751800	-0.05322700
H	-1.53820200	1.78232000	-1.14876300

P$_{1a\text{-}f0\text{-}ketone}$

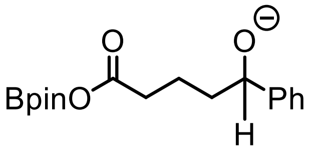

E= -1063.41287238

G$_{Corr}$= 0.33589

-1 1

C	4.19988500	-1.00297100	-0.37699900
C	3.02663800	-0.01339500	-0.07700700
C	1.66637600	-0.57968300	-0.48202300
H	3.03723800	0.19813300	1.00398100
H	3.19115700	0.94480800	-0.59450700
C	0.51739900	0.35580700	-0.11023000
H	1.65350800	-0.76087500	-1.56579900
H	1.52627400	-1.55193500	0.00129400
H	0.48610200	0.53098300	0.97417500
H	0.65111300	1.35095300	-0.55825200
C	5.51417800	-0.23940100	-0.10104700
C	6.28371600	-0.53391900	1.03061600
C	5.96847200	0.77288100	-0.96155700
C	7.46290900	0.16801200	1.30714000
H	5.94075500	-1.33518500	1.67889800
C	7.14691100	1.47677700	-0.69760800
H	5.39192200	1.00797600	-1.85569500
C	7.90114000	1.17823300	0.44446500
H	8.04457100	-0.07756800	2.19367600
H	7.48127800	2.25320300	-1.38278900
H	8.82039200	1.72068100	0.65271600
O	4.08400400	-2.18647800	0.28448700
C	-0.84690500	-0.13052400	-0.52325300
O	-1.09623900	-1.14334400	-1.14048200
O	-1.82435600	0.75531400	-0.15030300
C	-5.38513200	0.88009500	0.20612800
C	-5.12932200	-0.67396800	0.18369000
B	-3.17101000	0.44645300	-0.04825300
C	-5.43060000	-1.31902300	-1.17090700
H	-5.05101700	-2.34533000	-1.16750700
H	-6.50782300	-1.34988900	-1.36190900
H	-4.94621700	-0.77878500	-1.99046000
C	-5.80397400	-1.45032900	1.30608000
H	-6.89240300	-1.34480400	1.24080100
H	-5.55942200	-2.51348300	1.21535400
H	-5.47724700	-1.10572600	2.28968000
C	-5.57303200	1.44296300	1.61665500
H	-5.55467000	2.53619200	1.56858800
H	-6.53336200	1.13292000	2.04015600
H	-4.77407800	1.11608500	2.28983600
C	-6.50188000	1.35729600	-0.71209800
H	-7.45644100	0.91004900	-0.41399900
H	-6.59868100	2.44532400	-0.63873900
H	-6.30551400	1.09957800	-1.75515600
O	-3.67640200	-0.75054700	0.37099000
O	-4.11103200	1.41003400	-0.29218500
H	4.17915600	-1.12850100	-1.49541200

$P_{1a\text{-}f0\text{-}ester}$

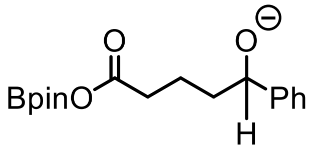

E= -1063.41566426

G_{Corr}= 0.335159

-1 1

C	4.20154500	-0.76346300	0.12912000
C	2.97055100	0.04553200	-0.23557700
C	1.66417200	-0.74639900	-0.24268500
H	2.90095900	0.89090500	0.46465000
H	3.14231000	0.50176900	-1.22142500
C	0.46337400	0.12975700	-0.60813700
H	1.73913000	-1.57774600	-0.95164800
H	1.50746700	-1.19670400	0.74709500
H	0.39387000	0.97590300	0.09058100
H	0.59136500	0.54486900	-1.61816800
C	5.53324100	-0.07232100	0.15803000
C	5.67596800	1.29365200	-0.13676700
C	6.67480500	-0.82014000	0.49263500
C	6.93427900	1.89765800	-0.09654300
H	4.81116500	1.89336200	-0.39852000
C	7.93066300	-0.21807700	0.53248900
H	6.55838400	-1.87494300	0.71893400
C	8.06312600	1.14418800	0.23782800
H	7.03219000	2.95490700	-0.32610300
H	8.80613400	-0.80667700	0.79232700
H	9.04179200	1.61527500	0.26874600
O	4.12358100	-1.96000400	0.39917000
C	-0.85182000	-0.66704300	-0.58166100
O	-0.98435100	-1.62145400	-1.45242700
O	-1.92193100	0.51643800	-0.82246000
C	-5.34330700	0.94605600	0.11233200
C	-5.08605500	-0.55523100	0.49972400
B	-3.17317100	0.36349600	-0.38123400
C	-5.60825700	-1.54171000	-0.55147500
H	-5.22517200	-2.54089700	-0.32019000
H	-6.70264100	-1.58374000	-0.55401200
H	-5.26675800	-1.27065900	-1.55570000
C	-5.58558700	-0.95426400	1.88405300
H	-6.67109900	-0.82037500	1.95585400
H	-5.36103400	-2.01081500	2.06622500
H	-5.10788600	-0.36361700	2.66954500
C	-5.27536500	1.89293200	1.31673400
H	-5.26791100	2.92654400	0.95515000
H	-6.13971800	1.76576700	1.97712900
H	-4.36400000	1.72861900	1.90101500
C	-6.62708300	1.19221800	-0.67295600
H	-7.50218400	0.88950100	-0.08638100
H	-6.72292100	2.25991900	-0.89875100
H	-6.63209500	0.64204500	-1.61696600
O	-3.63818500	-0.63224300	0.48115000
O	-4.20523900	1.24205500	-0.73644600
H	-1.09198300	-0.93909500	0.47201700

7. Copies of NMR spectra

1-phenyl-5-((4,4,5,5-tetramethyl-1,3,2-dioxaborolan-2-yl)oxy)pentan-1-one (2a) and 2,2'-((1-phenylpentane-1,5diyl)bis(oxy))bis(4,4,5,5-tetramethyl-1,3,2-dioxaborolane) (2a')

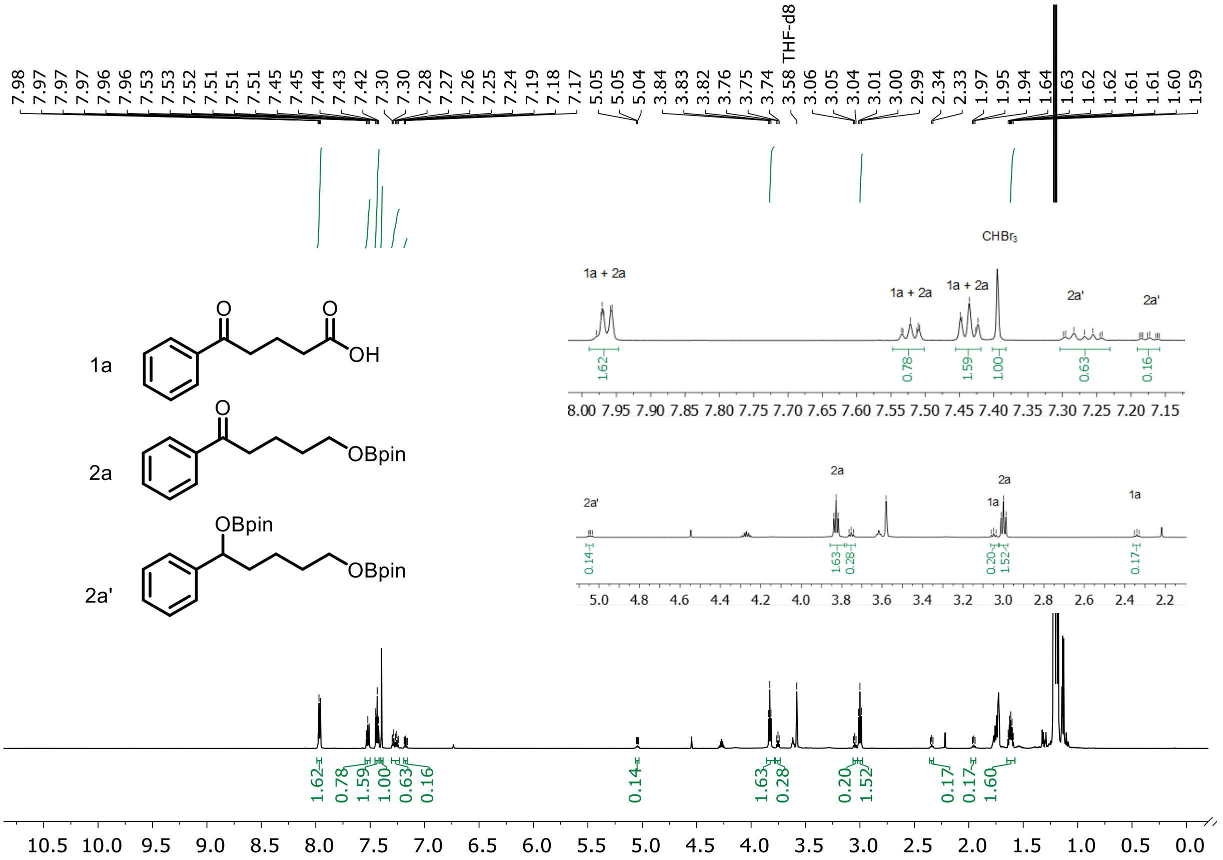

6-phenyl-1-hexanol (2b)

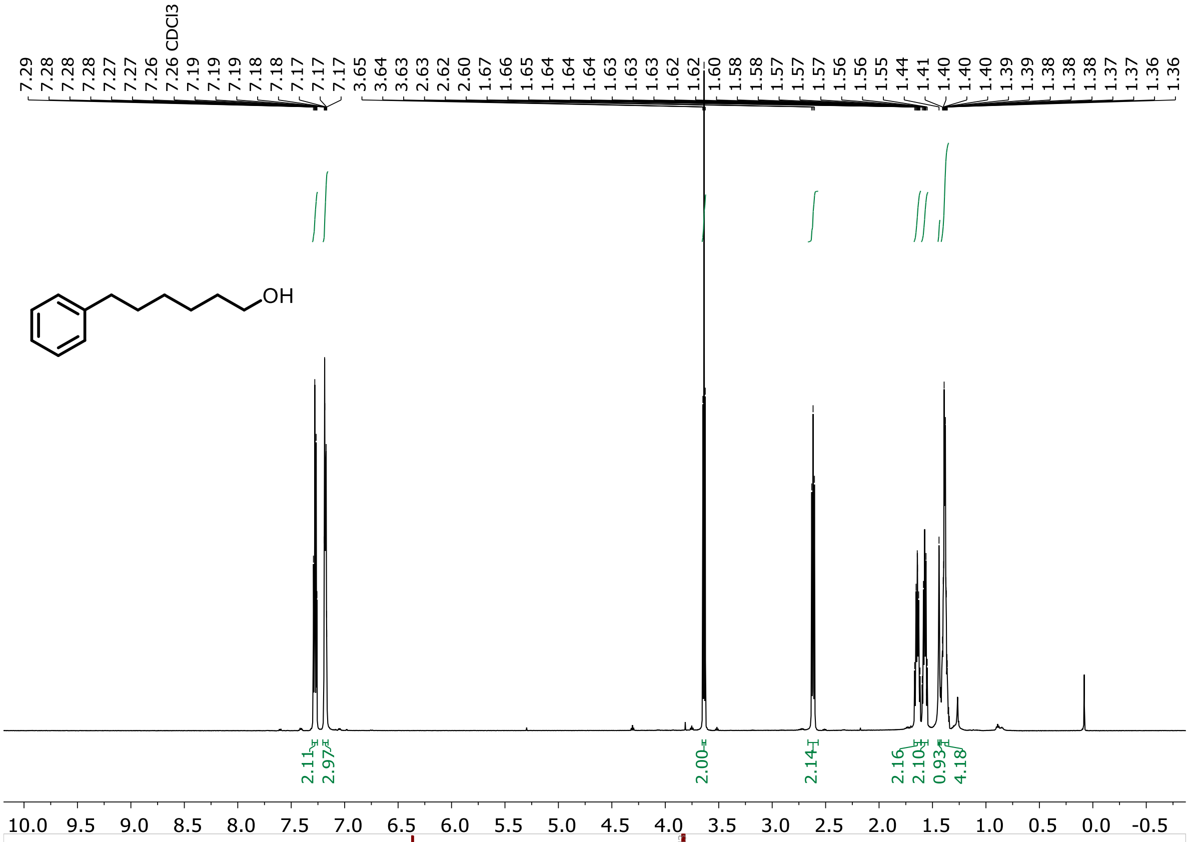

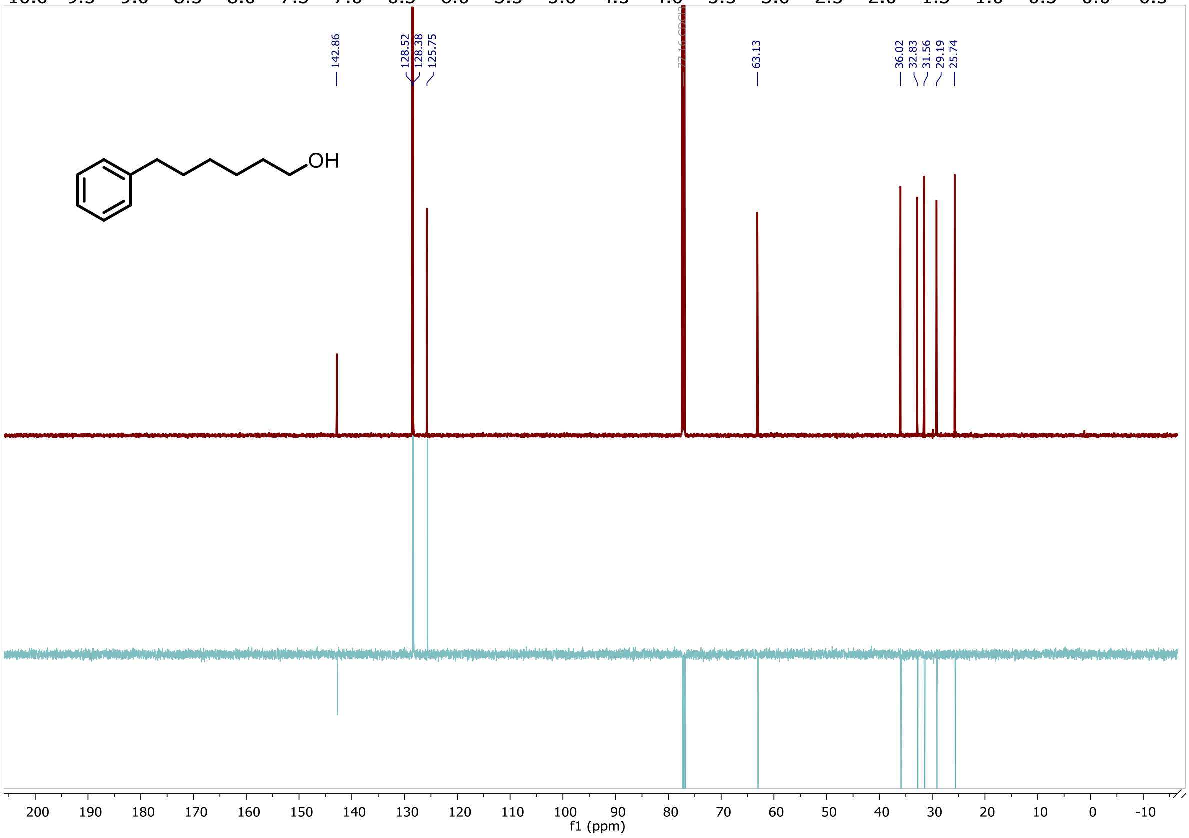

2-methyl-3-phenylpropanol (2c)

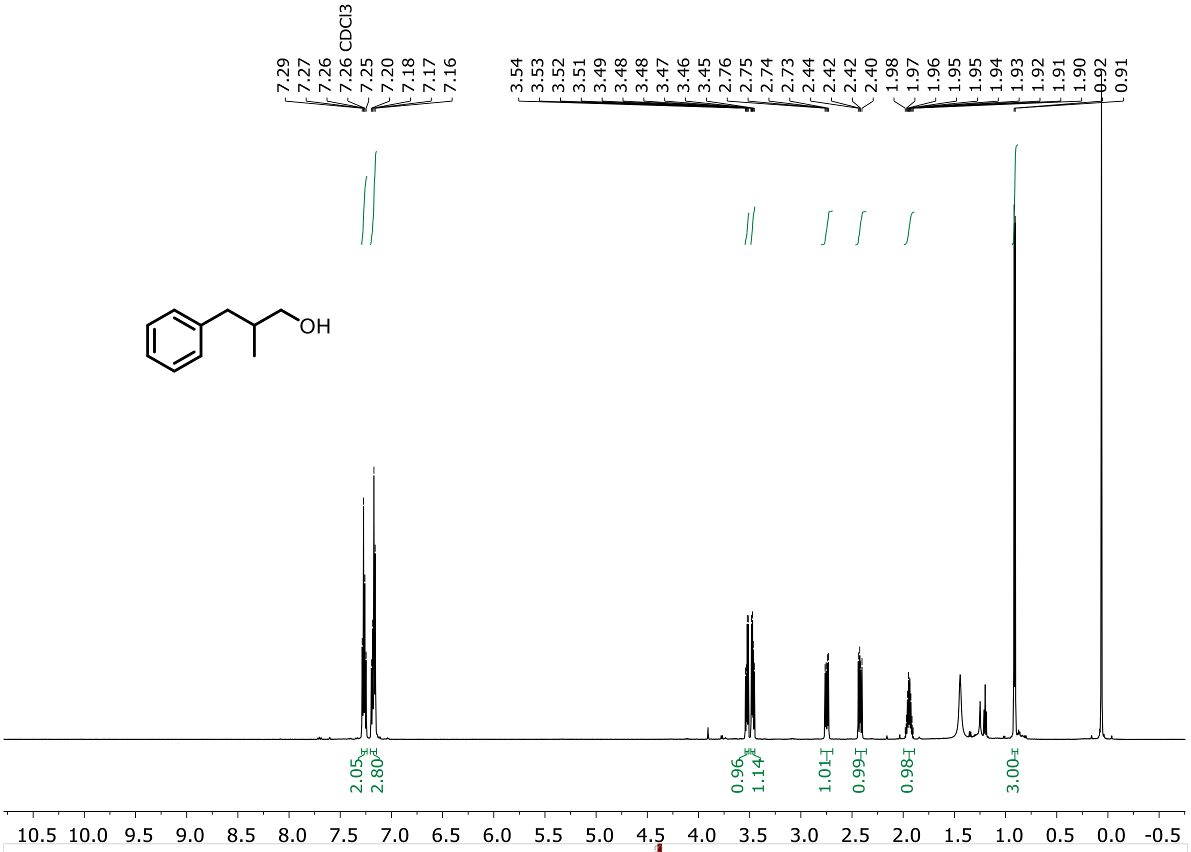

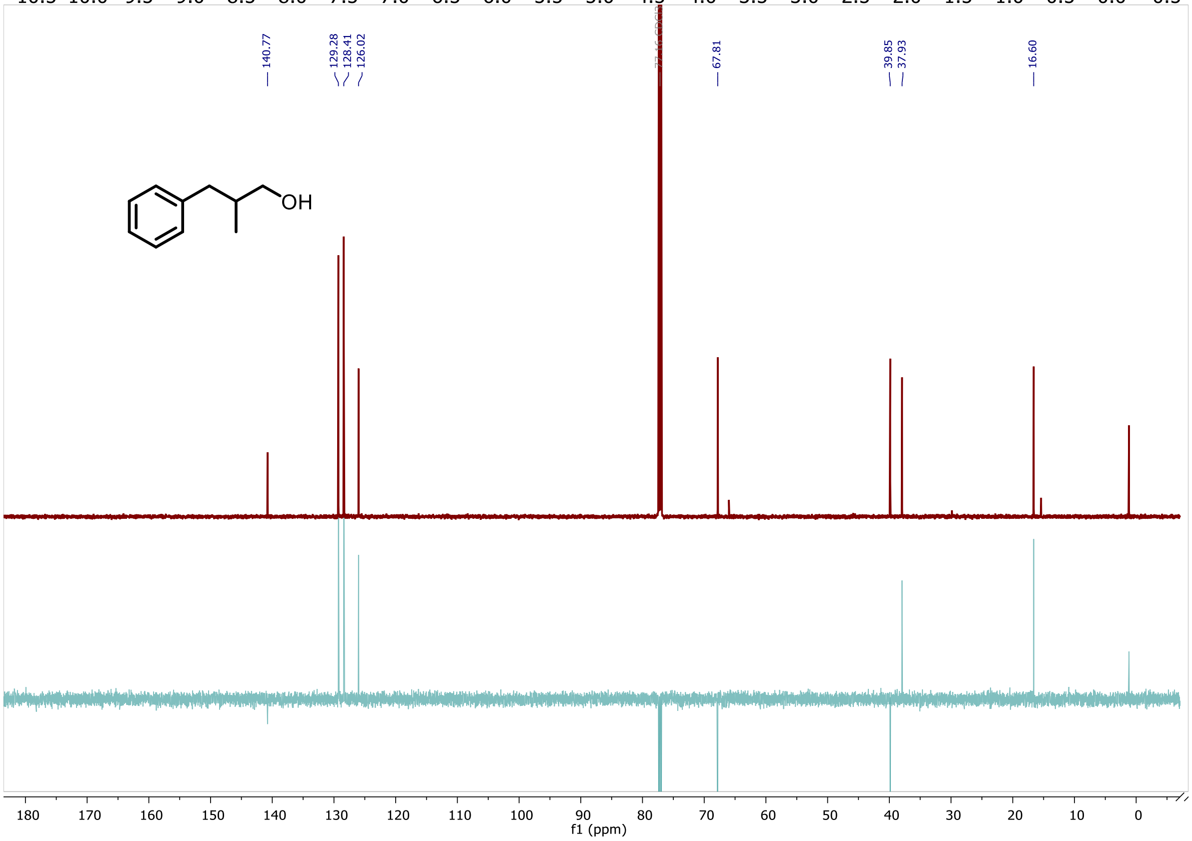

Benzyl alcohol (2d)

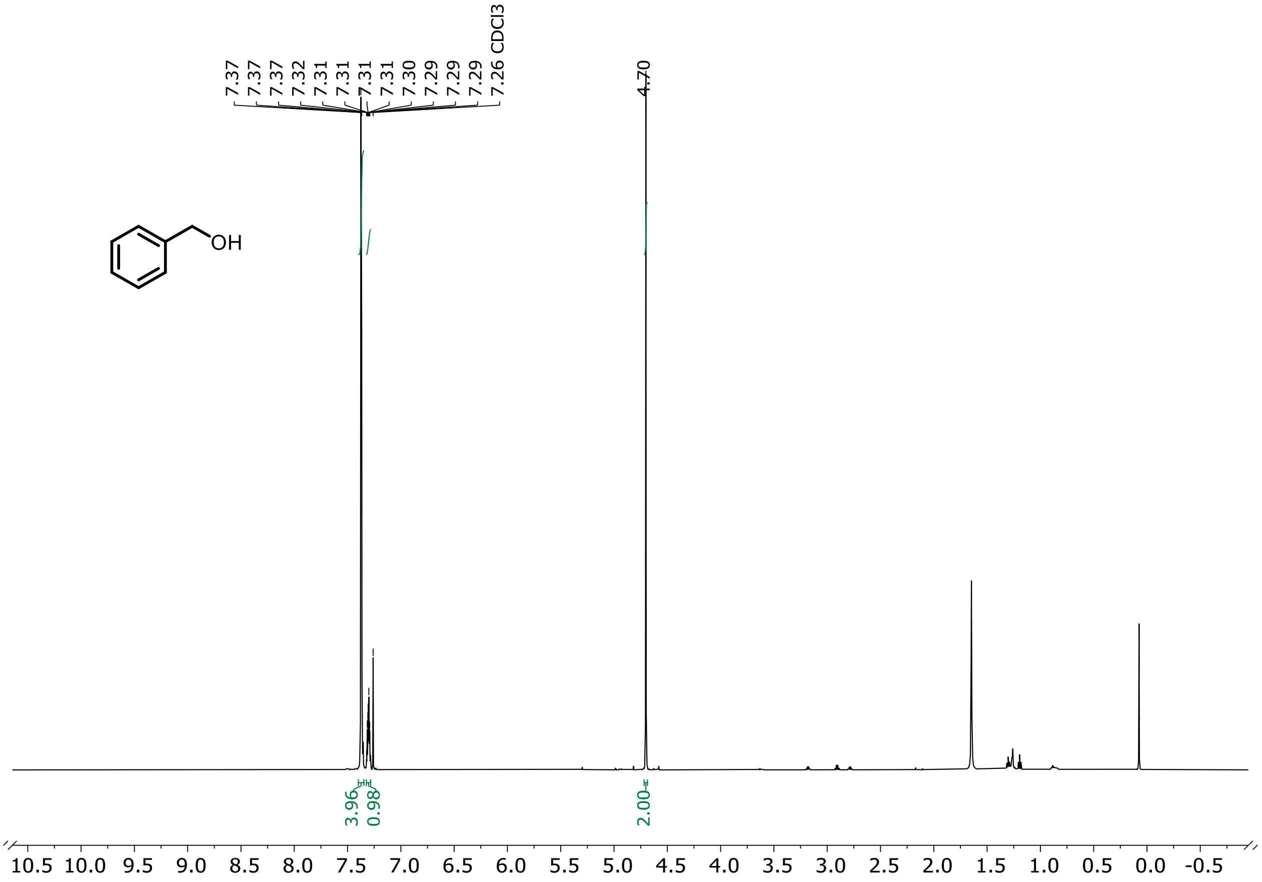

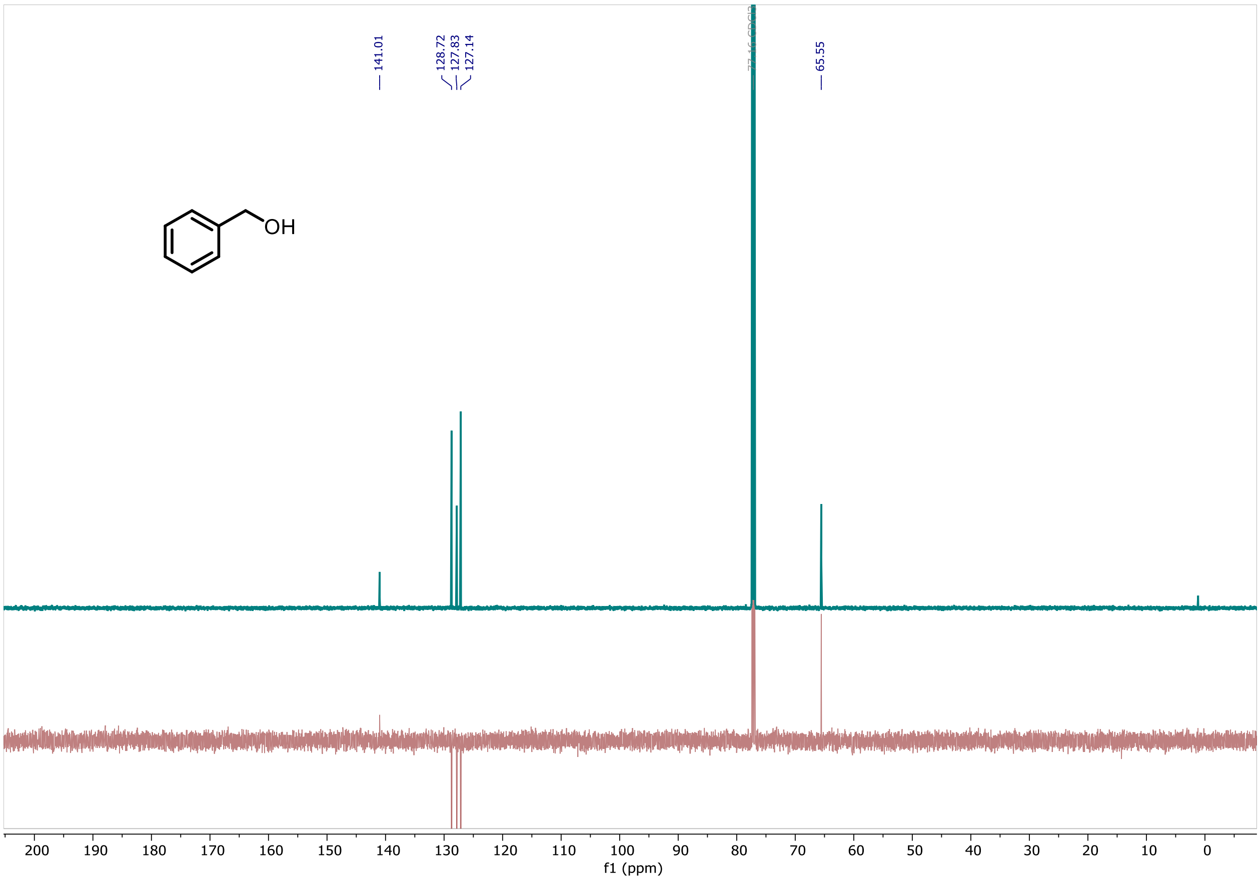

4-methyl benzyl alcohol (2e)

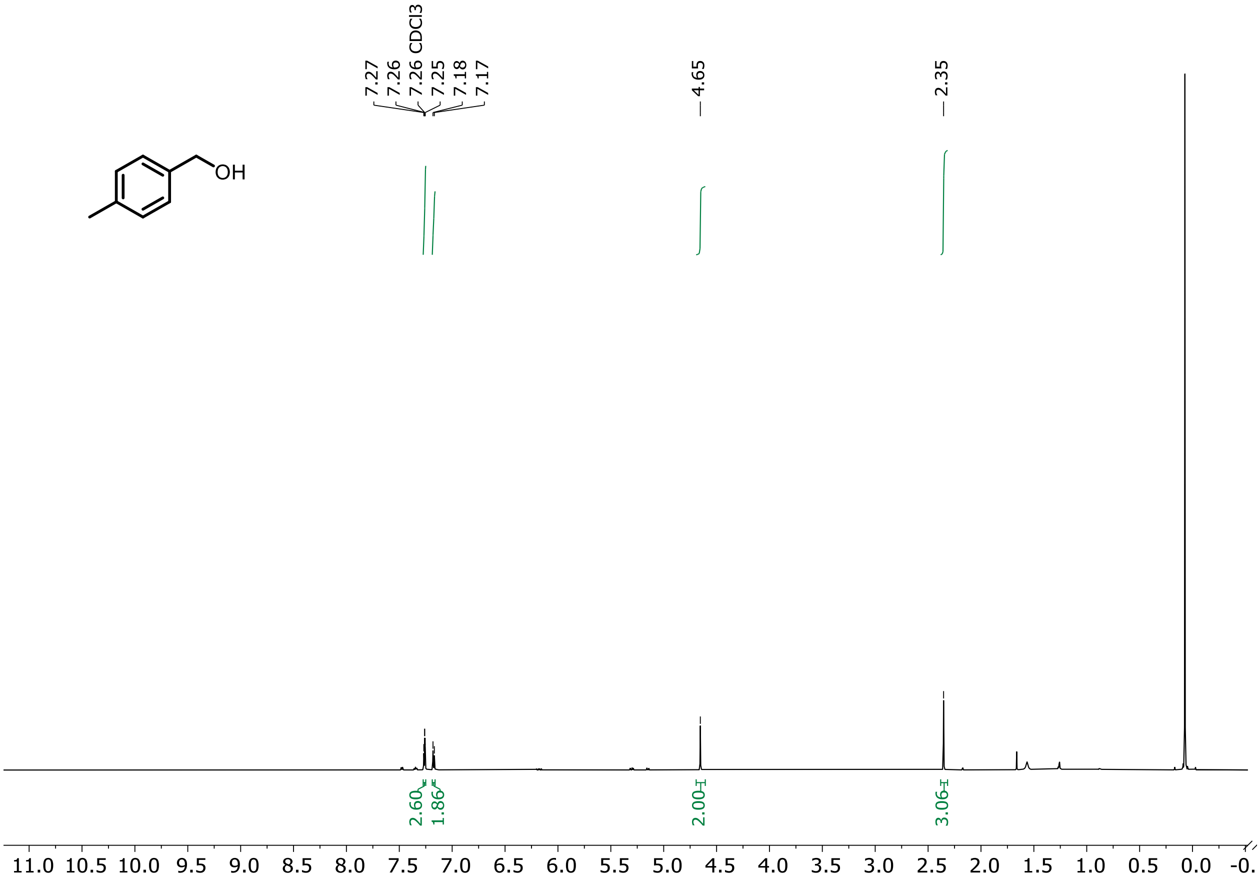

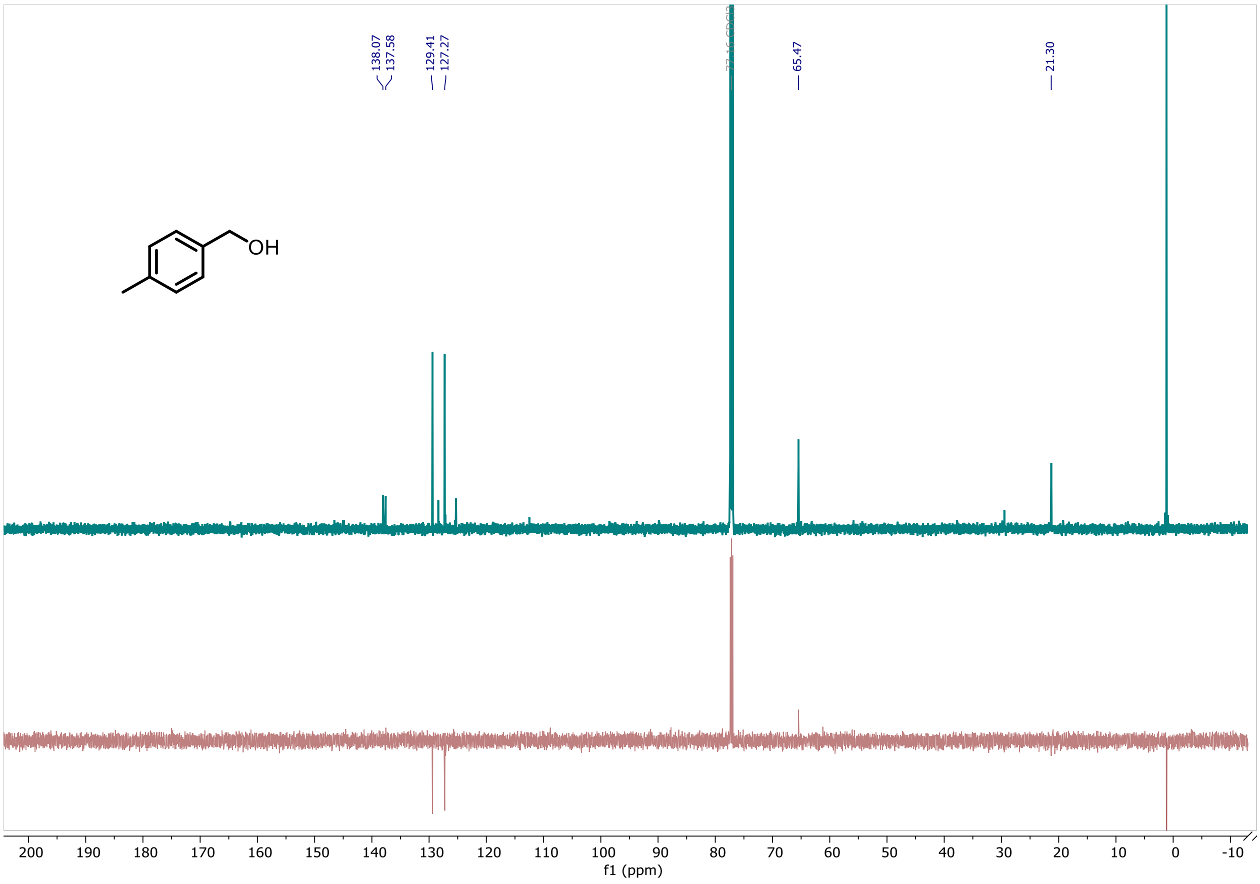

4-chlorobenzyl alcohol (2f)

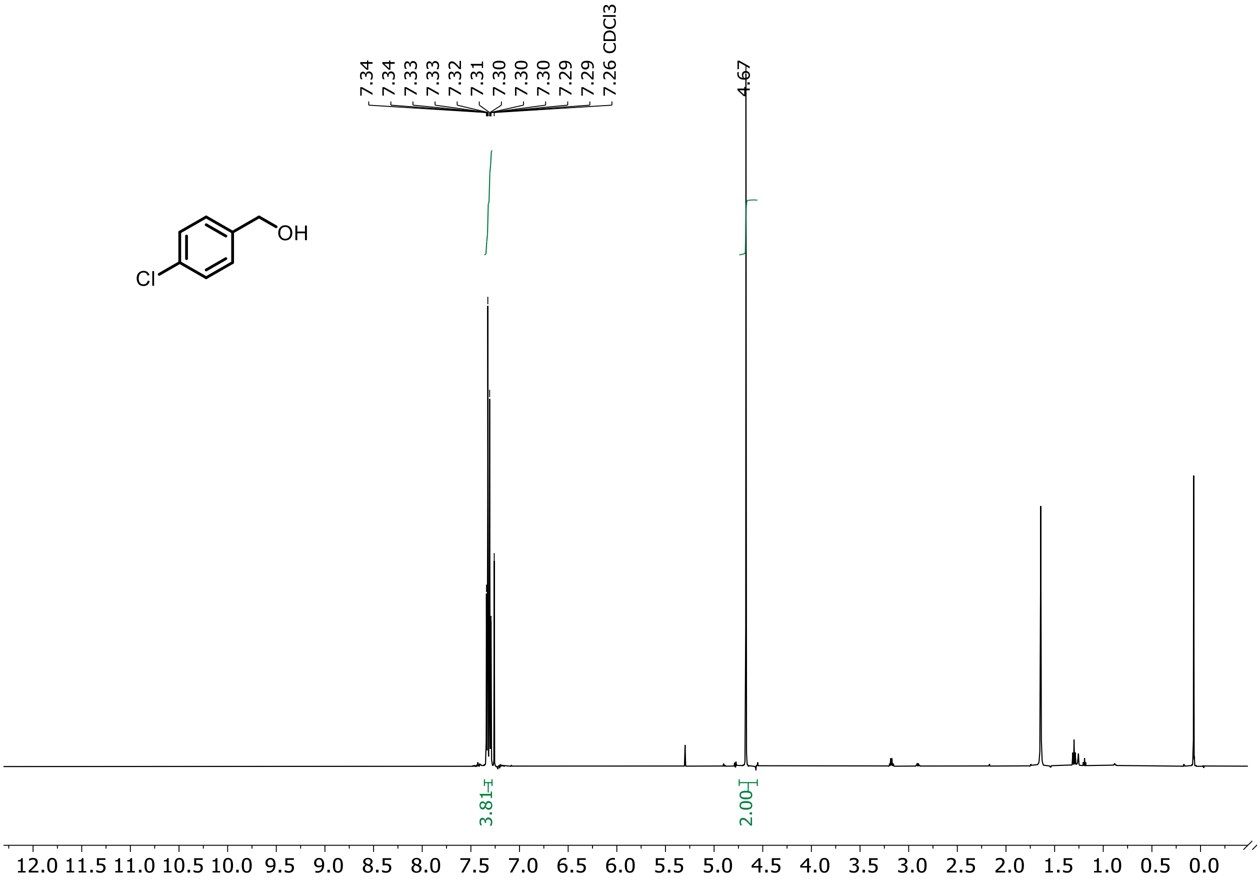

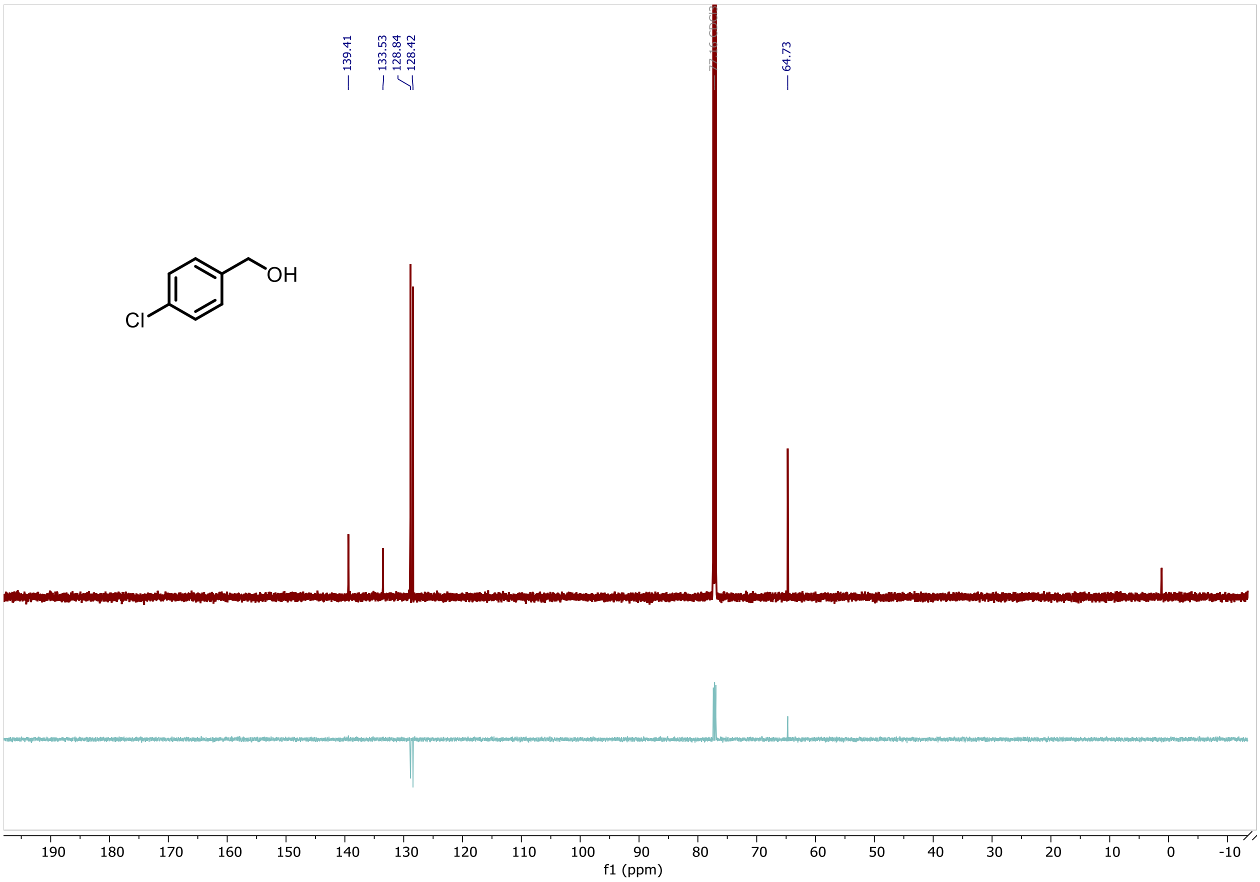

4-methoxybenzyl alcohol (2g)

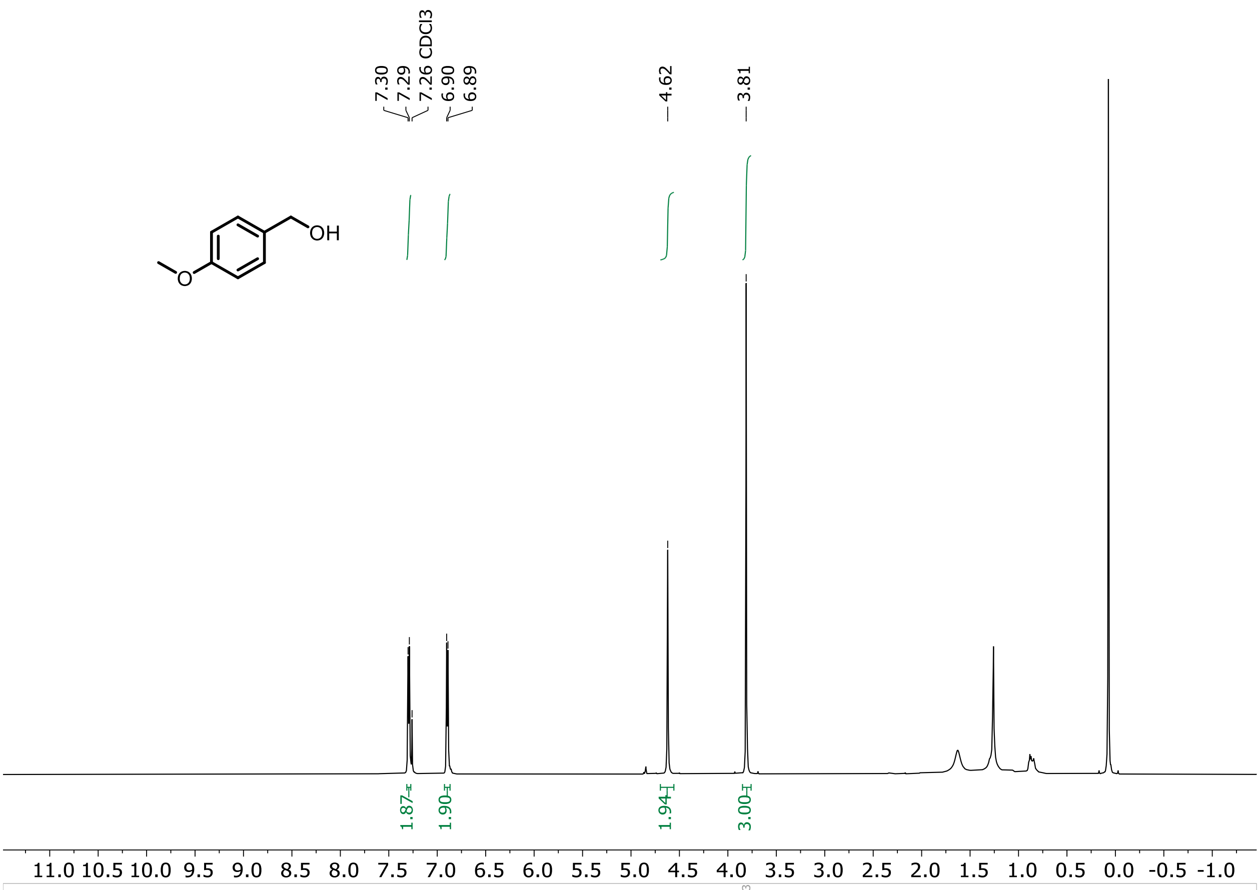

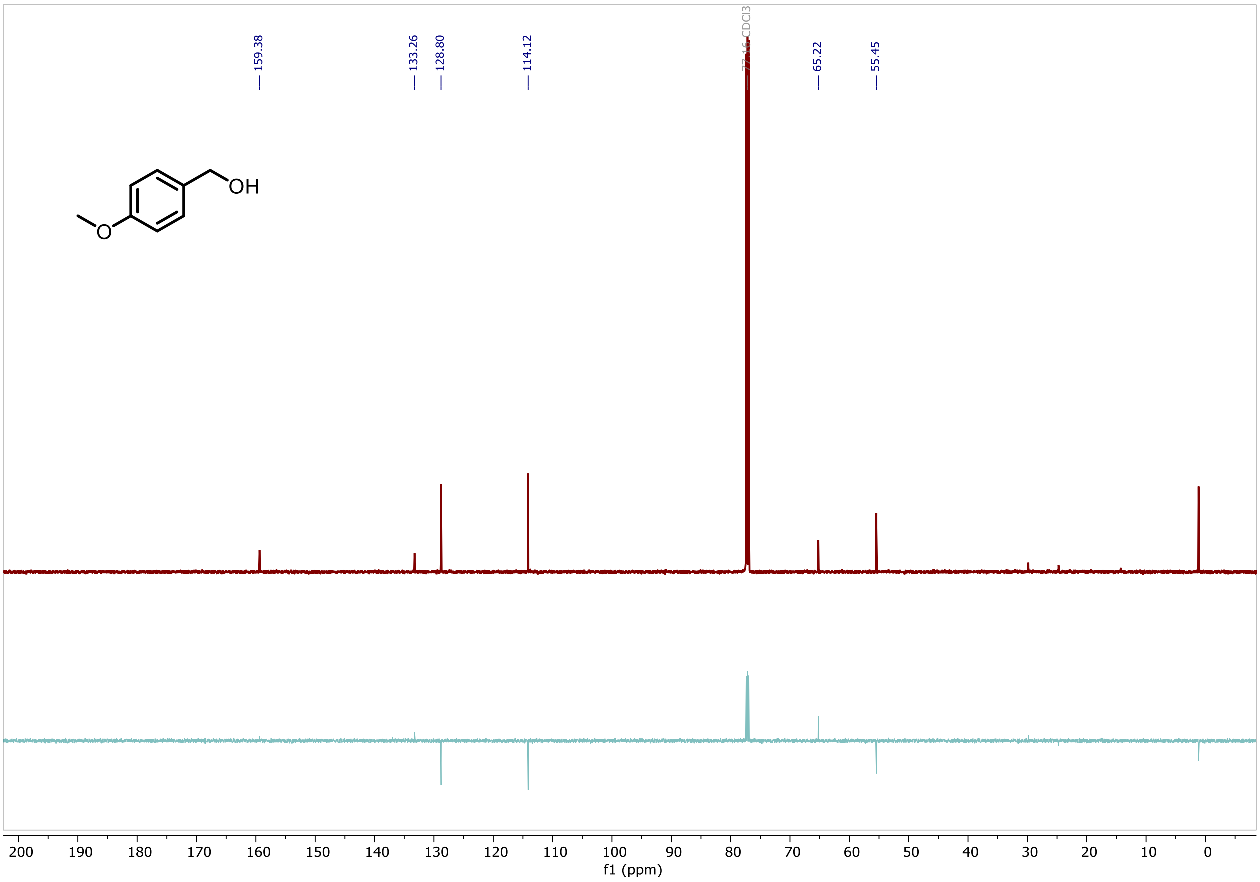

Biphenyl-4-yl-methanol (2h)

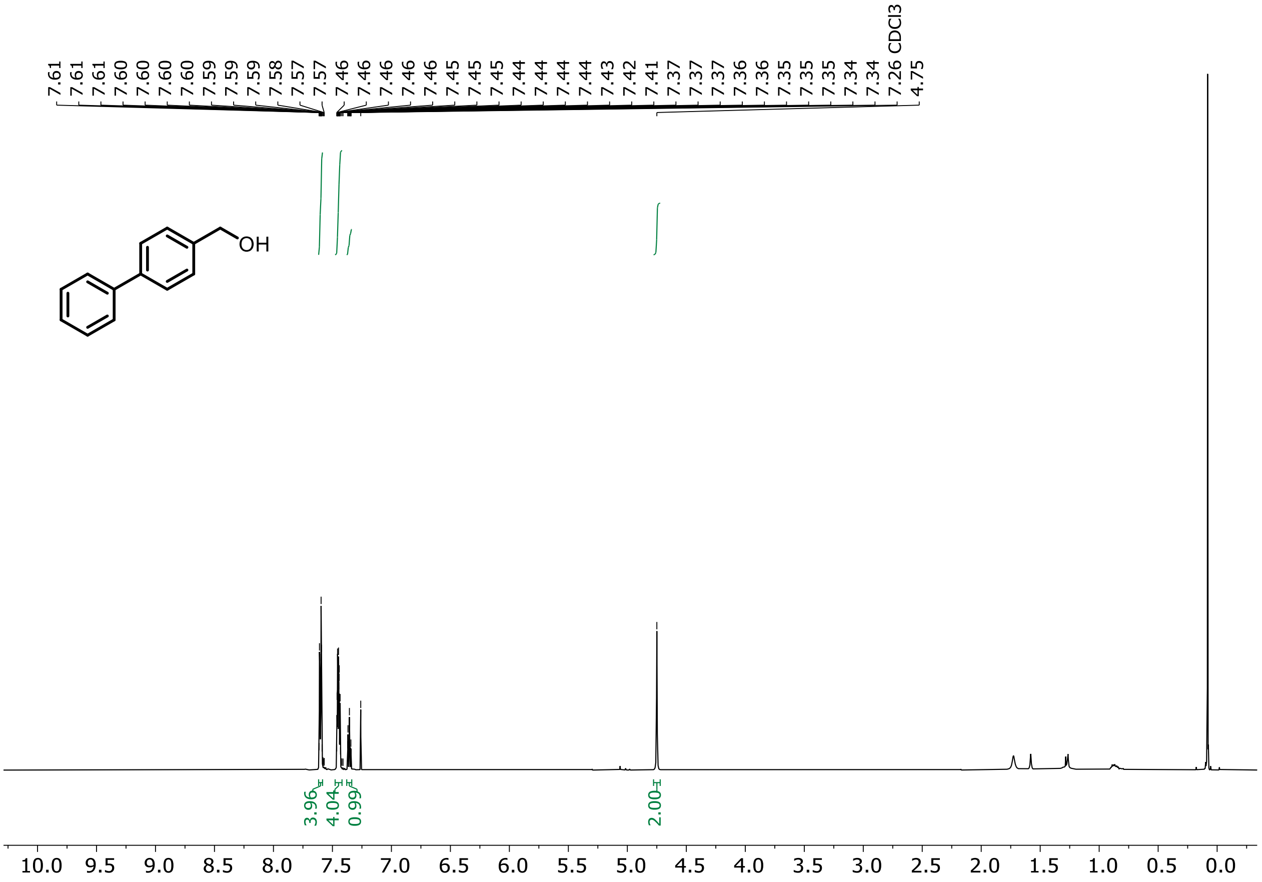

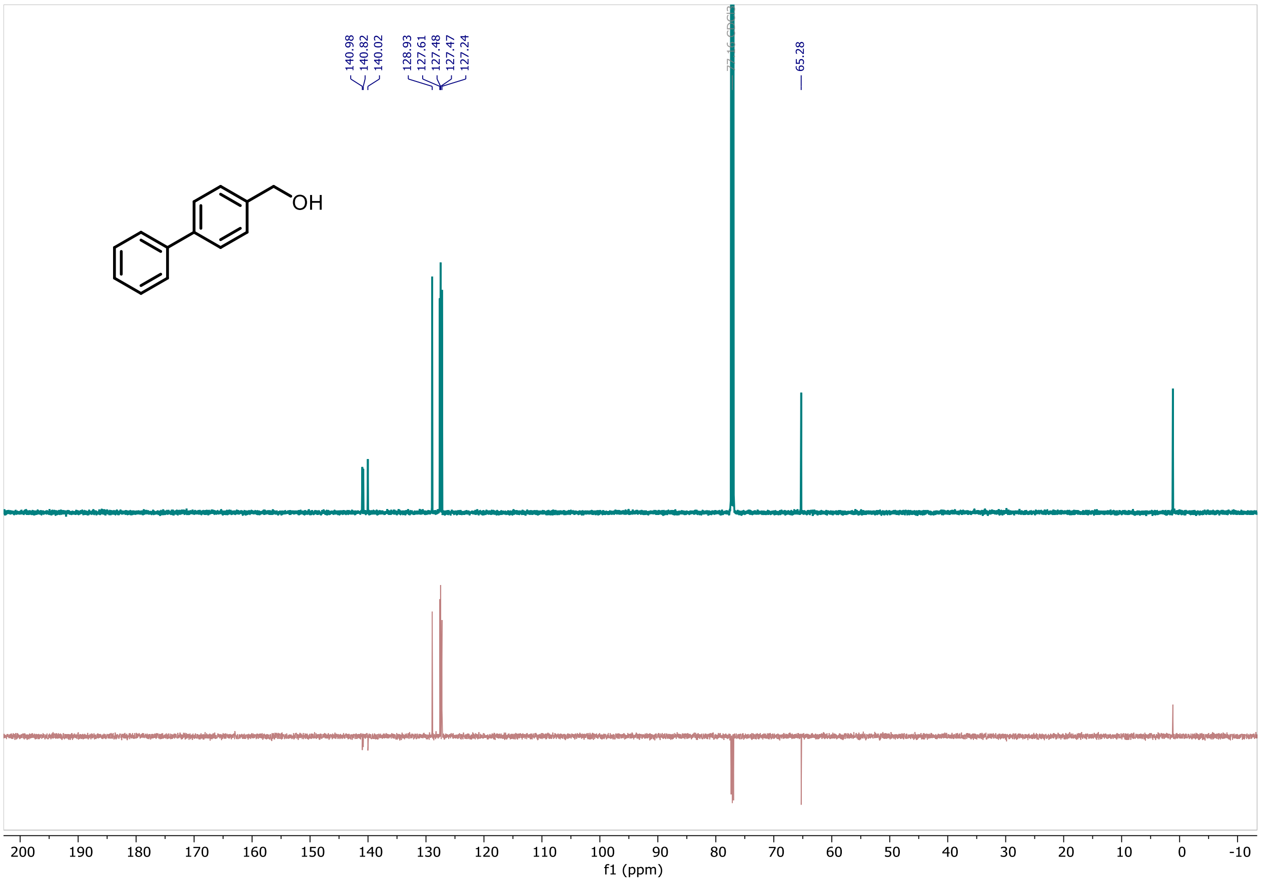

2-((2-methoxybenzyl)oxy)-4,4,5,5-tetramethyl-1,3,2-dioxaborolane (2k)

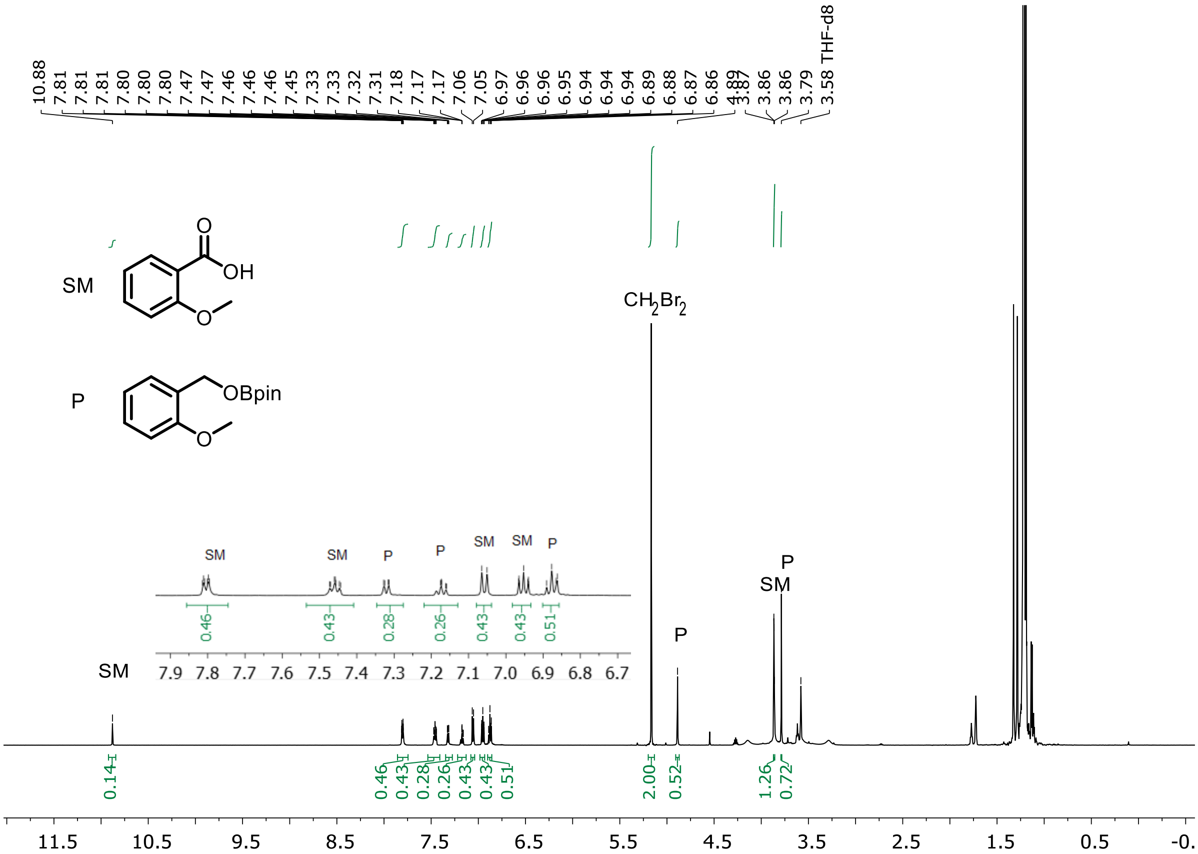

(3E)-4-phenyl-3-buten-1-ol (2l)

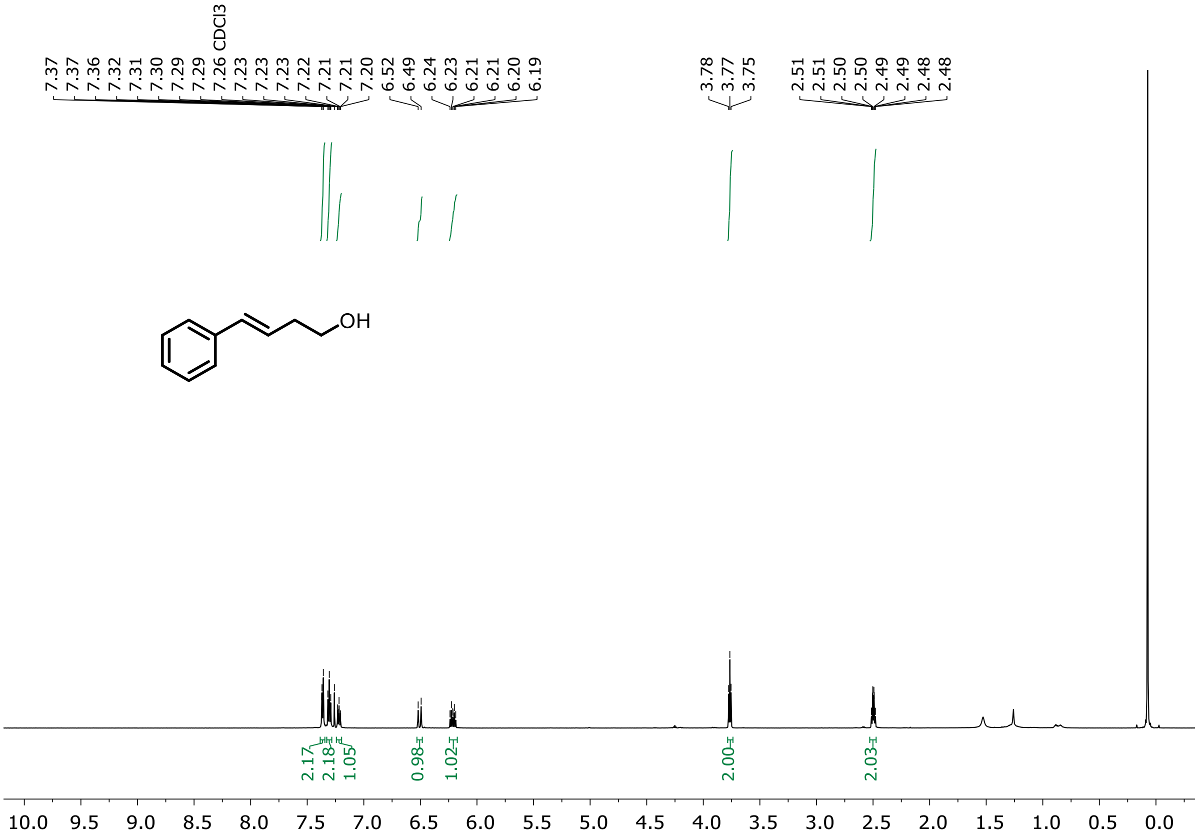

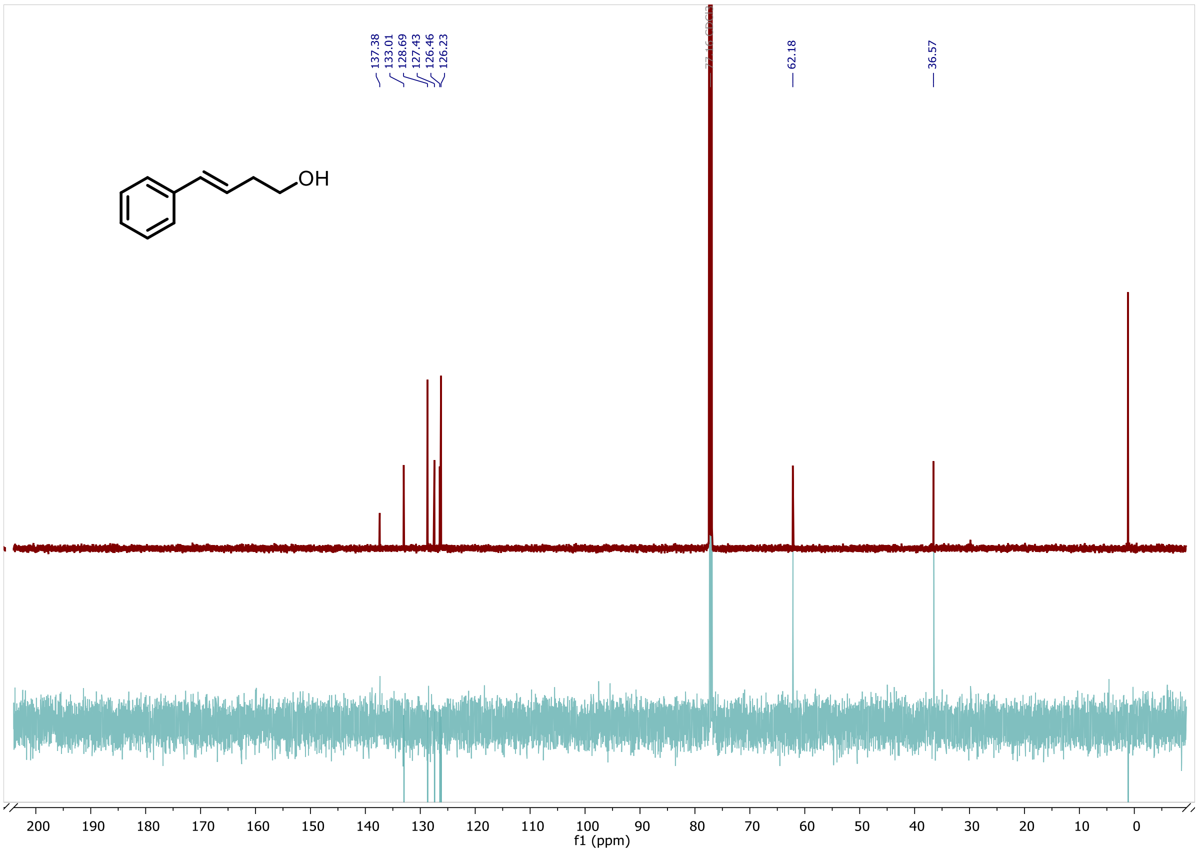

Ethyl 3-(3-hydroxypropyl) benzoate (2m)

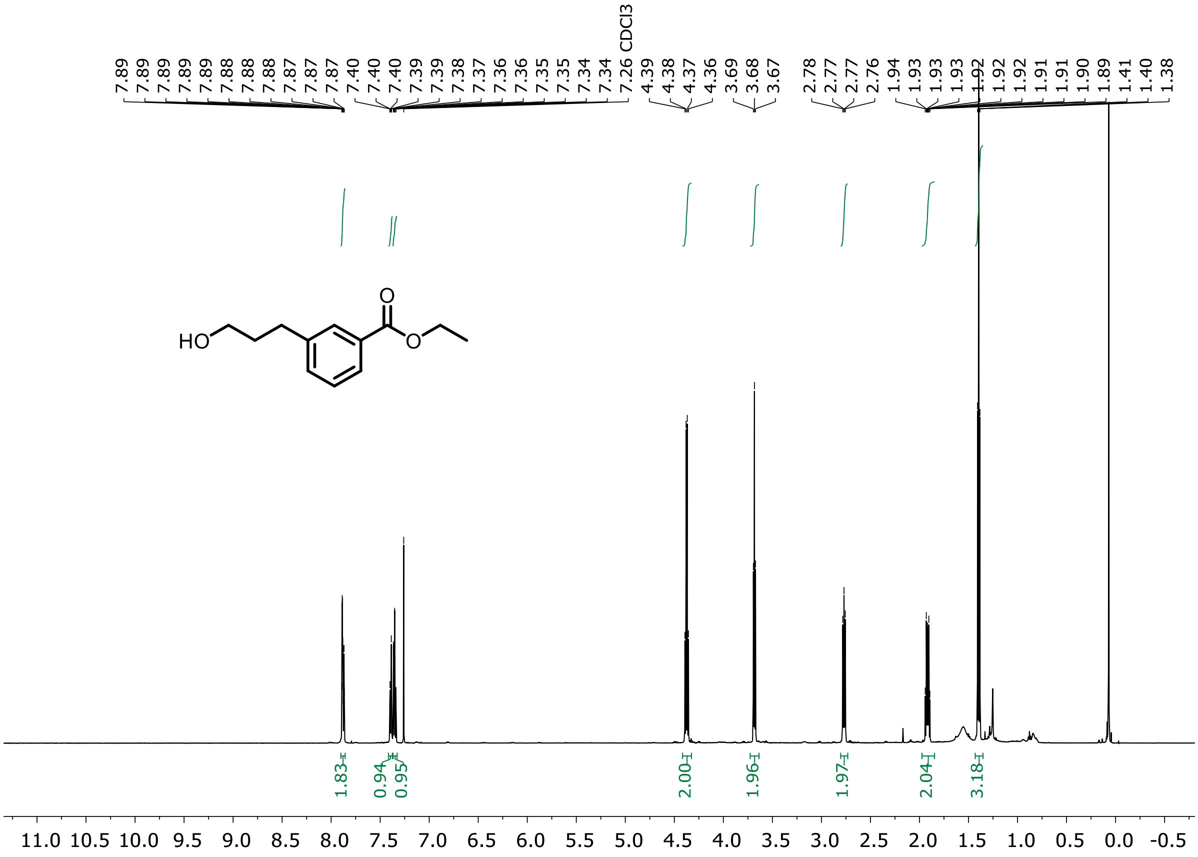

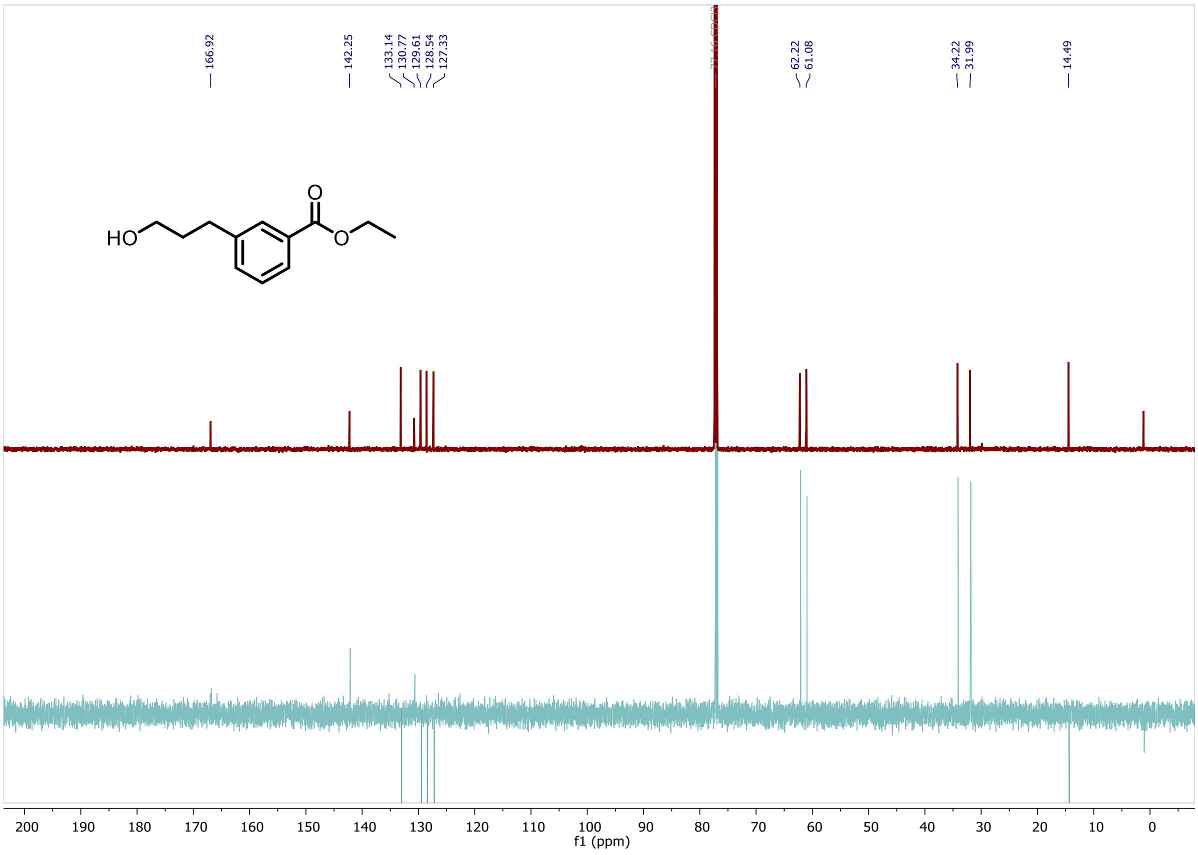

3-(4-nitrophenyl)-1-propanol (2n)

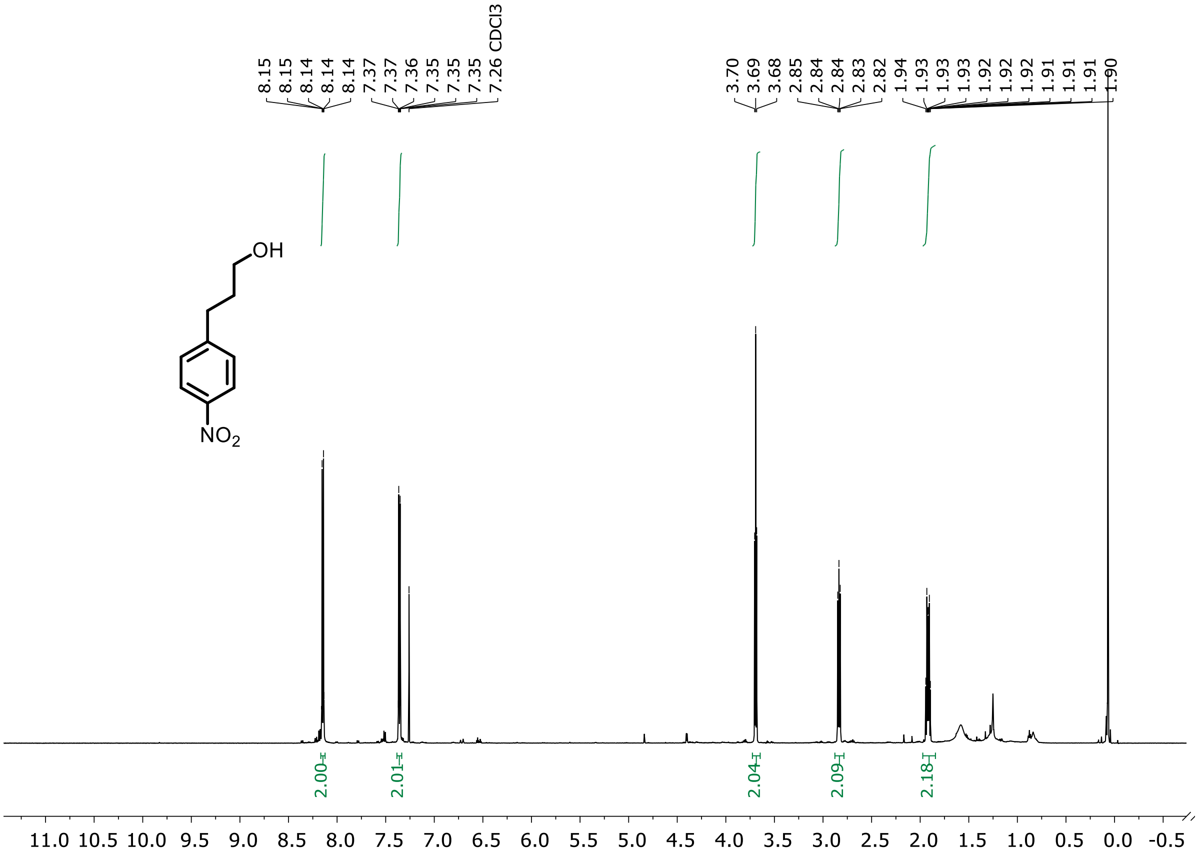

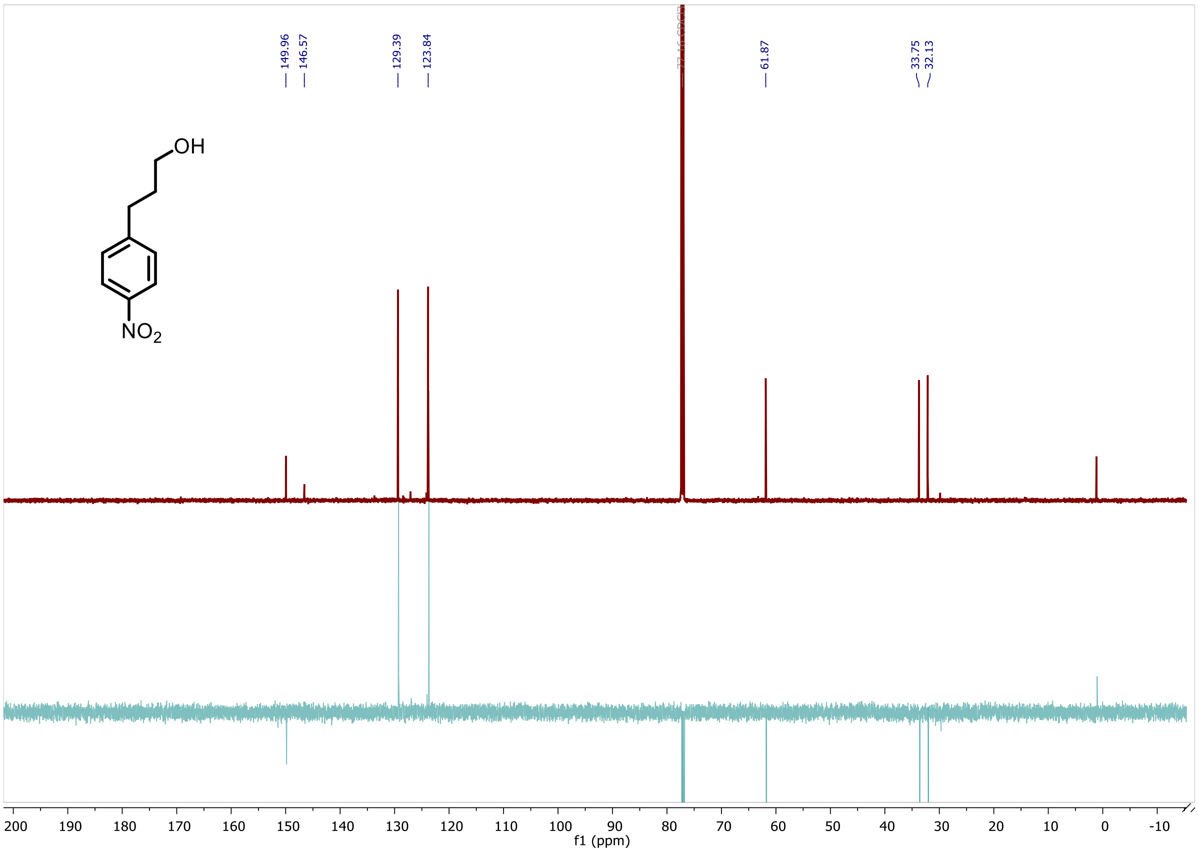

1-bromo-6-hexanol (2o)

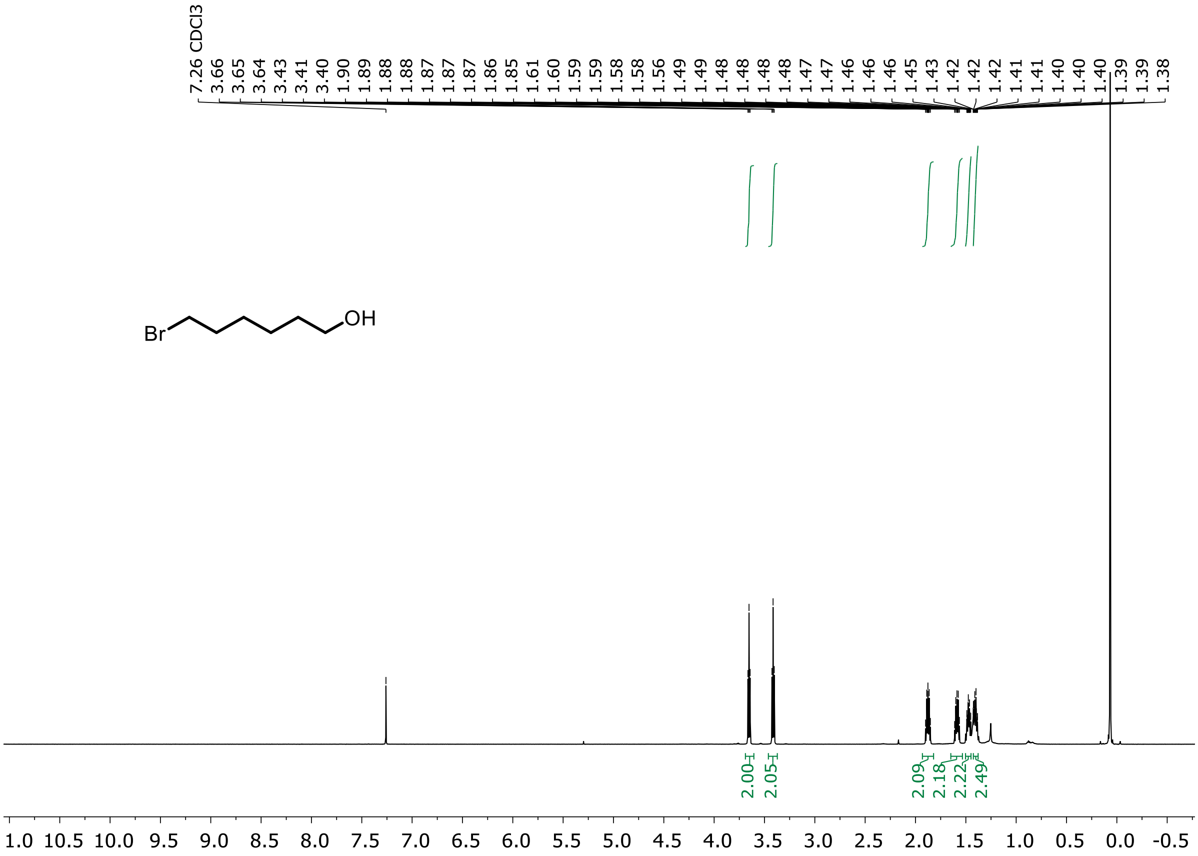

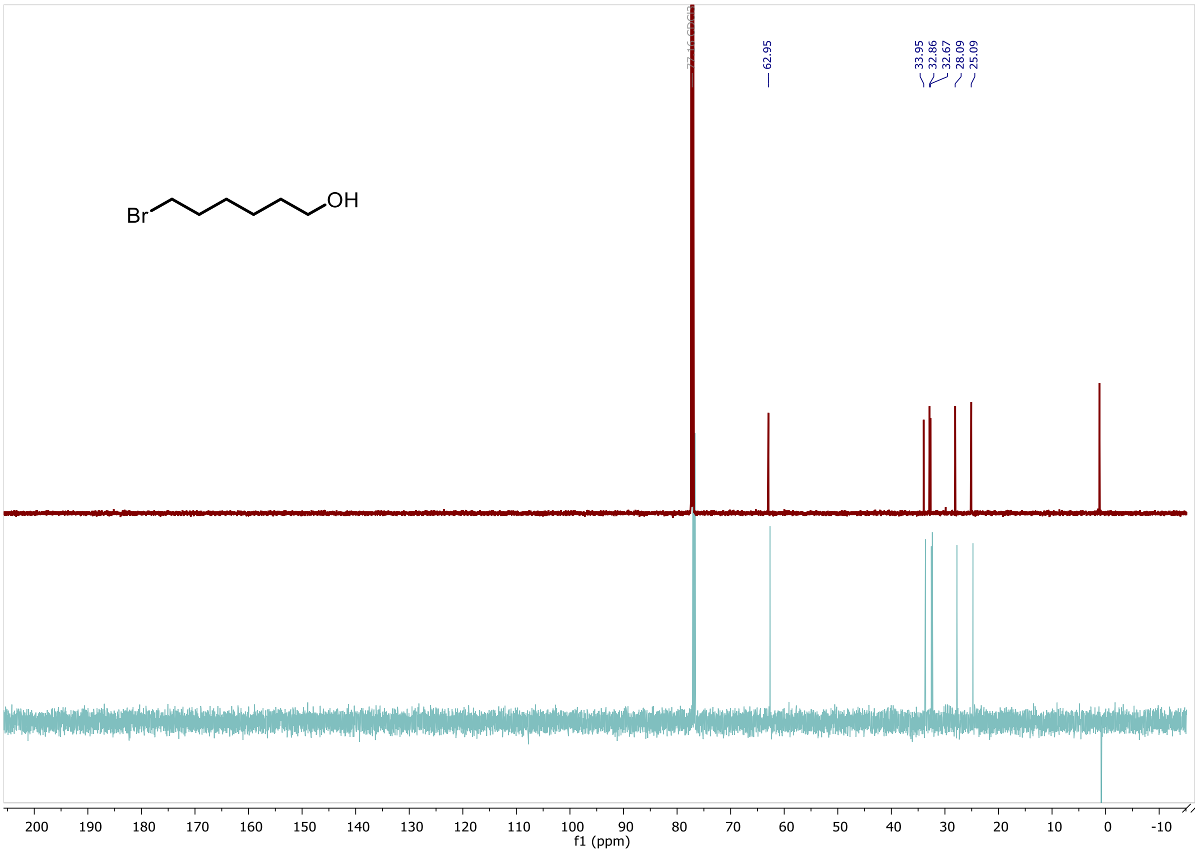

4-(3-Hydroxypropyl) benzonitrile (2p)

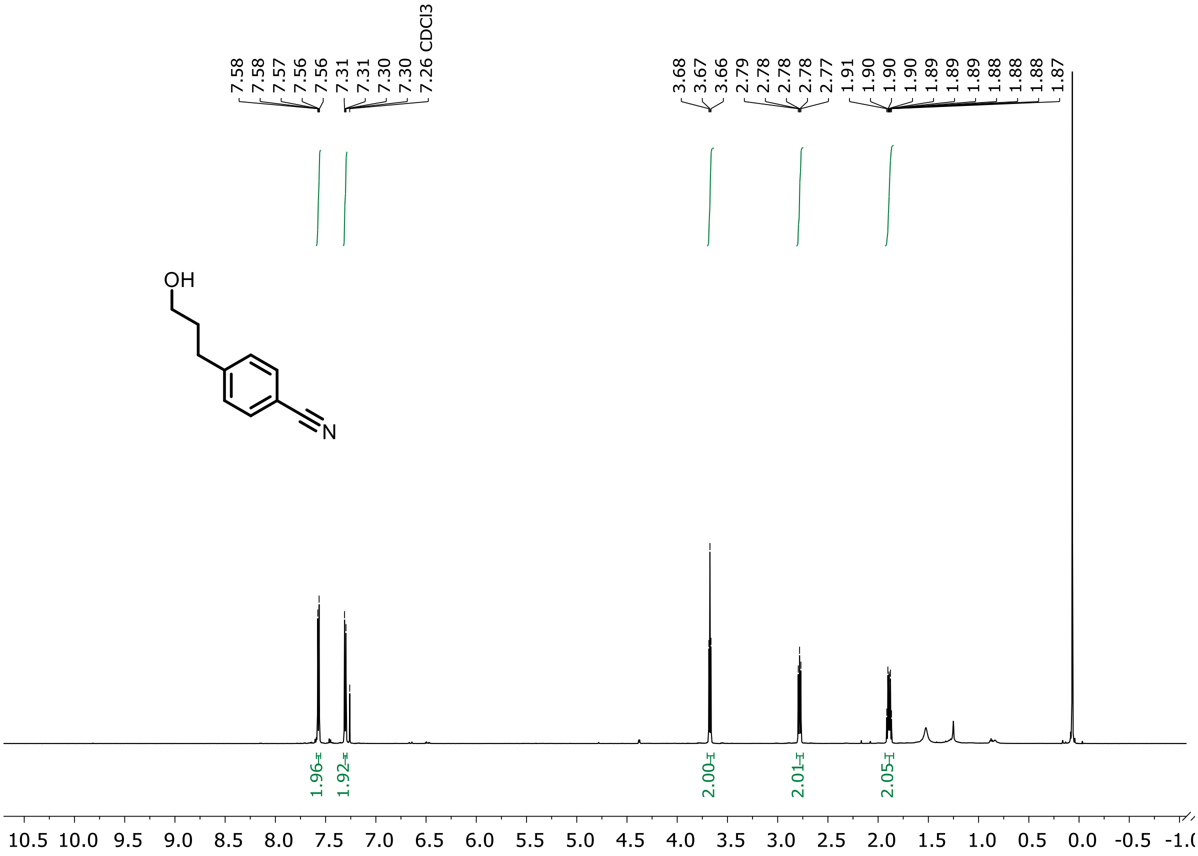

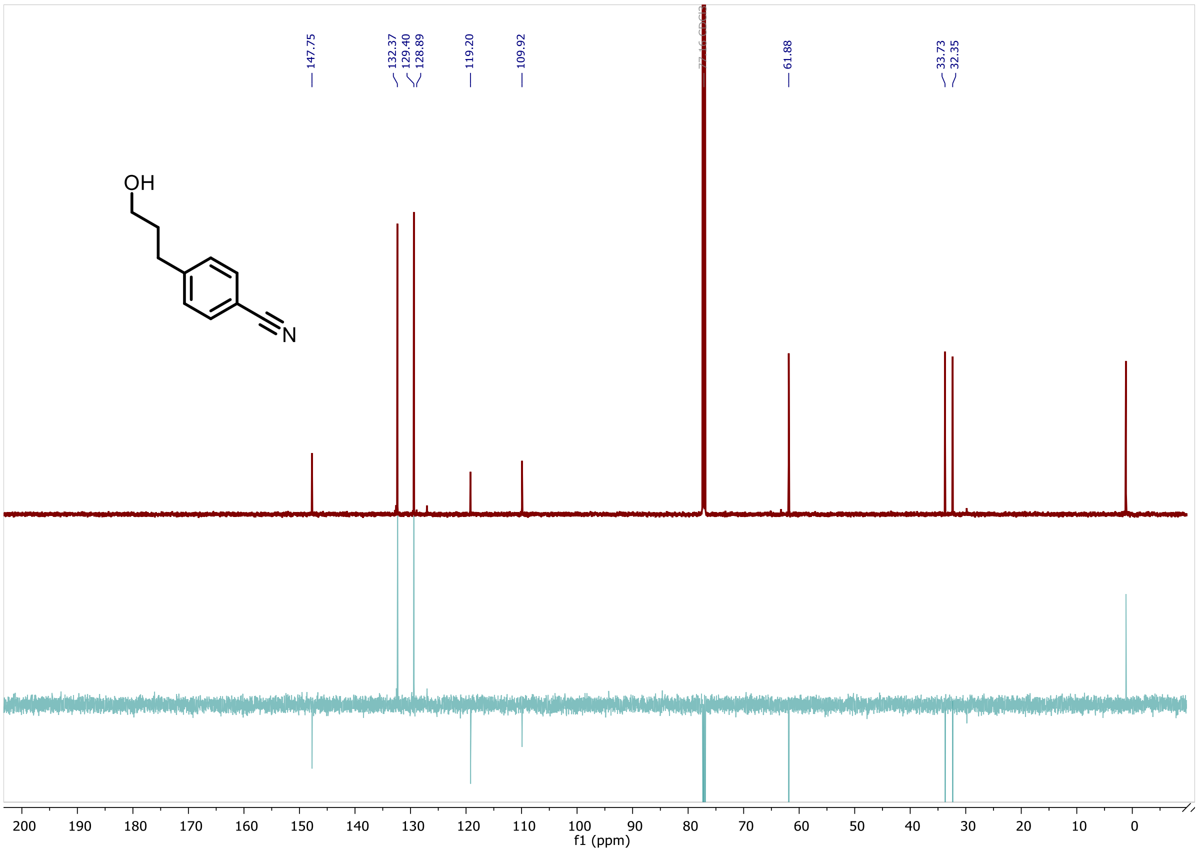

4-((4,4,5,5-tetramethyl-1,3,2-dioxaborolan-2-yl)oxy)butanenitrile (2q)

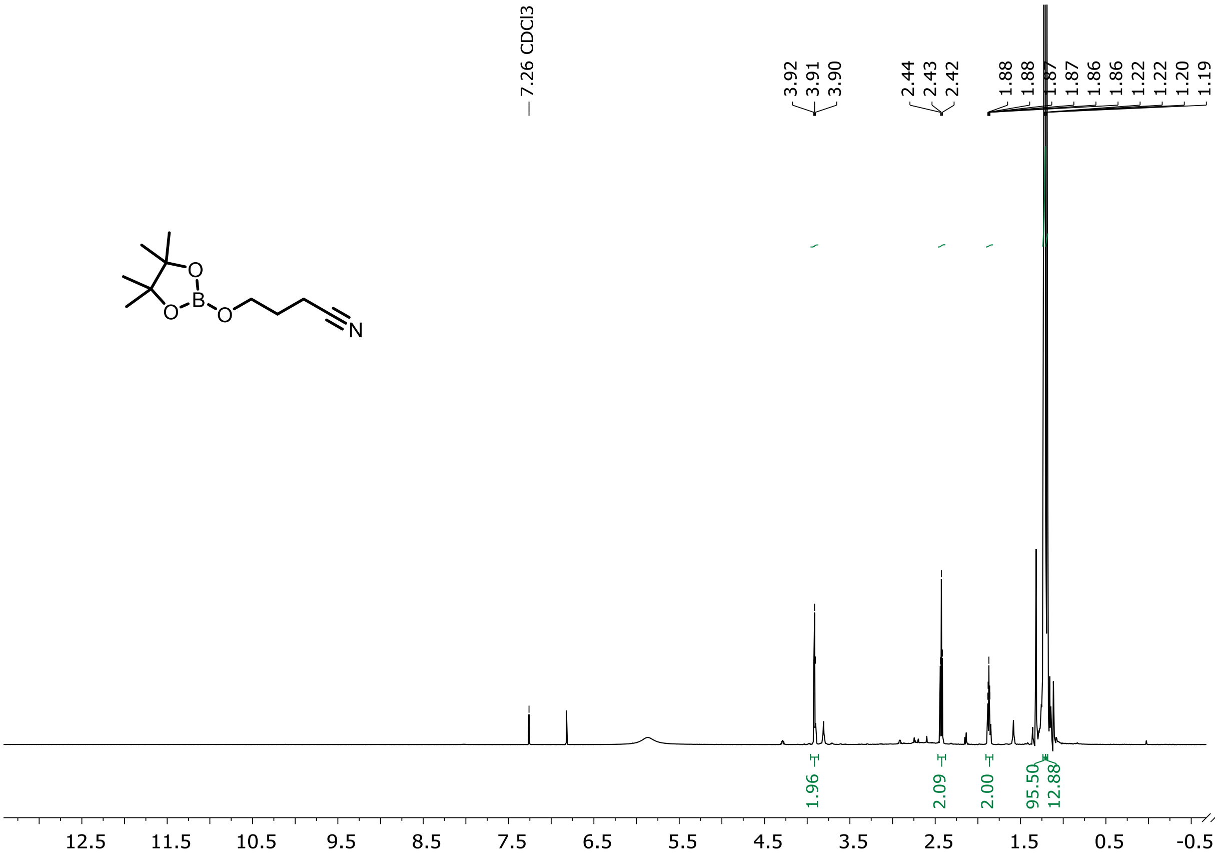

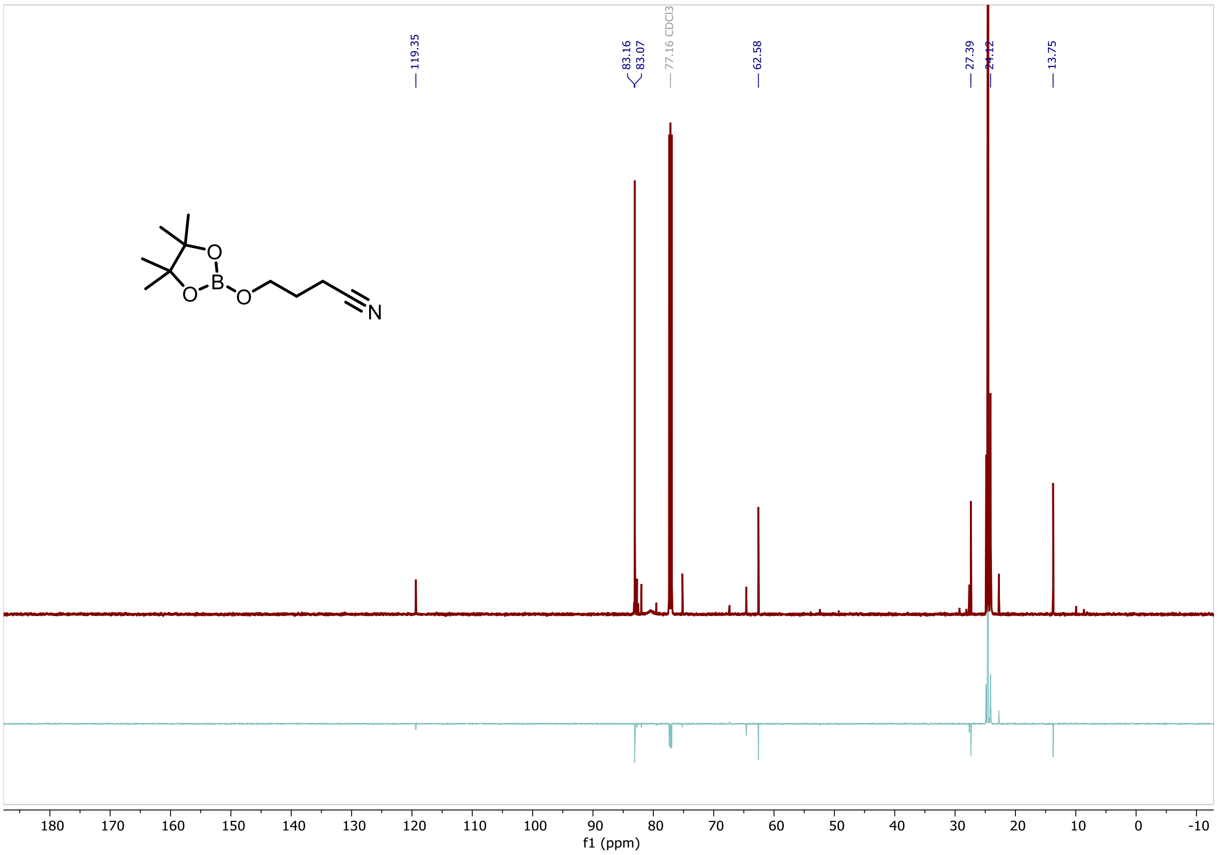

N-(3-hydroxypropyl) benzamide (2r)

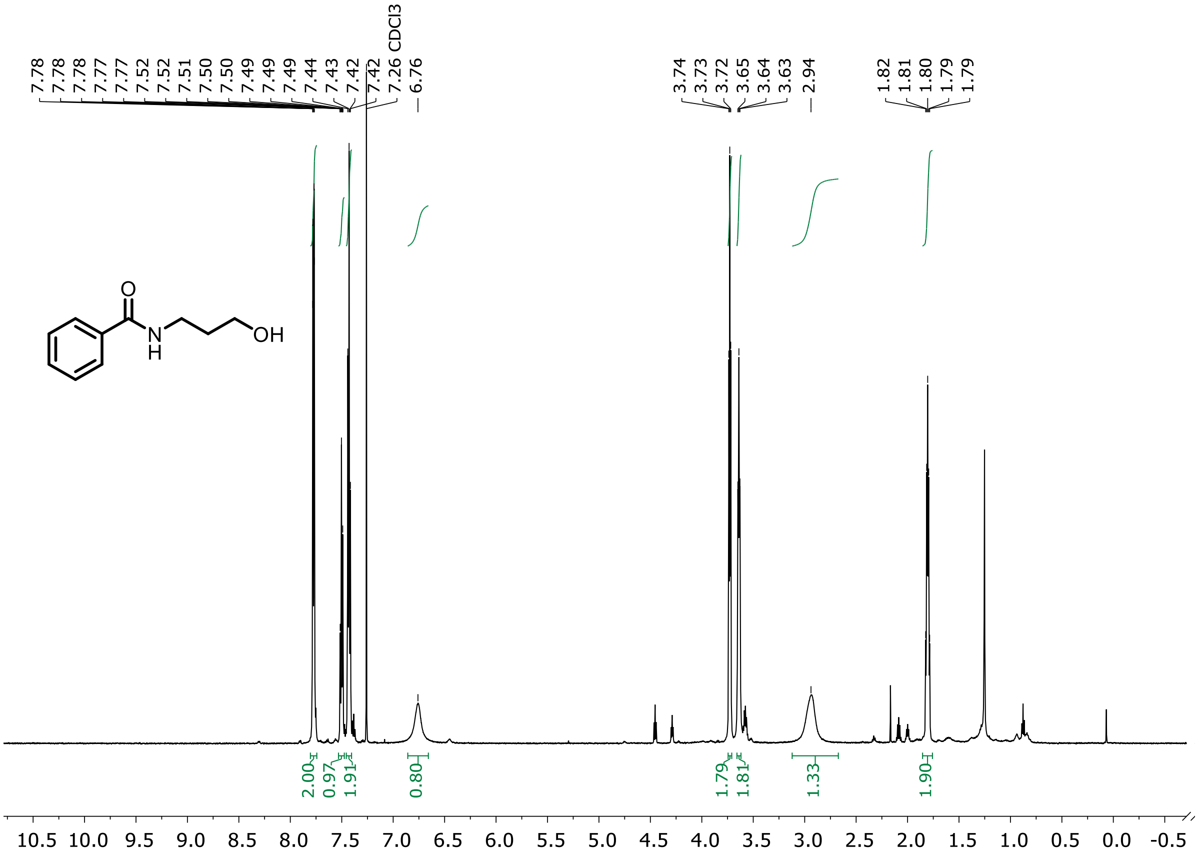

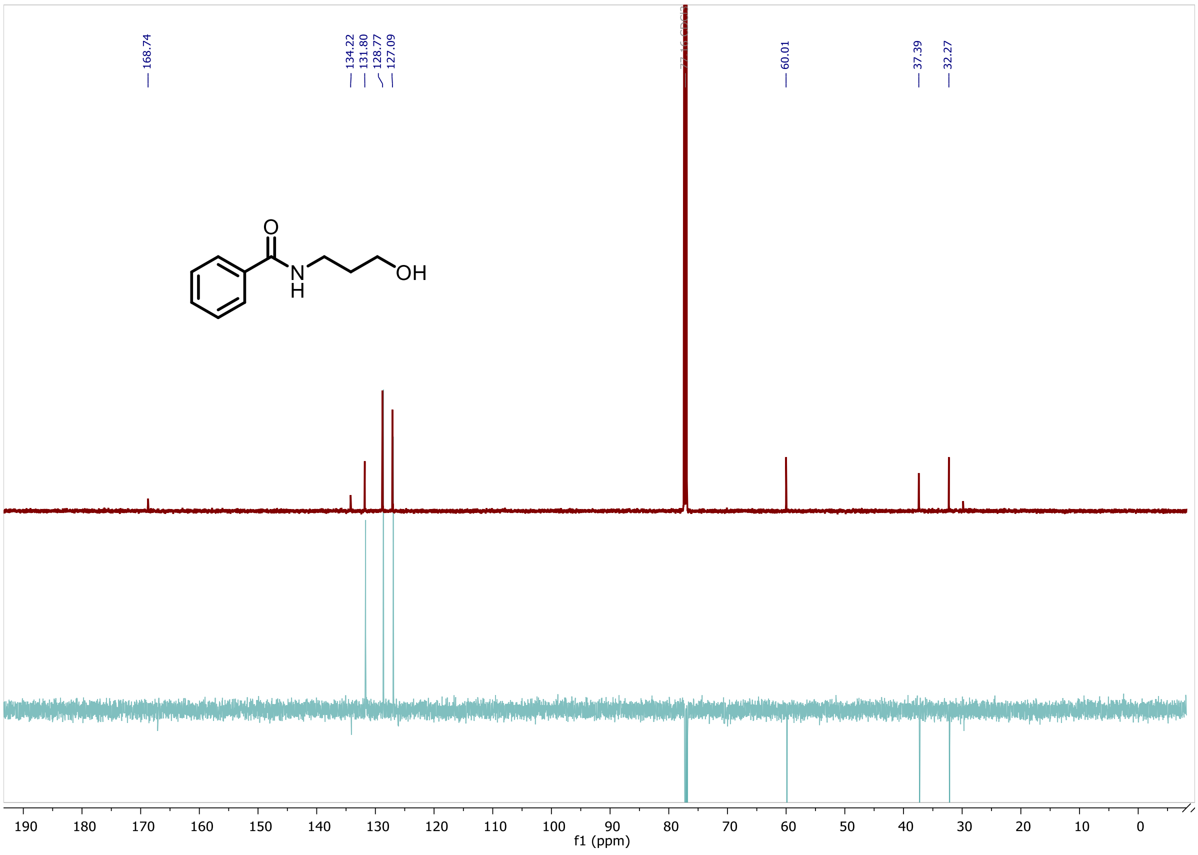

Benzyl(7-hydroxyheptyl)carbamate (2s) and benzyl(7-((4,4,5,5(--tetramethyl-1,2,3-dioxaborolan-2-yl)oxy)heptyl) carbamate (2s')

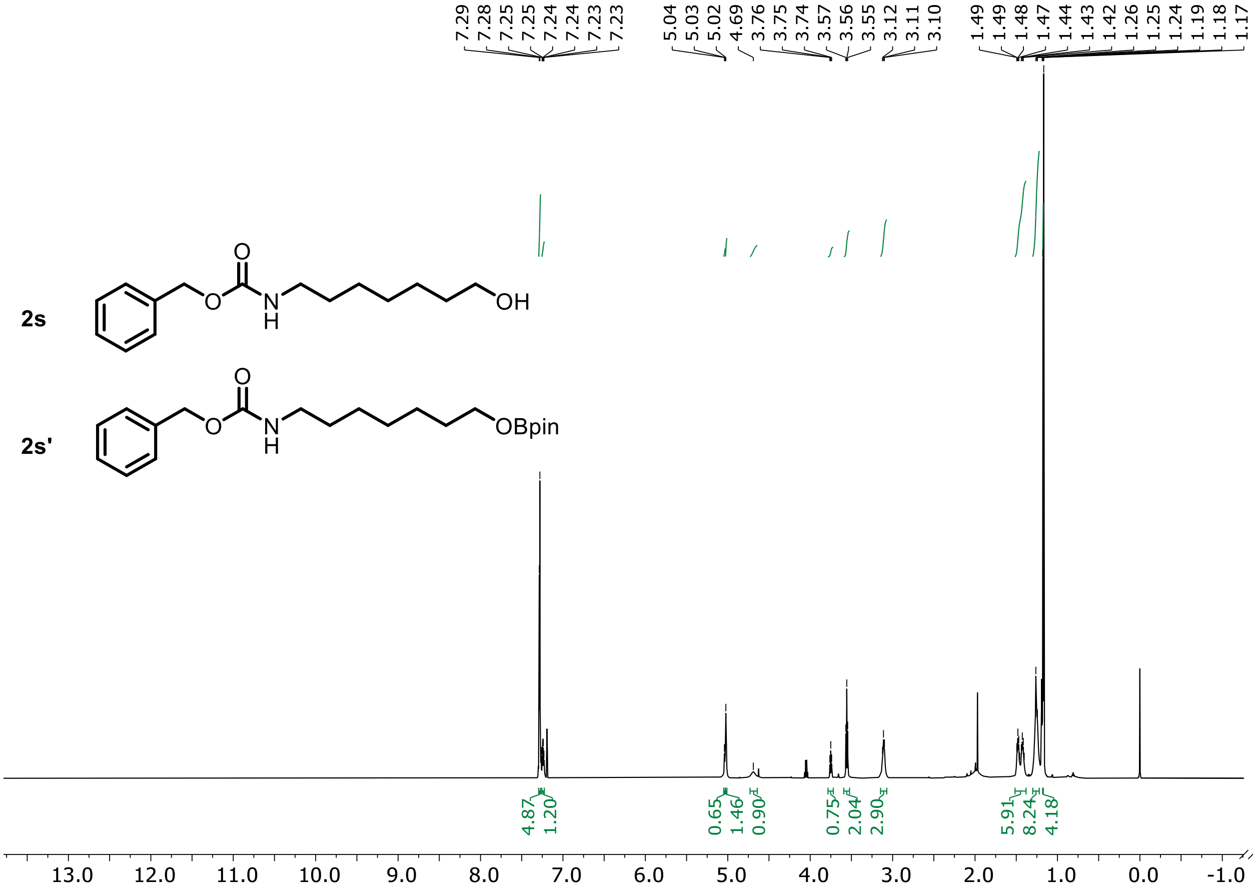

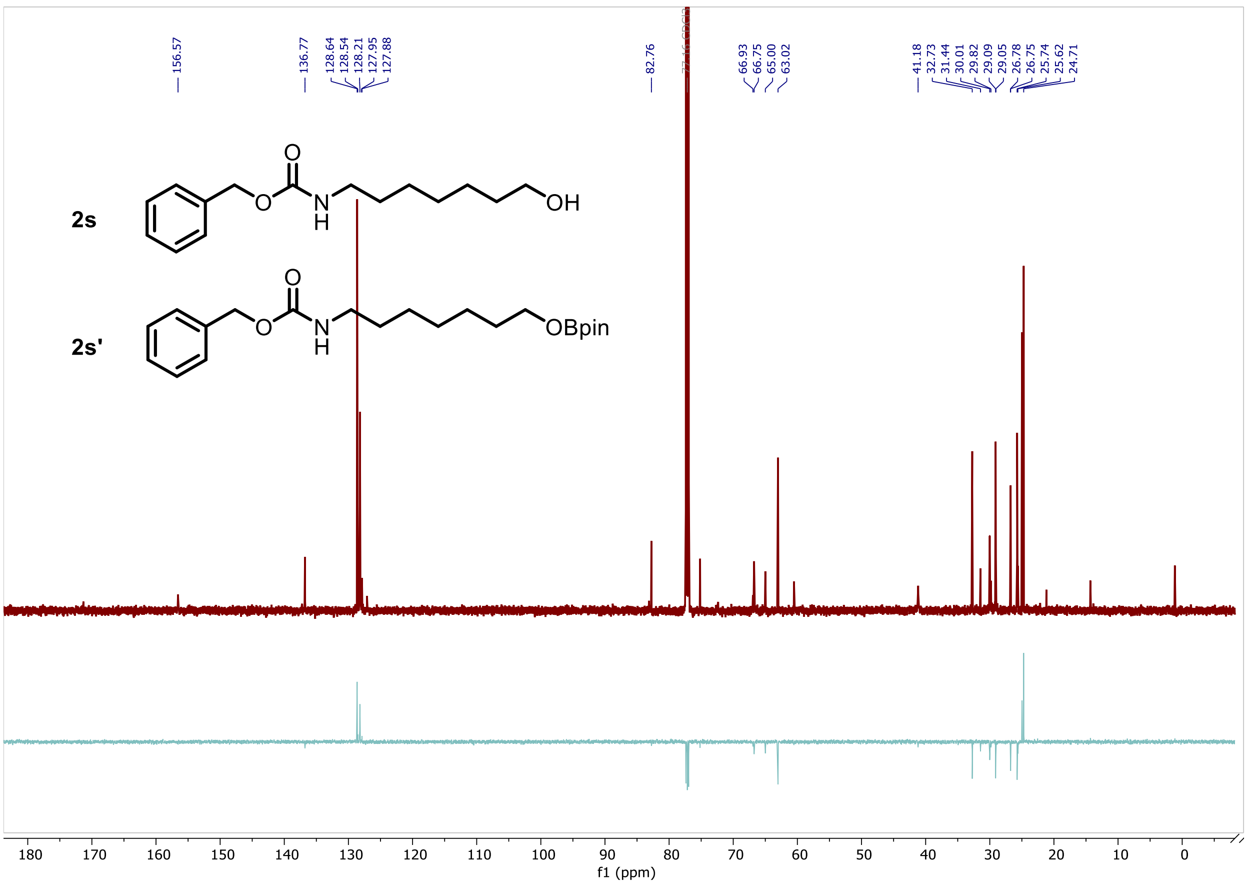

2-(6-methoxy-2-naphthyl)-propyl alcohol (2t)

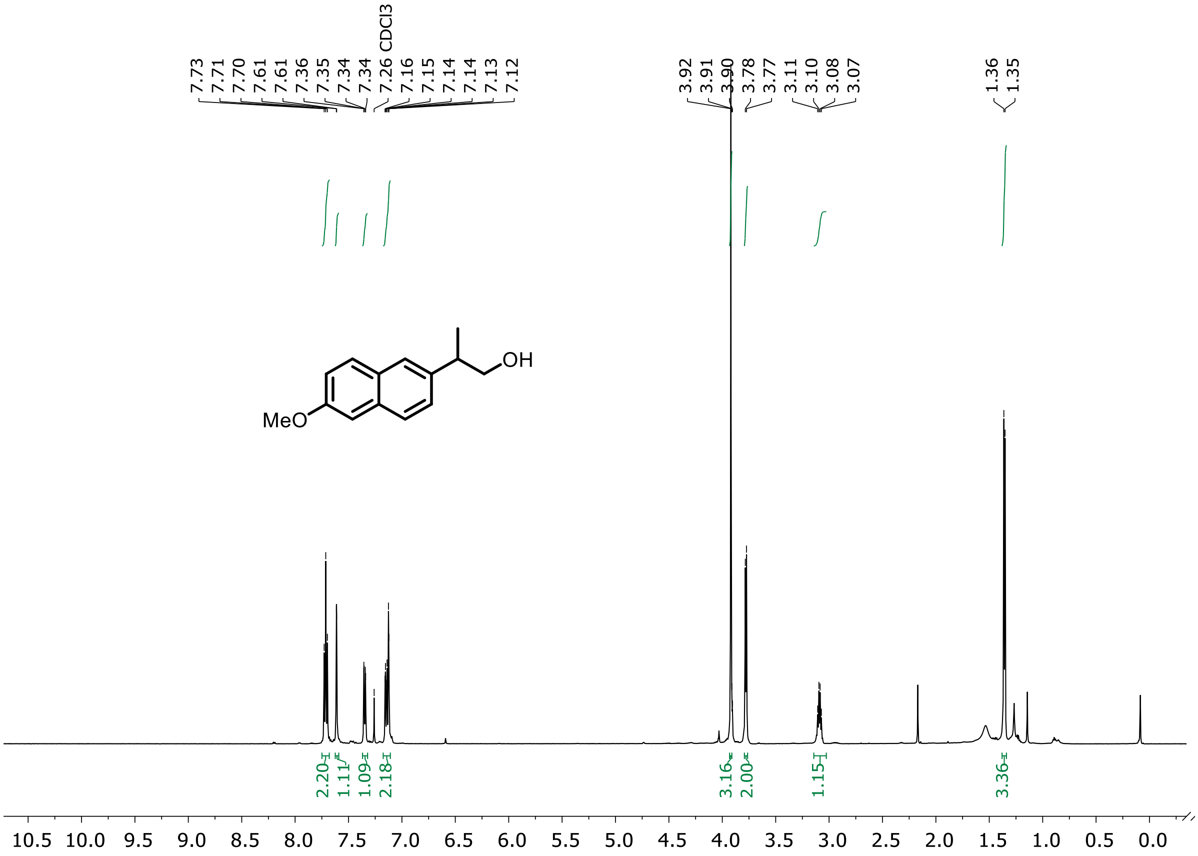

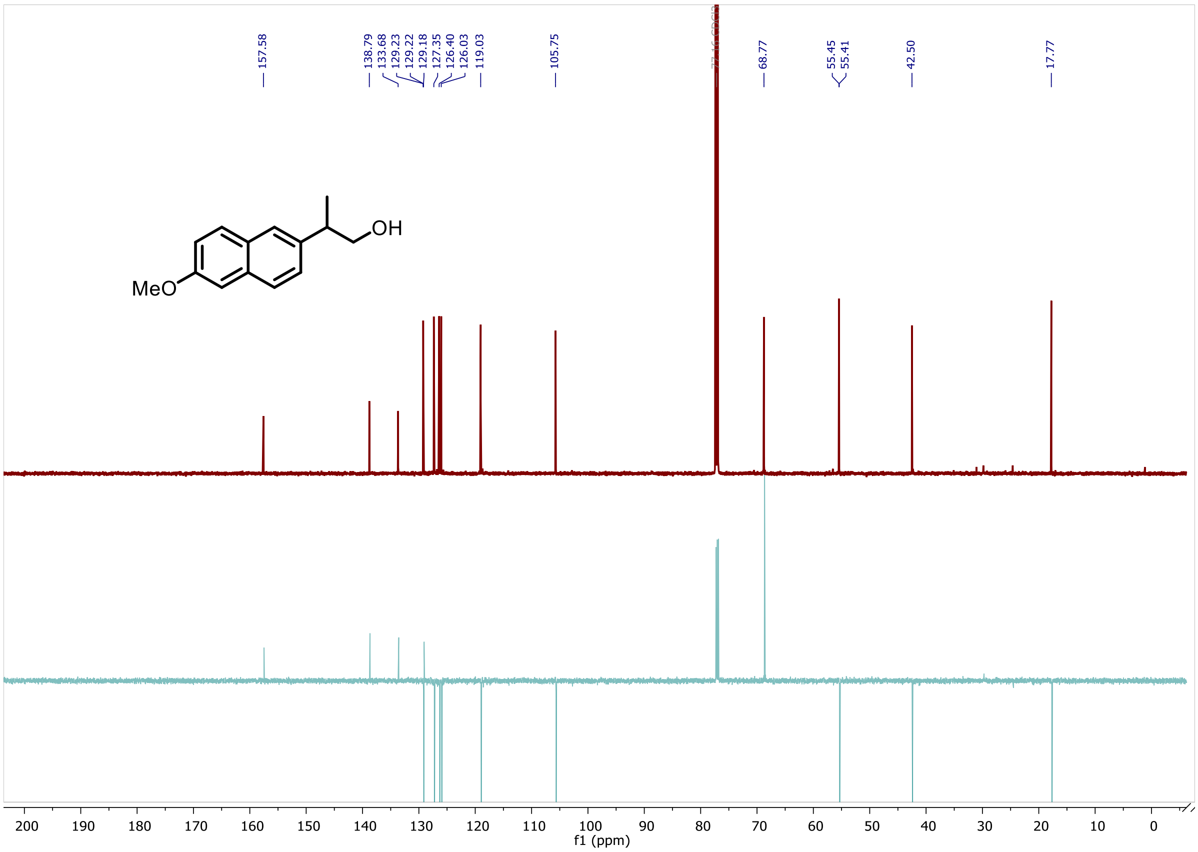

2-(3-benzoylphenyl)-propanol (2u)

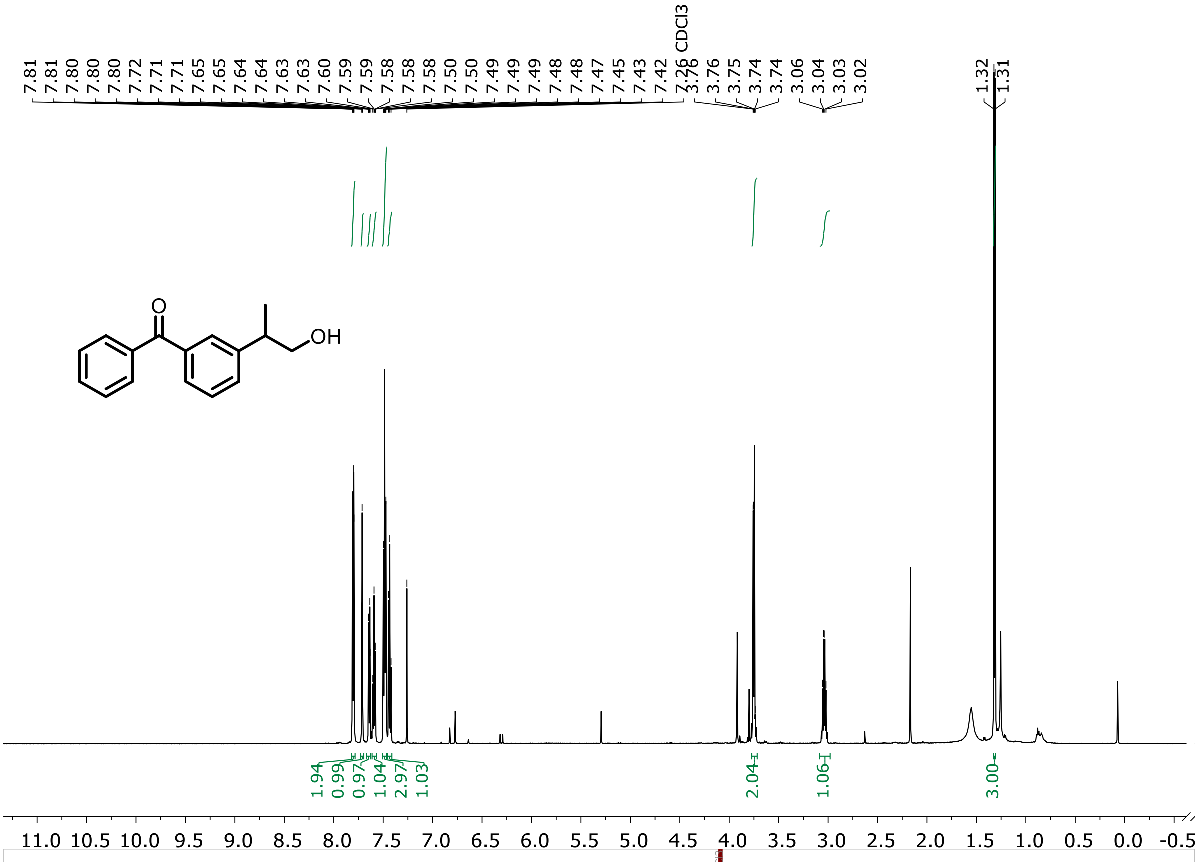

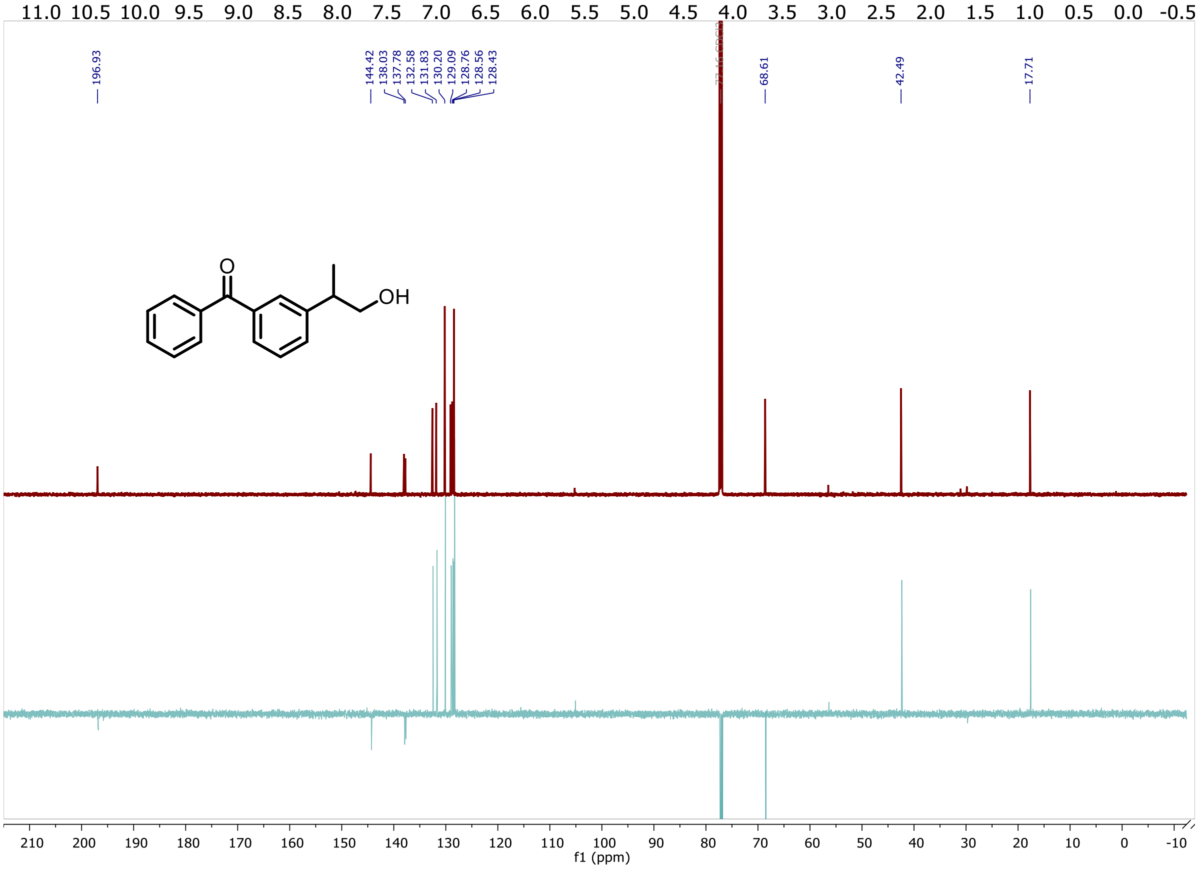

(4-chlorophenyl)(3-(2-hydroxyethyl)-5-methoxy-2-methyl-1H-indol-1-yl)methanone (2v)

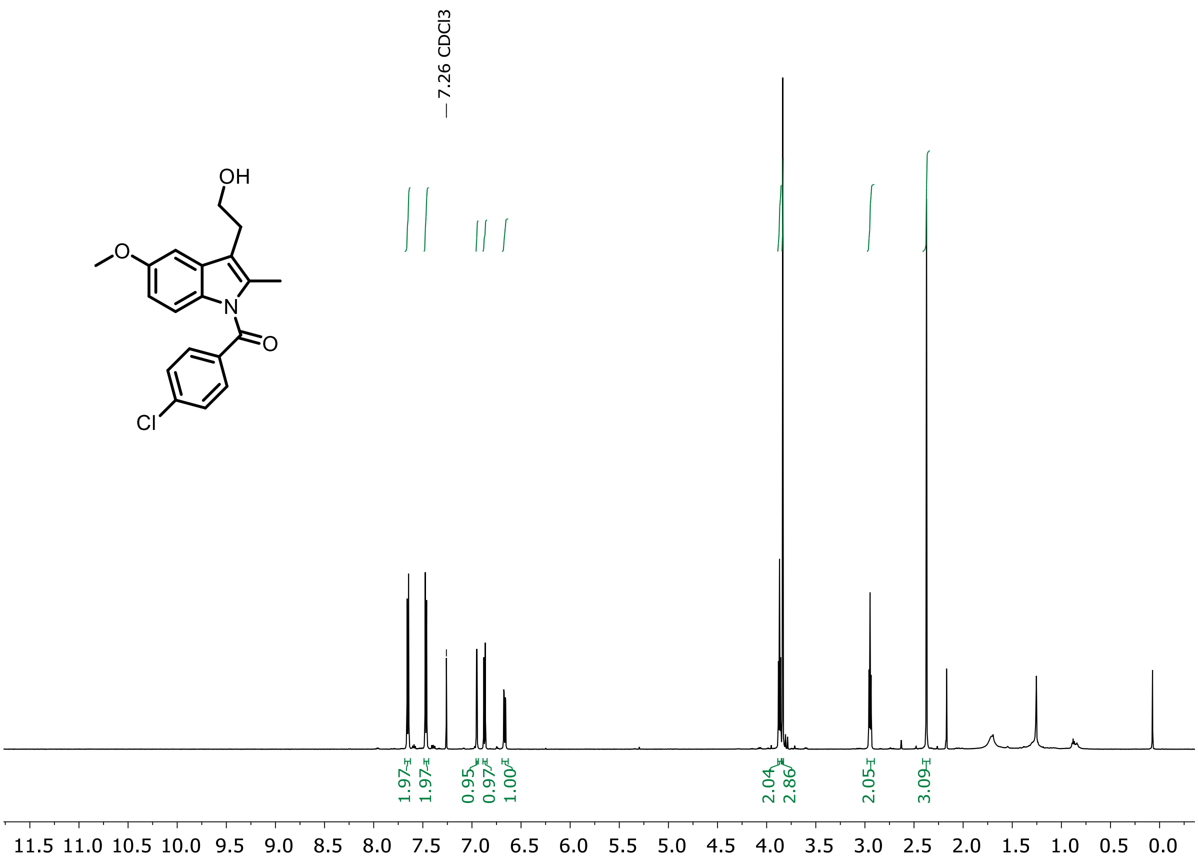

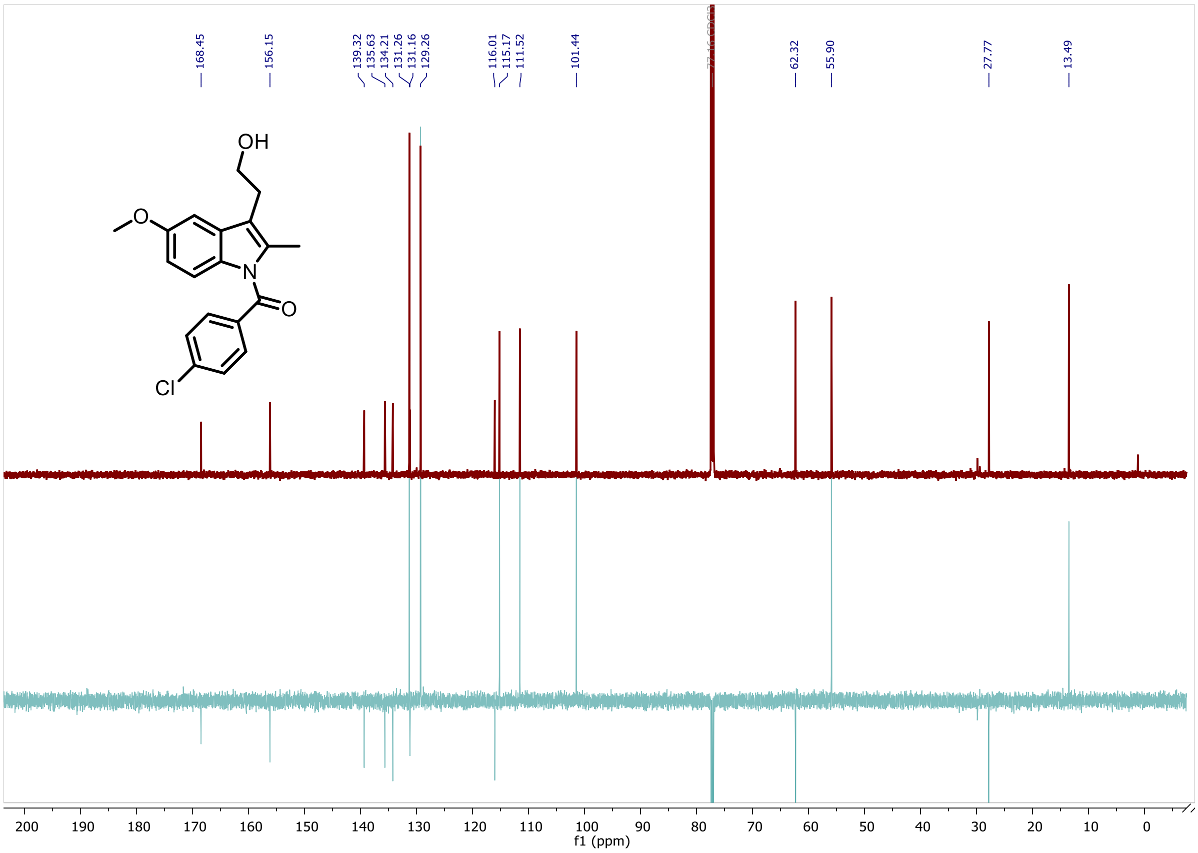

2-(6,11-dihydro-11-oxodibanzoxepin-2-yl) ethanol (2w)

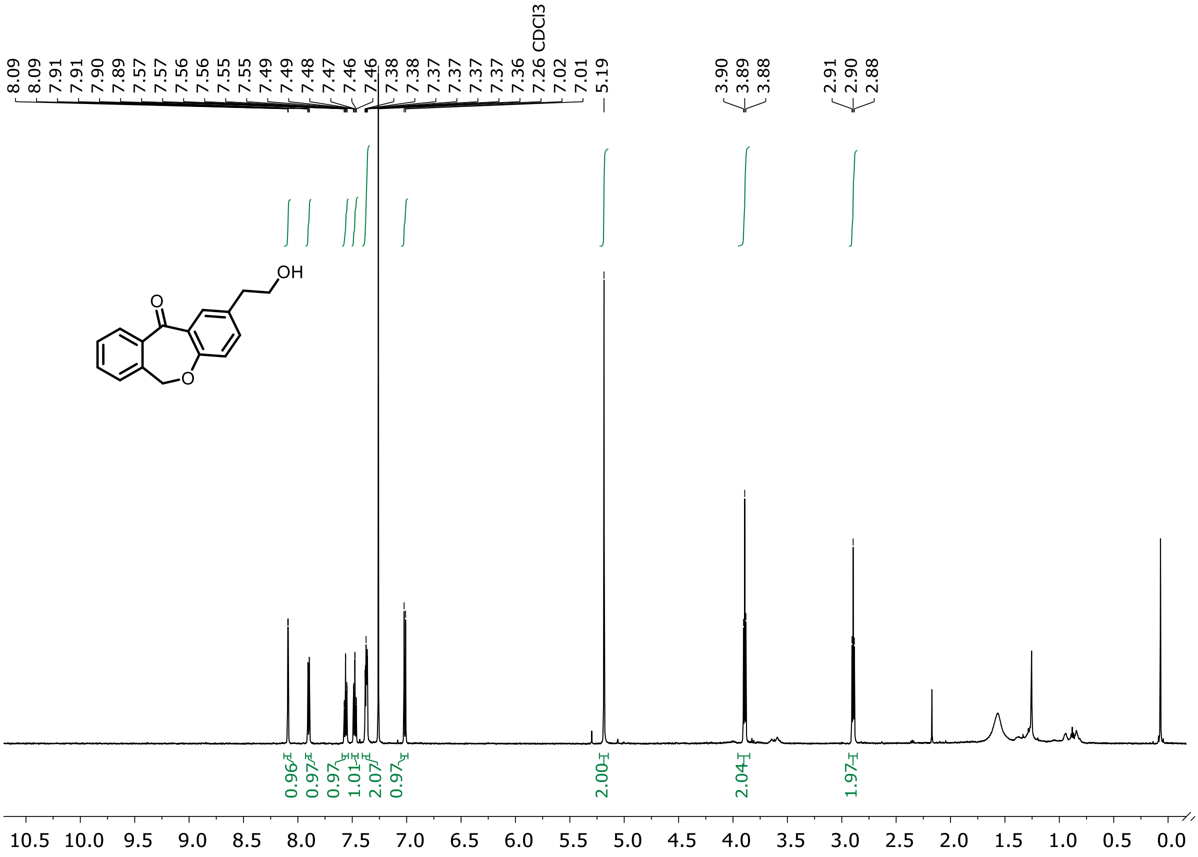

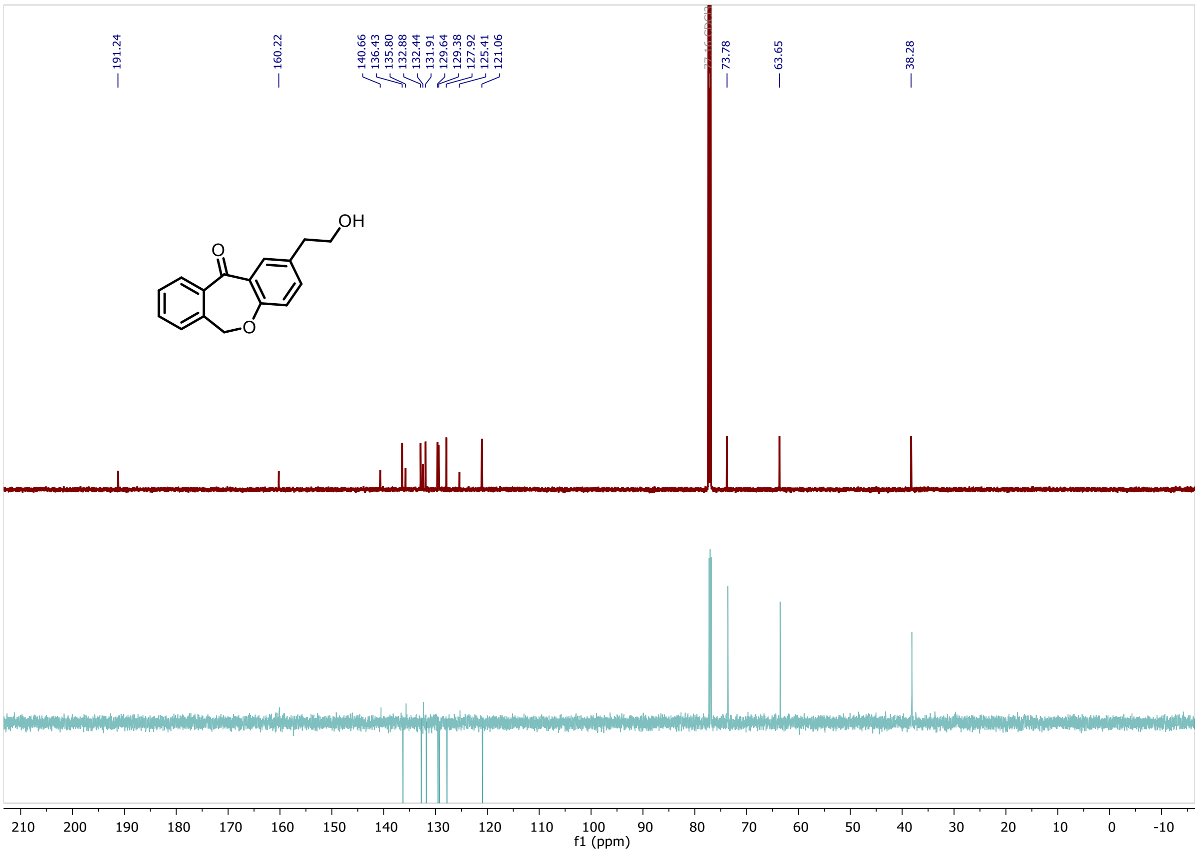

8. References

(1) Gesinski, M. R.; Tadpetch, K.; Rychnovsky, S. D. Symmetric Macrocycles by a Prins Dimerization and Macrocyclization Strategy. *Org. Lett.* **2009**, *11* (22), 5342–5345. https://doi.org/10.1021/ol9022062.

(2) *Pd-Catalyzed β-C(sp3)–H Arylation of Propionic Acid and Related Aliphatic Acids - Ghosh - 2017 - Chemistry – A European Journal - Wiley Online Library*. https://chemistry-europe.onlinelibrary.wiley.com/doi/full/10.1002/chem.201705449 (accessed 2021-12-17).

(3) Larionov, E.; Lin, L.; Guénée, L.; Mazet, C. Scope and Mechanism in Palladium-Catalyzed Isomerizations of Highly Substituted Allylic, Homoallylic, and Alkenyl Alcohols. *J. Am. Chem. Soc.* **2014**, *136* (48), 16882–16894. https://doi.org/10.1021/ja508736u.

(4) Colbon, P.; Ruan, J.; Purdie, M.; Mulholland, K.; Xiao, J. Double Arylation of Allyl Alcohol via a One-Pot Heck Arylation–Isomerization–Acylation Cascade. *Org. Lett.* **2011**, *13* (20), 5456–5459. https://doi.org/10.1021/ol202144z.

(5) Kawasaki, M.; Goto, M.; Kawabata, S.; Kometani, T. The Effect of Vinyl Esters on the Enantioselectivity of the Lipase-Catalysed Transesterification of Alcohols. *Tetrahedron: Asymmetry* **2001**, *12* (4), 585–596. https://doi.org/10.1016/S0957-4166(01)00083-0.

(6) Zhu, D.; Mukherjee, C.; Biehl, E. R.; Hua, L. Nitrilase-Catalyzed Selective Hydrolysis of Dinitriles and Green Access to the Cyanocarboxylic Acids of Pharmaceutical Importance. *Advanced Synthesis & Catalysis* **2007**, *349* (10), 1667–1670. https://doi.org/10.1002/adsc.200700067.

(7) Wang, Z.; Zhu, L.; Yin, F.; Su, Z.; Li, Z.; Li, C. Silver-Catalyzed Decarboxylative Chlorination of Aliphatic Carboxylic Acids. *J. Am. Chem. Soc.* **2012**, *134* (9), 4258–4263. https://doi.org/10.1021/ja210361z.

(8) Jia, Z.; Zhou, F.; Liu, M.; Li, X.; Chan, A. S. C.; Li, C.-J. Silver-Catalyzed Hydrogenation of Aldehydes in Water. *Angewandte Chemie International Edition* **2013**, *52* (45), 11871–11874. https://doi.org/10.1002/anie.201306243.

(9) Fuentes, J. A.; Smith, S. M.; Scharbert, M. T.; Carpenter, I.; Cordes, D. B.; Slawin, A. M. Z.; Clarke, M. L. On the Functional Group Tolerance of Ester Hydrogenation and Polyester Depolymerisation Catalysed by Ruthenium Complexes of Tridentate Aminophosphine Ligands. *Chemistry – A European Journal* **2015**, *21* (30), 10851–10860. https://doi.org/10.1002/chem.201500907.

(10) Bolduc, T. G.; Lee, C.; Chappell, W. P.; Sammis, G. M. Thionyl Fluoride-Mediated One-Pot Substitutions and Reductions of Carboxylic Acids. *J. Org. Chem.* **2022**, *87* (11), 7308–7318. https://doi.org/10.1021/acs.joc.2c00496.

(11) Pfennig, V. S.; Villella, R. C.; Nikodemus, J.; Bolm, C. Mechanochemical Grignard Reactions with Gaseous CO2 and Sodium Methyl Carbonate**. *Angewandte Chemie International Edition* **2022**, *61* (9), e202116514. https://doi.org/10.1002/anie.202116514.

(12) Bisai, M. K.; Das, T.; Vanka, K.; Sen, S. S. Easily Accessible Lithium Compound Catalyzed Mild and Facile Hydroboration and Cyanosilylation of Aldehydes and Ketones. *Chem. Commun.* **2018**, *54* (50), 6843–6846. https://doi.org/10.1039/C8CC02314J.

(13) He, J.; Baldwin, J. E.; Lee, V. Studies towards the Synthesis of the Antibiotic Tetrodecamycin. *Synlett* **2018**, *29* (8), 1117–1121. https://doi.org/10.1055/s-0037-1609303.

(14) Utsumi, T.; Noda, K.; Kawauchi, D.; Ueda, H.; Tokuyama, H. Nitrile Synthesis by Aerobic Oxidation of Primary Amines and in Situ Generated Imines from Aldehydes and Ammonium Salt with Grubbs Catalyst. *Advanced Synthesis & Catalysis* **2020**, *362* (17), 3583–3588. https://doi.org/10.1002/adsc.202000663.

(15) Kriegelstein, M.; Profous, D.; Přibylka, A.; Cankař, P. The Assignment of the Absolute Configuration of β-Chiral Primary Alcohols with Axially Chiral Trifluoromethylbenzimidazolylbenzoic Acid. *J. Org. Chem.* **2020**, *85* (20), 12912–12921. https://doi.org/10.1021/acs.joc.0c01510.

(16) Rashed, Md. N.; Masuda, K.; Ichitsuka, T.; Koumura, N.; Sato, K.; Kobayashi, S. Zirconium Oxide-Catalyzed Direct Amidation of Unactivated Esters under Continuous-Flow Conditions. *Advanced Synthesis & Catalysis* **2021**, *363* (10), 2529–2535. https://doi.org/10.1002/adsc.202001496.

(17) Wei, J.; Zhao, L.; He, C.; Zheng, S.; Reek, J. N. H.; Duan, C. Metal–Organic Capsules with NADH Mimics as Switchable Selectivity Regulators for Photocatalytic Transfer Hydrogenation. *J. Am. Chem. Soc.* **2019**, *141* (32), 12707–12716. https://doi.org/10.1021/jacs.9b05351.
(18) (a) Gaussian 16, Revision C.01, M. J. Frisch, G. W. Trucks, H. B. Schlegel, G. E. Scuseria, M. A. Robb, J. R. Cheeseman, G. Scalmani, V. Barone, G. A. Petersson, H. Nakatsuji, X. Li, M. Caricato, A. V. Marenich, J. Bloino, B. G. Janesko, R. Gomperts, B. Mennucci, H. P. Hratchian, J. V. Ortiz, A. F. Izmaylov, J. L. Sonnenberg, D. Williams-Young, F. Ding, F. Lipparini, F. Egidi, J. Goings, B. Peng, A. Petrone, T. Henderson, D. Ranasinghe, V. G. Zakrzewski, J. Gao, N. Rega, G. Zheng, W. Liang, M. Hada, M. Ehara, K. Toyota, R. Fukuda, J. Hasegawa, M. Ishida, T. Nakajima, Y. Honda, O. Kitao, H. Nakai, T. Vreven, K. Throssell, J. A. Montgomery, Jr., J. E. Peralta, F. Ogliaro, M. J. Bearpark, J. J. Heyd, E. N. Brothers, K. N. Kudin, V. N. Staroverov, T. A. Keith, R. Kobayashi, J. Normand, K. Raghavachari, A. P. Rendell, J. C. Burant, S. S. Iyengar, J. Tomasi, M. Cossi, J. M. Millam, M. Klene, C. Adamo, R. Cammi, J. W. Ochterski, R. L. Martin, K. Morokuma, O. Farkas, J. B. Foresman, D. J. Fox, Gaussian, Inc., Wallingford CT, 2016;
(b) S. Grimme, J. Antony, S. Ehrlich, H. Krieg, *J. Chem. Phys.* **2010**, *132*, 154104
(19) Y. Zhao, D. G. Truhlar, Theor. *Chem. Acc.*, **2008**, *120*, 215-241
(20) M. Cossi, N. Rega, G. Scalmani, V. Barone *J. Comp. Chem.*, **2003**, *24*, 669-681
(21) CYLview20; Legault, C. Y., Université de Sherbrooke, 2020 (http://www.cylview.org)
